# Supplementary material for: Modular access to alkylfluorides via radical decarboxylative-desulfonylative gem-difunctionalization
Source: Nat Commun. 2025 May 20;16:4702. doi: 10.1038/s41467-025-60011-0 (PMC12092675; doi:10.1038/s41467-025-60011-0)
Supplement: Supplementary file 1 — Supplementary Information [file 41467_2025_60011_MOESM1_ESM.pdf]

Supplementary Information for

Modular Access to Alkylfluorides via Radical Decarboxylative-Desulfonylative *gem*-Difunctionalization

Xianjin Wang,[1,#] Haotian Li,[2,#] Yasu Chen,[1] Ziqiang Wang,[1,2] Xinxin Wu,[2] Chen Zhu[1,2,*]

[1] Frontiers Science Center for Transformative Molecules, School of Chemistry and Chemical Engineering, State Key Laboratory of Synergistic Chem-Bio Synthesis, and Shanghai Key Laboratory for Molecular Engineering of Chiral Drugs, Shanghai Jiao Tong University, 800 Dongchuan Road, Shanghai 200240, China

[2] Key Laboratory of Organic Synthesis of Jiangsu Province, College of Chemistry, Chemical Engineering and Materials Science, Soochow University, 199 Ren-Ai Road, Suzhou, Jiangsu 215123, China

[#] These authors contributed equally

[*]Correspondence to: chzhu@sjtu.edu.cn

Contents

1. General experimental details 3

2. General procedures for preparing α-sulfonyl carboxylic acids 3

2.1 Method A 3

2.2 Method B 5

3. Characterization of products 9

4. Product Transformations 23

4.1 Synthesis of 4 23

4.2 Synthesis of 5 24

4.3 Synthesis of 6 25

4.4 Synthesis of 7 25

4.5 Synthesis of 8 26

5. Stern-Volmer Studies 26

6. UV-vis experiments 27

7. Cyclic voltammograms 28

8. Quantum yield measurements 28

9. ^{1}H, ^{19}F, ^{13}C NMR spectra 30

10. References 123

1. General experimental details

All reactions were maintained under a nitrogen atmosphere unless otherwise stated. Commercially available reagents were used without further purification. DMF was distilled from CaH under reduced pressure, and DCM was distilled from CaH. Fourier tansform infrared (FT-IR) spectra were recorded on a BRUKER VERTEX 70, ν_{max} in cm^{-1}. ^{1}H-NMR spectra were recorded on a BRUKER AVANCE III HD (400 MHz) spectrometer. Chemical shifts are reported in ppm from tetramethylsilane with the solvent resonance as internal standard ($CDCl_3$: δ 7.26, DMSO-d^6: δ 2.50). Data are reported as follows: chemical shift, multiplicity (s = singlet, d = doublet, t = triplet, q = quadruplet, br = broad, m = multiplet), coupling constants (Hz) and integration. ^{13}C-NMR spectra were recorded on a BRUKER AVANCE III HD (100 MHz) spectrometer with complete proton decoupling. Chemical shifts are reported in ppm from tetramethylsilane with the solvent resonance as the internal standard ($CDCl_3$: δ 77.00, DMSO-d^6: δ 40.0). ^{19}F-NMR spectra were recorded on a BRUKER AVANCE III HD (376 MHz) spectrometer. Mass spectra were measured with an Agilent Technologies 6120 Quadrupole LC/MS. High resolution mass spectrometry (HRMS) were measured with a GCT Premier™ and BRUKER micrOTF-Q III. Melting points were measured using INESA WRR and values are uncorrected.

2. General procedures for preparing *α*-sulfonyl carboxylic acids

2.1 Method A

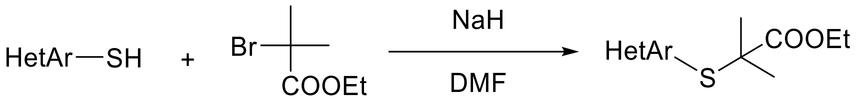

First Step: A dry flask was evacuated and backfilled with N_2 for 3 times, where thiophenol (30 mmol) and NaH (36 mmol) in DMF (100 mL) was added at 0 °C. The mixture was stirred for 20 min. Then alkyl iodide (36 mmol) was added dropwise by syringe at 0 °C and the mixture was stirred at rt for 12 h. After the reaction was complete, the mixture was quenched by aq. NH_4Cl at 0 °C. The mixture was extracted with Et_2O for 3 times. Then the organic phase was combined and dried over anhydrous $MgSO_4$. The solvent was removed under vacuum to afford the crude product which was used in next step without further purification.

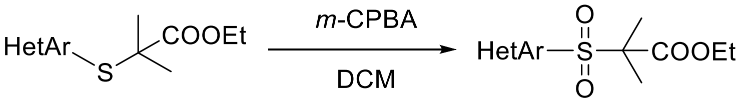

Second Step: A stirred solution of the starting material (20 mmol) in DCM (100 mL) was added *m*-CPBA (3 equiv., 60 mmol) slowly at 0 °C. Then the reaction mixture was warmed to room temperature and stirred for 12 h. After the reaction was complete, the mixture was added aq. $NaHCO_3$ and stirred for 1 h at 0 °C. After filtration through a pad of celite, the residue was washed with DCM. The organic phase was combined and dried over anhydrous $MgSO_4$. The solvent was removed under vacuum and the residue was purified by flash column chromatography on silica gel to provide the product.

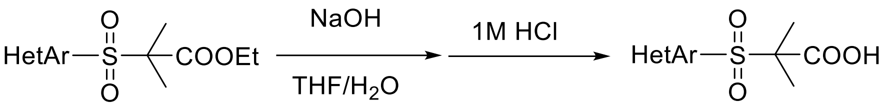

Third step: The starting material (10 mmol) was dissolved in THF (20 mL) at 0 °C. Then NaOH (30 mmol) dissolved in 20 mL H_2O was added slowly. The reaction mixture was warmed to room temperature. After monitoring the complete consumption of sulfone by TLC, the solution was adjusted to pH=1 with 1 M hydrochloric acid. The mixture was extracted with EtOAc, and the combined organic layers were washed with brine, dried over anhydrous $MgSO_4$, and concentrated in vacuo to give the crude product which was further purified by recrystallization in DCM/ Petroleum ether cosolvent.

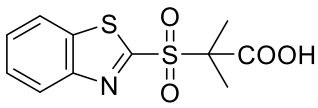

2a

2a: white solid, m.p. 152-153 °C. **^{1}H NMR (400 MHz, DMSO-*d*$_6$)** δ 8.37-8.32 (m, 1H), 8.32-8.27 (m, 1H), 7.77-7.67 (m, 2H), 1.70 (s, 6H); **^{13}C NMR (100 MHz, DMSO-*d*$_6$)** δ 169.3, 164.7, 152.6, 137.3, 128.8, 128.4, 125.6, 123.8, 70.6, 20.2. **FT-IR**: ν (cm^{-1}) 2987, 2901, 1716, 1436, 1333, 1250, 1066. **HRMS [ESI]** calcd for $C_{11}H_{11}NO_4S_2Na^+$ $[M+Na]^+$ 308.0027, found 308.0026.

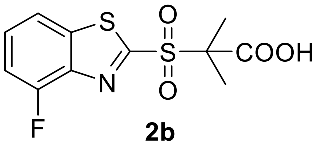

2b

2b: white solid, m.p. 159-160 °C. **^{1}H NMR (400 MHz, DMSO-*d*$_6$)** δ 8.22-8.14 (m, 1H), 7.81-7.69 (m, 1H), 7.67-7.54 (m, 1H), 1.71 (s, 6H); **^{13}C NMR (100 MHz, DMSO-*d*$_6$)** δ 169.2, 165.6, 156.4 (d, $J_{C\text{-}F}$ = 257.6 Hz), 141.6 (d, $J_{C\text{-}F}$ = 14.4 Hz), 139.8 (d, $J_{C\text{-}F}$ = 2.5 Hz), 130.1 (d, $J_{C\text{-}F}$ = 5.9 Hz), 120.0, 113.7 (d, $J_{C\text{-}F}$ = 15.7 Hz), 70.8, 20.2; **^{19}F NMR** (376 MHz, DMSO-*d*$_6$) δ -120.5 (s). **FT-IR**: ν (cm^{-1}) 2987, 2901, 1684, 1507, 1394, 1250, 1075. **HRMS [ESI]** calcd for $C_{11}H_{11}FNO_4S_2^+$ $[M+H]^+$ 304.0108, found 304.0109.

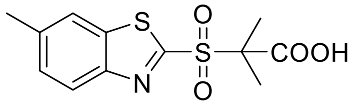

2c

2c: white solid, m.p. 152-153 °C. **^{1}H NMR (400 MHz, DMSO-*d*$_6$)** δ 8.16 (d, *J* = 8.4 Hz, 1H), 8.12 (s, 1H), 7.55 (dd, *J* = 8.4, 1.2 Hz, 1H), 2.51 (s, 3H), 1.68 (s, 6H); **^{13}C NMR (100 MHz, DMSO-*d*$_6$)** δ 169.3, 163.2, 150.9, 139.1, 137.6, 130.1, 125.1, 122.9, 70.5, 21.8, 20.2. **FT-IR**: ν (cm^{-1}) 2987, 2901, 1716, 1699, 1456, 1328, 1241. **HRMS [ESI]** calcd for $C_{12}H_{13}NO_4S_2Na^+$ $[M+Na]^+$ 322.0184, found 322.0178.

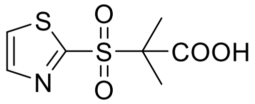

2d

2d: white solid, m.p. 144-145 °C. **^{1}H NMR (400 MHz, DMSO-*d*$_6$)** δ 8.37 (d, *J* = 3.2 Hz, 1H), 8.22 (d, *J* = 3.2 Hz, 1H), 1.61 (s, 6H); **^{13}C NMR (100 MHz, DMSO-*d*$_6$)** δ 169.4, 162.8, 145.8, 130.1, 70.1, 20.3. **FT-IR**: ν (cm^{-1}) 2987, 2901, 1684, 1395, 1321, 1152. **HRMS [ESI]** calcd for $C_7H_9NO_4S_2Na^+$ $[M+Na]^+$ 257.9865, found 257.9866.

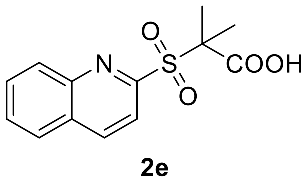

2e

2e: white solid, m.p. 133-134 °C. **^{1}H NMR (400 MHz, DMSO-*d*$_6$)** δ 8.74 (d, *J* = 8.8 Hz, 1H), 8.22-8.15 (m, 2H), 8.07 (d, *J* = 8.8 Hz, 1H), 8.01-7.93 (m, 1H), 7.89-7.81 (m, 1H), 1.64 (s, 6H); **^{13}C NMR (100 MHz, DMSO-*d*$_6$)** δ 170.1, 155.6, 146.7, 139.4, 132.1, 130.1, 130.1, 129.3, 128.8, 120.6, 69.3, 20.6. **FT-IR**: ν (cm^{-1}) 3178, 2988, 2907, 1728, 1656, 1473, 1419, 1295, 1116. **HRMS [ESI]** calcd for $C_{13}H_{13}NO_4SNa^+$ $[M+Na]^+$ 302.0458, found 322.0467.

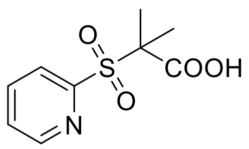

2f

2f: white solid, m.p. 149-150 °C. **^{1}H NMR (400 MHz, DMSO-*d*$_6$)** δ 8.78 (d, *J* = 4.0 Hz, 1H), 8.14 (td, *J* = 8.0, 1.6 Hz, 1H), 8.06-7.98 (m, 1H), 7.79-7.72 (m, 1H), 1.56 (s, 6H); **^{13}C NMR (100 MHz, DMSO-*d*$_6$)** δ 170.0, 155.6, 150.5, 139.1, 128.7, 125.6, 69.0, 20.6. **FT-IR**: ν (cm^{-1}) 3092, 2987, 2901, 1749, 1489, 1394, 1250. **HRMS [ESI]** calcd for $C_9H_{11}NO_4SNa^+$ [M+Na]$^+$ 252.0301, found 253.0302.

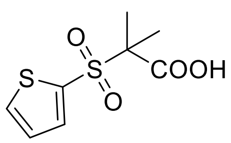

2g

2g: white solid, m.p. 141-143 °C. **^{1}H NMR (500 MHz, DMSO-*d*$_6$)** δ 8.31-8.00 (m, 1H), 7.81-7.60 (m, 1H), 7.29 (dd, *J* = 5.0, 4.0 Hz, 1H), 1.51 (s, 6H); **^{13}C NMR (125 MHz, DMSO-*d*$_6$)** δ 169.9, 137.4, 137.2, 136.1, 128.6, 69.4, 20.5. **FT-IR**: ν (cm^{-1}) 2987, 2901, 1684, 1395, 1321, 1152, 950. **HRMS [ESI]** calcd for $C_9H_{11}NO_4SNa^+$ [M+Na]$^+$ 256.9913, found 256.9908.

2.2 Method B

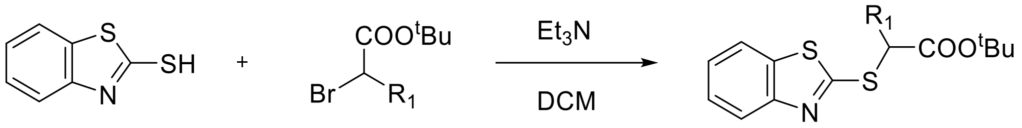

First Step: A dry flask was evacuated and backfilled with N_2 for 3 times, where thiophenol (30 mmol) was dissolved in DCM (50 mL). Then Et_3N (9 mL) was added slowly and stirred for 30 min, followed by the addition of easter (30 mmol). The reaction was stirred for another 3 h. After the reaction was complete, the solvent was removed under vacuum. Then H_2O was added to the mixture, which was extracted with EtOAc for two times. The organic layers were combined and dried over anhydrous $MgSO_4$. The solvent was removed under vacuum to afford the crude product which was used in next step without further purification.

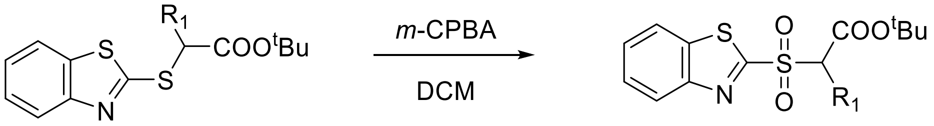

Second Step: A stirred solution of the starting material (20 mmol) in DCM (100 mL) was added *m*-CPBA (3 equiv., 60 mmol) slowly at 0 °C. The reaction mixture was warmed to room temperature and stirred for 12 h. After the reaction was complete, the mixture was added aq. $NaHCO_3$ and stirred for 1 h at 0 °C. After filtration through a pad of celite, the residue was washed with DCM. The organic layers were combined and dried over anhydrous $MgSO_4$. The solvent was removed under vacuum and the residue was purified by flash column chromatography on silica gel to provide the product.

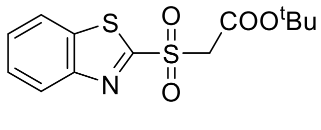

S1

S1: white solid, m.p. 110-115 °C. Purification by flash column chromatography (eluent: EtOAc/Petroleum ether = 1/10). **^{1}H NMR (400 MHz, $CDCl_3$)** δ 8.25-8.19 (m, 1H), 8.05-8.00 (m, 1H), 7.68-7.57 (m, 2H), 4.49 (s, 2H), 1.33 (s, 9H); **^{13}C NMR (100 MHz, $CDCl_3$)** δ 165.2, 160.4, 152.4, 136.8, 128.2, 127.7, 125.5, 122.3, 84.3, 59.9, 27.6. **FT-IR**: ν (cm^{-1}) 2981, 2930, 1737, 1469, 1336, 1273, 1152, 1123. **HRMS [ESI]** calcd for $C_{13}H_{15}NO_4S_2Na^+$

[M+Na]+ 336.0335, found 336.0335.

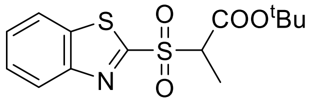

S2

S2: white solid, m.p. 100-101 °C. Purification by flash column chromatography (eluent: EtOAc/Petroleum ether = 1/20). **^{1}H NMR (400 MHz, $CDCl_3$)** δ 8.21 (d, *J* = 8.0 Hz, 1H), 8.01 (d, *J* = 8.0 Hz, 1H), 7.69-7.54 (m, 2H), 4.57 (q, *J* = 7.2 Hz, 1H), 1.74 (d, *J* = 7.2 Hz, 3H), 1.29 (s, 9H); **^{13}C NMR (100 MHz, $CDCl_3$)** δ 164.9, 163.9, 152.6, 136.8, 128.1, 127.6, 125.4, 122.2, 83.9, 64.9, 27.5, 10.6. **FT-IR**: ν (cm^{-1}) 2974, 2935, 1732, 1457, 1327, 1255. **HRMS [ESI]** calcd for $C_{14}H_{17}NO_4S_2Na^+$ [M+Na]+ 350.0491, found 350.0493.

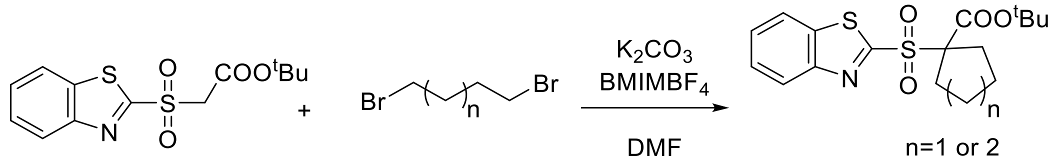

Third Step (A): S1 (5 mmol) was dissolved in DMF (20 mL), then added dibromoalkane (5 mmol 1 equiv) and K_2CO_3 (6 mmol 1.2 equiv), 1-Butyl-3-methylimidazolium tetrafluoroborate (0.5 mmol 0.1 equiv) was added slowly. Stirred for 12 h. After the reaction was complete, the mixture was extracted with Et_2O for 3 times. Then the organic phase was combined and dried over anhydrous $MgSO_4$. The solvent was removed under vacuum and the residue was purified by flash column chromatography on silica gel to provide the product.

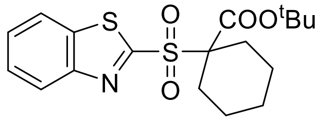

S3

S3: white solid, m.p. 130-131 °C. Purification by flash column chromatography (eluent: EtOAc/Petroleum ether = 1/20). **^{1}H NMR (400 MHz, $CDCl_3$)** δ 8.23 (d, *J* = 8.0 Hz, 1H), 7.99 (d, *J* = 8.0 Hz, 1H), 7.70-7.49 (m, 2H), 2.72-2.49 (m, 2H), 2.09-1.95 (m, 2H), 1.92-1.79 (m, 2H), 1.72-1.63 (m, 1H),1.38 (s, 9H), 1.33-1.22 (m, 3H); **^{13}C NMR (100 MHz, $CDCl_3$)** δ 165.2, 163.2, 152.7, 137.4, 127.9, 127.5, 125.7, 122.0, 83.5, 75.7, 28.0, 27.6, 24.5, 22.9. **FT-IR**: ν (cm^{-1}) 2932, 2866, 1716, 1455, 1326, 1309, 1238, 1122. **HRMS [ESI]** calcd for $C_{18}H_{23}NO_4S_2Na^+$ [M+Na]+ 404.0960, found 404.0961.

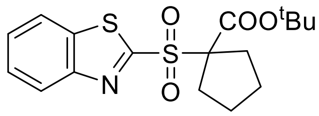

S4

S4: white solid, m.p. 90-91 °C. Purification by flash column chromatography (eluent: EtOAc/Petroleum ether = 1/20). **^{1}H NMR (400 MHz, $CDCl_3$)** δ 8.26-8.17 (m, 1H), 8.04-7.96 (m, 1H), 7.66-7.53 (m, 2H), 2.85-2.73 (m, 2H), 2.54-2.44 (m, 2H), 1.99-1.86 (m, 2H), 1.80-1.67 (m, 2H), 1.29 (s, 9H); **^{13}C NMR (100 MHz, $CDCl_3$)** δ 166.9, 165.4, 152.7, 137.0, 127.8, 127.4, 125.5, 122.0, 83.5, 80.3, 32.8, 27.5, 25.7. **FT-IR: ν (cm^{-1})** 2970, 2930, 2870, 1731, 1457, 1316, 1125. **HRMS [ESI]** calcd for $C_{17}H_{21}NO_4S_2Na^+$ [M+Na]+ 390.0804, found 390.0803.

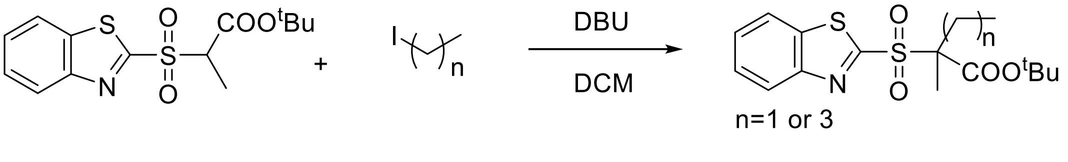

Third Step (B): S2 (5 mmol) was dissolved in DCM (20 mL), then DBU (7.5 mmol) and iodoalkane

(7.5 mmol) were added slowly to the solution. The reaction was stirred for 6 h. After the reaction was complete, the solvent was removed under vacuum and the residue was purified by flash column chromatography on silica gel to provide the product.

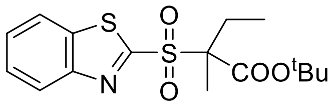

S5

S5: white solid, m.p. 74-75 ºC. Purification by flash column chromatography (eluent: EtOAc/Petroleum ether = 1/20). **^{1}H NMR (400 MHz, CDCl$_3$)** δ 8.23 (d, J = 8.0 Hz, 1H), 8.03-7.93 (m, 1H), 7.66-7.54 (m, 2H), 2.66-2.52 (m, 1H), 2.18-2.06 (m, 1H), 1.71 (s, 3H), 1.36 (s, 9H), 0.99 (t, J = 7.6 Hz, 3H); **^{13}C NMR (100 MHz, CDCl$_3$)** δ 165.9, 164.2, 152.8, 137.2, 127.9, 127.5, 125.7, 122.0, 83.7, 75.6, 27.6, 26.6, 15.8, 8.5. **FT-IR**: ν (cm^{-1}) 2976, 2942, 1716, 1469, 1456, 1325, 1129. **HRMS [ESI]** calcd for $C_{16}H_{21}NO_4S_2Na^+$ [M+Na]$^+$ 378.0804, found 378.0803.

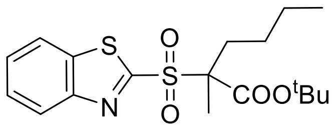

S6

S6: white solid, m.p. 65-66 ºC. Purification by flash column chromatography (eluent: EtOAc/Petroleum ether = 1/20). **^{1}H NMR (400 MHz, CDCl$_3$)** δ 8.27-8.20 (m, 1H), 8.03-7.97 (m, 1H), 7.66-7.54 (m, 2H), 2.57-2.45 (m, 1H), 2.12-2.01 (m, 1H), 1.72 (s, 3H), 1.49-1.39 (m, 3H), 1.37 (s, 9H) 1.22-1.10 (m, 1H), 0.92 (t, J = 7.2 Hz, 3H); **^{13}C NMR (100 MHz, CDCl$_3$)** δ 166.0, 164.1, 152.8, 137.3, 127.9, 127.5, 125.7, 122.0, 83.7, 75.2, 32.6, 27.6, 26.3, 22.8, 16.4, 13.8. **FT-IR**: ν (cm^{-1}) 2953, 2910, 2866, 1716, 1456, 1312, 1237, 1145. **HRMS [ESI]** calcd for $C_{18}H_{25}NO_4S_2Na^+$ [M+Na]$^+$ 406.1117, found 406.1114.

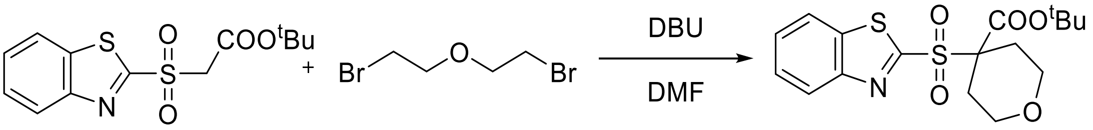

Third Step (C): S1 (5 mmol) was dissolved in DMF (20 mL), then DBU (5 mmol 1.0 equiv) and 2,2'-dibromodiethyl ether (5 mmol) were added slowly. The reaction mixture was stirred at 85 °C for 3 hours. After the reaction was complete, the mixture was extracted with Et_2O for 3 times. Then the organic layers were combined and dried over anhydrous $MgSO_4$. The solvent was removed under vacuum and the residue was purified by flash column chromatography on silica gel to provide the product.

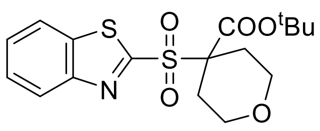

S7

S7: white solid, m.p. 125-126 ºC. Purification by flash column chromatography (eluent: EtOAc/Petroleum ether = 1/10). **^{1}H NMR (400 MHz, CDCl$_3$)** δ 8.29-8.21 (m, 1H), 8.06-7.97 (m, 1H), 7.69-7.56 (m, 2H), 4.10-4.01 (m, 2H), 3.44-3.34 (m, 2H), 2.53-2.37 (m, 4H), 1.39 (s, 9H); **^{13}C NMR (100 MHz, CDCl$_3$)** δ 164.6, 162.6, 152.8, 137.3, 128.2, 127.7, 125.7, 122.1, 84.4, 73.1, 64.7, 28.2, 27.6. **FT-IR**: ν (cm^{-1}) 2981, 2930, 2860, 1732, 1458, 1330, 1241, 1151, 1121. **HRMS [ESI]** calcd for $C_{17}H_{21}NO_5S_2Na^+$ [M+Na]$^+$ 406.0753, found 406.0756.

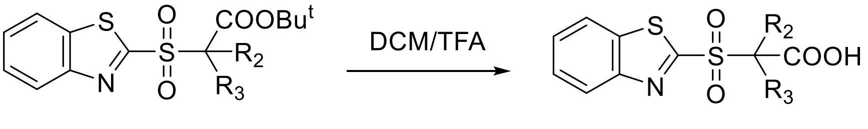

Fourth Step: The starting material (5 mmol) was dissolved in DCM (10 mL). TFA (10 mL) was added slowly to the reaction mixture, which was then stirred for 2h. The mixture was concentrated in vacuo to give the crude acid, which was further purified by recrystallization in DCM/Petroleum ether.

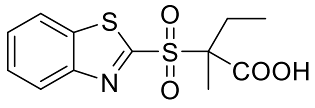

2h

2h: from **S5**, white solid, m.p. 140-141°C. **^{1}H NMR (400 MHz, DMSO-*d*$_6$)** δ 8.38-8.32 (m, 1H), 8.32-8.26 (m, 1H), 7.78-7.66 (m, 2H), 2.47-2.36 (m, 1H), 2.06-1.93 (m, 1H), 1.61 (s, 3H), 0.93 (t, *J* = 7.2 Hz, 3H); **^{13}C NMR (100 MHz, DMSO-*d*$_6$)** δ 168.5, 164.7, 152.6, 137.4, 128.8, 128.4, 125.6, 123.8, 75.0, 26.4, 16.1, 9.0. **FT-IR**: ν (cm^{-1}) 2978, 2941, 2867, 1710, 1457, 1326, 1237, 1150. **HRMS [ESI]** calcd for $C_{12}H_{13}NO_4S_2Na^+$ [M+Na]$^+$ 322.0184, found 322.0178.

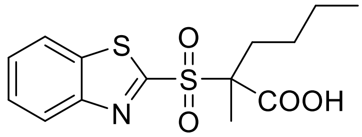

2i

2i: from **S6**, white solid, m.p. 104-105 °C. **^{1}H NMR (400 MHz, DMSO-*d*$_6$)** δ 8.38-8.32 (m, 1H), 8.32-8.26 (m, 1H), 7.77-7.66 (m, 2H), 2.41-2.27 (m, 1H), 2.01-1.89 (m, 1H), 1.62 (s, 3H), 1.48-1.24 (m, 3H), 1.18-1.05 (m, 1H), 0.86 (t, *J* = 7.2 Hz, 3H); **^{13}C NMR (100 MHz, DMSO-*d*$_6$)** δ 168.6, 164.6, 152.6, 137.4, 128.8, 128.4, 125.6, 123.8, 74.5, 32.6, 26.3, 22.8, 16.7, 14.1. **FT-IR**: ν (cm^{-1}) 2952, 2930, 2870, 1714, 1469, 1312, 1148, 1117. **HRMS [ESI]** calcd for $C_{14}H_{18}NO_4S_2^+$ [M+H]$^+$ 328.0671, found 328.0671.

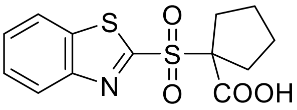

2j

2j: from **S4**, white solid, m.p. 131-132 °C. **^{1}H NMR (400 MHz, DMSO-*d*$_6$)** δ 8.39-8.31 (m, 1H), 8.31-8.24 (m, 1H), 7.77-7.65 (m, 2H), 2.69-2.57 (m, 2H), 2.45-2.33 (m, 2H), 1.87-1.74 (m, 2H), 1.73-1.62 (m, 2H); **^{13}C NMR (100 MHz, DMSO-*d*$_6$)** δ 169.5, 166.0, 152.6, 137.1, 128.7, 128.4, 125.5, 123.8, 79.7, 32.7, 26.0. **FT-IR**: ν (cm^{-1}) 2885, 2574, 2277, 1711, 1466, 1334, 1131. **HRMS [ESI]** calcd for $C_{13}H_{13}NO_4S_2Na^+$ [M+Na]$^+$ 334.0178, found 334.0179.

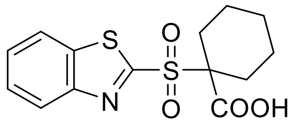

2k

2k: from **S3**, white solid, m.p. 150-151 °C. **^{1}H NMR (400 MHz, DMSO-*d*$_6$)** δ 8.38-8.32 (m, 1H), 8.32-8.26 (m, 1H), 7.80-7.62 (m, 2H), 2.50-2.42 (m, 2H), 1.94-1.70 (m, 4H), 1.64-1.53 (m, 1H), 1.31-1.12 (m, 3H); **^{13}C NMR (100 MHz, DMSO-*d*$_6$)** δ 167.7, 163.6, 152.7, 137.5, 128.8, 128.4, 125.6, 123.8, 75.1, 28.0, 24.4, 23.0. **FT-IR**: ν (cm^{-1}) 2980, 2939, 2867, 1716, 1455, 1326, 1145, 1128. **HRMS [ESI]** calcd for $C_{14}H_{15}NO_4S_2Na^+$ [M+Na]$^+$ 348.0335, found 348.0325.

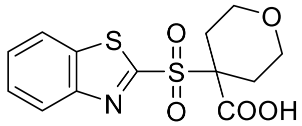

2l

2l: from **S7**, white solid, m.p. 172-173 °C. **^{1}H NMR (400 MHz, DMSO-*d*$_6$)** δ 8.40-8.34 (m, 1H), 8.34-8.27 (m, 1H), 7.87-7.63 (m, 2H), 4.05-3.96 (m, 2H), 3.30-3.20 (m, 2H), 2.39-2.28 (m, 2H), 2.23-2.10 (m, 2H); **^{13}C NMR (100 MHz, DMSO-*d*$_6$)** δ 167.2, 163.2, 152.7, 137.5, 128.9, 128.5, 125.7, 123.8, 72.7, 64.4, 28.2. **FT-IR**: ν (cm^{-1}) 2977, 2939, 2872, 1731, 1467, 1325, 1156, 1128. **HRMS [ESI]** calcd for $C_{13}H_{13}NO_5S_2Na^+$ [M+Na]$^+$ 350.0133, found 350.0141.

3. Characterization of products

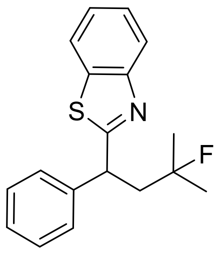

3a

3a: yellow oil. Purification by flash column chromatography on silica gel (eluent: EtOAc/Petroleum ether = 1/200). **^{1}H NMR (400 MHz, $CDCl_3$)** δ 8.04-7.99 (m, 1H), 7.81-7.75 (m, 1H), 7.47-7.41 (m, 3H), 7.36-7.29 (m, 3H), 7.28-7.22 (m, 1H), 4.70 (t, J = 6.4 Hz, 1H), 3.13-2.99 (m, 1H), 2.59-2.46 (m, 1H), 1.33 (d, J = 21.6 Hz, 3H), 1.31 (d, J = 21.6 Hz, 3H); **^{13}C NMR (100 MHz, $CDCl_3$)** δ 175.0, 153.0, 142.5, 135.4, 128.8, 128.0, 127.3, 125.8, 124.8, 122.9, 121.5, 95.2 (d, J_{C-F} = 166.1 Hz), 46.4 (d, J_{C-F} = 4.2 Hz), 46.3 (d, J_{C-F} = 22.5 Hz), 27.6 (d, J_{C-F} = 24.4 Hz), 27.1 (d, J_{C-F} = 24.4 Hz); **^{19}F NMR (376 MHz, $CDCl_3$)** δ -136.8 (s). **FT-IR**: ν (cm^{-1}) 3062, 2978, 1685, 1558, 1512, 1454, 1222. **HRMS [ESI]** calcd for $C_{18}H_{18}FNSNa^+$ $[M+Na]^+$ 322.1036, found 322.1031.

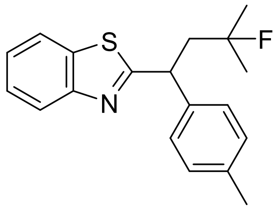

3b

3b: white solid, m.p. 69-70 °C. Purification by flash column chromatography on silica gel (eluent: EtOAc/Petroleum ether = 1/200). **^{1}H NMR (400 MHz, $CDCl_3$)** δ 8.01 (d, J = 8.4 Hz, 1H), 7.77 (d, J = 8.0 Hz, 1H), 7.47-7.41 (m, 1H), 7.34-7.28 (m, 3H), 7.14 (d, J = 8.0 Hz, 2H), 4.66 (t, J = 6.4 Hz, 1H), 3.12-2.96 (m, 1H), 2.57-2.45 (m, 1H), 2.32 (s, 3H), 1.36 (d, J = 21.2 Hz, 3H), 1.31 (d, J = 21.2 Hz, 3H); **^{13}C NMR (100 MHz, $CDCl_3$)** δ 175.4, 153.1, 139.6, 137.0, 135.4, 129.5, 127.9, 125.8, 124.7, 122.9, 121.5, 96.2 (d, J_{C-F} = 166.1 Hz), 46.3 (d, J_{C-F} = 22.4 Hz), 46.0 (d, J_{C-F} = 4.3 Hz), 27.6 (d, J_{C-F} = 24.3 Hz), 27.1 (d, J_{C-F} = 24.4 Hz), 21.0; **^{19}F NMR (376 MHz, $CDCl_3$)** δ -136.6 (s). **FT-IR**: ν (cm^{-1}) 2987, 2901, 1406, 1384, 1254, 1075. **HRMS [ESI]** calcd for $C_{19}H_{20}FNSNa^+$ $[M+Na]^+$ 336.1193, found 336.1193.

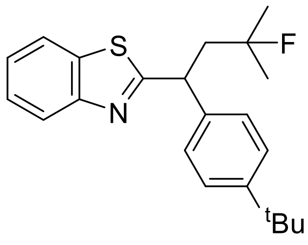

3c

3c: yellow solid, m.p. 76-77 °C. Purification by flash column chromatography on silica gel (eluent: EtOAc/Petroleum ether = 1/200). **^{1}H NMR (400 MHz, $CDCl_3$)** δ 8.01 (d, J = 8.0 Hz, 1H), 7.78 (d, J = 8.0 Hz, 1H), 7.44 (t, J = 8.0 Hz, 1H), 7.38-7.28 (m, 5H), 4.68 (dd, J = 6.8 Hz, 6.0 Hz, 1H), 3.05 (ddd, J = 22.4, 14.8, 7.6 Hz, 1H), 2.50 (ddd, J = 17.6, 14.8, 5.6 Hz, 1H), 1.36 (d, J = 21.6 Hz, 3H), 1.30 (d, J = 21.6 Hz, 3H), 1.29 (s, 9H); **^{13}C NMR (100 MHz, $CDCl_3$)** δ 175.3, 153.1, 150.1, 139.5, 135.4, 127.6, 125.9, 125.8, 124.8, 122.9, 121.5, 95.3 (d, J_{C-F} = 166.0 Hz), 46.5 (d, J_{C-F} = 22.6 Hz), 45.9 (d, J_{C-F} = 4.3 Hz), 34.5, 31.4, 27.6 (d, J_{C-F} = 24.3 Hz), 27.1 (d, J_{C-F} = 24.5 Hz); **^{19}F NMR** (376 MHz, $CDCl_3$) δ -136.6 (s). **FT-IR**: ν (cm^{-1}) 2987, 2922, 1698, 1576, 1456, 1373, 1241. **HRMS [ESI]** calcd for $C_{22}H_{27}FNS^+$ $[M+H]^+$ 356.1843, found 356.1838.

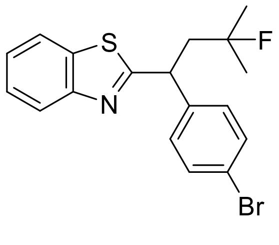

3d

3d: white solid, m.p. 87-88 °C. Purification by flash column chromatography on silica gel (eluent: EtOAc/Petroleum ether = 1/100). **^{1}H NMR (400 MHz, $CDCl_3$)** δ 8.00 (d, J = 8.0 Hz, 1H), 7.79 (d, J = 8.0 Hz, 1H), 7.49-7.42 (m, 3H), 7.37-7.28 (m, 3H), 4.66 (t, J = 6.8 Hz, 1H), 3.10-2.94 (m, 1H), 2.53-2.40 (m, 1H), 1.35 (d, J = 21.2 Hz, 3H), 1.30 (d, J = 21.2 Hz, 3H); **^{13}C NMR (100 MHz, $CDCl_3$)** δ 174.1, 153.0, 141.6, 135.2, 132.0, 130.0, 126.0, 125.0, 123.0, 121.5, 121.2, 95.0 (d, J_{C-F} = 174.8 Hz), 46.3 (d, J_{C-F} = 22.4 Hz), 45.7 (d, J_{C-F} = 3.8 Hz), 27.8 (d, J_{C-F} = 22.4 Hz), 27.0 (d, J_{C-F} = 24.5 Hz); **^{19}F NMR (376 MHz, $CDCl_3$)** δ -137.4 (s). **FT-IR**: ν (cm^{-1}) 2987, 2973, 2901, 1551, 1456, 1384, 1250. **HRMS**

[ESI] calcd for $C_{18}H_{17}BrFNS^+$ $[M+H]^+$ 377.0244, found 377.0245.

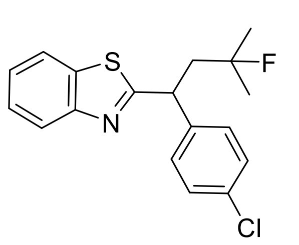

3e

3e: white solid, m.p. 91-92 ºC. Purification by flash column chromatography on silica gel (eluent: EtOAc/Petroleum ether = 1/200). **^{1}H NMR (400 MHz, $CDCl_3$)** δ 8.01 (d, *J* = 8.4 Hz, 1H), 7.79 (d, *J* = 7.6 Hz, 1H), 7.45 (t, *J* = 7.6 Hz, 1H), 7.39-7.27 (m, 5H), 4.67 (t, *J* = 6.8 Hz, 1H), 3.09-2.95 (m, 1H), 2.53-2.40 (m, 1H), 1.36 (d, *J* = 21.6 Hz, 3H), 1.30 (d, *J* = 21.6 Hz, 3H); **^{13}C NMR (100 MHz, $CDCl_3$)** δ 174.2, 153.0, 141.0, 135.2, 133.1, 129.4, 129.0, 126.0, 124.9, 122.9, 121.5, 95.1 (d, $J_{C\text{-}F}$ = 166.4 Hz), 46.3 (d, *J* = 22.3 Hz), 45.6 (d, $J_{C\text{-}F}$ = 4.0 Hz), 27.8 (d, $J_{C\text{-}F}$ = 24.3 Hz), 26.9 (d, $J_{C\text{-}F}$ = 24.5 Hz); **^{19}F NMR (376 MHz, $CDCl_3$)** δ -137.3 (s). **FT-IR**: ν (cm^{-1}) 2987, 2901, 1339, 1250, 1229, 1066. **HRMS [ESI]** calcd for $C_{18}H_{17}ClFNS^+$ $[M+H]^+$ 333.0749, found 333.0749.

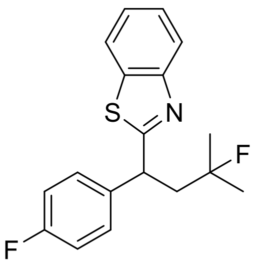

3f

3f: white solid, m.p. 60-61 ºC. Purification by flash column chromatography on silica gel (eluent: EtOAc/Petroleum ether = 1/200). **^{1}H NMR (400 MHz, $CDCl_3$)** δ 8.01 (d, *J* = 8.0 Hz, 1H), 7.79 (d, *J* = 7.6 Hz, 1H), 7.49-7.30 (m, 4H), 7.06-6.97 (m, 2H), 4.68 (t, *J* = 6.4 Hz, 1H), 3.10-2.95 (m, 1H), 2.54-2.41 (m, 1H), 1.35 (d, *J* = 21.6 Hz, 3H), 1.30 (d, *J* = 21.6 Hz, 3H); **^{13}C NMR (100 MHz, $CDCl_3$)** δ 174.7, 162.0 (d, $J_{C\text{-}F}$ = 244.4 Hz), 153.1, 138.4, 135.3, 129.6 (d, $J_{C\text{-}F}$ = 8.0 Hz), 126.0, 124.9, 123.0, 121.6, 115.7 (d, $J_{C\text{-}F}$ = 21.4 Hz), 95.1 (d, $J_{C\text{-}F}$ = 166.3 Hz), 46.5 (d, $J_{C\text{-}F}$ = 22.4 Hz), 45.6 (d, $J_{C\text{-}F}$ = 4.1 Hz), 27.8 (d, $J_{C\text{-}F}$ = 24.4 Hz), 27.0 (d, $J_{C\text{-}F}$ = 24.4 Hz); **^{19}F NMR (376 MHz, $CDCl_3$)** δ -115.2(s), -137.3 (s,). **FT-IR**: ν (cm^{-1}) 2972, 2853, 1654, 1541, 1373, 1222. **HRMS [ESI]** calcd for $C_{18}H_{17}F_2NSNa^+$ $[M+Na]^+$ 340.0942, found 340.0945.

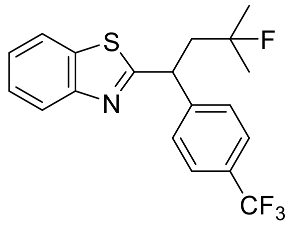

3g

3g: yellow oil. Purification by flash column chromatography on silica gel (eluent: EtOAc/Petroleum ether = 1/200). **^{1}H NMR (400 MHz, $CDCl_3$)** δ 8.02 (d, *J* = 8.4 Hz, 1H), 7.80 (d, *J* = 8.0 Hz, 1H), 7.62-7.53 (m, 4H), 7.46 (t, *J* = 7.6 Hz, 1H), 7.35 (t, *J* = 7.6 Hz, 1H), 4.76 (t, *J* = 6.4 Hz, 1H), 3.06 (ddd, *J* = 24.0, 14.4, 7.2 Hz, 1H), 2.5 (ddd, *J* = 21.6, 16.0, 6.0 Hz, 1H), 1.37 (d, *J* = 21.6 Hz, 3H), 1.31 (d, *J* = 21.6 Hz, 3H); **^{13}C NMR (100 MHz, $CDCl_3$)** δ 173.4, 153.0, 146.5, 135.2, 129.5 (q, $J_{C\text{-}F}$ = 32.4 Hz), 128.4, 126.1, 125.8 (q, $J_{C\text{-}F}$ = 3.8 Hz), 125.1, 124.0 (q, $J_{C\text{-}F}$ = 271.2 Hz), 123.0, 121.6, 95.0 (d, $J_{C\text{-}F}$ = 167.5 Hz), 46.4 (d, $J_{C\text{-}F}$ = 22.5 Hz), 46.0 (d, $J_{C\text{-}F}$ = 3.8 Hz), 27.8 (d, $J_{C\text{-}F}$ = 24.5 Hz), 26.8 (d, $J_{C\text{-}F}$ = 24.6 Hz); **^{19}F NMR (376 MHz, $CDCl_3$)** δ -62.5 (s), -137.7 (s). **FT-IR**: ν (cm^{-1}) 2981, 2929, 1618, 1322, 1164, 1106, 1067. **HRMS [ESI]** calcd for $C_{19}H_{17}F_4NS^+$ $[M+H]^+$ 367.1012, found 367.1020.

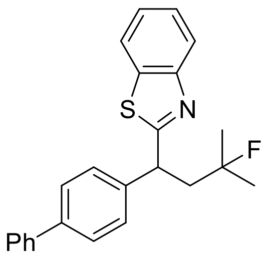

3h

3h: yellow oil. Purification by flash column chromatography on silica gel (eluent: EtOAc/Petroleum ether = 1/200).**^{1}H NMR (500 MHz, $CDCl_3$)** δ 8.03 (d, *J* = 8.0 Hz, 1H), 7.80 (d, *J* = 8.0 Hz, 1H), 7.60-7.55 (m, 4H), 7.53-7.49 (m, 2H), 7.47-7.40 (m, 3H), 7.34 (t, *J* = 7.5 Hz, 2H), 4.75 (t, *J* = 6.5 Hz, 1H), 3.25-2.98 (m, 1H), 2.67-2.47 (m, 1H), 1.39 (d, *J* = 21.5 Hz, 3H), 1.34 (d, *J* = 21.5 Hz, 3H); **^{13}C NMR (125 MHz, $CDCl_3$)** δ 174.9, 153.1, 141.6, 140.6, 140.2, 135.4, 128.8, 128.5, 127.6, 127.3, 127.1, 125.9, 124.9, 122.9, 121.6, 95.3 (d, $J_{C\text{-}F}$ = 167.1 Hz), 46.4 (d, $J_{C\text{-}F}$ = 22.8 Hz), 46.1 (d, $J_{C\text{-}F}$ = 4.2 Hz), 27.7 (d, $J_{C\text{-}F}$

= 24.5 Hz), 27.1 (d, J_{C-F} = 24.7 Hz); **^{19}F NMR (471 MHz, $CDCl_3$)** δ -136.9. **FT-IR**: ν (cm^{-1}) 3062, 2980, 2830, 1898, 1618, 1422, 1264, 1106. **HRMS [ESI]** calcd for $C_{24}H_{23}FNS^+$ $[M+H]^+$ 376.1530, found 376.1532.

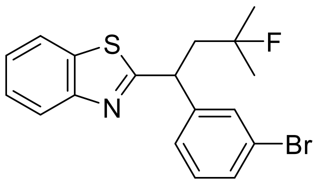

3i

3i: yellow oil. Purification by flash column chromatography on silica gel (eluent: EtOAc/Petroleum ether = 1/200). **^{1}H NMR (400 MHz, $CDCl_3$)** δ 8.03 (d, J = 8.0 Hz, 1H), 7.78 (d, J = 8.0 Hz, 1H), 7.62-7.52 (m, 2H), 7.45 (t, J = 8.0 Hz, 1H), 7.36-7.27 (m, 2H), 7.14-7.07 (m, 1H), 5.28 (t, J = 6.4 Hz, 1H), 3.16-3.02 (m, 1H), 2.58-2.45 (m, 1H), 1.39 (d, J = 21.2 Hz, 3H), 1.34 (d, J = 21.2 Hz, 3H); **^{13}C NMR (100 MHz, $CDCl_3$)** δ 173.3, 153.0, 141.4, 135.3, 133.1 129.8, 128.7, 127.9, 125.9, 124.9, 124.2, 122.9, 121.5, 95.0 (d, J_{C-F} = 166.4 Hz), 45.8 (d, J_{C-F} = 22.4 Hz), 44.5 (d, J_{C-F} = 4.1 Hz), 27.6 (d, J_{C-F} = 24.3 Hz), 27.0 (d, J_{C-F} = 24.5 Hz); **^{19}F NMR (376 MHz, $CDCl_3$)** δ -136.3 (s). **FT-IR**: ν (cm^{-1}) 3062, 2978, 2932, 1590, 1510, 1456, 1435, 1372, 1223, 1195. **HRMS [ESI]** calcd for $C_{18}H_{17}BrFNSNa^+$ $[M+Na]^+$ 402.0122, found 402.0123.

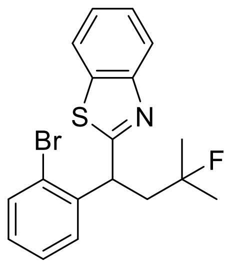

3j

3j: yellow oil. Purification by flash column chromatography on silica gel (eluent: EtOAc/Petroleum ether = 1/200). **^{1}H NMR (400 MHz, $CDCl_3$)** δ 8.03 (d, J = 8.0 Hz, 1H), 7.78 (d, J = 7.6 Hz, 1H), 7.62-7.52 (m, 2H), 7.45 (t, J = 7.6 Hz, 1H), 7.37-7.26 (m, 2H), 7.14-7.07 (m, 1H), 5.27 (t, J = 6.8 Hz, 1H), 3.16-3.02 (m, 1H), 2.59-2.45 (m, 1H), 1.39 (d, J = 21.2 Hz, 3H), 1.34 (d, J = 21.2 Hz, 3H); **^{13}C NMR (100 MHz, $CDCl_3$)** δ 173.3, 153.0, 141.4, 135.3, 133.1, 129.8, 128.7, 127.9, 125.9, 124.9, 124.2, 122.9, 121.5, 95.0 (d, J_{C-F} = 166.6 Hz), 45.8 (d, J_{C-F} = 22.4 Hz), 44.5 (d, J_{C-F} = 4.1 Hz), 27.6 (d, J_{C-F} = 24.4 Hz), 27.0 (d, J_{C-F} = 24.4 Hz); **^{19}F NMR (376 MHz, $CDCl_3$)** δ -137.5 (s). **FT-IR**: ν (cm^{-1}) 3061, 2978, 2924, 1591, 1510, 1435, 1372, 1241, 1195. **HRMS [ESI]** calcd for $C_{18}H_{17}BrFNS^+$ $[M+H]^+$ 377.0244, found 377.0252.

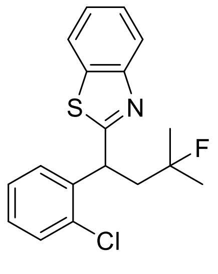

3k

3k: white solid, m.p. 62-63 °C. Purification by flash column chromatography on silica gel (eluent: EtOAc/Petroleum ether = 1/200). **^{1}H NMR (400 MHz, $CDCl_3$)** δ 8.02 (d, J = 8.0 Hz, 1H), 7.78 (d, J = 8.0 Hz, 1H), 7.53 (dd, J = 7.6, 1.6 Hz, 1H), 7.48-7.38 (m, 2H), 7.36-7.30 (m, 1H), 7.26-7.16 (m, 2H), 5.28 (t, J = 6.8 Hz, 1H), 3.16-3.02 (m, 1H), 2.57-2.46 (m, 1H), 1.39 (d, J = 21.2 Hz, 3H), 1.34 (d, J = 21.2 Hz, 3H); **^{13}C NMR (100 MHz, $CDCl_3$)** δ 173.3, 153.0, 139.8, 135.3, 133.3, 129.8, 129.6, 128.5, 127.3, 125.9, 124.9, 122.9, 121.5, 95.1 (d, J_{C-F} = 166.7 Hz), 45.7 (d, J_{C-F} = 22.5 Hz), 41.7 (d, J_{C-F} = 4.0 Hz), 27.6 (d, J_{C-F} = 24.3 Hz), 27.0 (d, J_{C-F} = 24.6 Hz); **^{19}F NMR** (376 MHz, $CDCl_3$) δ -136.6 (s). **FT-IR**: ν (cm^{-1}) 3060, 2982, 2925, 2852, 1571, 1473, 1182. **HRMS [ESI]** calcd for $C_{18}H_{17}ClFNS^+$ $[M+H]^+$ 333.0749, found 333.0761.

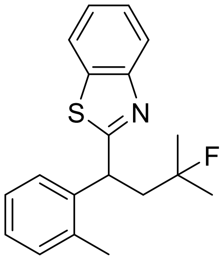

3l

3l: yellow oil. Purification by flash column chromatography on silica gel (eluent: EtOAc/Petroleum ether = 1/200). **^{1}H NMR (500 MHz, CDCl$_3$)** δ 8.02 (d, *J* = 8.0 Hz, 1H), 7.77 (d, *J* = 8.0 Hz, 1H), 7.50-7.36 (m, 2H), 7.35-7.30 (m, 1H), 7.21-7.12 (m, 3H), 4.97 (t, *J* = 6.5 Hz, 1H), 3.29-2.87 (m, 1H), 2.52 (s, 3H), 1.35 (d, *J* = 22.0 Hz, 3H), 1.31 (d, *J* = 22.0 Hz, 3H); **^{13}C NMR (125 MHz, CDCl$_3$)** δ 175.2, 153.0, 142.5, 138.5, 135.4, 128.7, 128.7, 128.0, 125.8, 125.0, 124.8, 122.9, 121.5, 95.3 (d, $J_{C\text{-}F}$ = 166.9 Hz), 46.3 (d, $J_{C\text{-}F}$ = 22.4 Hz), 46.2 (d, $J_{C\text{-}F}$ = 10.9 Hz), 27.6 (d, $J_{C\text{-}F}$ = 24.4 Hz), 27.1 (d, $J_{C\text{-}F}$ = 24.6 Hz), 21.4; **^{19}F NMR (376 MHz, CDCl$_3$)** δ -136.6 (s). **FT-IR**: ν (cm^{-1}) 2986, 2931, 1406, 1384, 1254, 1076. **HRMS [ESI]** calcd for $C_{19}H_{21}FNS^+$ $[M+H]^+$ 314.1373, found 314.1374.

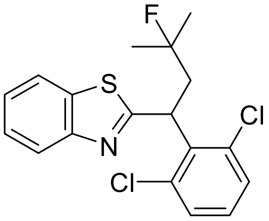

3m

3m: colorless oil. Purification by flash column chromatography on silica gel (eluent: EtOAc/Petroleum ether = 1/100). **^{1}H NMR (400 MHz, CDCl$_3$)** δ 8.00 (d, *J* = 8.0 Hz, 1H), 7.75 (d, *J* = 8.0 Hz, 1H), 7.47-7.40 (m, 2H), 7.36-7.27 (m, 2H), 7.24-7.18 (m, 1H), 5.54 (dd, *J* = 7.2, 4.0 Hz, 1H), 3.38 (ddd, *J* = 26.8, 14.8, 4.0 Hz, 1H), 2.84 (td, *J* = 15.2, 7.2 Hz, 1H), 1.51 (d, *J* = 21.2 Hz, 3H), 1.42 (d, *J* = 21.2 Hz, 3H); **^{13}C NMR (100 MHz, CDCl$_3$)** δ 173.2, 152.8, 138.5, 135.7, 130.1, 129.3, 128.6, 125.8, 124.8, 122.9, 121.4, 95.4 (d, $J_{C\text{-}F}$ = 166.5 Hz), 42.4 (d, $J_{C\text{-}F}$ = 3.6. Hz), 42.4 (d, *J* = 21.6 Hz), 27.7 (d, $J_{C\text{-}F}$ = 24.5 Hz), 26.6 (d, $J_{C\text{-}F}$ = 24.4 Hz); **^{19}F NMR (376 MHz, CDCl$_3$)** δ -139.0 (s). **FT-IR**: ν (cm^{-1}) 3063, 2979, 2929, 1684, 1579, 1560, 1456, 1386. **HRMS [ESI]** calcd for $C_{18}H_{16}Cl_2FNSNa^+$ $[M+Na]^+$ 390.0257, found 390.0256.

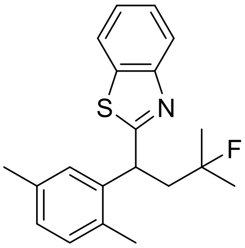

3n

3n: yellow oil. Purification by flash column chromatography on silica gel (eluent: EtOAc/Petroleum ether = 1/200). **^{1}H NMR (500 MHz, CDCl$_3$)** δ 8.03 (d, *J* = 8.0 Hz, 1H), 7.77 (d, *J* = 8.0 Hz, 1H), 7.45 (t, *J* = 7.5 Hz, 1H), 7.33 (t, *J* = 7.5 Hz, 1H), 7.19 (s, 1H), 7.10-7.05 (m, 1H), 6.99-6.94 (m, 1H), 4.92 (t, *J* = 6.5 Hz, 1H), 3.18-3.03 (m, 2H), 2.47 (s, 3H), 2.28 (s, 3H), 1.35 (d, *J* = 21.5 Hz, 3H), 1.30 (d, *J* = 21.5 Hz, 3H); **^{13}C NMR (125 MHz, CDCl$_3$)** δ 175.2, 153.0, 140.7, 135.9, 135.4, 132.3, 130.7, 128.2, 127.9, 125.8, 124.7, 122.8, 121.5, 95.3 (d, $J_{C\text{-}F}$ = 166.9 Hz), 46.0 (d, $J_{C\text{-}F}$ = 22.6 Hz), 41.5 (d, $J_{C\text{-}F}$ = 4.0 Hz), 27.7 (d, $J_{C\text{-}F}$ = 24.5 Hz), 27.1 (d, $J_{C\text{-}F}$ = 24.6 Hz), 21.1, 19.4; **^{19}F NMR (471 MHz, CDCl$_3$)** δ -136.9 (s). **FT-IR**: ν (cm^{-1}) 2977, 2911, 1506, 1432, 1384, 1175. **HRMS [ESI]** calcd for $C_{20}H_{23}FNS^+$ $[M+H]^+$ 328.1530, found 328.1529.

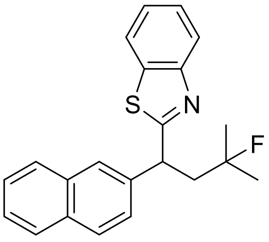

3o

3o: white solid, m.p. 89-90 ^{o}C. Purification by flash column chromatography on silica gel (eluent: EtOAc/Petroleum ether = 1/200). **^{1}H NMR (500 MHz, CDCl$_3$)** δ 8.05 (d, *J* = 8.0 Hz, 1H), 7.88 (d, *J* = 2.0 Hz, 1H), 7.85-7.73 (m, 4H), 7.57 (dd, *J* = 8.5, 2.0 Hz, 1H), 7.51-7.42 (m, 3H), 7.32 (t, *J* = 7.5 Hz, 1H), 4.88 (t, *J* = 6.5 Hz, 1H), 3.36-2.99 (m, 1H), 2.70-2.58 (m, 1H), 1.38 (d, *J* = 21.2 Hz, 3H) , 1.34 (d, *J* = 21.2 Hz, 3H) ; **^{13}C NMR (125 MHz, CDCl$_3$)** δ 174.9, 153.0, 139.9, 135.4, 133.5, 132.7, 128.7, 127.9, 127.6, 126.8, 126.3, 126.0, 126.0, 125.9, 124.8, 122.9, 121.5, 95.3 (d, $J_{C\text{-}F}$ = 167.2 Hz), 46.4 (d, $J_{C\text{-}F}$ = 4.3 Hz), 46.1 (d, $J_{C\text{-}F}$ = 22.6 Hz), 27.6 (d, $J_{C\text{-}F}$ = 24.5 Hz), 27.1 (d, $J_{C\text{-}F}$ = 24.5 Hz); **^{19}F NMR (471**

MHz, CDCl$_3$) δ -137.3 (s). **FT-IR**: ν (cm^{-1}) 3092, 2880, 2730, 1798, 1618, 1522, 1364, 1206. **HRMS [ESI]** calcd for $C_{22}H_{21}FNS^+$ [M+H]$^+$ 350.1373, found 350.1367.

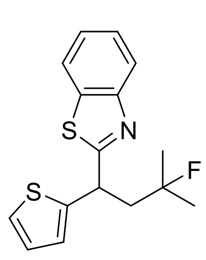

3p

3p: yellow oil. Purification by flash column chromatography on silica gel (eluent: EtOAc/Petroleum ether = 1/200). **^{1}H NMR (500 MHz, CDCl$_3$)** δ 8.01 (d, *J* = 8.0 Hz, 1H), 7.80 (d, *J* = 8.0 Hz, 1H), 7.45 (t, *J* = 7.5 Hz, 1H), 7.34 (t, *J* = 7.5 Hz, 1H), 7.22 (d, *J* = 5.0 Hz, 1H), 7.05 (d, *J* = 3.5 Hz, 1H), 6.95 (t, *J* = 4.5 Hz, 1H), 5.01 (t, *J* = 6.5 Hz, 1H), 3.08-2.93 (m, 1H), 2.62-2.50 (m, 1H), 1.37 (d, *J* = 21.5 Hz, 3H), 1.33 (d, *J* = 21.5 Hz, 3H); **^{13}C NMR (125 MHz, CDCl$_3$)** δ 174.6, 152.9, 145.6, 135.4, 126.9, 126.0, 125.4, 125.0, 124.9, 123.0, 121.6, 95.0 (d, J_{C-F} = 167.5 Hz), 47.6 (d, J_{C-F} = 22.9 Hz), 41.4 (d, J_{C-F} = 4.6 Hz), 27.2 (d, J_{C-F} = 23.9 Hz), 27.2 (d, J_{C-F} = 23.9 Hz); **^{19}F NMR (471 MHz, CDCl$_3$)** δ -139.1 (s). **FT-IR**: ν (cm^{-1}) 2978, 2989, 2714, 1681, 1669, 1512, 1213, 980. **HRMS [ESI]** calcd for $C_{16}H_{16}FNS_2Na^+$ [M+Na]$^+$ 328.0600, found 328.0607.

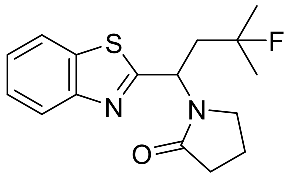

3q

3q: white solid, m.p. 90-91 ºC. Purification by flash column chromatography on silica gel (eluent: EtOAc/Petroleum ether = 1/10). **^{1}H NMR (400 MHz, CDCl$_3$)** δ 7.99 (d, *J* = 8.0 Hz, 1H), 7.85 (d, *J* = 8.0 Hz, 1H), 7.47 (t, *J* = 7.6 Hz, 1H), 7.38 (t, *J* = 8.0 Hz, 1H), 5.94 (dd, *J* = 10.4, 3.6 Hz, 1H), 3.55 (dd, *J* = 16.4, 8.0 Hz, 1H), 3.27 (td, *J* = 8.4, 5.2 Hz, 1H), 2.76-2.50 (m, 2H), 2.44 (t, *J* = 8.0 Hz, 2H), 2.10-1.94 (m, 2H), 1.48 (d, *J* = 21.6 Hz, 3H), 1.48 (d, *J* = 21.6 Hz, 3H); **^{13}C NMR (100 MHz, CDCl$_3$)** δ 175.1, 170.8, 152.9, 135.3, 126.1, 125.3, 123.1, 121.7, 94.7 (d, J_{C-F} = 165.9 Hz), 49.8, 43.0 (d, J_{C-F} = 3.2 Hz), 39.9 (d, J_{C-F} = 21.4 Hz), 31.1, 28.4 (d, J_{C-F} = 24.4 Hz), 25.5 (d, J_{C-F} = 24.8 Hz), 18.3; **^{19}F NMR (376 MHz, CDCl$_3$)** δ -141.0 (s). **FT-IR**: ν (cm^{-1}) 2956, 2850, 1645, 1557, 1373, 1313. **HRMS [ESI]** calcd for $C_{16}H_{19}FN_2OS^+$ [M+H]$^+$ 306.1197, found 306.1198.

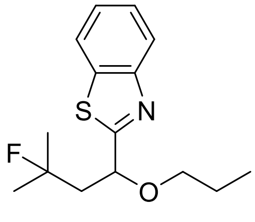

3r

3r: colorless oil. Purification by flash column chromatography on silica gel (eluent: EtOAc/Petroleum ether = 1/50). **^{1}H NMR (400 MHz, CDCl$_3$)** δ 8.00 (d, *J* = 8.4 Hz, 1H), 7.90 (d, *J* = 8.0 Hz, 1H), 7.47 (t, *J* = 7.6 Hz, 1H), 7.38 (t, *J* = 7.6 Hz, 1H), 4.99-4.89 (m, 1H), 3.63-3.45 (m, 2H), 2.32-2.16 (m, 2H), 1.64-1.57 (m, 2H), 1.50 (d, *J* = 21.2 Hz, 3H), 1.45 (d, *J* = 21.2 Hz, 3H), 0.91 (t, *J* = 7.2 Hz, 3H); **^{13}C NMR (100 MHz, CDCl$_3$)** δ 175.9, 153.1, 134.9, 126.0, 125.1, 123.0, 121.9, 94.4 (d, J_{C-F} = 165.9 Hz), 70.1, 48.1 (d, J_{C-F} = 23.1Hz), 31.9, 28.3 (d, J_{C-F} = 24.1 Hz), 26.6 (d, J_{C-F} = 24.7 Hz), 19.3, 13.9; **^{19}F NMR (376 MHz, CDCl$_3$)** δ -134.9 (s). **FT-IR**: ν (cm^{-1}) 2958, 2871, 1595, 1436, 1373, 1237, 1122. **HRMS [ESI]** calcd for $C_{15}H_{20}FNOSNa^+$ [M+Na]$^+$ 318.1298, found 318.1299.

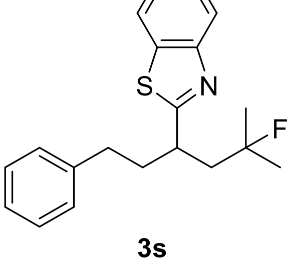

3s

3s: colorless oil. Purification by flash column chromatography on silica gel (eluent: EtOAc/Petroleum ether = 1/100). **^{1}H NMR (400 MHz, CDCl$_3$)** δ 8.02 (d, *J* = 8.0 Hz, 1H), 7.88 (d, *J* = 7.6 Hz, 1H), 7.51-7.44 (m, 1H), 7.41-7.34 (m, 1H), 7.28-7.24 (m, 2H), 7.21-7.13 (m, 3H), 3.55-3.45 (m, 1H), 2.70-2.54 (m, 2H), 2.47-2.32 (m, 1H), 2.23-2.11 (m, 3H), 1.34 (d, *J* = 20.8 Hz, 3H), 1.30 (d, *J* = 20.8 Hz, 3H); **^{13}C NMR (100 MHz, CDCl$_3$)** δ 176.4, 153.1, 141.5, 134.8, 128.4, 128.4, 126.0, 125.9, 124.8, 122.8, 121.7, 95.3 (d, J_{C-F} = 165.5 Hz), 46.9 (d, J_{C-F} = 22.3 Hz), 40.6 (d, J_{C-F} = 3.1 Hz), 39.5,

33.4, 27.9 (d, $J_{C\text{-}F}$ = 24.3 Hz), 26.4 (d, $J_{C\text{-}F}$ = 24.4 Hz); **FT-IR**: ν (cm^{-1}) 3061, 3026, 2928, 1603, 1515, 1454, 1104. **^{19}F NMR (376 MHz, $CDCl_3$)** δ -135.9 (s). **HRMS [ESI]** calcd for $C_{20}H_{23}FNS^+$ $[M+H]^+$ 328.1530, found 328.1531.

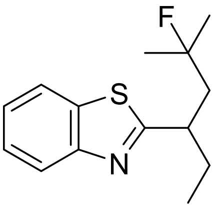

3t

3t: yellow oil. Purification by flash column chromatography on silica gel (eluent: EtOAc/Petroleum ether = 1/200). **^{1}H NMR (400 MHz, $CDCl_3$)** δ 7.98 (d, J = 8.0 Hz, 1H), 7.85 (d, J = 7.6 Hz, 1H), 7.49-7.41 (m, 1H), 7.38-7.31 (m, 1H), 3.39-3.29 (m, 1H), 2.44-2.29 (m, 1H), 2.18-2.05 (m, 1H), 1.93-1.78 (m, 2H), 1.34 (d, J = 21.2 Hz, 3H), 1.29 (d, J = 21.2 Hz, 3H), 0.91 (t, J = 7.6 Hz, 3H); **^{13}C NMR (100 MHz, $CDCl_3$)** δ 176.8, 153.0, 134.8 , 125.8, 124.7, 122.7, 121.6, 95.3 (d, $J_{C\text{-}F}$ = 167.1 Hz), 46.6 (d, $J_{C\text{-}F}$ = 22.6 Hz), 42.4 (d, $J_{C\text{-}F}$ = 3.5 Hz), 31.0, 27.8 (d, $J_{C\text{-}F}$ = 24.6 Hz), 26.4 (d, $J_{C\text{-}F}$ = 24.7 Hz), 11.8; **^{19}F NMR (376 MHz, $CDCl_3$)** δ -136.0 (s). **FT-IR**: ν (cm^{-1}) 2976, 2931, 2875, 1594, 1559, 1437, 1279. **HRMS [ESI]** calcd for $C_{14}H_{18}FNSNa^+$ $[M+Na]^+$ 274.1036, found 274.1035.

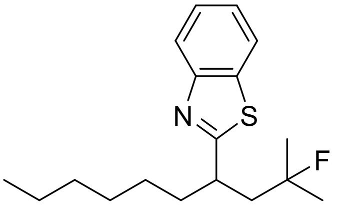

3u

3u: yellow oil. Purification by flash column chromatography on silica gel (eluent: EtOAc/Petroleum ether = 1/200). **^{1}H NMR (400 MHz, $CDCl_3$)** δ 8.01-7.95 (m, 1H), 7.88-7.81 (m, 1H), 7.48-7.41 (m, 1H), 7.38-7.31 (m, 1H), 3.35-3.46 (m, 1H), 2.43-2.28 (m, 1H), 2.17-2.04 (m, 1H), 1.85-1.76 (m, 2H), 1.37-1.20 (m, 14H), 0.84 (t, J = 6.8 Hz, 3H); **^{13}C NMR (100 MHz, $CDCl_3$)** δ 177.1, 153.0, 134.7, 125.8, 124.7, 122.7, 121.6, 95.3 (d, $J_{C\text{-}F}$ = 165.3 Hz), 47.0 (d, $J_{C\text{-}F}$ = 22.4 Hz), 40.9 (d, $J_{C\text{-}F}$ = 3.8 Hz), 38.0, 31.6, 29.1, 27.8 (d, $J_{C\text{-}F}$ = 24.5 Hz), 27.1, 26.5 (d, $J_{C\text{-}F}$ = 24.5 Hz), 22.5, 14.0; **^{19}F NMR (376 MHz, $CDCl_3$)** δ -135.8 (s). **FT-IR**: ν (cm^{-1}) 3064, 2955, 2927, 1595, 1517, 1438, 1373. **HRMS [ESI]** calcd for $C_{18}H_{26}FNS^+$ $[M+H]^+$ 308.1843, found 308.1840.

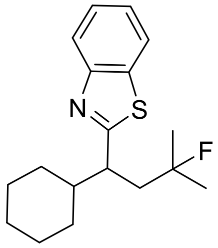

3v

3v: white solid, m.p. 49-50 °C. Purification by flash column chromatography on silica gel (eluent: EtOAc/Petroleum ether = 1/200). **^{1}H NMR (400 MHz, $CDCl_3$)** δ 7.99 (d, J = 8.0 Hz, 1H), 7.84 (d, J = 8.0 Hz, 1H), 7.48-7.41 (m, 1H), 7.38-7.31 (m, 1H), 3.27-3.19 (m, 1H), 2.52-2.35 (m, 1H), 2.24-2.11 (m, 1H), 1.96-1.86 (m, 1H), 1.80-1.58 (m, 4H), 1.31 (d, J = 21.6 Hz, 3H), 1.27-0.96 (m, 9H); **^{13}C NMR (100 MHz, $CDCl_3$)** δ 175.8, 152.9, 134.7, 125.7, 124.6, 122.6, 121.4, 95.5 (d, $J_{C\text{-}F}$ = 165.4 Hz), 46.1 (d, $J_{C\text{-}F}$ = 3.8 Hz), 44.3, 43.6 (d, $J_{C\text{-}F}$ = 22.5 Hz), 30.6, 30.4, 27.6 (d, $J_{C\text{-}F}$ = 24.3 Hz), 26.5 (d, $J_{C\text{-}F}$ = 24.5 Hz), 26.4, 26.3, 26.3; **^{19}F NMR (376 MHz, $CDCl_3$)** δ -136.2 (s). **FT-IR**: ν (cm^{-1}) 2958, 2841, 2713, 2326, 2246, 1577, 1541, 1311, 1130. **HRMS [ESI]** calcd for $C_{18}H_{25}FNS^+$ $[M+H]^+$ 306.1686, found 306.1680.

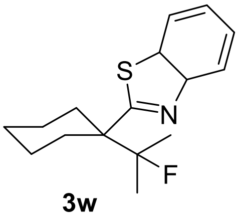

3w

3w: white solid, m.p 55-56 °C. Purification by flash column chromatography on silica gel (eluent: EtOAc/Petroleum ether = 1/100). **^{1}H NMR (400 MHz, $CDCl_3$)** δ 7.99 (d, J = 8.0 Hz, 1H), 7.86 (d, J = 7.6 Hz, 1H), 7.44 (t, J = 7.6 Hz, 1H), 7.34 (t, J = 7.6 Hz, 1H), 2.33 (d, J = 22.8 Hz, 2H), 2.27-2.17 (m, 2H), 1.98-1.87 (m, 2H), 1.76-1.64 (m, 2H), 1.64-1.53 (m, 2H), 1.51-1.42 (m, 2H), 1.18 (s, 3H), 1.12 (s, 3H); **^{13}C NMR (100 MHz, $CDCl_3$)** δ 180.6, 153.0, 135.0, 125.6, 124.6, 122.7, 121.5, 96.2 (d, $J_{C\text{-}F}$ = 165.3 Hz), 44.5, 38.3, 38.3, 28.2 (d,

J_{C-F} = 24.4 Hz), 25.8, 22.2; **^{19}F NMR (376 MHz, CDCl$_3$)** δ -135.8 (s). **FT-IR**: ν (cm^{-1}) 2976, 2853, 1624, 1541, 1434, 1375, 1243. **HRMS [ESI]** calcd for $C_{17}H_{22}FNS^+$ [M+H]$^+$ 291.1452, found 291.1459.

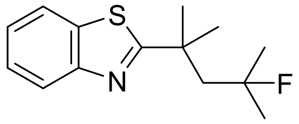

3x

3x: yellow solid, m.p. 48-49 °C. Purification by flash column chromatography on silica gel (eluent: EtOAc/Petroleum ether = 1/200). **^{1}H NMR (400 MHz, CDCl$_3$)** δ 7.98 (d, *J* = 8.0 Hz, 1H), 7.86 (d, *J* = 7.6 Hz, 1H), 7.45 (t, *J* = 7.2 Hz, 1H), 7.35 (t, *J* = 7.2 Hz, 1H), 2.39 (d, *J* = 24.4 Hz, 2H), 1.58 (s, 6H), 1.22 (d, *J* = 21.2 Hz,, 3H), 1.17 (d, *J* = 21.2 Hz,, 3H); **^{13}C NMR (100 MHz, CDCl$_3$)** δ 181.3, 153.2, 134.9, 125.8, 124.6, 122.6, 121.5, 96.0 (d, J_{C-F} = 165.2 Hz), 52.5 (d, *J* = 21.0 Hz), 40.8, 30.3, 30.3, 28.1 (d, J_{C-F} = 24.3 Hz); **^{19}F NMR (376 MHz, CDCl$_3$)** δ -133.1 (s). **FT-IR**: ν (cm^{-1}) 2987, 2921, 1635, 1593, 1372, 1248. **HRMS [ESI]** calcd for $C_{14}H_{18}FNSNa^+$ [M+Na]$^+$ 274.1036, found 274.1036.

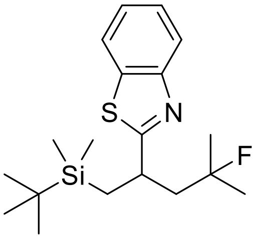

3y

3y: white solid, m.p. 55-56 °C. Purification by flash column chromatography on silica gel (eluent: EtOAc/Petroleum ether = 1/200) **^{1}H NMR (400 MHz, CDCl$_3$)** δ 7.96 (d, *J* = 8.0 Hz, 1H), 7.84 (d, *J* = 8.0 Hz, 1H), 7.44 (t, *J* = 7.6 Hz, 1H), 7.34 (t, *J* = 7.6 Hz, 1H), 3.61-3.51 (m, 1H), 2.45-2.30 (m, 1H), 2.19-2.06 (m, 1H), 1.32 (d, *J* = 21.6 Hz, 3H), 1.27 (d, *J* = 21.6 Hz, 3H), 1.21-1.14 (m, 2H), 0.85 (s, 9H), 0.02 (s, 3H), -0.27 (s, 3H); **^{13}C NMR (100 MHz, CDCl$_3$)** δ 178.7, 152.8, 134.6, 125.8, 124.7, 122.6, 121.6, 95.3 (d, J_{C-F} = 165.4 Hz), 50.3 (d, J_{C-F} = 24.4 Hz), 37.1 (d, J_{C-F} = 3.7 Hz), 27.8 (d, J_{C-F} = 24.4 Hz), 26.6 (d, J_{C-F} = 24.5 Hz), 26.4, 22.3, 16.5, -5.7, -6.2; **^{19}F NMR (376 MHz, CDCl$_3$)** δ -135.5 (s). **FT-IR**: ν (cm^{-1}) 3059, 2928, 2854, 1541, 1457, 1371. **HRMS [ESI]** calcd for $C_{19}H_{31}FNSSi^+$ [M+H]$^+$ 352.1925, found 352.1926.

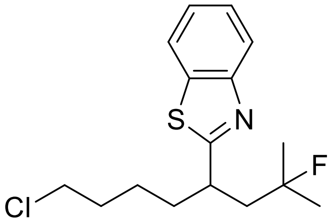

3z

3z: yellow oil. Purification by flash column chromatography on silica gel (eluent: EtOAc/Petroleum ether = 1/100). **^{1}H NMR (400 MHz, CDCl$_3$)** δ 8.02-7.95 (m, 1H), 7.88-7.82 (m, 1H), 7.49-7.42 (m, 1H), 7.39-7.32 (m, 1H), 3.50-3.38 (m, 3H), 2.44-2.29 (m, 1H), 2.17-2.05 (m, 1H), 1.91-1.67 (m, 5H), 1.53-1.44 (m, 1H), 1.35 (d, *J* = 21.2 Hz, 3H), 1.29 (d, *J* = 21.2 Hz, 3H); **^{13}C NMR (100 MHz, CDCl$_3$)** δ 176.4, 152.9, 134.7, 125.9, 124.8, 122.7, 121.6, 95.2 (d, J_{C-F} = 165.6 Hz), 46.8 (d, J_{C-F} = 22.2 Hz), 44.6, 40.7 (d, *J* = 3.3 Hz), 37.0, 32.3, 27.8 (d, J_{C-F} = 24.4 Hz), 26.4 (d, J_{C-F} = 24.5 Hz), 24.5; **^{19}F NMR (376 MHz, CDCl$_3$)** δ -136.4 (s). **FT-IR**: ν (cm^{-1}) 2978, 2866, 1724, 1516, 1437, 1386, 1106. **HRMS [ESI]** calcd for $C_{16}H_{22}ClFNS^+$ [M+H]$^+$ 314.1140, found 314.1144.

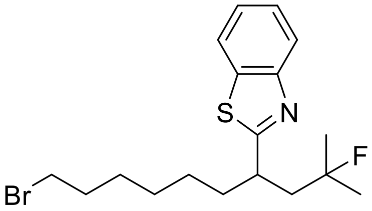

3aa

3aa: yellow solid, m.p. 103-104 °C. Purification by flash column chromatography on silica gel (eluent: EtOAc/Petroleum ether = 1/100). **^{1}H NMR (400 MHz, CDCl$_3$)** δ 7.98 (d, *J* = 8.4 Hz, 1H), 7.85 (d, *J* = 8.0 Hz, 1H), 7.48-7.41 (m, 1H), 7.39-7.31 (m, 1H), 3.46-3.38 (m, 1H), 3.35 (t, *J* = 6.8 Hz, 2H), 2.43-2.28 (m, 1H), 2.17-2.04 (m, 1H), 1.87-1.75 (m, 4H), 1.42-1.24 (m, 12H); **^{13}C NMR (100 MHz, CDCl$_3$)** δ 176.8, 153.0, 134.7, 125.8, 124.7, 122.7, 121.6, 95.3 (d, J_{C-F} = 165.4 Hz), 47.1 (d, J_{C-F} = 22.2 Hz), 40.8 (d, J_{C-F} = 3.4 Hz), 37.8, 33.8, 32.6,

28.5, 27.9, 27.8 (d, $J_{C\text{-}F}$ = 24.4 Hz), 26.9, 26.4 (d, $J_{C\text{-}F}$ = 24.6 Hz); **^{19}F NMR (376 MHz, CDCl$_3$)** δ -136.1 (s). **FT-IR**: ν (cm^{-1}) 3063, 2978, 2931, 2855, 1594, 1516, 1437, 1241. **HRMS [ESI]** calcd for $C_{18}H_{26}BrFNS^+$ [M+H]$^+$ 386.0948, found 386.0949.

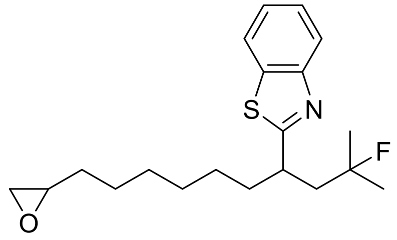

3ab

3ab: yellow oil. Purification by flash column chromatography on silica gel (eluent: EtOAc/Petroleum ether = 1/10). **^{1}H NMR (400 MHz, CDCl$_3$)** δ 7.97 (d, J = 8.0 Hz, 1H), 7.85 (d, J = 8.0 Hz, 1H), 7.45 (t, J = 7.6 Hz, 1H), 7.35 (t, J = 7.6 Hz, 1H), 3.46-3.36 (m, 1H), 2.89-2.82 (m, 1H), 2.74-2.68 (m, 1H), 2.46-2.27 (m, 2H), 2.16-2.04 (m, 1H), 1.87-1.75 (m, 2H), 1.51-1.44 (m, 2H), 1.39-1.22 (m, 14H); **^{13}C NMR (100 MHz, CDCl$_3$)** δ 177.0, 153.0, 134.7, 125.8, 124.7, 122.7, 121.6, 95.3 (d, $J_{C\text{-}F}$ = 165.4 Hz), 52.3, 47.1, 47.0 (d, $J_{C\text{-}F}$ = 21.8 Hz), 40.9 (d, $J_{C\text{-}F}$ = 3.5 Hz), 37.9, 32.4, 29.3, 29.2, 27.8 (d, $J_{C\text{-}F}$ = 24.5 Hz), 27.0, 26.4 (d, $J_{C\text{-}F}$ = 24.5 Hz), 25.8; **^{19}F NMR (376 MHz, CDCl$_3$)** δ -136.0 (s). **FT-IR**: ν (cm^{-1}) 2978, 2927, 2855, 1594, 1456, 1372, 1242, 1100. **HRMS [ESI]** calcd for $C_{20}H_{29}FNOS^+$ [M+H]$^+$ 350.1948, found 350.1946.

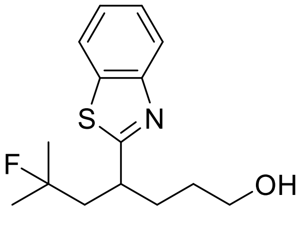

3ac

3ac: yellow oil. Purification by flash column chromatography on silica gel (eluent: EtOAc/Petroleum ether = 1/10). **^{1}H NMR (400 MHz, CDCl$_3$)** δ 7.98-7.92 (m, 1H), 7.87-7.80 (m, 1H), 7.47-7.40 (m, 1H), 7.38-7.30 (m, 1H), 3.64-3.57 (m, 2H), 3.51-3.41 (m, 1H), 2.42-2.27 (m, 1H), 2.19-2.04 (m, 2H), 1.97-1.81 (m, 2H), 1.65-1.54 (m, 1H), 1.54-1.45 (m, 1H), 1.34 (d, J = 21.6 Hz, 3H), 1.28 (d, J = 21.6 Hz, 3H); **^{13}C NMR (100 MHz, CDCl$_3$)** δ 176.7, 152.8, 134.6, 125.9, 124.8, 122.6, 121.6, 95.2 (d, $J_{C\text{-}F}$ = 165.7 Hz), 62.3, 47.0 (d, $J_{C\text{-}F}$ = 22.3 Hz), 40.5 (d, $J_{C\text{-}F}$ = 3.5 Hz), 34.0, 30.1, 27.7 (d, $J_{C\text{-}F}$ = 24.4 Hz), 26.4 (d, $J_{C\text{-}F}$ = 24.5 Hz); **^{19}F NMR (376 MHz, CDCl$_3$)** δ -136.3 (s). **FT-IR**: ν (cm^{-1}) 3351, 3063, 2978, 2932, 1594, 1455, 1386, 1117. **HRMS [ESI]** calcd for $C_{15}H_{20}FNOS^+$ [M+H]$^+$ 281.1244, found 281.1252.

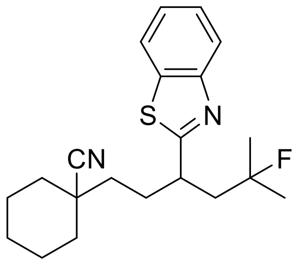

3ad

3ad: yellow oil. Purification by flash column chromatography on silica gel (eluent: EtOAc/Petroleum ether = 1/10). **^{1}H NMR (400 MHz, CDCl$_3$)** δ 7.97 (d, J = 8.0 Hz, 1H), 7.85 (d, J = 8.0 Hz, 1H), 7.49-7.43 (m, 1H), 7.40-7.33 (m, 1H), 3.49-3.39 (m, 1H), 2.46-2.30 (m, 1H), 2.19-1.97 (m, 3H), 1.91 (d, J = 13.6 Hz, 2H), 1.74-1.51 (m, 6H), 1.43-1.32 (m, 4H), 1.27 (d, J = 21.6 Hz, 3H), 1.20-1.03 (m, 3H); **^{13}C NMR (100 MHz, CDCl$_3$)** δ 175.8, 152.8, 134.7, 125.9, 124.9, 123.3, 122.7, 121.7, 95.0 (d, $J_{C\text{-}F}$ = 165.9 Hz), 46.8 (d, $J_{C\text{-}F}$ = 22.3 Hz), 40.8 (d, $J_{C\text{-}F}$ = 3.0 Hz), 38.7, 37.7, 35.8, 35.2, 32.5, 27.7 (d, $J_{C\text{-}F}$ = 24.3 Hz), 26.4 (d, $J_{C\text{-}F}$ = 24.5 Hz), 25.3, 22.9, 22.9; **^{19}F NMR (376 MHz, CDCl$_3$)** δ -136.7 (s). **FT-IR**: ν (cm^{-1}) 2977, 2927, 2366, 1730, 1455, 1371, 1186. **HRMS [ESI]** calcd for $C_{21}H_{27}FN2S^+$ [M+H]$^+$ 358.1873, found 358.1884.

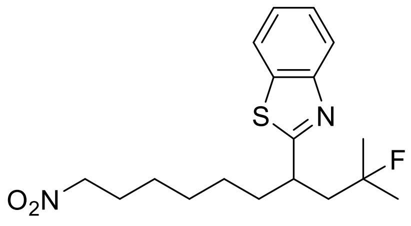

3ae

3ae: yellow oil. Purification by flash column chromatography on silica gel (eluent: EtOAc/Petroleum ether = 1/20). **^{1}H NMR (400 MHz, CDCl$_3$)** δ 7.97 (d, J = 8.0 Hz, 1H), 7.85 (d, J = 8.0 Hz, 1H), 7.49-7.20 (m, 1H), 7.39-7.32 (m, 1H), 4.32 (t, J = 7.2 Hz, 2H), 3.46-3.36 (m, 1H), 2.43-2.28 (m, 1H), 2.15-2.04 (m, 1H), 1.99-1.90 (m, 2H), 1.82 (q, J = 7.2 Hz, 2H), 1.38-1.25 (m, 12H); **^{13}C NMR (100 MHz, CDCl$_3$)** δ 176.6, 152.9, 134.6, 125.9, 124.7, 122.7, 121.6, 95.2 (d, $J_{C\text{-}F}$ = 165.6 Hz), 75.5, 47.0 (d, $J_{C\text{-}F}$ = 22.3 Hz), 40.7 (d, $J_{C\text{-}F}$ = 3.4 Hz), 37.6, 28.5, 27.8 (d, $J_{C\text{-}F}$ = 24.3 Hz), 27.2, 26.7, 26.4 (d, $J_{C\text{-}F}$ = 24.5 Hz), 25.9; **^{19}F NMR (376 MHz, CDCl$_3$)** δ -136.3 (s). **FT-IR**: ν (cm^{-1}) 2979, 2929, 2858, 1548, 1516, 1436, 1384, 1279. **HRMS [ESI]** calcd for $C_{18}H_{26}FN_2O_2S^+$ $[M+H]^+$ 353.1694, found 353.1697.

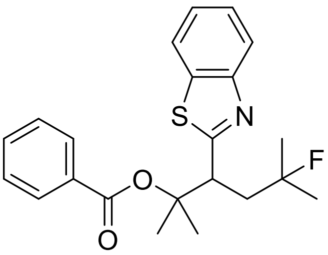

3af

3af: white solid, m.p. 130-131 ºC. Purification by flash column chromatography on silica gel (eluent: EtOAc/Petroleum ether = 1/10). **^{1}H NMR (400 MHz, CDCl$_3$)** δ 8.08-7.98 (m, 3H), 7.84 (d, J = 7.6 Hz, 1H), 7.60-7.53 (m, 1H), 7.50-7.41 (m, 3H), 7.39-7.33 (m, 1H), 4.11 (d, J = 10.0 Hz, 1H), 2.77-2.62 (m, 1H), 2.46-2.32 (m, 1H), 1.74 (s, 3H), 1.73 (s, 3H), 1.33 (d, J = 21.6 Hz, 3H), 1.25 (d, J = 21.6 Hz, 3H); **^{13}C NMR (100 MHz, CDCl$_3$)** δ 171.8, 165.5, 152.7, 135.1, 132.7, 131.5, 129.5, 128.3, 125.9, 125.0, 123.0, 121.4, 95.1 (d, $J_{C\text{-}F}$ = 166.2 Hz), 84.3, 49.5 (d, $J_{C\text{-}F}$ = 4.2 Hz), 42.1 (d, $J_{C\text{-}F}$ = 23.0 Hz), 27.5 (d, $J_{C\text{-}F}$ = 24.4 Hz), 26.5 (d, $J_{C\text{-}F}$ = 24.6 Hz), 24.7, 24.0; **^{19}F NMR (376 MHz, CDCl$_3$)** δ -136.4 (s). **FT-IR**: ν (cm^{-1}) 3375, 2987, 2901,1772, 1456, 1405, 1259. **HRMS [ESI]** calcd for $C_{22}H_{24}FNO_2SNa^+$ $[M+Na]^+$ 408.1404, found 408.1405.

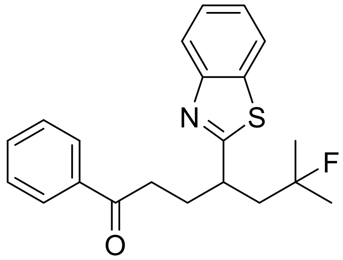

3ag

3ag: white solid, m.p. 51-52 ºC. Purification by flash column chromatography on silica gel (eluent: EtOAc/Petroleum ether = 1/10). **^{1}H NMR (400 MHz, CDCl$_3$)** δ 7.97 (d, J = 8.4 Hz, 1H), 7.89-7.81 (m, 3H), 7.56-7.32 (m, 5H), 3.63-3.53 (m, 1H), 3.08-2.87 (m, 2H), 2.51-2.32 (m, 2H), 2.29-2.12 (m, 2H), 1.38 (d, J = 21.2 Hz, 3H), 1.34 (d, J = 21.2 Hz, 3H); **^{13}C NMR (100 MHz, CDCl$_3$)** δ 199.3, 175.9, 153.0, 136.7, 134.8, 133.0, 128.5, 128.0, 126.0, 124.9, 122.8, 121.7, 95.2 (d, $J_{C\text{-}F}$ = 165.7 Hz), 47.1 (d, $J_{C\text{-}F}$ = 22.2 Hz), 40.1 (d, $J_{C\text{-}F}$ = 3.2 Hz), 35.8, 31.7, 27.7 (d, $J_{C\text{-}F}$ = 24.4 Hz), 26.5 (d, $J_{C\text{-}F}$ = 24.5 Hz); **^{19}F NMR (376 MHz, CDCl$_3$)** δ -136.3 (s). **FT-IR**: ν (cm^{-1}) 3060, 2962, 2833, 1688, 1596, 1446, 1370, 1261, 1154. **HRMS [ESI]** calcd for $C_{21}H_{22}FNOSNa^+$ $[M+Na]^+$ 378.1298, found 378.1298.

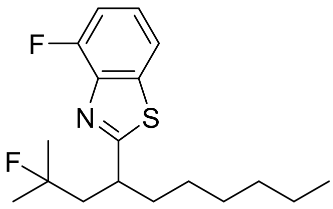

3ah

3ah: colorless oil. Purification by flash column chromatography on silica gel (eluent: EtOAc/Petroleum ether = 1/200). **^{1}H NMR (400 MHz, $CDCl_3$)** δ 7.66-7.55 (m, 1H), 7.29 (td, *J* = 8.0, 4.8 Hz, 1H), 7.20-7.09 (m, 1H), 3.61-3.39 (m, 1H), 2.36 (ddd, *J* = 23.2, 14.4, 8.4 Hz, 1H), 2.10 (ddd, *J* = 18.4, 14.4, 4.0 Hz, 1H), 1.86-1.74 (m, 2H), 1.40-1.15 (m, 14H), 0.84 (t, *J* = 6.8 Hz, 3H); **^{13}C NMR (100 MHz, $CDCl_3$)** δ 177.9, 155.4 (d, $J_{C\text{-}F}$ = 255.6 Hz), 141.7 (d, $J_{C\text{-}F}$ = 13.4 Hz), 137.5 (d, $J_{C\text{-}F}$ = 3.3 Hz), 125.4 (d, $J_{C\text{-}F}$ = 7.1 Hz), 117.3 (d, $J_{C\text{-}F}$ = 4.2 Hz), 111.6 (d, $J_{C\text{-}F}$ = 18.2 Hz), 95.3 (d, $J_{C\text{-}F}$ = 166.5 Hz), 47.0 (d, $J_{C\text{-}F}$ = 22.4 Hz), 41.1 (d, $J_{C\text{-}F}$ = 3.3 Hz), 38.0, 31.6, 29.1, 27.6 (d, $J_{C\text{-}F}$ = 24.6 Hz), 27.1, 26.6 (d, $J_{C\text{-}F}$ = 24.6 Hz), 22.5, 14.0; **^{19}F NMR (376 MHz, $CDCl_3$)** δ -122.1 (s), -136.0 (s). **FT-IR**: ν (cm^{-1}) 2955, 2927, 2856, 1612, 1569, 1513, 1316, 1244. **HRMS [ESI]** calcd for $C_{18}H_{25}F_2NSNa^+$ $[M+Na]^+$ 348.1568, found 348.1570.

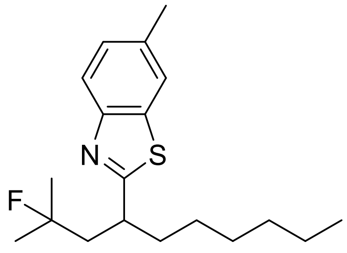

3ai

3ai: colorless oil. Purification by flash column chromatography on silica gel (eluent: EtOAc/Petroleum ether = 1/200). **^{1}H NMR (400 MHz, $CDCl_3$)** δ 7.84 (d, *J* = 8.0 Hz, 1H), 7.63 (s, 1H), 7.31-7.21 (m, 1H), 3.42-3.31 (m, 1H), 2.47 (s, 3H), 2.33 (ddd, *J* = 24.0, 14.8, 8.8 Hz, 1H), 2.16-2.03 (m, 1H), 1.85-1.71 (m, 2H), 1.38-1.14 (m, 14H), 0.84 (t, *J* = 6.8 Hz, 3H); **^{13}C NMR (100 MHz, $CDCl_3$)** δ 175.9, 151.0, 134.9, 134.7, 127.3, 122.1, 121.4, 95.4 (d, $J_{C\text{-}F}$ = 166.2 Hz), 47.0 (d, $J_{C\text{-}F}$ = 22.6 Hz), 40.9 (d, $J_{C\text{-}F}$ = 3.7 Hz), 38.0, 31.6, 29.1, 27.8 (d, $J_{C\text{-}F}$ = 24.5 Hz), 27.1, 26.4 (d, $J_{C\text{-}F}$ = 24.7 Hz), 22.6, 21.4, 14.0; **^{19}F NMR (376 MHz, $CDCl_3$)** δ -135.5 (s). **FT-IR**: ν (cm^{-1}) 2977, 2954, 2925, 1696, 1608, 1519, 1372, 1245. **HRMS [ESI]** calcd for $C_{19}H_{29}FNS^+$ $[M+H]^+$ 322.1999, found 322.1997.

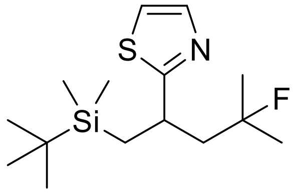

3aj

3aj: colorless oil. Purification by flash column chromatography on silica gel (eluent: EtOAc/Petroleum ether = 1/100). **^{1}H NMR (400 MHz, $CDCl_3$)** δ 7.63 (d, *J* = 3.2 Hz, 1H), 7.18 (d, *J* = 3.2 Hz, 1H), 3.55-3.46 (m, 1H), 2.33-2.19 (m, 1H), 2.12-2.01 (m, 1H), 1.25 (d, *J* = 21.6 Hz, 3H), 1.23 (d, *J* = 21.6 Hz, 3H), 1.10 (d, *J* = 7.2 Hz, 2H), 0.83 (s, 9H), -0.03 (s, 3H), -0.37 (s, 3H); **^{13}C NMR (100 MHz, $CDCl_3$)** δ 177.6, 141.7, 117.7, 95.3 (d, $J_{C\text{-}F}$ = 165.0 Hz), 51.2 (d, $J_{C\text{-}F}$ = 21.9 Hz), 36.1 (d, $J_{C\text{-}F}$ = 3.9 Hz), 27.7 (d, $J_{C\text{-}F}$ = 24.4 Hz), 26.4 (d, $J_{C\text{-}F}$ = 24.5 Hz), 26.4, 22.6, 16.5, -6.1, -6.4; **^{19}F NMR (376 MHz, $CDCl_3$)** δ -135.4 (s). **FT-IR**: ν (cm^{-1}) 2979, 2928, 2856, 1501, 1441, 1362. **HRMS [ESI]** calcd for $C_{15}H_{29}FNSSi^+$ $[M+H]^+$ 302.1769, found 302.1771.

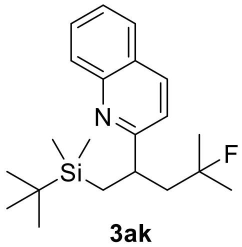

3ak

3ak: colorless oil. Purification by flash column chromatography on silica gel (eluent: EtOAc/Petroleum ether = 1/100). **^{1}H NMR (400 MHz, $CDCl_3$)** δ 8.08-8.02 (m, 2H), 7.79-7.74 (m, 1H), 7.70-7.63 (m, 1H), 7.50-7.44 (m, 1H), 7.32 (d, *J* = 8.8 Hz, 1H), 3.41-3.31 (m, 1H), 2.53-2.38 (m, 1H), 2.07 (ddd, *J* = 18.4, 14.4, 4.0 Hz, 1H), 1.22 (d, *J* = 22.0 Hz, 3H), 1.27 (d, *J* = 21.6 Hz, 3H), 1.16-1.03 (m, 2H), 0.83 (s, 9H), -0.03 (s, 3H), -0.44 (s, 3H); **^{13}C NMR (100 MHz, $CDCl_3$)** δ 167.2, 147.8, 136.1, 129.1, 129.1, 127.5, 126.9, 125.6, 120.8, 95.8 (d, $J_{C\text{-}F}$ = 164.4 Hz), 49.6 (d, $J_{C\text{-}F}$ = 21.7 Hz), 44.5 (d, $J_{C\text{-}F}$ = 4.0 Hz), 27.8 (d, $J_{C\text{-}F}$ = 24.5 Hz), 26.8 (d, $J_{C\text{-}F}$ = 24.5 Hz), 26.4, 21.3 16.5, -5.6, -6.0; **^{19}F NMR (376 MHz, $CDCl_3$)** δ -133.8 (s). **FT-IR**: ν (cm^{-1}) 2977,

2927, 2855, 1619, 1503, 1427, 1385, 1248. **HRMS [ESI]** calcd for $C_{21}H_{32}FNSi^+$ [M+H]$^+$ 345.2283, found 345.2286.

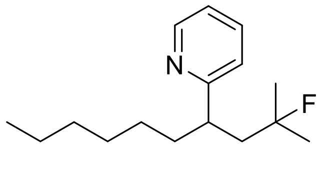

3al

3al: colorless oil. Purification by flash column chromatography on silica gel (eluent: EtOAc/Petroleum ether = 1/100). **^{1}H NMR (400 MHz, CDCl$_3$)** δ 8.53 (d, *J* = 4.0 Hz, 1H), 7.56 (td, *J* = 7.6, 1.6 Hz, 1H), 7.12 (d, *J* = 8.0 Hz, 1H), 7.07 (dd, *J* = 7.2, 5.2 Hz, 1H), 3.00-2.90 (m, 1H), 2.37-2.22 (m, 1H), 1.99-1.88 (m, 1H), 1.74-1.57 (m, 2H), 1.31-1.10 (m, 14H), 0.82 (t, *J* = 6.4 Hz, 3H); **^{13}C NMR (100 MHz, CDCl$_3$)** δ 165.4, 149.2, 136.0, 123.1, 121.0, 95.8 (d, J_{C-F} = 164.3 Hz), 46.4 (d, J_{C-F} = 22.0 Hz), 43.5 (d, J_{C-F} = 3.8 Hz), 37.6, 31.7, 29.2, 27.8 (d, J_{C-F} = 24.5 Hz), 27.3, 26.7 (d, J_{C-F} = 24.5 Hz), 22.5, 14.0; **^{19}F NMR (376 MHz, CDCl$_3$)** δ -135.1 (s). **FT-IR**: ν (cm^{-1}) 2978, 2926, 1590, 1471, 1434, 1384, 1229. **HRMS [ESI]** calcd for $C_{16}H_{27}FN^+$ [M+H]$^+$ 252.2122, found 252.2120.

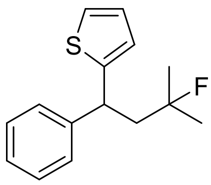

3am

3am: colorless oil. Purification by flash column chromatography on silica gel (eluent: EtOAc/Petroleum ether = 1/100). **^{1}H NMR (500 MHz, CDCl$_3$)** δ 7.33-7.28 (m, 4H), 7.23-7.19 (m, 1H), 7.13 (dd, *J* = 5.0, 1.2 Hz, 1H), 6.93-6.88 (m, 1H), 6.85-6.82 (m, 1H), 4.45 (t, *J* = 7.0 Hz, 1H), 2.56-2.47 (m, 2H), 1.29 (d, *J* = 3.5 Hz, 3H), 1.24 (d, *J* = 3.5 Hz, 3H); **^{13}C NMR (126 MHz, CDCl$_3$)** δ 149.9, 145.0, 128.6, 127.6, 126.6, 126.6, 123.7, 123.6, 95.4 (d, J_{C-F} = 166.4 Hz), 48.5 (d, J_{C-F} = 23.1 Hz), 42.5 (d, J_{C-F} = 5.5 Hz), 27.4 (d, J_{C-F} = 23.9 Hz), 27.2 (d, J_{C-F} = 23.9 Hz); **^{19}F NMR (376 MHz, CDCl$_3$)** δ -135.6 (s). **FT-IR**: ν (cm^{-1}) 2988, 2826, 1690, 1571, 1434, 1284, 1129. **HRMS [ESI]** calcd for $C_{15}H_{18}FS^+$ [M+H]$^+$ 249.1108, found 249.1112.

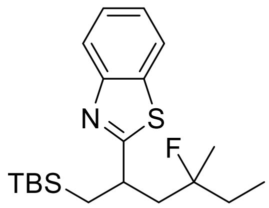

3an

3an: colorless oil. Purification by flash column chromatography on silica gel (eluent: EtOAc/Petroleum ether = 1/100). **^{1}H NMR (400 MHz, CDCl$_3$)** δ 7.96 (d, *J* = 8.0 Hz, 1H, two isomers), 7.83 (d, *J* = 8.0 Hz, 1H, two isomers), 7.47-7.40 (m, 1H, two isomers), 7.38-7.29 (m, 1H, two isomers), 3.63-3.51 (m, 1H, two isomers), 2.46-2.24 (m, 1H, two isomers), 2.18-2.00 (m, 1H, two isomers), 1.76-1.46 (m, 2H, two isomers), 1.35-1.11 (m, 5H, two isomers), 0.94-0.87 (m, 3H, two isomers), 0.85 (s, 9H, two isomers), 0.02 (s, 1.5H, one isomer), 0.01 (s, 1.5H, one isomer), -0.26 (s, 1.5H, one isomer), -0.29 (s, 1.5H, one isomer); **^{13}C NMR (100 MHz, CDCl$_3$)** δ 178.9 & 178.6 (two isomers), 152.9 & 152.8 (two isomers), 134.7 (overlap, two isomers), 125.8 & 125.8 (two isomers), 124.6 & 124.96 (two isomers), 122.7 (overlap, two isomers), 121.6 (overlap, two isomers), 97.3 (d, J_{C-F} = 168.1 Hz, overlap, two isomers), 48.9 (d, J_{C-F} = 21.9 Hz, one isomer) & 48.1 (d, J_{C-F} = 21.9 Hz, one isomer), 36.9 (d, J_{C-F} = 3.7 Hz, one isomer) & 36.8 (d, J_{C-F} = 3.0 Hz, one isomer), 33.6 (d, J_{C-F} = 23.2 Hz, one isomer) & 33.2 (d, J_{C-F} = 23.4 Hz, one isomer), 26.4 (overlap, two isomers), 24.1 (d, J_{C-F} = 24.7 Hz, one isomer) & 23.4 (d, J_{C-F} = 24.8 Hz, one isomer), 22.4 & 22.3 (two isomers), 16.5 (overlap, two isomers), 7.9 & 7.9 (two isomers), -5.8 (overlap, two isomers), -6.1 (overlap, two isomers); **^{19}F NMR (376 MHz, CDCl$_3$)** δ -143.9 (s, one isomer), -145.2 (s, one isomer). **FT-IR**: ν (cm^{-1}) 2951, 2927, 2855, 1533, 1462, 1437, 1379, 1361, 1248, 1096. **HRMS [ESI]** calcd for $C_{20}H_{32}FNSSiNa^+$ [M+Na]$^+$ 388.1907, found 388.1908.

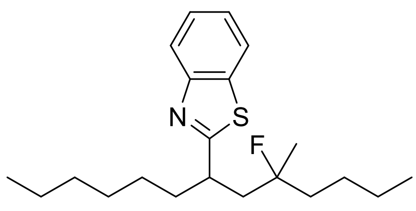

3ao

3ao: yellow oil. Purification by flash column chromatography on silica gel (eluent: EtOAc/Petroleum ether = 1/200). **^{1}H NMR (400 MHz, $CDCl_3$)** δ 7.98 (d, *J* = 8.0 Hz, 1H, two isomers), 7.84 (d, *J* = 8.0 Hz, 1H, two isomers), 7.44 (t, *J* = 7.6 Hz, 1H, two isomers), 7.34 (t, *J* = 7.6 Hz, 1H, two isomers), 3.48-3.33 (m, 1H, two isomers), 2.45-2.24 (m, 1H, two isomers), 2.16-1.98 (m, 1H, two isomers), 1.88-1.73 (m, 2H, two isomers), 1.67-1.51 (m, 2H, two isomers), 1.38-1.10 (m, 15H, two isomers), 0.94-0.75 (m, 6H, two isomers); **^{13}C NMR (100 MHz, $CDCl_3$)** δ 177.2 & 177.1 (two isomers), 153.0 & 152.9 (two isomers), 134.7 (overlap, two isomers), 125.8 & 125.8 (two isomers), 124.6 & 124.6 (two isomers), 122.7 & 122.6 (two isomers), 121.6 (overlap, two isomers), 97.2 (d, $J_{C\text{-}F}$ = 167.4 Hz, one isomer) & 97.2 (d, $J_{C\text{-}F}$ = 167.4 Hz, one isomer), 40.7 & 40.5 (two isomers), 45.6 (d, $J_{C\text{-}F}$ = 22.3 Hz, one isomer) & 45.2 (d, $J_{C\text{-}F}$ = 22.3 Hz, one isomer), 40.7 (d, $J_{C\text{-}F}$ = 4.0 Hz, one isomer) & 40.6 (d, $J_{C\text{-}F}$ = 4.1 Hz, one isomer), 39.3 & 39.1 (two isomers), 38.1 & 38.0 (two isomers), 31.6 (overlap, two isomers), 29.1 (overlap, two isomers), 27.2 & 27.1 (two isomers), 25.8 (d, $J_{C\text{-}F}$ = 5.9 Hz, one isomer) & 25.8 (d, $J_{C\text{-}F}$ = 5.6 Hz, one isomer), 24.9 (d, $J_{C\text{-}F}$ = 24.6 Hz, one isomer) & 24.1 (d, $J_{C\text{-}F}$ = 24.7 Hz, one isomer), 23.0 (d, $J_{C\text{-}F}$ = 3.6 Hz, one isomer) & 22.6 (one isomer), 14.0 (overlap, two isomers), 13.9 & 13.9 (two isomers); **^{19}F NMR (376 MHz, $CDCl_3$)** δ -141.2 (s, one isomer), -142.8 (s, one isomer). **FT-IR**: ν (cm^{-1}) 2954, 2927, 2856, 1517, 1456, 1437, 1378, 1311, 1241, 1093, 1013. **HRMS [ESI]** calcd for $C_{21}H_{32}FNSNa^+$ $[M+Na]^+$ 372.2138, found 372.2136.

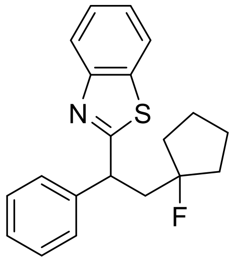

3ap

3ap: yellow solid, m.p. 92-93 °C. Purification by flash column chromatography on silica gel (eluent: EtOAc/Petroleum ether = 1/200). **^{1}H NMR (400 MHz, $CDCl_3$)** δ 8.04-7.99 (m, 1H), 7.80-7.75 (m, 1H), 7.47-7.41 (m, 3H), 7.35-7.28 (m, 3H), 7.27-7.22 (m, 1H), 4.75 (t, *J* = 6.8 Hz, 1H), 3.12 (ddd, *J* = 21.6, 14.8, 6.8 Hz, 1H), 2.62 (ddd, *J* = 21.2, 14.8, 6.8 Hz, 1H), 1.93-1.66 (m, 4H), 1.64-1.24 (m, 4H); **^{13}C NMR (100 MHz, $CDCl_3$)** δ 175.0, 153.1, 142.5, 135.3, 128.8, 128.2, 127.3, 125.8, 124.7, 122.9, 121.5, 106.2 (d, $J_{C\text{-}F}$ = 172.9 Hz), 46.8 (d, $J_{C\text{-}F}$ = 3.3 Hz), 43.7 (d, $J_{C\text{-}F}$ = 23.1 Hz), 38.0 (d, $J_{C\text{-}F}$ = 23.3 Hz), 37.9 (d, $J_{C\text{-}F}$ = 23.3 Hz), 23.6, 23.4; **^{19}F NMR (376 MHz, $CDCl_3$)** δ -144.0 (s). **FT-IR**: ν (cm^{-1}) 2960, 2923, 1593, 1454, 1394, 1213, 1189. **HRMS [ESI]** calcd for $C_{20}H_{20}FNSNa^+$ $[M+Na]^+$ 348.1193, found 348.1197.

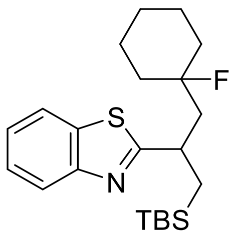

3aq

3aq: yellow oil. Purification by flash column chromatography on silica gel (eluent: EtOAc/Petroleum ether = 1/200). **^{1}H NMR (400 MHz, $CDCl_3$)** δ 7.96 (d, *J* = 8.0 Hz, 1H), 7.83 (d, *J* = 7.6 Hz, 1H), 7.48-7.39 (m, 1H), 7.38-7.29 (m, 1H), 3.70-3.52 (m, 1H), 2.46-2.30 (m, 1H), 2.12-1.98 (m, 1H), 1.92-1.80 (m, 1H), 1.79-1.69 (m, 1H), 1.65-1.22 (m, 7H), 1.22-1.09 (m, 3H), 0.85 (s, 9H), 0.06-0.00 (m, 3H), -0.25 (s, 3H); **^{13}C NMR (100 MHz, $CDCl_3$)** δ 179.1, 152.9, 134.7, 125.8, 124.6, 122.6, 121.6, 95.7 (d, $J_{C\text{-}F}$ = 171.6 Hz), 49.5 (d, $J_{C\text{-}F}$ = 21.9 Hz), 36.2, 36.1 (d, $J_{C\text{-}F}$ = 19.5 Hz), 34.9 (d, $J_{C\text{-}F}$ = 22.4 Hz), 26.4, 25.2, 22.6, 21.9 (d, $J_{C\text{-}F}$ = 2.9 Hz), 21.8 (d, $J_{C\text{-}F}$ = 2.9 Hz), 16.5, -5.7, -6.1; **^{19}F NMR (376 MHz, $CDCl_3$)** δ -154.5 (s). **FT-IR**: ν (cm^{-1}) 2930, 2855, 1517, 1437, 1247, 1153, 1095. **HRMS [ESI]** calcd for $C_{22}H_{34}FNSSiNa^+$ $[M+Na]^+$ 414.2057, found 414.2047.

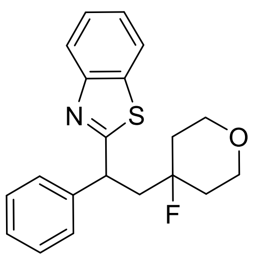

3ar

3ar: yellow oil. Purification by flash column chromatography on silica gel (eluent: EtOAc/Petroleum ether = 1/5). **^{1}H NMR (400 MHz, CDCl$_3$)** δ 8.01 (d, *J* = 8.4 Hz, 1H), 7.77 (d, *J* = 7.6 Hz, 1H), 7.47-7.38 (m, 3H), 7.36-7.29 (m, 3H), 7.27-7.21 (m, 1H), 4.74 (t, *J* = 6.4 Hz, 1H), 3.79-3.60 (m, 4H), 3.09 (ddd, *J* = 23.2, 14.8, 6.8 Hz, 1H), 2.49 (ddd, *J* = 18.8, 14.8, 6.0 Hz, 1H), 1.84-1.58 (m, 4H); **^{13}C NMR (100 MHz, CDCl$_3$)** δ 174.6, 153.0, 142.4, 135.4, 128.9, 128.0, 127.4, 126.0, 124.9, 123.0, 121.5, 93.0 (d, $J_{C\text{-}F}$ = 172.5 Hz), 63.6, 45.6 (d, $J_{C\text{-}F}$ = 21.7 Hz), 45.2 (d, $J_{C\text{-}F}$ = 3.7 Hz), 36.2 (d, $J_{C\text{-}F}$ = 21.6 Hz), 35.6 (d, $J_{C\text{-}F}$ = 21.6 Hz); **^{19}F NMR (376 MHz, CDCl$_3$)** δ -159.4 (s). **FT-IR**: ν (cm^{-1}) 2954, 2919, 2860, 1508, 1455, 1368, 1311, 1235, 1146, 1103. **HRMS [ESI]** calcd for $C_{20}H_{20}FNOSNa^+$ [M+Na]$^+$ 364.1148, found 364.1149.

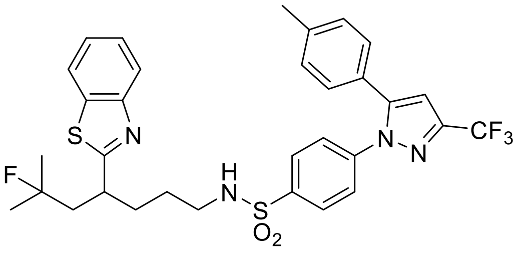

3as

3as: yellow oil. Purification by flash column chromatography on silica gel (eluent: EtOAc/Petroleum ether = 1/10). **^{1}H NMR (400 MHz, CDCl$_3$)** δ 7.92 (d, *J* = 8.0 Hz, 1H), 7.83-7.78 (m, 1H), 7.78-7.72 (m, 2H), 7.47-7.39 (m, 1H), 7.39-7.29 (m, 3H), 7.16 (d, *J* = 8.0 Hz, 2H), 7.08 (d, *J* = 8.0 Hz, 2H), 6.74 (s, 1H), 5.08 (t, *J* = 6.0 Hz, 1H), 3.44-3.34 (m, 1H), 2.99-2.87 (m, 2H), 2.37 (s, 3H), 2.35-2.20 (m, 1H), 2.11-1.98 (m, 1H), 1.93-1.77 (m, 2H), 1.54-1.37 (m, 2H),1.33 (d, *J* = 20.8 Hz, 3H), 1.27 (d, *J* = 20.8 Hz, 3H); **^{13}C NMR (100 MHz, CDCl$_3$)** δ 176.0, 152.7, 145.2, 144.0 (q, $J_{C\text{-}F}$ = 38.7 Hz), 142.3, 139.7, 139.5, 134.5, 129.7, 128.6, 127.9, 126.0, 125.6, 125.4, 124.9, 122.6, 121.6, 121.0 (q, $J_{C\text{-}F}$ = 267.5 Hz), 106.2, 95.1 (d, $J_{C\text{-}F}$ = 166.8 Hz), 46.8 (d, $J_{C\text{-}F}$ = 22.1 Hz), 42.9, 40.1 (d, $J_{C\text{-}F}$ = 2.8 Hz), 34.2, 27.8 (d, $J_{C\text{-}F}$ = 24.6 Hz), 26.9, 26.3 (d, $J_{C\text{-}F}$ = 24.7 Hz), 21.3; **^{19}F NMR (376 MHz, CDCl$_3$)** δ -62.4 (s), -136.9 (s). **FT-IR**: ν (cm^{-1}) 3281, 2979, 2929, 2869, 1597, 1471, 1373, 1235, 1156, 1133, 1094. **HRMS [ESI]** calcd for $C_{32}H_{32}F_4N_4O_2S_2Na^+$ [M+Na]$^+$ 667.1801, found 667.1803.

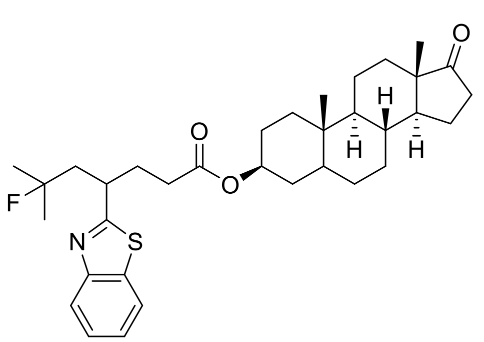

3at

3at: yellow oil. Purification by flash column chromatography on silica gel (eluent: EtOAc/Petroleum ether = 1/10). **^{1}H NMR (400 MHz, CDCl$_3$)** δ 7.96 (d, *J* = 8.0 Hz, 1H, two isomers), 7.83 (d, *J* = 7.6 Hz, 1H, two isomers), 7.44 (t, *J* = 7.2 Hz, 1H, two isomers), 7.34 (t, *J* = 7.6 Hz, 1H, two isomers), 5.04-4.90 (m, 1H), 3.56-3.44 (m, 1H, two isomers), 2.48-1.99 (m, 8H, two isomers), 1.97-1.85 (m, 1H, two isomers), 1.82-1.44 (m, 10H, two isomers), 1.34 (d, *J* = 21.2 Hz, 3H, two isomers), 1.29 (d, *J* = 21.2 Hz, 3H, two isomers), 1.26-1.11 (m, 8H, two isomers), 1.03-0.90 (m, 1H, two isomers), 0.84 (s, 3H, two isomers), 0.78 (s, 3H, two isomers); **^{13}C NMR (100 MHz, CDCl$_3$)** δ 221.3 (overlap, two isomers), 175.5 (overlap, two isomers), 172.3 & 172.3 (two isomers), 153.0 (overlap, two isomers), 134.7 (overlap, two isomers), 125.9 (overlap, two isomers), 124.9 (overlap, two isomers), 122.7 (overlap, two isomers), 121.6 (overlap, two isomers), 95.1 (d, $J_{C\text{-}F}$ = 166.0 Hz, overlap, two isomers), 70.0 (overlap, two isomers), 54.1 (overlap, two isomers), 51.4 (overlap, two isomers), 47.8 (overlap, two isomers), 46.7 (d, $J_{C\text{-}F}$ = 22.3 Hz, overlap, two isomers), 39.9 (overlap, two

isomers), 39.9 (d, $J_{C\text{-}F}$ = 3.3 Hz, overlap, two isomers), 39.9 (overlap, two isomers), 35.8 (overlap, two isomers), 35.9 (overlap, two isomers), 34.9 (overlap, two isomers), 32.8 & 32.7 (two isomers), 32.4 & 32.4 (two isomers), 32.0 & 32.0 (two isomers), 31.5 (overlap, two isomers), 30.6 & 30.6 (two isomers), 28.0 & 27.9 (two isomers), 27.8 (d, $J_{C\text{-}F}$ = 24.5 Hz, one isomer) & 27.7 (d, $J_{C\text{-}F}$ = 24.5 Hz, one isomer), 26.4 (d, $J_{C\text{-}F}$ = 24.6 Hz, overlap, two isomers), 26.0 & 26.0 (two isomers), 21.7 (overlap, two isomers), 20.0 (overlap, two isomers), 13.8 (overlap, two isomers), 11.3 (overlap, two isomers); **^{19}F NMR (376 MHz, CDCl$_3$)** δ -136.4 (s, one isomer), -136.5 (s, one isomer). **FT-IR**: ν (cm^{-1}) 2987, 2901, 1617, 1558, 1456, 1250, 1075. **HRMS [ESI]** calcd for $C_{34}H_{46}FNO_3S^+$ $[M+H]^+$ 567.3177, found 567.3178.

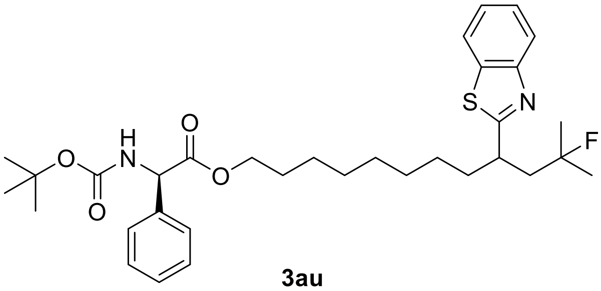

3au

3au: yellow oil. Purification by flash column chromatography on silica gel (eluent: EtOAc/Petroleum ether = 1/10). **^{1}H NMR (400 MHz, CDCl$_3$)** δ 7.97 (d, J = 8.0 Hz, 1H, two isomers), 7.85 (d, J = 8.0 Hz, 1H, two isomers), 7.44 (t, J = 7.2 Hz, 1H, two isomers), 7.38-7.27 (m, 6H, two isomers), 5.65-5.51 (m, 0.8H, one isomer), 5.44-5.33 (m, 0.2H, one isomer), 5.32-5.25 (m, 0.8H, one isomer), 5.17-5.02 (m, 0.2H, one isomer), 4.07 (t, J = 6.8 Hz, 2H, two isomers), 3.45-3.35 (m, 1H, two isomers), 2.44-2.27 (m, 1H, two isomers), 2.17-2.03 (m, 1H, two isomers), 1.85-1.74 (m, 2H, two isomers), 1.55-1.48 (m, 2H, two isomers), 1.42 (s, 9H, two isomers), 1.37-1.25 (m, 8H, two isomers), 1.24-1.19 (m, 2H, two isomers), 1.14 (s, 6H, two isomers); **^{13}C NMR (100 MHz, CDCl$_3$)** δ 177.0 (overlap, two isomers), 171.2 (overlap, two isomers), 154.8 (overlap, two isomers), 153.0 (overlap, two isomers), 137.2 (overlap, two isomers), 134.7 (overlap, two isomers), 128.8 (overlap, two isomers, 128.3 (overlap, two isomers), 127.0 (overlap, two isomers), 125.8 (overlap, two isomers), 124.7 (overlap, two isomers), 122.7 (overlap, two isomers), 121.6 (overlap, two isomers), 95.3 (d, $J_{C\text{-}F}$ = 165.5 Hz, overlap, two isomers), 80.0 (overlap, two isomers), 65.7 (overlap, two isomers), 57.6 (overlap, two isomers), 47.0 (d, $J_{C\text{-}F}$ = 22.5 Hz, overlap, two isomers), 40.9 (d, $J_{C\text{-}F}$ = 3.4 Hz, overlap, two isomers), 37.9 (overlap, two isomers), 29.2 (overlap, two isomers), 29.1 (overlap, two isomers), 28.9 (overlap, two isomers), 28.3 (overlap, two isomers), 27.8 (d, $J_{C\text{-}F}$ = 24.5 Hz, overlap, two isomers), 27.1 (overlap, two isomers), 26.4 (d, $J_{C\text{-}F}$ = 24.7 Hz, overlap, two isomers), 25.5 (overlap, two isomers); **^{19}F NMR (376 MHz, CDCl$_3$)** δ -135.9 (overlap, two isomers). **FT-IR**: ν (cm^{-1}) 3433, 2977, 2928, 2855, 1738, 1713, 1494, 1455, 1366, 1243, 1159. **HRMS [ESI]** calcd for $C_{33}H_{45}FN_2O_4SNa^+$ $[M+Na]^+$ 607.2976, found 607.2975.

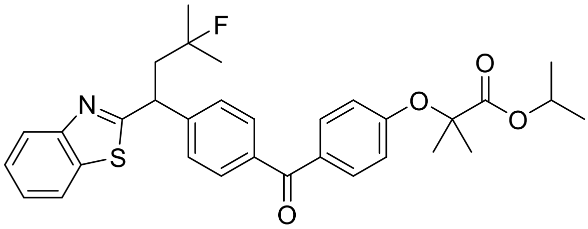

3av

3av: yellow oil. Purification by flash column chromatography on silica gel (eluent: EtOAc/Petroleum ether = 1/40). **^{1}H NMR (400 MHz, CDCl$_3$)** δ 8.02 (d, J = 8.4 Hz, 1H), 7.80 (d, J = 7.6 Hz, 1H), 7.76-7.67 (m, 4H), 7.53 (d, J = 8.0 Hz, 2H), 7.46 (t, J = 7.6 Hz, 1H), 7.34 (t, J = 7.6 Hz, 1H), 6.84 (d, J = 8.4 Hz, 2H), 5.14-5.00 (m, 1H), 4.78 (t, J = 6.4 Hz, 1H), 3.16-2.99 (m, 1H), 2.53 (ddd, J = 16.8, 14.8, 5.6 Hz, 1H), 1.65 (s, 6H), 1.36 (d, J = 21.2 Hz, 3H), 1.33 (d, J = 21.2 Hz, 3H), 1.19 (d, J = 6.0 Hz, 6H); **^{13}C NMR (100 MHz, CDCl$_3$)** δ 194.9, 173.7, 173.1, 159.5, 153.0, 146.6, 137.0, 135.3, 132.0, 130.5, 130.4, 127.9, 126.0, 125.0, 123.0, 121.6, 117.1, 95.1 (d, $J_{C\text{-}F}$ =

166.4 Hz), 79.4, 69.3, 46.3 (d, J_{C-F} = 22.4 Hz), 46.2, 27.8 (d, J_{C-F} = 24.1 Hz), 26.9 (d, J_{C-F} = 24.5 Hz), 25.4, 21.5; **^{19}F NMR (376 MHz, CDCl$_3$)** δ -137.4 (s). **FT-IR**: ν (cm^{-1}) 2980, 2934, 1728, 1650, 1597, 1505, 1373, 1279, 1246, 1174, 1143, 1099. **HRMS [ESI]** calcd for $C_{32}H_{34}FNO_4SNa^+$ [M+Na]$^+$ 570.2084, found 570.2078.

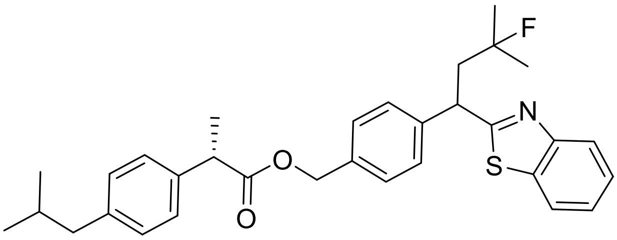

3aw

3aw: yellow oil. Purification by flash column chromatography on silica gel (eluent: EtOAc/Petroleum ether = 1/50). **^{1}H NMR (400 MHz, CDCl$_3$)** δ 8.00 (d, *J* = 8.4 Hz, 1H, two isomers), 7.78 (d, *J* = 7.6 Hz, 1H, two isomers), 7.48-7.41 (m, 1H, two isomers), 7.39-7.30 (m, 3H, two isomers), 7.22-7.14 (m, 4H, two isomers), 7.10-7.03 (m, 2H, two isomers), 5.12-5.01 (m, 2H, two isomers), 4.68 (t, *J* = 6.4 Hz, 1H, two isomers), 3.73 (q, *J* = 7.2 Hz, 1H, two isomers), 3.11-2.95 (m, 1H, two isomers), 2.54-2.45 (m, 1H, two isomers), 2.43 (dd, *J* = 7.2, 2.8 Hz, 2H, two isomers), 1.90-1.77 (m, 1H, two isomers), 1.49 (d, *J* = 7.2 Hz, 3H, two isomers), 1.33 (d, *J* = 21.2 Hz, 3H, two isomers), 1.31 (d, *J* = 21.2 Hz, 3H, two isomers), 0.89 (d, *J* = 6.4 Hz, 6H, two isomers); **^{13}C NMR (100 MHz, CDCl$_3$)** δ 174.6 (overlap, two isomers), 174.5 (overlap, two isomers), 153.0 (overlap, two isomers), 142.4 (overlap, two isomers), 140.5 (overlap, two isomers), 137.6 (overlap, two isomers), 135.3 (overlap, two isomers), 135.1 (overlap, two isomers), 129.3 (overlap, two isomers), 128.3 (overlap, two isomers), 128.1 (overlap, two isomers), 127.2 (overlap, two isomers), 125.9 (overlap, two isomers), 124.9 (overlap, two isomers), 122.9 (overlap, two isomers), 121.5 (overlap, two isomers), 95.1 (d, J_{C-F} = 167.3 Hz, overlap, two isomers), 65.9 (overlap, two isomers), 46.3 (d, J_{C-F} = 22.6 Hz, overlap, two isomers), 46.0 (d, J_{C-F} = 4.1 Hz, overlap, two isomers), 45.1 (overlap, two isomers), 45.0 (overlap, two isomers), 30.2 (overlap, two isomers), 27.7 (d, J_{C-F} = 24.6 Hz, overlap, two isomers), 27.0 (d, J_{C-F} = 24.6 Hz, overlap, two isomers), 22.4 (overlap, two isomers), 18.4 (overlap, two isomers); **^{19}F NMR (376 MHz, CDCl$_3$)** δ -137.0 (s, one isomer), -137.0 (s, one isomer). **FT-IR**: ν (cm^{-1}) 2976, 2953, 2929, 1733, 1511, 1436, 1373, 1156. **HRMS [ESI]** calcd for $C_{32}H_{36}FNO_2SNa^+$ [M+Na]$^+$ 540.2349, found 540.2350.

4. Product Transformations

4.1 Synthesis of 4 [1]

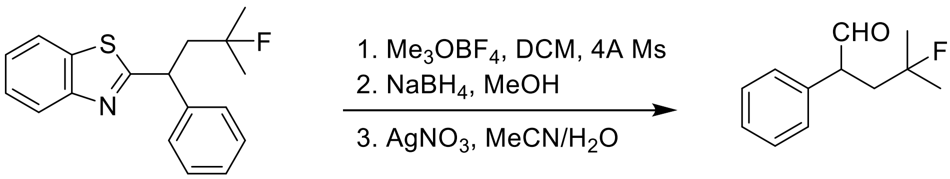

3a 4

3a (0.1 mmol), activated 4 Å molecular sieves powders (150 mg), and anhydrous DCM (1 mL) were stirred at rt for 10 min, and then Me_3OBF_4 (0.25 mmol) was added. After stirred at rt for 2 h, another batch of Me_3OBF_4 (0.25 mmol) was added to the suspension, which was continued to react until **3a** was fully consumed as determined by TLC. The reaction was concentrated without filtering off the molecular

sieves to give the crude N-methylbenzothiazolium salt. The residue was re-dissolve in MeOH (1 mL), which was then cooled to 0 °C and added $NaBH_4$ (0.15 mmol). Another batch of $NaBH_4$ (0.1 mmol) was added to the reaction until the starting material had been consumed as determined by TLC. The mixture was diluted with acetone, filtered through a pad of Celite, and concentrated to give the crude benzothiazolines. To a vigorously stirred solution of the crude benzothiazolines in CH_2Cl_2 (0.3 mL) and CH_3CN (1.5 mL) were added H_2O (0.18 mL) followed by $AgNO_3$ (0.3 mmol). The mixture was stirred at rt (monitored by TLC), and then diluted with 1 M phosphate buffer at pH 7 (0.1 mL). After stirred for 15 min, the reaction mixture was diluted with 1 M phosphate buffer at pH 7 (2.5 mL) and partially concentrated to remove CH_3CN. The suspension was extracted with EtOAc, and the combined organic layers were dried over Na_2SO_4, filtered through a pad of Celite, and concentrated. The residue was purified by flash column chromatography on silica gel to afford product **4**.

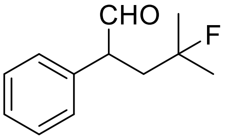

4

4: 67% yield, colorless oil. Purificati on by flash column chromatography on silica gel (eluent: EtOAc/Petroleum ether = 1/200). **^{1}H NMR (400 MHz, $CDCl_3$)** δ 9.68-9.63 (m, 1H), 7.41-7.34 (m, 2H), 7.33-7.27 (m, 1H), 7.25-7.20 (m, 2H), 3.84 (ddd, J = 7.2, 5.6, 2.0 Hz, 1H), 2.71 (ddd, J = 18.8, 14.4, 7.2 Hz, 1H), 1.96 (ddd, J = 20.4, 14.4, 5.6 Hz, 1H), 1.36 (d, J = 21.6 Hz, 3H), 1.33 (d, J = 21.6 Hz, 3H); **^{13}C NMR (100 MHz, $CDCl_3$)** δ 199.4, 136.6, 129.2, 128.8, 127.7, 94.9 (d, J_{C-F} = 165.5 Hz), 54.6 (d, J_{C-F} = 2.8 Hz), 40.6 (d, J_{C-F} = 22.4 Hz), 27.6 (d, J_{C-F} = 24.1 Hz), 26.9 (d, J_{C-F} = 24.5 Hz); **^{19}F NMR (376 MHz, $CDCl_3$)** δ -137.7 (s). **FT-IR**: ν (cm^{-1}) 3063, 3030, 2935, 1722, 1492, 1454, 1387, 1136, 1077. **HRMS [ESI]** calcd for $C_{12}H_{15}FONa^+$ $[M+Na]^+$ 217.1005, found 217.0999.

4.2 Synthesis of 5 [2]

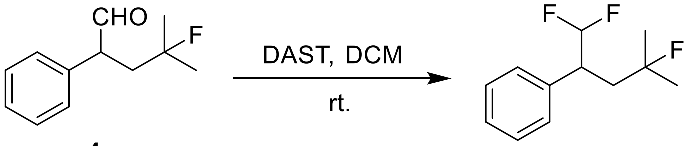

The solution of **4** (0.1 mmol) and diethylaminosulfur trifluoride (DAST) (0.12 mmol) in anhydrous DCM (2 mL) was stirred at rt for 4 h, and then concentrated. The residue was purified by flash column chromatography on silica gel to afford product **5**.

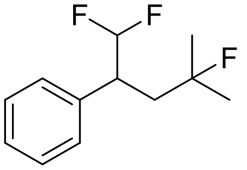

5

5: 76% yield, colorless oil. Purification by flash column chromatography on silica gel (eluent: EtOAc/Petroleum ether = 1/200). **^{1}H NMR (400 MHz, $CDCl_3$)** δ 7.31-7.24 (m, 2H), 7.24-7.17 (m, 3H), 5.79 (td, J = 56.8, 3.2 Hz, 1H), 3.29-3.11 (m, 1H), 2.33-2.18 (m, 1H), 2.04 (ddd, J = 24.0, 14.8, 8.8 Hz, 1H), 1.25 (d, J = 21.5 Hz, 3H), 1.14 (d, J = 21.3 Hz, 3H); **^{13}C NMR (100 MHz, $CDCl_3$)** δ 137.5 (dd, J = 4.8, 2.5 Hz), 129.1, 128.7, 127.6, 117.4 (td, J = 244.9, 1.8 Hz), 94.9 (d, J = 167.2 Hz), 45.7 (td, J = 19.8, 3.8 Hz), 39.4 (ddd, J = 22.9, 4.9, 3.0 Hz), 28.0 (d, J = 24.5 Hz), 26.6 (d, J = 24.8 Hz); **^{19}F NMR (376 MHz, $CDCl_3$)** δ -120.7 (d, J = 293.2 Hz), -122.2 (d, J = 293.2 Hz), -137.4 (s). **FT-IR**: ν (cm^{-1}) 3158, 3012, 2935, 1692, 1454, 1350, 1148, 1043. **HRMS [ESI]** calcd for $C_{12}H_{15}F_3Na^+$ $[M+Na]^+$ 239.1018, found 239.1020.

4.3 Synthesis of 6 [3]

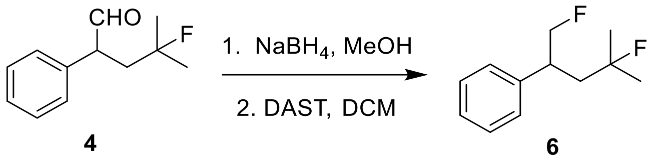

The solution of **4** (0.1 mmol) and $NaBH_4$ (1.2 equiv.) in MeOH (1 mL) were stirred at rt for 4 h, and then concentrated. Next, the diethylaminosulfur trifluoride (DAST) (0.12 mmol) in anhydrous DCM (2 mL) were added to the residue. The reaction was stirred at rt for 8 h, and then concentrated. The residue was purified by flash column chromatography on silica gel to afford product **6**.

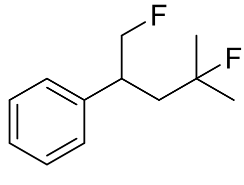

6

6: 67% yield, colorless oil. Purification by flash column chromatography on silica gel (eluent: EtOAc/Petroleum ether = 1/200). **^{1}H NMR (400 MHz, $CDCl_3$)** δ 7.36-7.29 (m, 2H), 7.27-7.19 (m, 3H), 5.10-4.80 (m, 1H), 3.12-2.78 (m, 2H), 2.10-1.77 (m, 2H), 1.42 (d, J = 9.6 Hz, 3H), 1.37 (d, J = 9.6 Hz, 3H); **^{13}C NMR (100 MHz, $CDCl_3$)** δ 136.8 (d, J = 4.4 Hz), 129.4, 128.5, 126.7, 94.4 (d, J = 165.5 Hz), 90.8 (dd, J = 171.0, 5.5 Hz), 45.7 (dd, J = 22.9, 20.2 Hz), 42.3 (d, J = 21.7 Hz), 28.4 (dd, J = 24.1, 1.6 Hz), 26.0 (dd, J = 24.8, 2.5 Hz); **^{19}F NMR (376 MHz, $CDCl_3$)** δ -135.9 (d, J = 4.0 Hz), -176.2 (d, J = 3.1 Hz). **FT-IR**: ν (cm^{-1}) 3201, 2975, 1692, 1454, 1353, 1146, 1074. **HRMS [ESI]** calcd for $C_{12}H_{16}F_2Na^+$ $[M+Na]^+$ 221.1112, found 221.1110.

4.4 Synthesis of 7 [4]

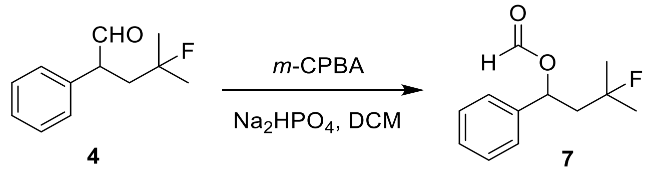

A stirred solution of the starting material **4** (0.1 mmol), Na_2HPO_4 (1.2 equiv.) in DCM (10 mL) was added with *m*-CPBA (3 equiv.) slowly at 0 °C, then the reaction mixture warmed to room temperature, stirred for 12 h. After the reaction was complete, the mixture was added by saturated aq. $NaHCO_3$ for 1 h at 0 °C. After filtration by diatomite, the filter residue was washed with DCM. Then the organic phase was combined and dried over anhydrous $MgSO_4$. The solvent was removed under vacuum and the residue was purified by flash column chromatography on silica gel to provide the product **7**.

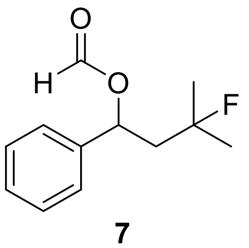

7

7: 61% yield, colorless oil. Purification by flash column chromatography on silica gel (eluent: EtOAc/Petroleum ether = 1/20). **^{1}H NMR (400 MHz, $CDCl_3$)** δ 7.99 (s, 1H), 7.40-7.20 (m, 5H), 6.05 (dd, J = 8.8, 3.6 Hz, 1H), 2.32 (ddd, J = 20.4, 15.2, 8.8 Hz, 1H), 2.03 (ddd, J = 18.8, 15.2, 3.6 Hz, 1H), 1.35 (d, J = 6.4 Hz, 3H), 1.30 (d, J = 6.4 Hz, 3H); **^{13}C NMR (100 MHz, $CDCl_3$)** δ 160.1, 140.4, 128.6, 128.3, 126.5, 94.1 (d, J = 167.2 Hz), 72.2 (d, J = 5.0 Hz), 47.2 (d, J = 23.0 Hz), 27.4 (d, J = 24.4 Hz), 26.9 (d, J = 24.7 Hz); **^{19}F NMR (376 MHz, $CDCl_3$)** δ -136.9. **FT-IR**: ν (cm^{-1}) 3202, 3030, 2594, 1735, 1592, 1350, 1121, 1050. **HRMS [ESI]** calcd for $C_{12}H_{15}FO_2Na^+$ $[M+Na]^+$ 233.0948, found 233.0950.

4.5 Synthesis of 8 [5]

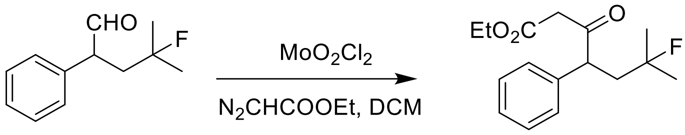

A stirred solution of the starting material **4** (0.1 mmol), MoO_2Cl_2 (5 mol %) in DCM (10 mL) was stirred at 30 °C under N_2. The conversion was monitored by TLC. After reaction completion, DCM was evaporated and the crude mixture was diluted with H_2O, extracted with EtOAc and and dried over anhydrous $MgSO_4$. The EtOAc was evaporated and the residue was purified by flash column chromatography on silica gel to provide the product **8**.

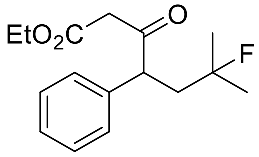

8

8: 58% yield, colorless oil. Purification by flash column chromatography on silica gel (eluent: EtOAc/Petroleum ether = 1/20). **^{1}H NMR (400 MHz, $CDCl_3$)** δ 7.41-7.15 (m, 5H), 4.26-4.01 (m, 3H), 3.39 (q, *J* = 15.2, 2H), 2.76 (ddd, *J* = 21.6, 14.8, 7.6 Hz, 1H), 1.98-1.83 (m, 1H), 1.33 (d, *J* = 3.2 Hz, 3H), 1.27 (d, *J* = 2.8 Hz, 3H), 1.21 (t, *J* = 6.8 Hz, 2H); **^{13}C NMR (100 MHz, $CDCl_3$)** δ 201.5, 166.9, 138.5, 129.2, 128.4, 127.6, 94.9 (d, *J* = 166.9 Hz), 61.3, 53.9 (d, *J* = 3.5 Hz), 48.1, 42.8 (d, *J* = 22.3 Hz), 27.3 (d, *J* = 6.8 Hz), 27.1 (d, *J* = 6.8 Hz), 13.7; **^{19}F NMR (376 MHz, $CDCl_3$)** δ -138.8. **FT-IR**: ν (cm^{-1}) 3218, 2870, 1748, 1622, 1592, 1387, 1236, 1032. **HRMS [ESI]** calcd for $C_{16}H_{21}FO_3Na^+$ $[M+Na]^+$ 303.1367, found 303.1365.

5. Stern-Volmer Studies

Emission intensities were recorded using a FLS980 (Edinburgh Instrument, UK) luminescence spectrophotometer. All $[Ir(dF(CF_3)ppy)_2(dtbbpy)]PF_6$ solutions were excited at 425 nm and the emission intensity was collected at 565 nm. In a typical experiment, to a $3{\cdot}10^{-6}$ M solution of $[Ir(dF(CF_3)ppy)_2(dtbbpy)]PF_6$ in CH_2Cl_2 was added the appropriate amount of a quencher the mixtures (**2a** and $KHCO_3$) in a screw-top quartz cuvette to obtain a total volume of 3.0 mL. After degassing the sample with a stream of N_2 for 10 minutes, the emission of the sample was collected. Then, another quencher Selectfluor® was test in the same way.

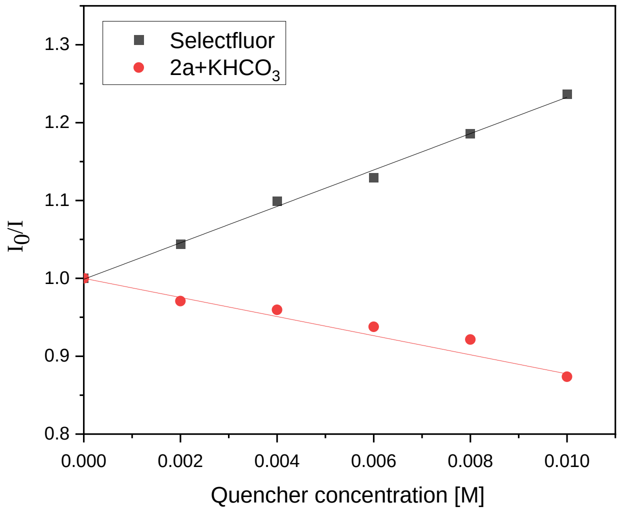

Supplementary Fig. 1 Stern-Volmer quenching plot

6. UV-vis experiments

Solutions of different complexes were introduced to a 1 cm path length quartz cuvette equipped witha Teflon® septum and analyzed using an ultraviolet spectrometer (Agilent Technologies Cary 5000 UV-vis-NIR). For **2a** (0.4 mmol), Selectfluor® (0.4 mmol), the mixtures (**2a** (0.4 mmol) and $KHCO_3$ (1.2 mmol)) and the mixtures ((**2a** (0.25 mmol), $KHCO_3$ (0.75 mmol) and Selectfluor® (0.75 mmol)) were dissolved in MeCN (4 mL) respectively. The mixtures were stirred for 5 min, then transformed to 1 cm path length quartz cuvettes, sealed with Teflon® septa and degassed with a stream of argon for 10 minutes.

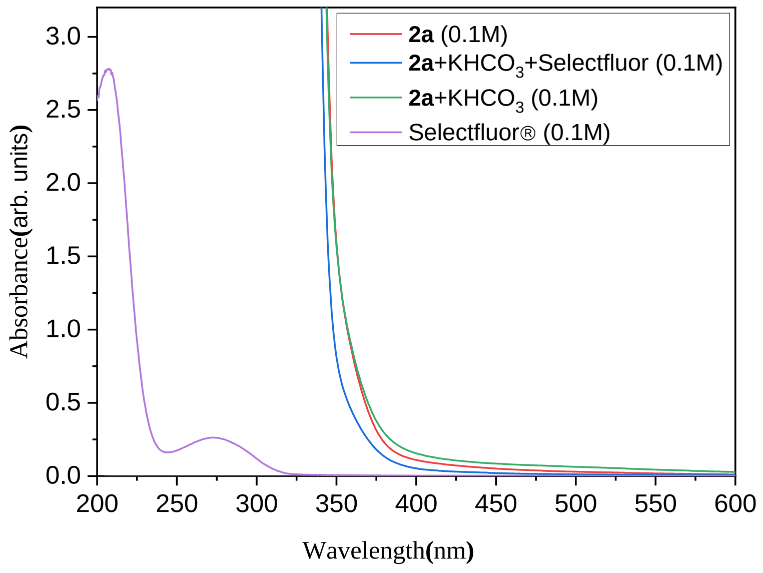

Supplementary Fig. 2 UV-vis absorption spectra

7. Cyclic voltammograms

All voltammograms were taken at room temperature using a mesh platinum (Pt) counter electrode, a glassy carbon working electrode (3 mm diameter), and a saturated calomel (SCE) reference electrode. The conditions of the experiments were the following: an acetonitrile solution of 0.1 M tetrabutylammonium tetrafluoroborate (Bu_4NBF_4) as the supporting electrolyte and 0.01 M **2a** and $KHCO_3$, a scan rate of 0.1 V/s, and a negative initial scan direction. The reported potentials were averages over segments, and were taken at half-height of the cathodic peaks (Ep/2) of **2a**, since all reductions were non-reversible.

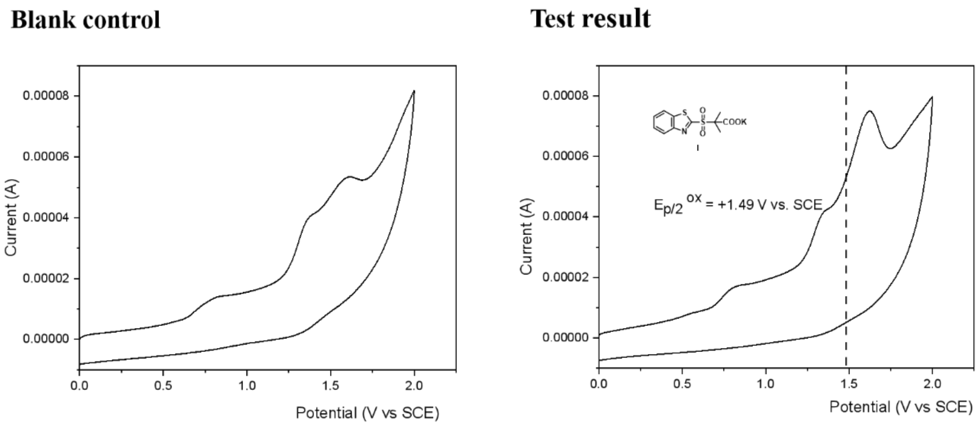

Supplementary Fig. 3 Cyclic voltammogram of **2a** in MeCN.

8. Quantum yield measurements

Determination of the light intensity at 456 nm

The photon flux of the kessil light (40 W, λ max = 456 nm) was determined by standard ferrioxalate actinometry following a modified literature procedure of Yoon[2] and Glorius[3]. A 0.15 M solution of ferrioxalate was prepared by dissolving potassium ferrioxalate hydrate (0.737 g) in H_2SO_4 (10 mL of a 0.05 M solution). A buffered solution of 1,10-phenanthroline was prepared by dissolving 1,10-phenanthroline (5.0 mg) and sodium acetate (1.13 g) in H_2SO_4 (5.0 mL of a 0.5 M solution). Both solutions were stored in the dark. To determine the photon flux of the LED, the ferrioxalate solution (3.0 mL) was placed in a cuvette and irradiated for 60 seconds at λmax = 456 nm. After irradiation, the phenanthroline solution (0.525 mL) was added to the cuvette and the mixture was allowed to stir in the dark for 1 h to allow the ferrous ions to completely coordinate to the phenanthroline. The absorbance of the solution was measured at 510 nm. The same procedure was repeated two more times. A nonirradiated sample was also prepared and the absorbance at 510 nm was measured. The average of the absorption of the irradiated and non-irradiated samples was determined and used to calculate the generated amount of Fe(II) according to the Lambert-Beer law (equation 1),

$$\textbf{mol Fe}^{2+} = (V \times \Delta A_{510nm}) / (l \times \varepsilon) \qquad (1)$$

where V is the total volume ($3.525 * 10^{-3}$ L), ΔA_{510nm} the difference between absorbance of irradiated samples and the non-irradiated (control) ones (at λ = 510 nm), l is the path length of the cuvette (1.0 cm),

and ε is the molar attenuation coefficient of the ferrioxalate actinometer λ = 510 nm (11100 L· mol–1·cm–1)[4]. The photonflux (ϕq) can be calculated using equation 2,

$$\textbf{photo flux} = \text{mol } Fe^{2+} / (\Phi_F \times t \times f) \qquad (2)$$

where Φ_F is the quantum yield of the ferrioxalate actinometer (1.11 at λ = 436 nm)[5] and t is the irradiation time (60 s). The fraction of light absorbed at λ = 456 nm by the actinometer (f) is calculated by using equation 3. A427nm is the absorbance of the ferrioxalate solution at λ = 456 nm.

$$f = 1 - 10^{-}A_{456\ nm} \qquad (3)$$

The absorbance (A_{456nm}) of the ferrioxalate solution was measured to be > 3 indicating that > 99.9% of the photons are absorbed (f > 0.999). The photon flux Φq was therefore calculated to be $5.3*10^{-9}$ einstein s^{-1} as an average of three experiments.

Determination of the reaction quantum yield

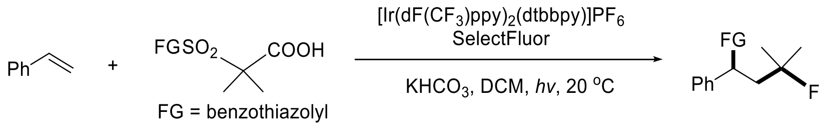

1a **2a** **3a**

The reaction mixture was stirred and irradiated by blue LED (λmax = 456 nm) for 7200 s. The yield of product was determined by ^1H NMR analysis using 1,3,5-Trimethoxybenzene as an internal standard. The yield of **3a** was determined to be 21% (0.021×10^{-3} mol of **3a**). The reaction quantum yield (Φ) was determined using equation 4 where the photon flux is 5.3×10^{-9} einsteins s^{-1} (determined by actinometry as described above), t is the reaction time (7200 s) and f is the fraction of incident light absorbed by the catalyst, determined using (equation 3).

$$\textbf{Quantum Yield} = \text{moles of product formed} / (\text{flux} \times f \times t) \qquad (4)$$

$$= 0.021 \times 10^{-3} / (5.3 \times 10^{-9} \times 1 \times 7200) = 0.55$$

9. ^{1}H, ^{19}F, ^{13}C NMR spectra

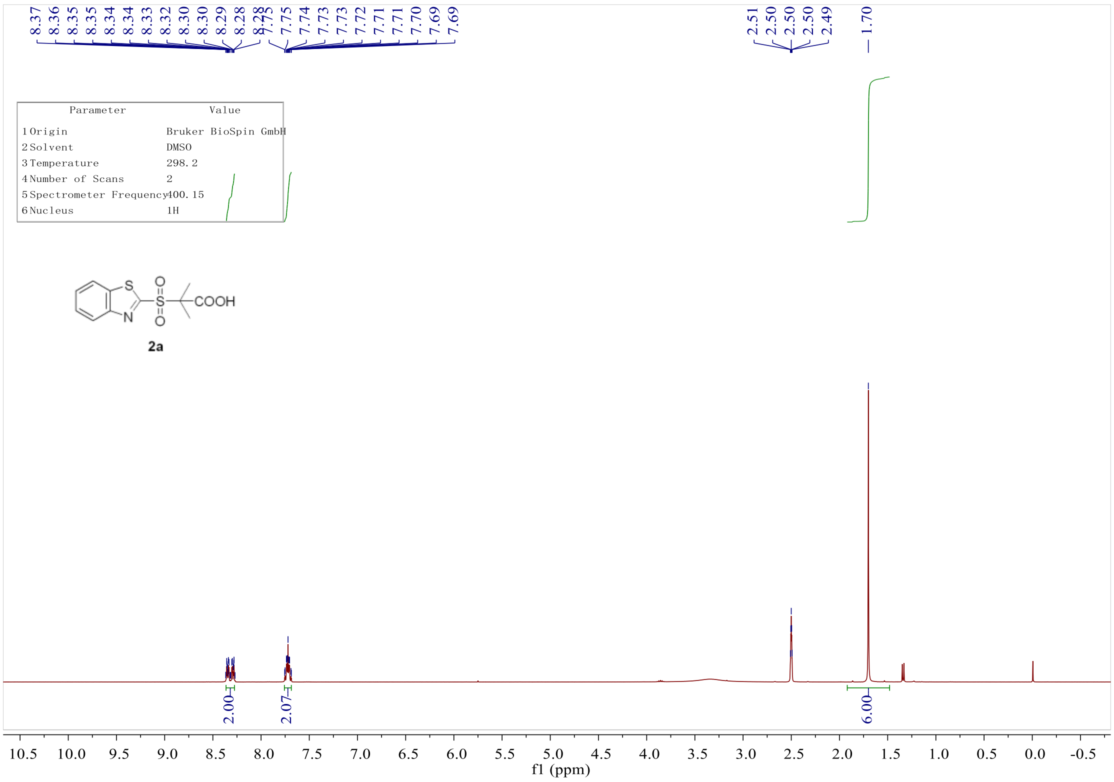

Supplementary Fig. 4 ^{1}H NMR spectra (400 MHz, DMSO, 25 ºC) of **2a**

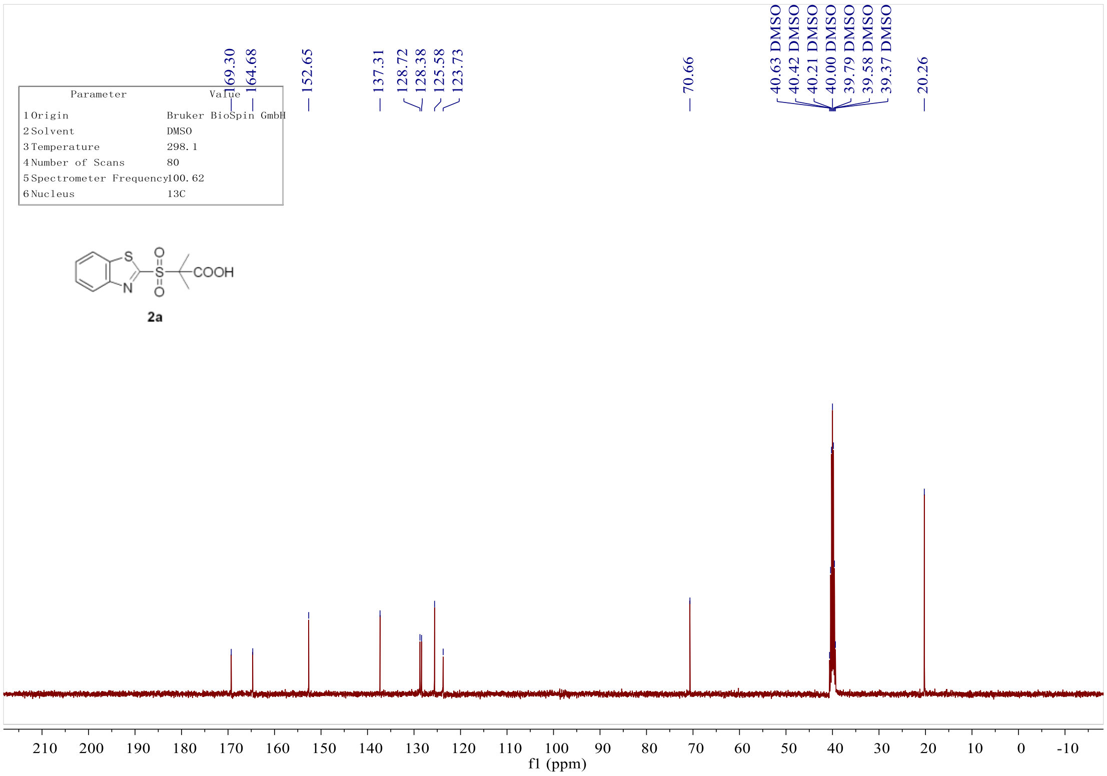

Supplementary Fig. 5 ^{13}C NMR spectra (100 MHz, DMSO, 25 ºC) of **2a**

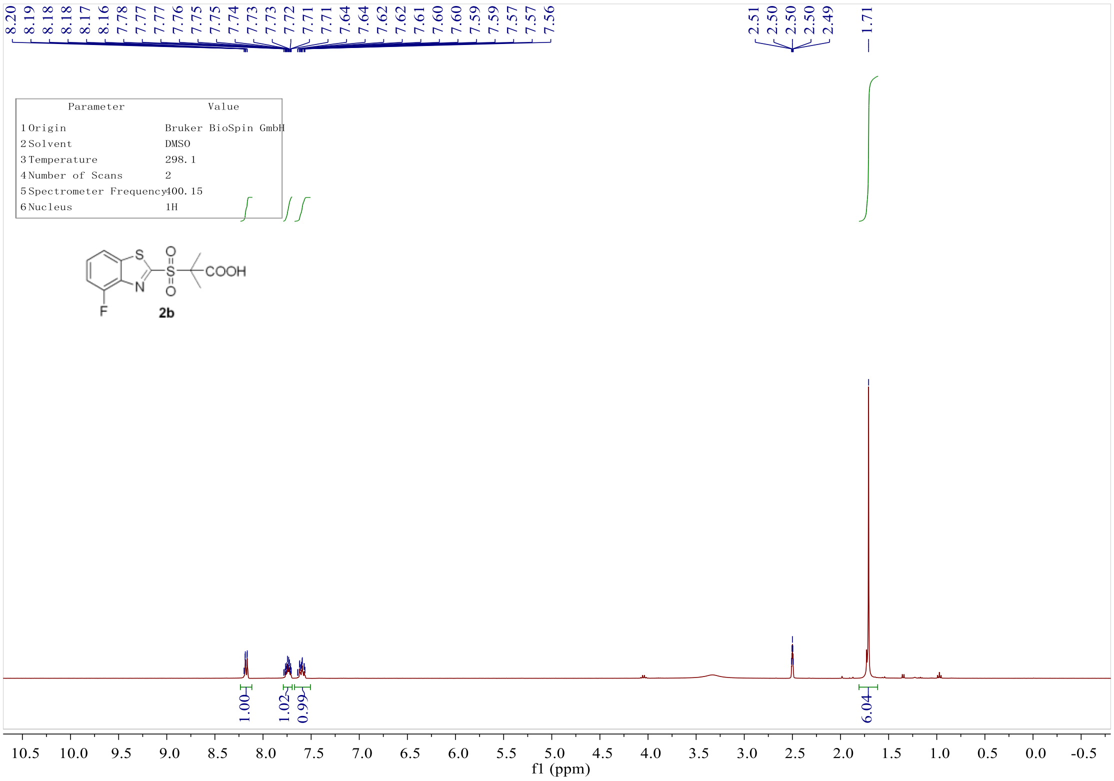

Supplementary Fig. 6 ^{1}H NMR spectra (400 MHz, DMSO, 25 ºC) of **2b**

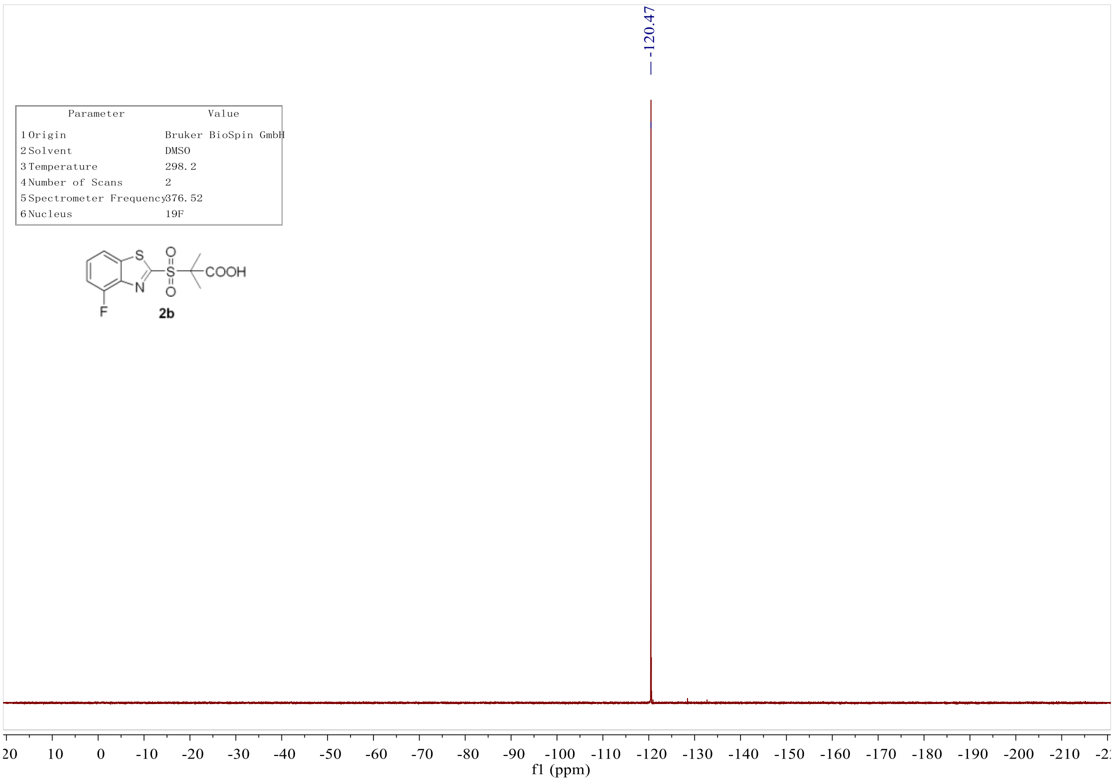

Supplementary Fig. 7 ^{19}F NMR spectra (376 MHz, DMSO, 25 ºC) of **2b**

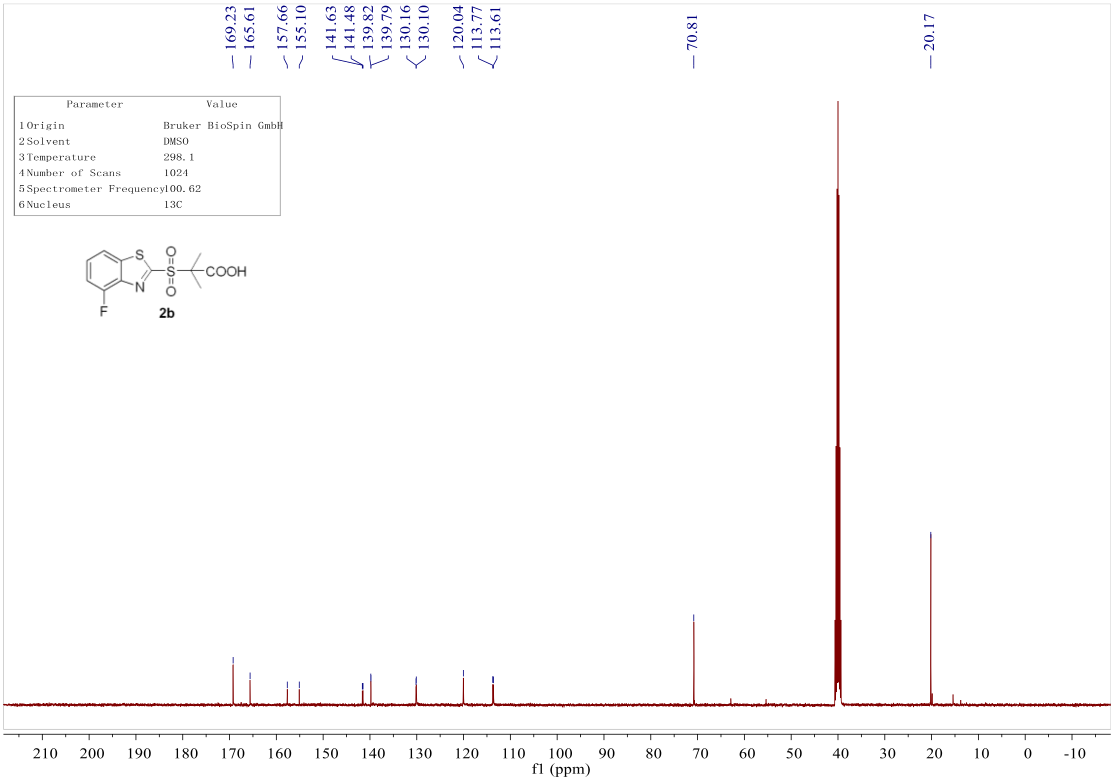

Supplementary Fig. 8 ^{13}C NMR spectra (100 MHz, DMSO, 25 ºC) of **2b**

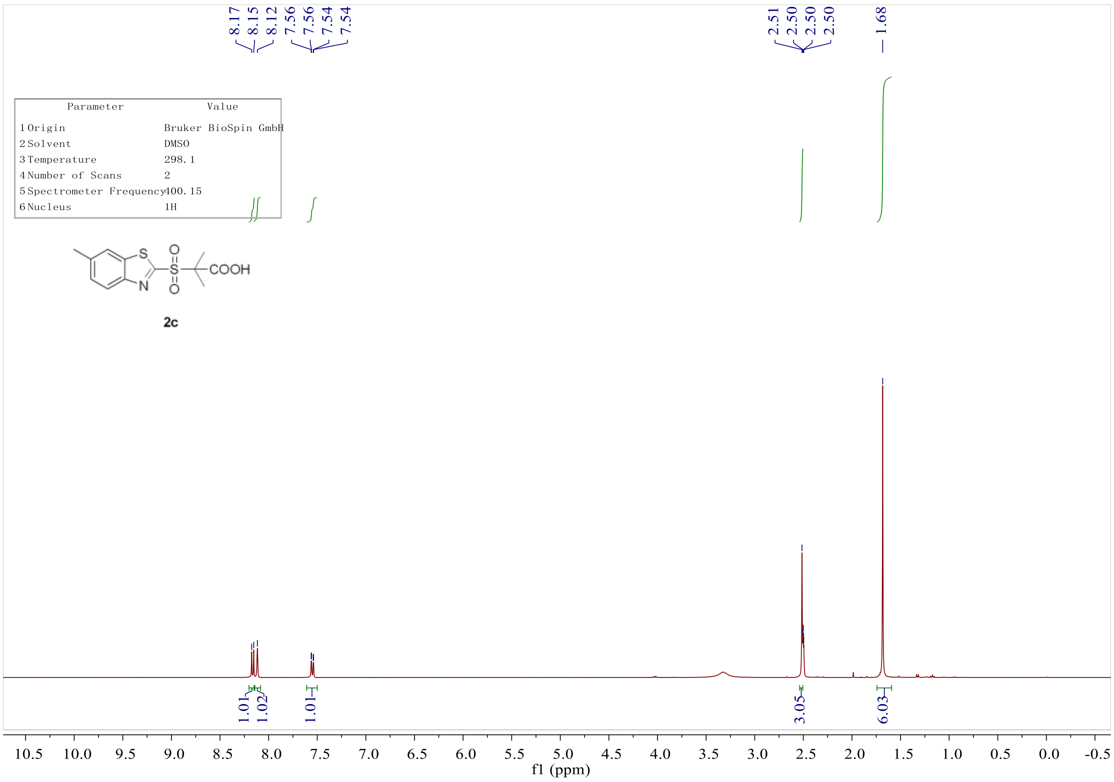

Supplementary Fig. 9 ^{1}H NMR spectra (400 MHz, DMSO, 25 ºC) of **2c**

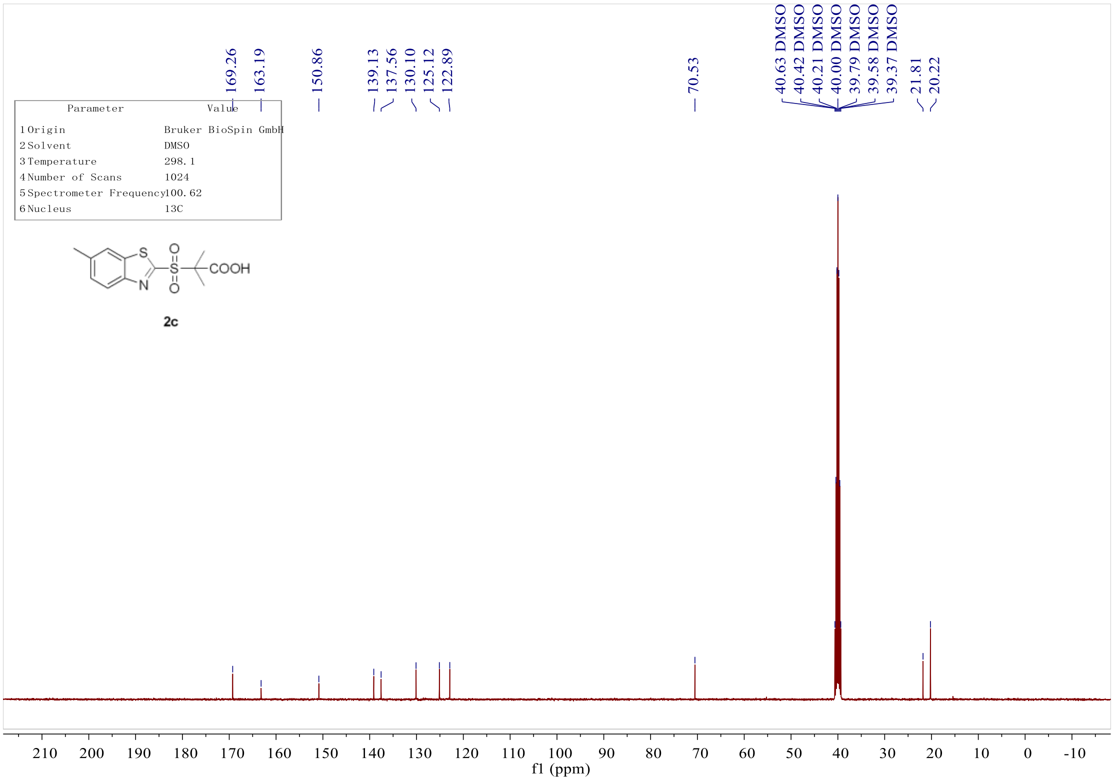

Supplementary Fig. 10 ^{13}C NMR spectra (100 MHz, DMSO, 25 °C) of **2c**

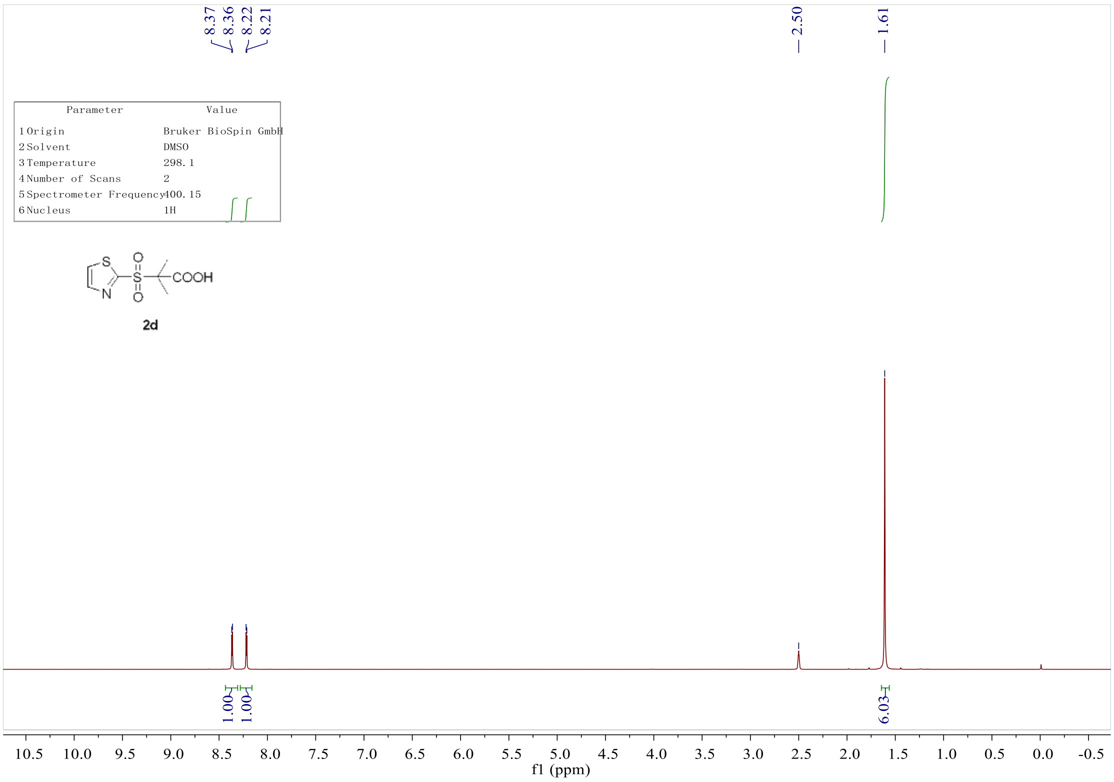

Supplementary Fig. 11 ^{1}H NMR spectra (400 MHz, DMSO, 25 °C) of **2d**

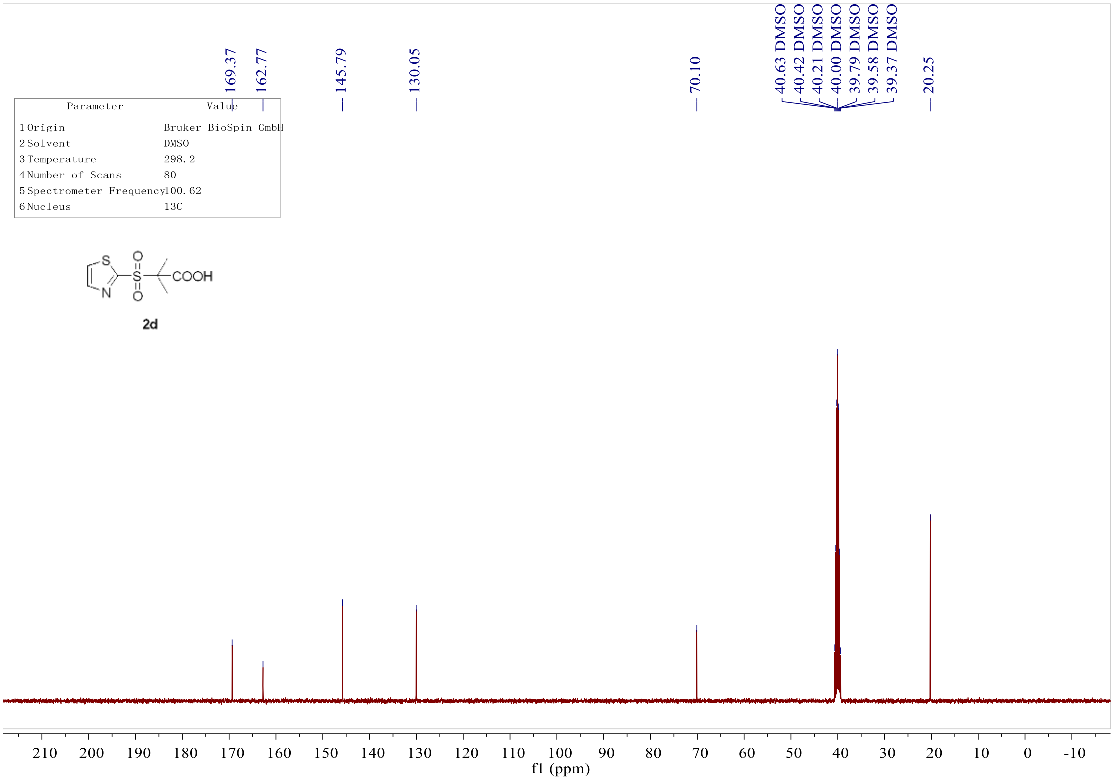

Supplementary Fig. 12 ^{13}C NMR spectra (100 MHz, DMSO, 25 °C) of **2d**

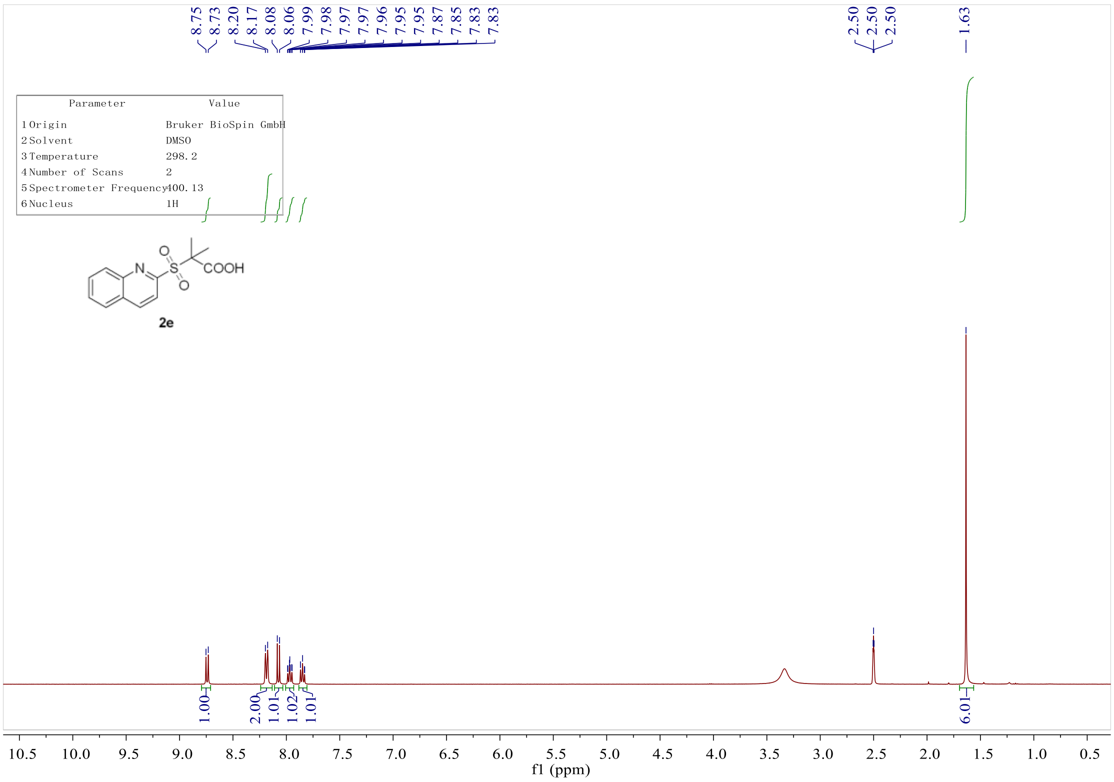

Supplementary Fig. 13 ^{1}H NMR spectra (400 MHz, DMSO, 25 °C) of **2e**

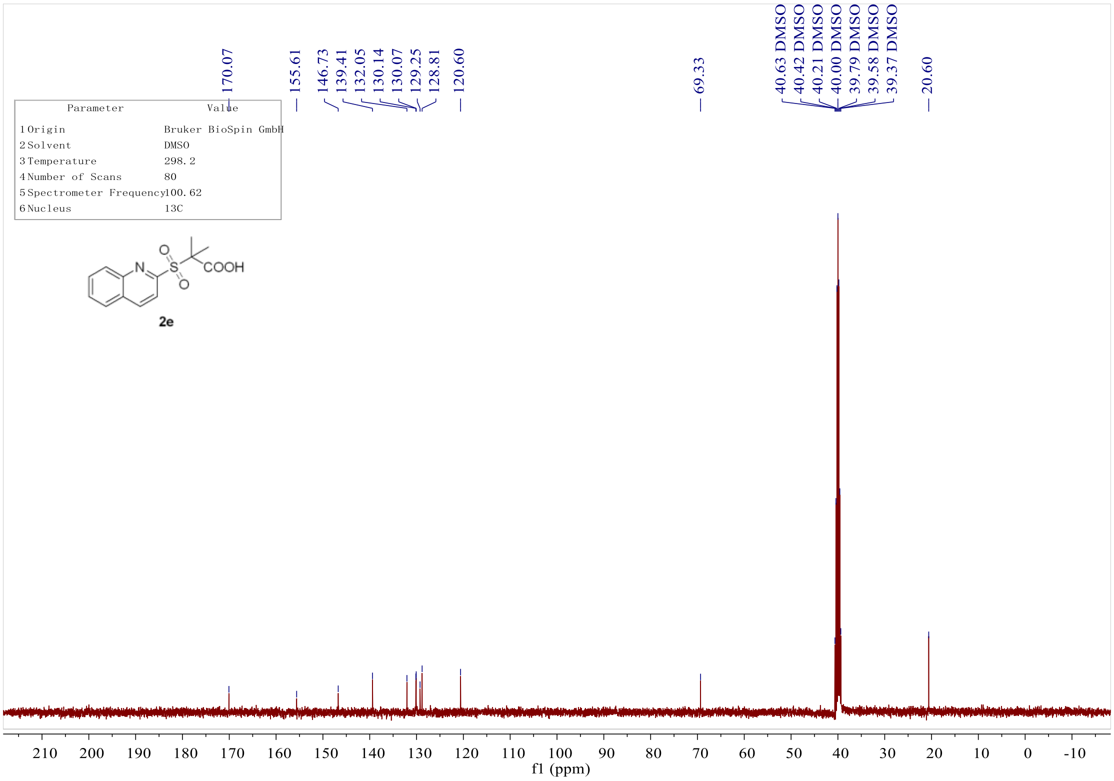

Supplementary Fig. 14 ^{13}C NMR spectra (100 MHz, DMSO, 25 ºC) of **2e**

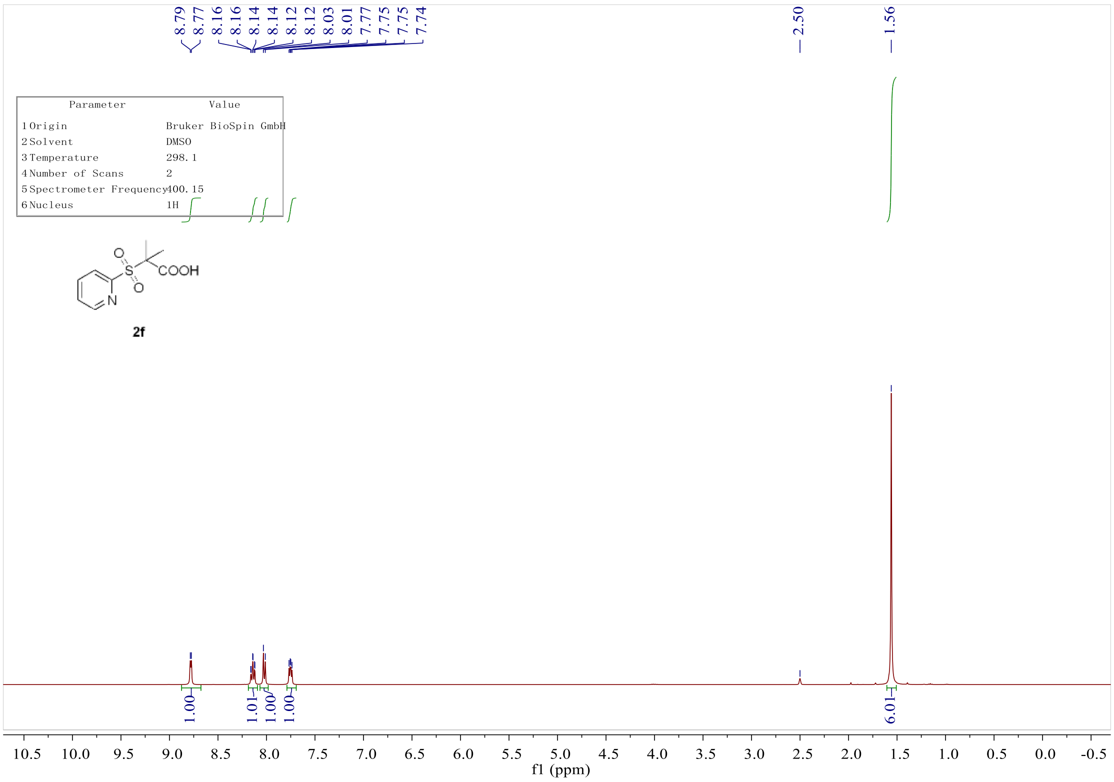

Supplementary Fig. 15 ^{1}H NMR spectra (400 MHz, DMSO, 25 ºC) of **2f**

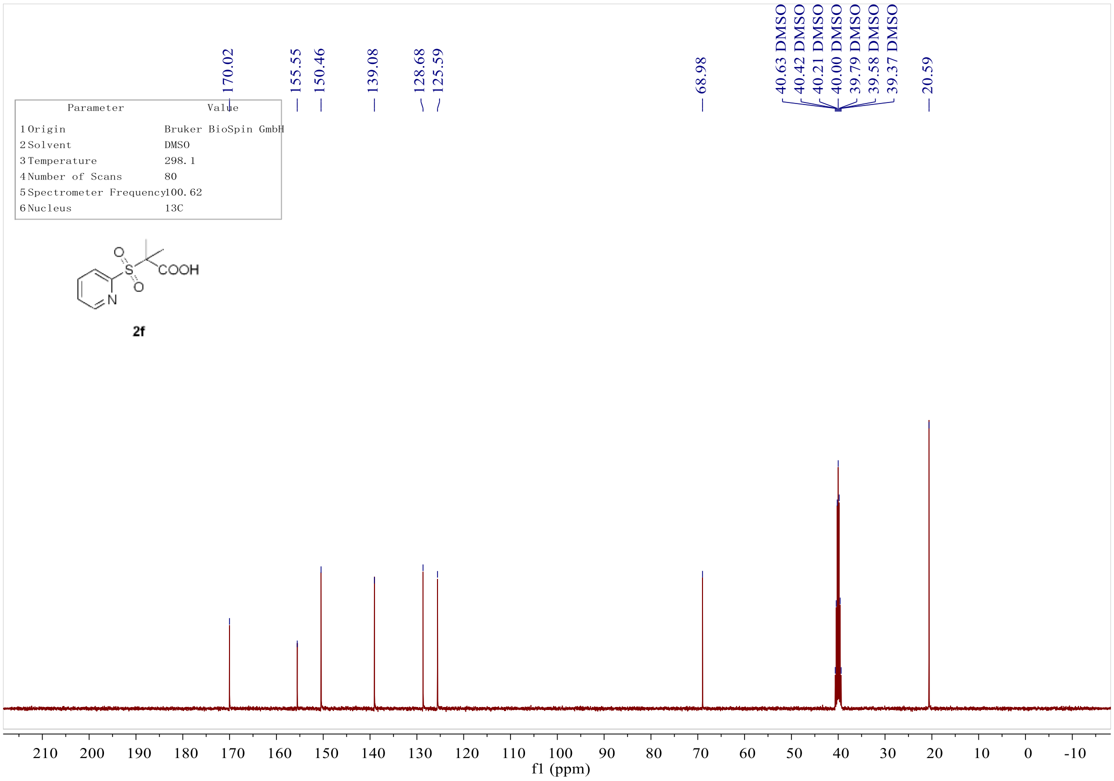

Supplementary Fig. 16 ^{13}C NMR spectra (100 MHz, DMSO, 25 °C) of **2f**

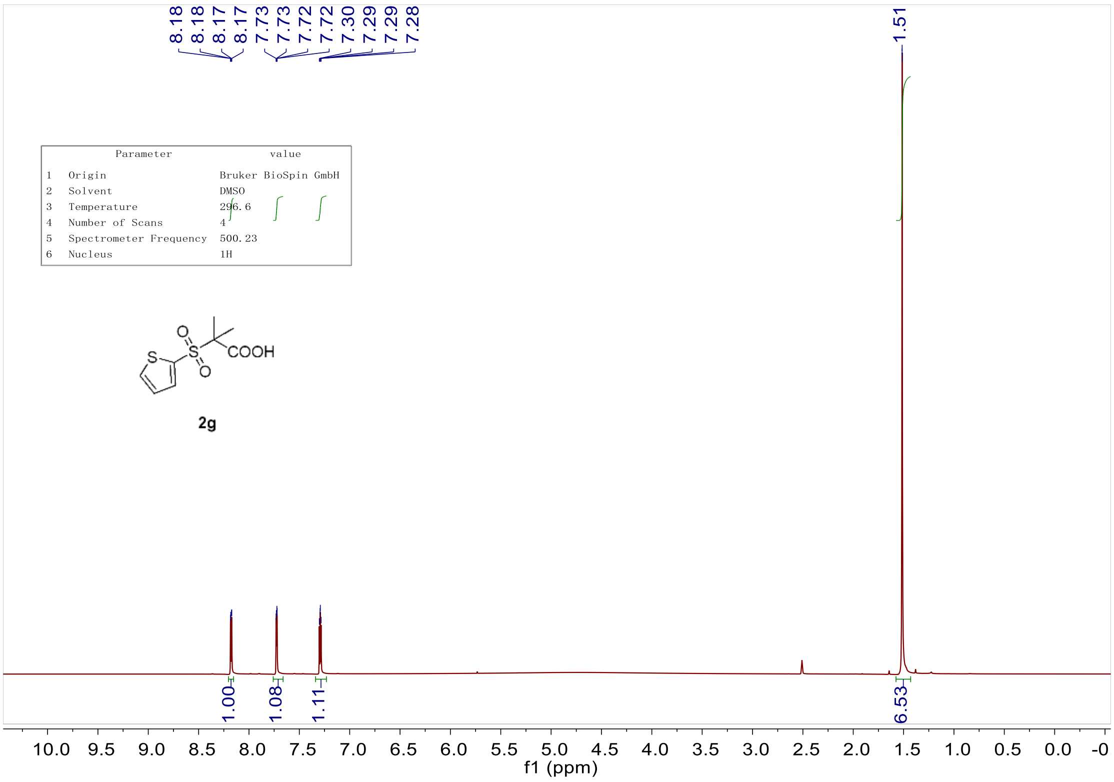

Supplementary Fig. 17 ^{1}H NMR spectra (500 MHz, DMSO, 25 °C) of **2g**

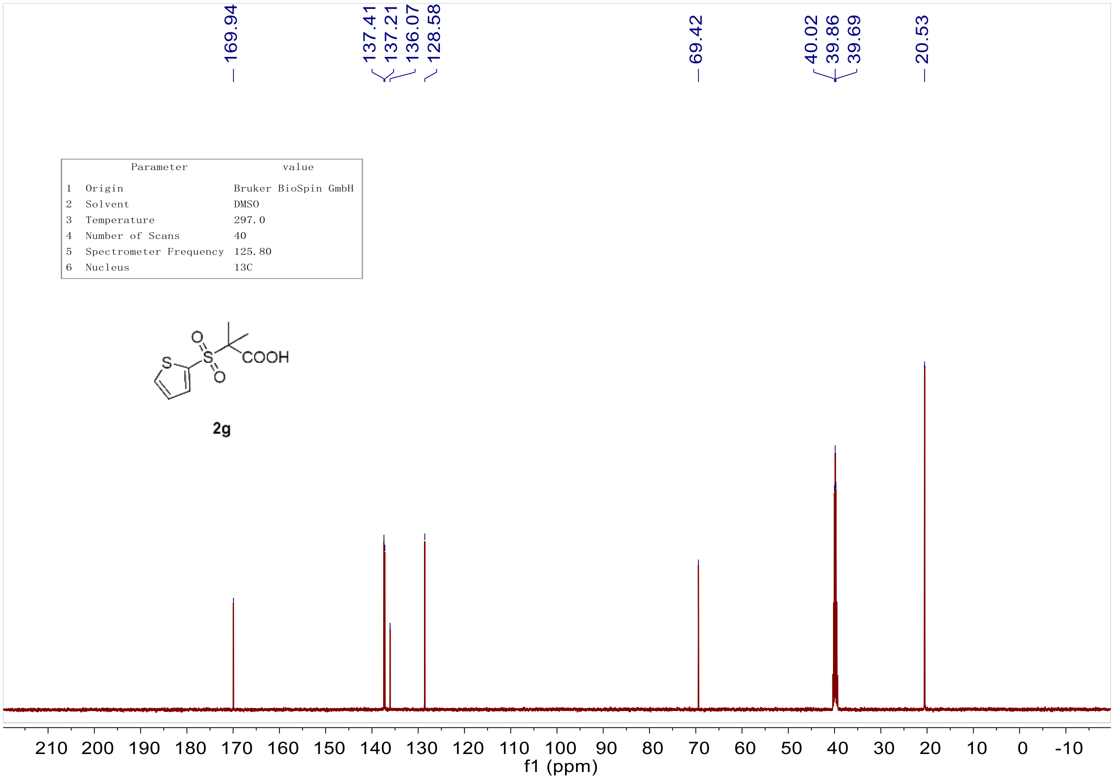

Supplementary Fig. 18 ^{13}C NMR spectra (125 MHz, DMSO, 25 °C) of **2g**

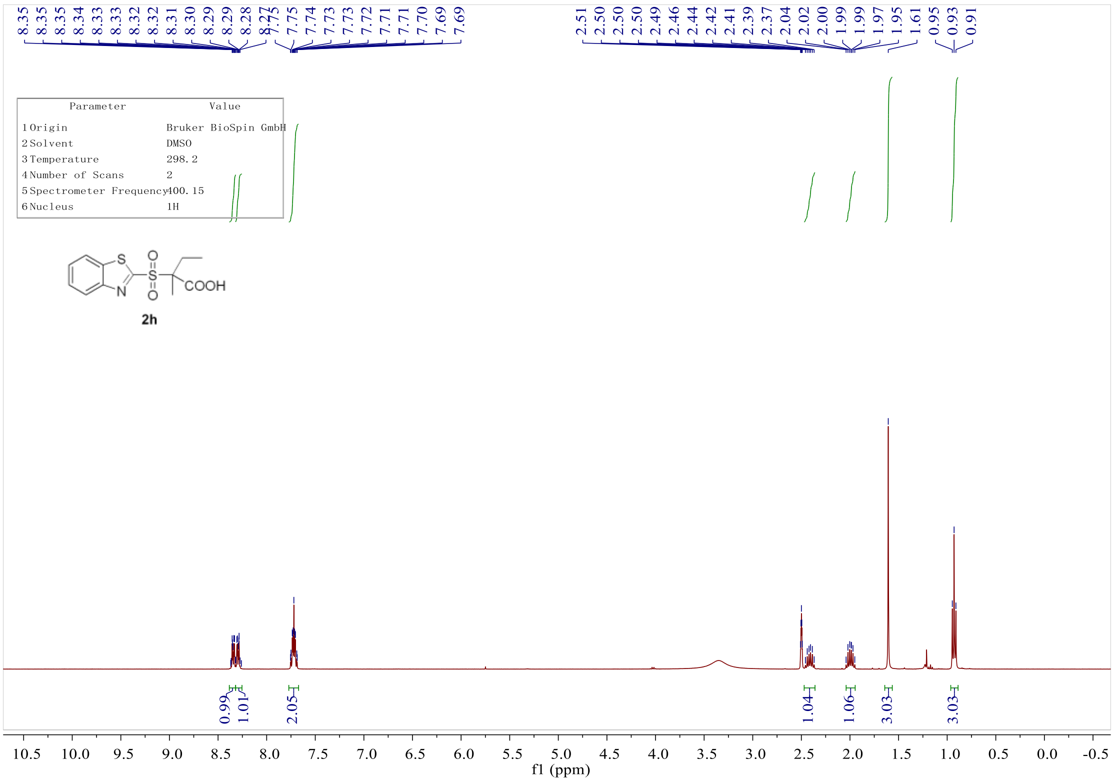

Supplementary Fig. 19 ^{1}H NMR spectra (400 MHz, DMSO, 25 °C) of **2h**

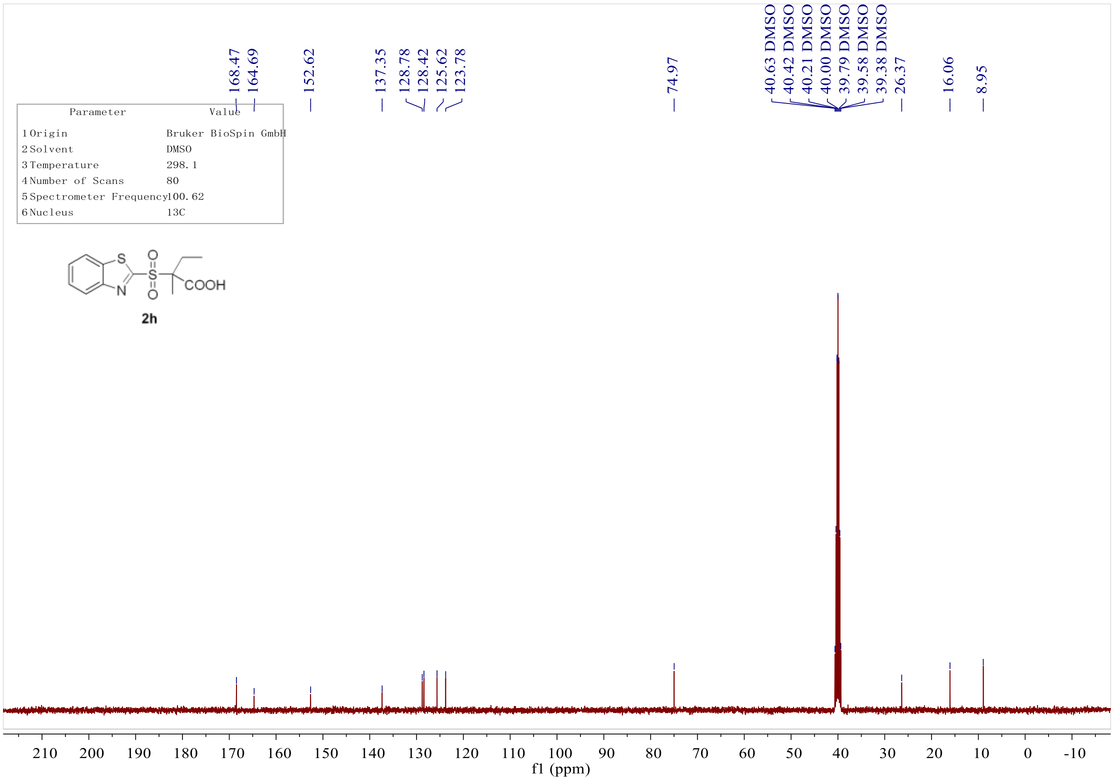

Supplementary Fig. 20 ^{13}C NMR spectra (100 MHz, DMSO, 25 °C) of **2h**

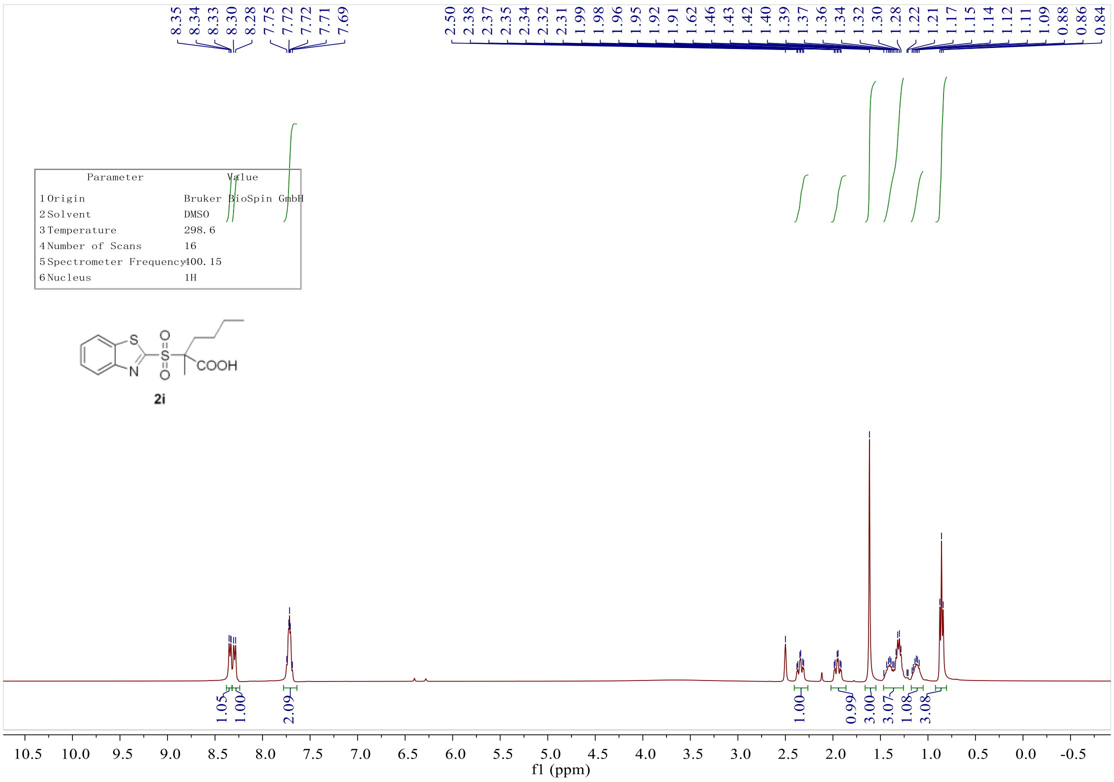

Supplementary Fig. 21 ^{1}H NMR spectra (400 MHz, DMSO, 25 °C) of **2i**

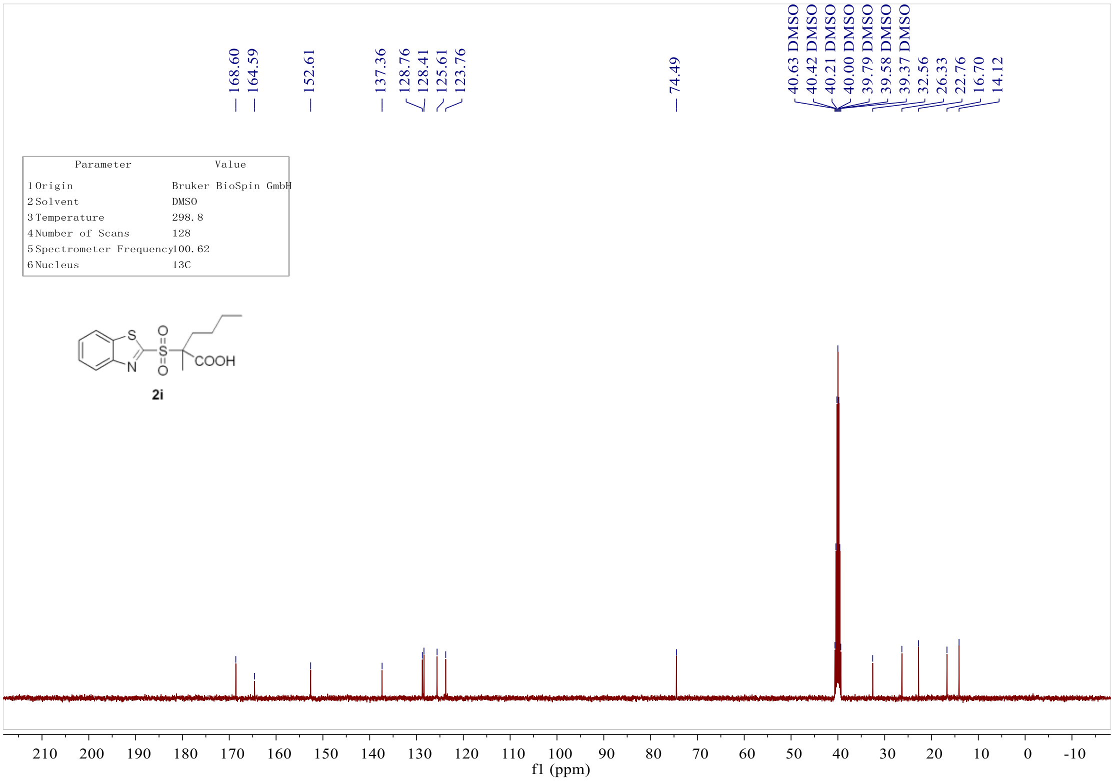

Supplementary Fig. 22 ^{13}C NMR spectra (100 MHz, DMSO, 25 ºC) of **2i**

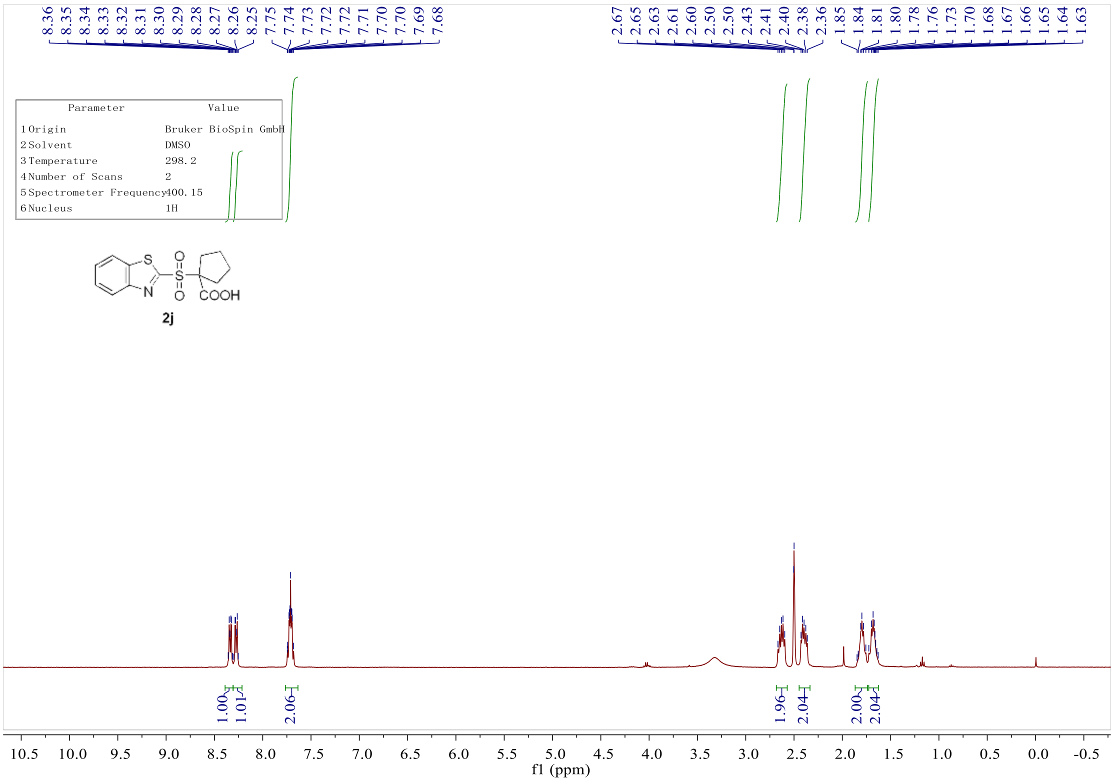

Supplementary Fig. 23 ^{1}H NMR spectra (400 MHz, DMSO, 25 ºC) of **2j**

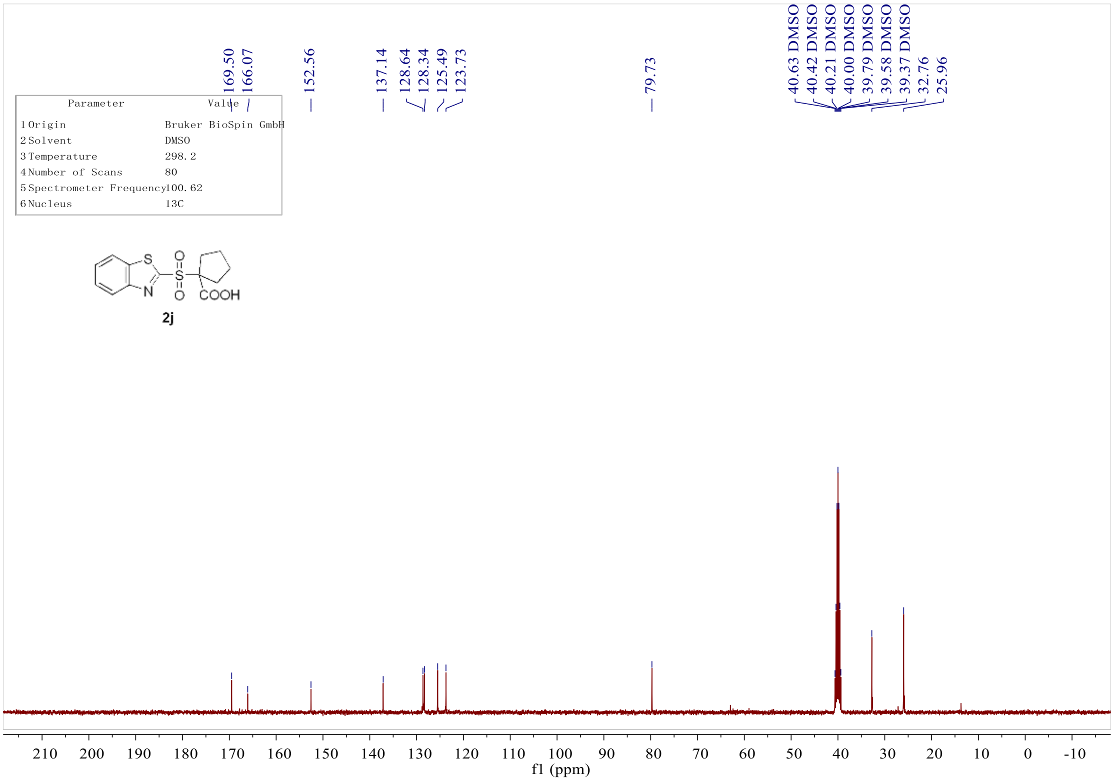

Supplementary Fig. 24 ^{13}C NMR spectra (100 MHz, DMSO, 25 °C) of **2j**

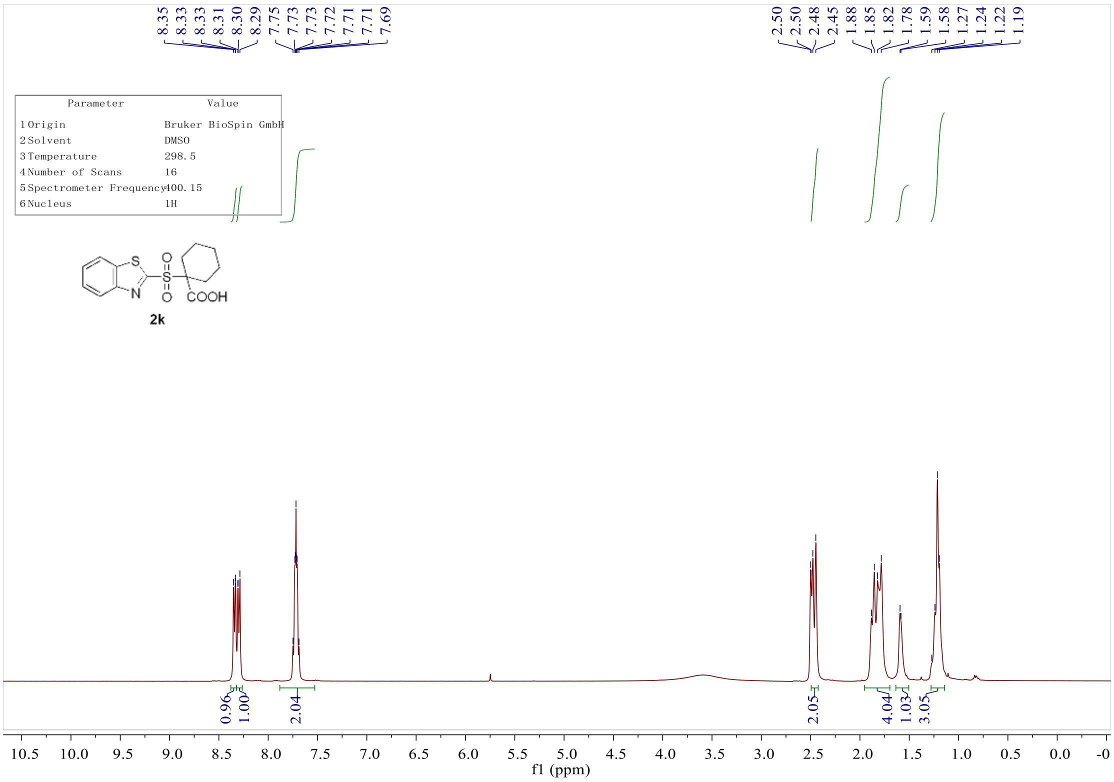

Supplementary Fig. 25 ^{1}H NMR spectra (400 MHz, DMSO, 25 °C) of **2k**

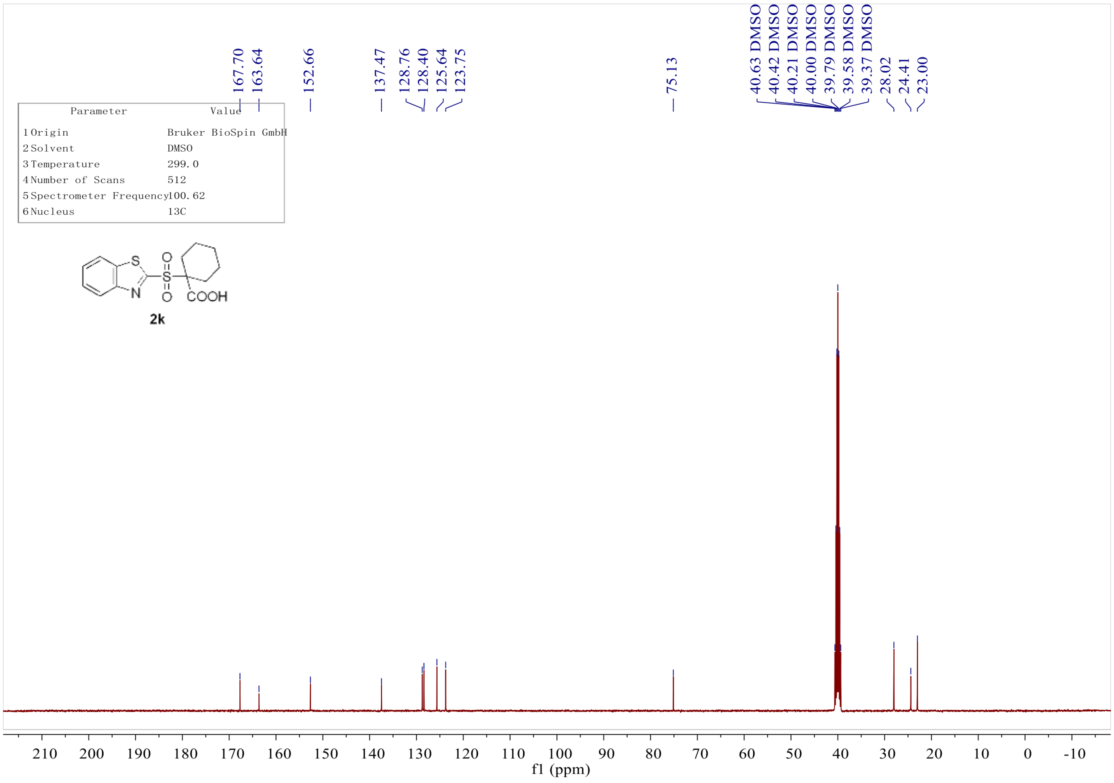

Supplementary Fig. 26 ^{13}C NMR spectra (100 MHz, DMSO, 25 °C) of **2k**

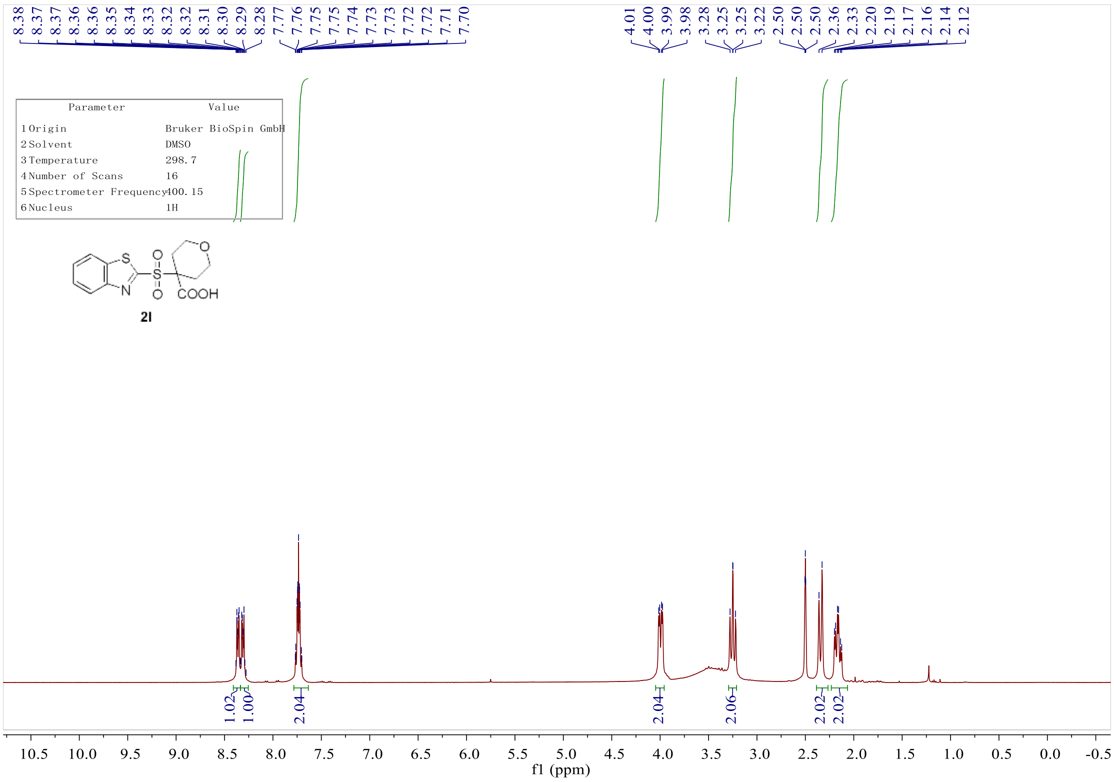

Supplementary Fig. 27 ^{1}H NMR spectra (400 MHz, DMSO, 25 °C) of **2l**

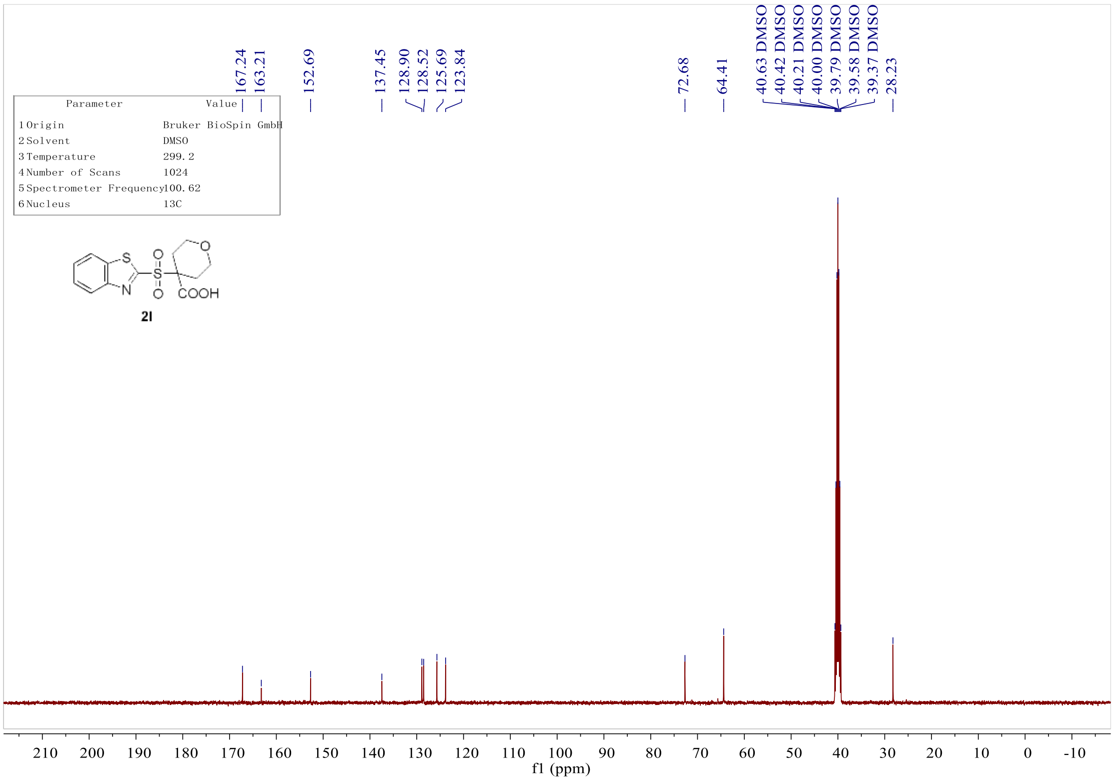

Supplementary Fig. 28 ^{13}C NMR spectra (100 MHz, DMSO, 25 ºC) of **2l**

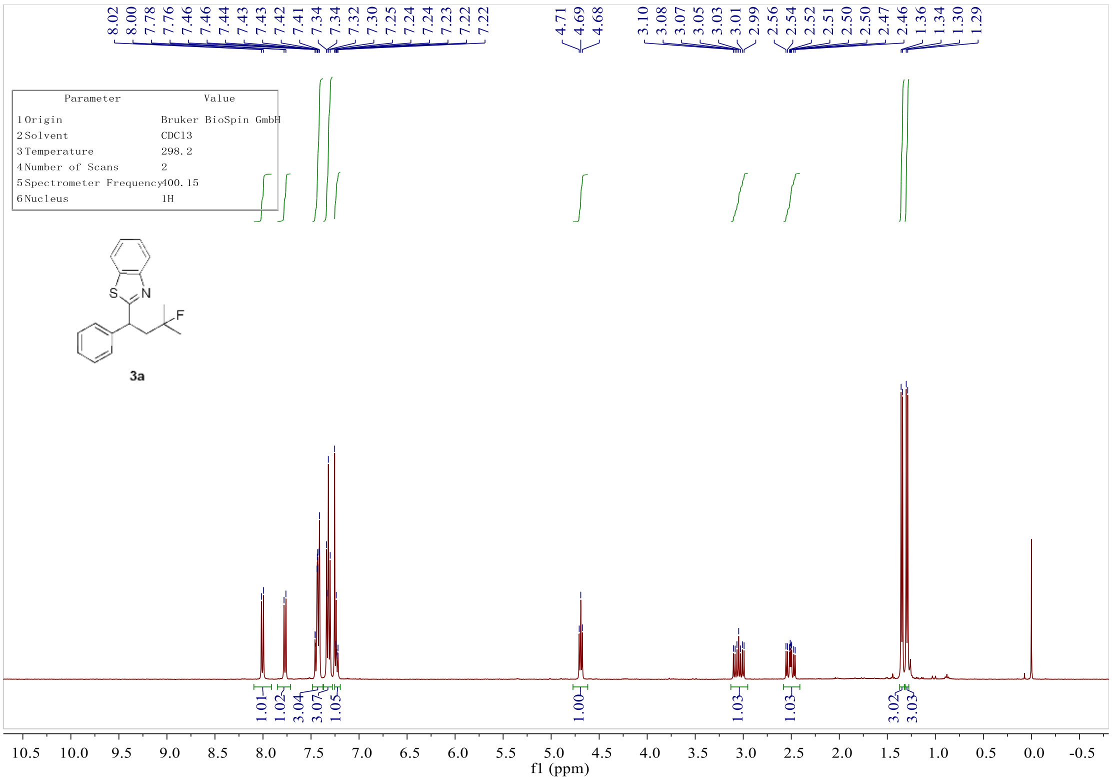

Supplementary Fig. 29 ^{1}H NMR spectra (400 MHz, $CDCl_3$, 25 ºC) of **3a**

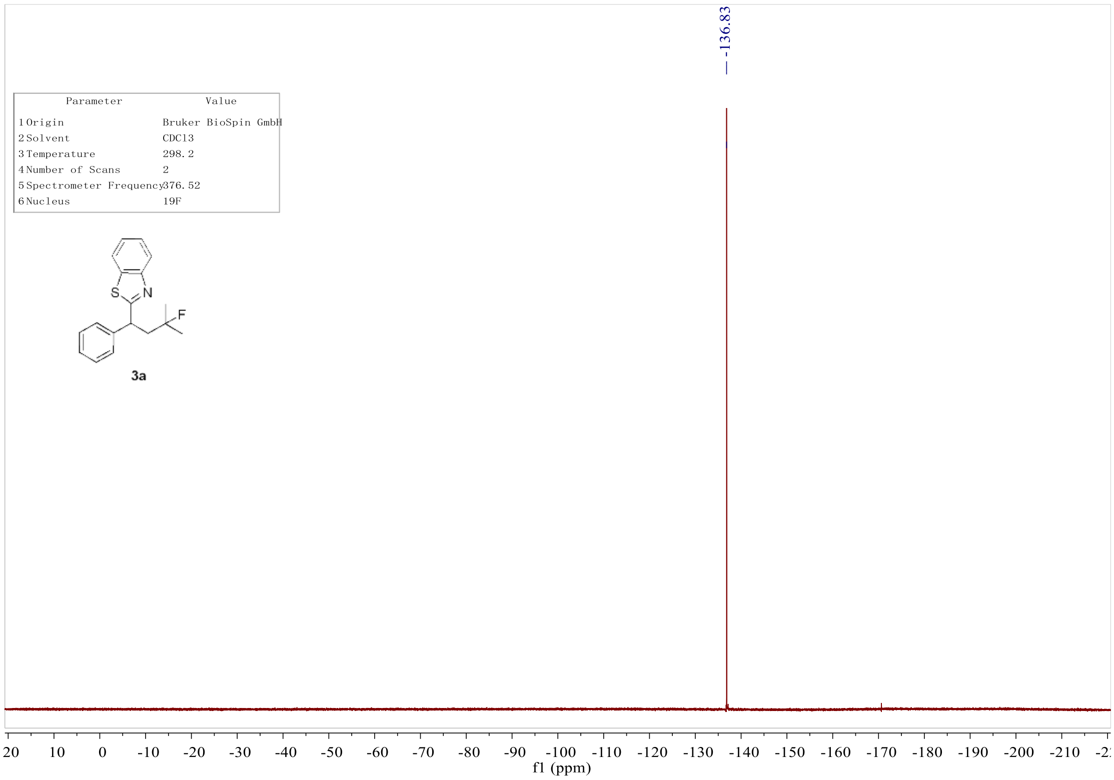

Supplementary Fig. 30 ^{19}F NMR spectra (376 MHz, $CDCl_3$, 25 °C) of **3a**

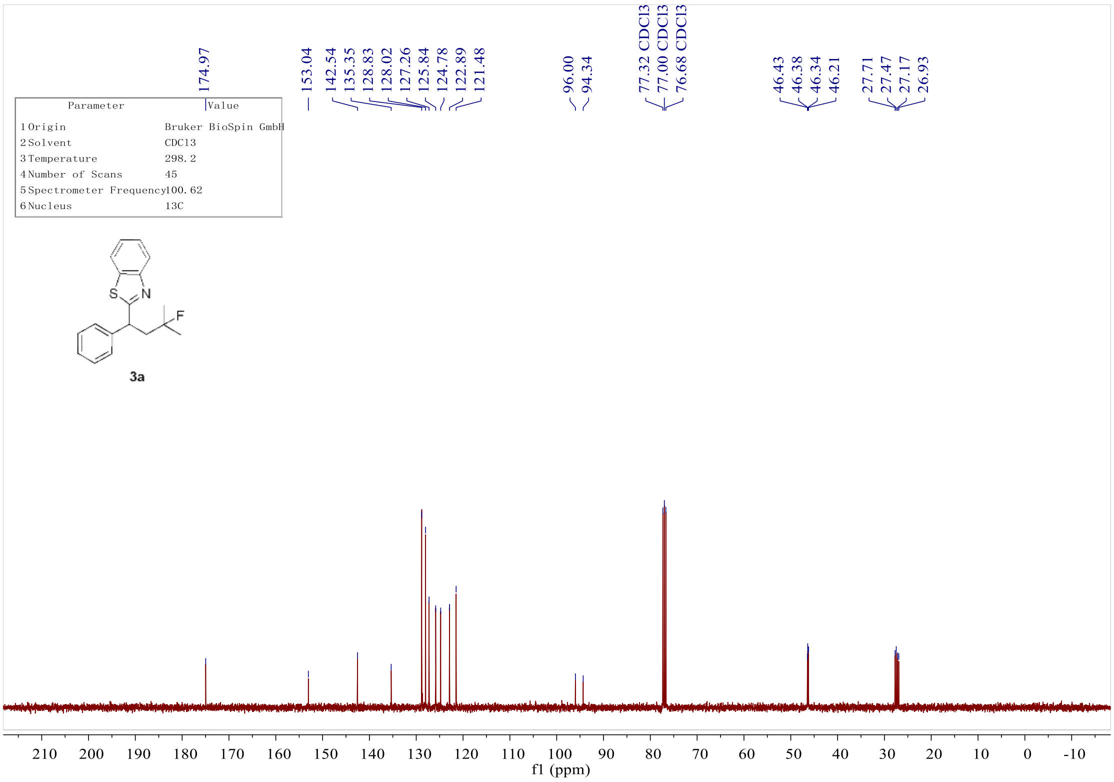

Supplementary Fig. 31 ^{13}C NMR spectra (100 MHz, $CDCl_3$, 25 °C) of **3a**

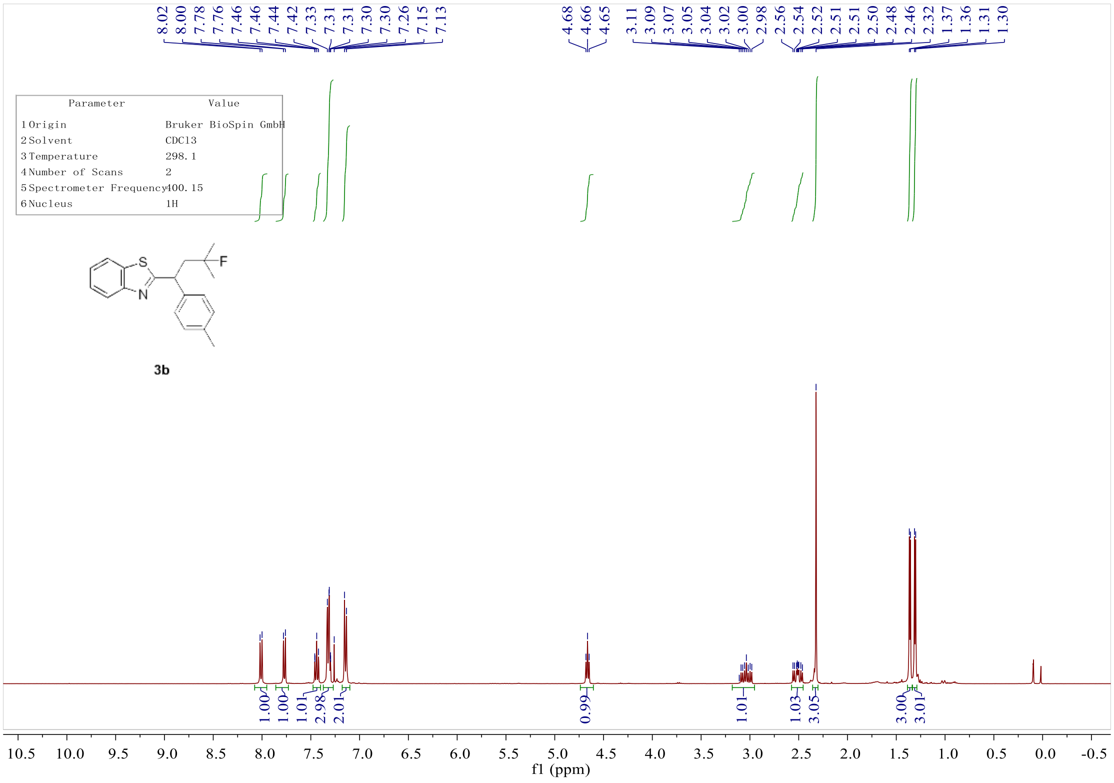

Supplementary Fig. 32 ^{1}H NMR spectra (400 MHz, $CDCl_3$, 25 °C) of **3b**

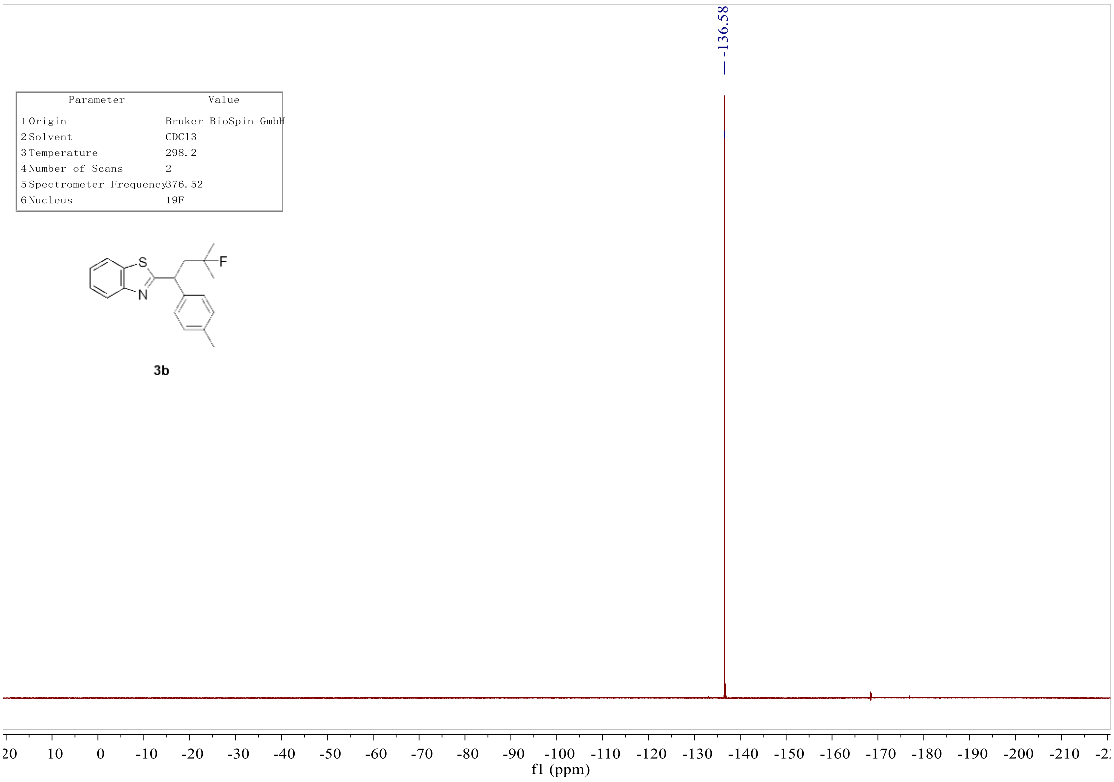

Supplementary Fig. 33 ^{19}F NMR spectra (376 MHz, $CDCl_3$, 25 °C) of **3b**

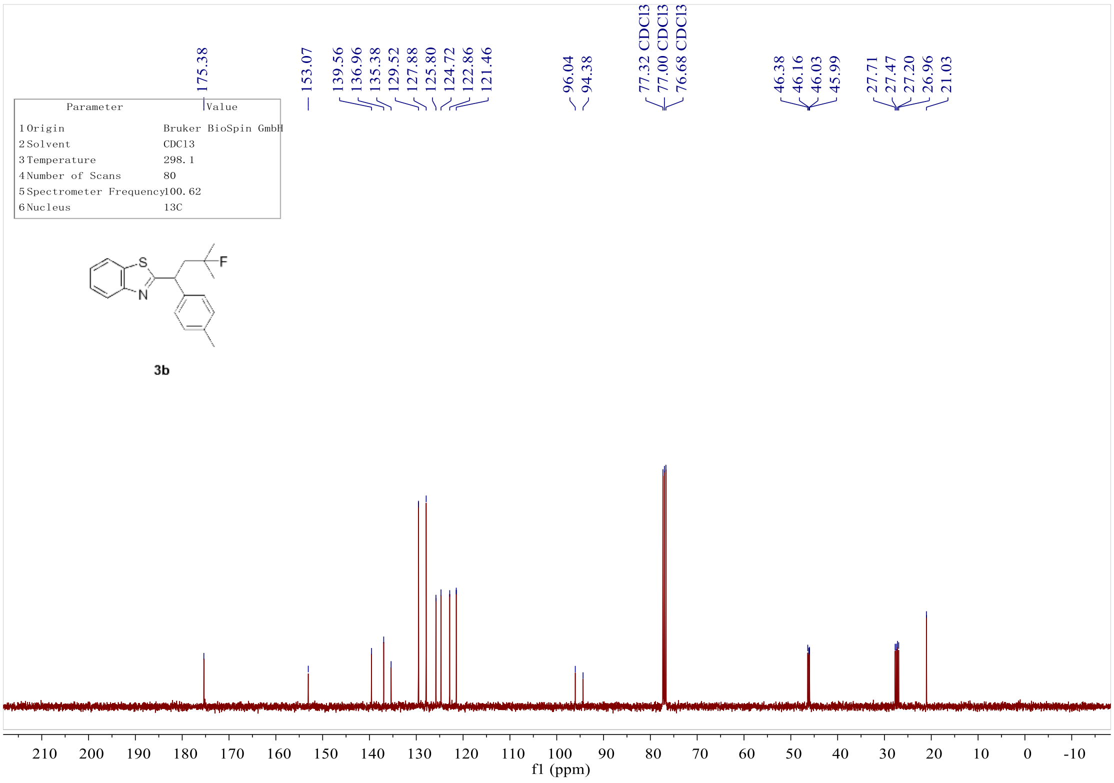

Supplementary Fig. 34 ^{13}C NMR spectra (100 MHz, $CDCl_3$, 25 °C) of **3b**

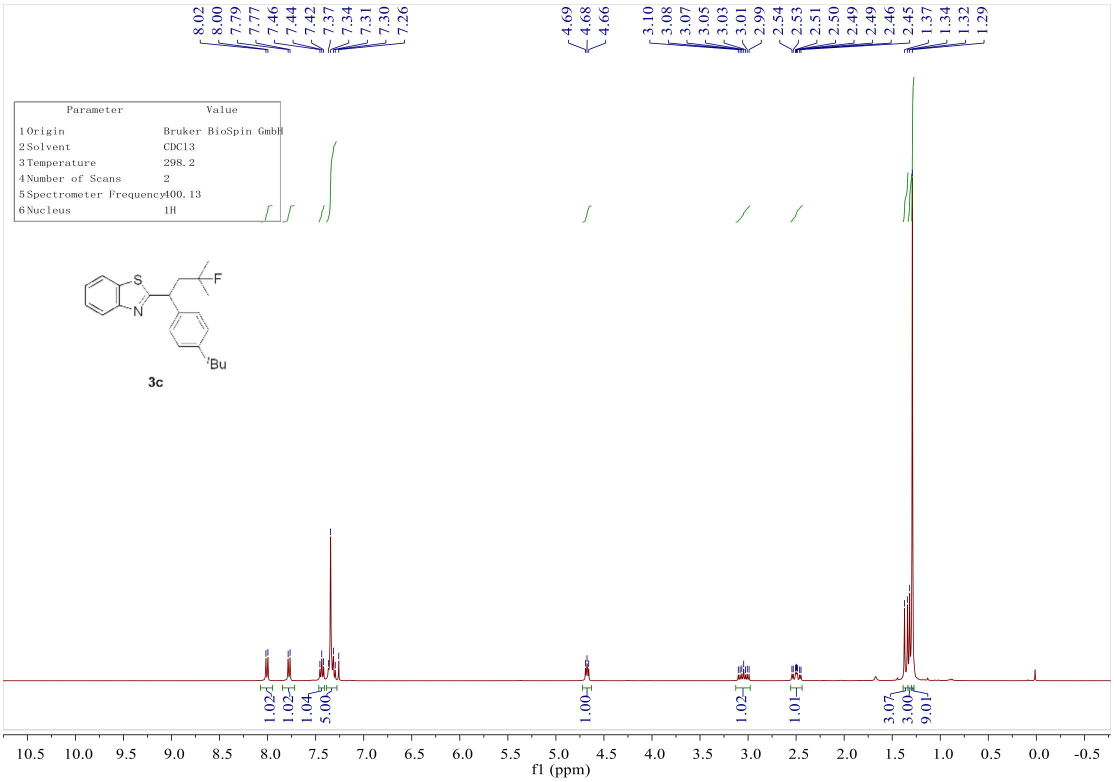

Supplementary Fig. 35 ^{1}H NMR spectra (400 MHz, $CDCl_3$, 25 °C) of **3c**

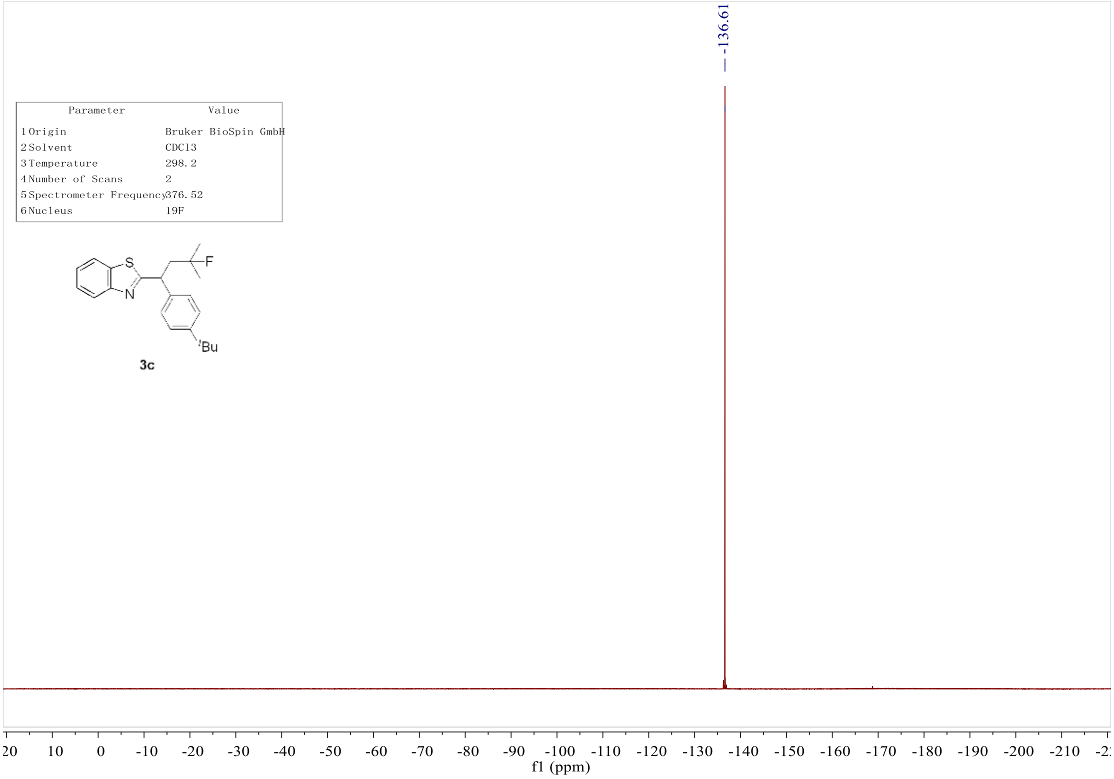

Supplementary Fig. 36 ^{19}F NMR spectra (376 MHz, $CDCl_3$, 25 °C) of **3c**

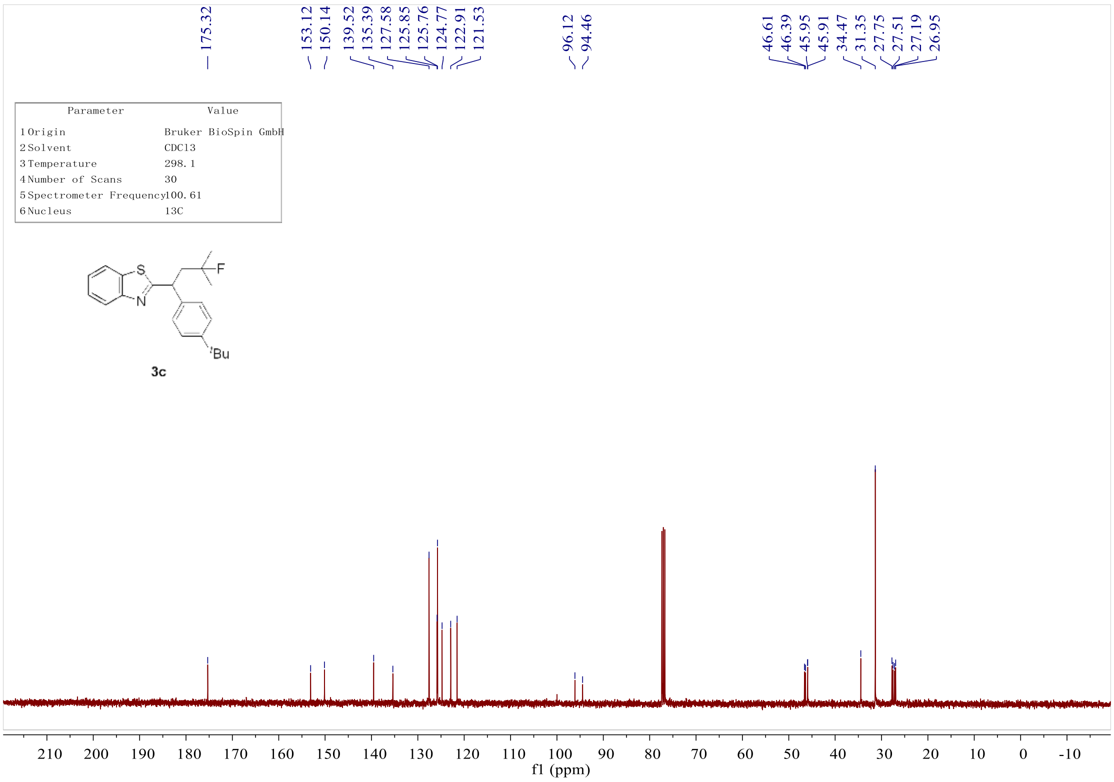

Supplementary Fig. 37 ^{13}C NMR spectra (100 MHz, $CDCl_3$, 25 °C) of **3c**

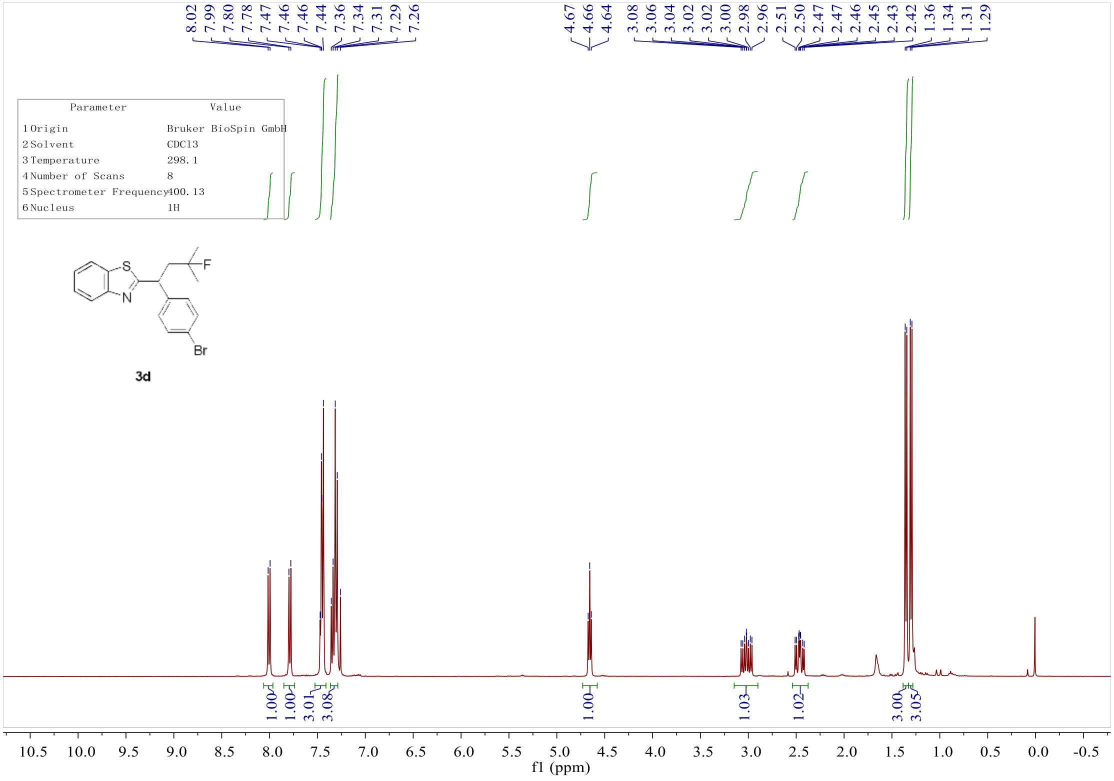

Supplementary Fig. 38 ^{1}H NMR spectra (400 MHz, $CDCl_3$, 25 °C) of **3d**

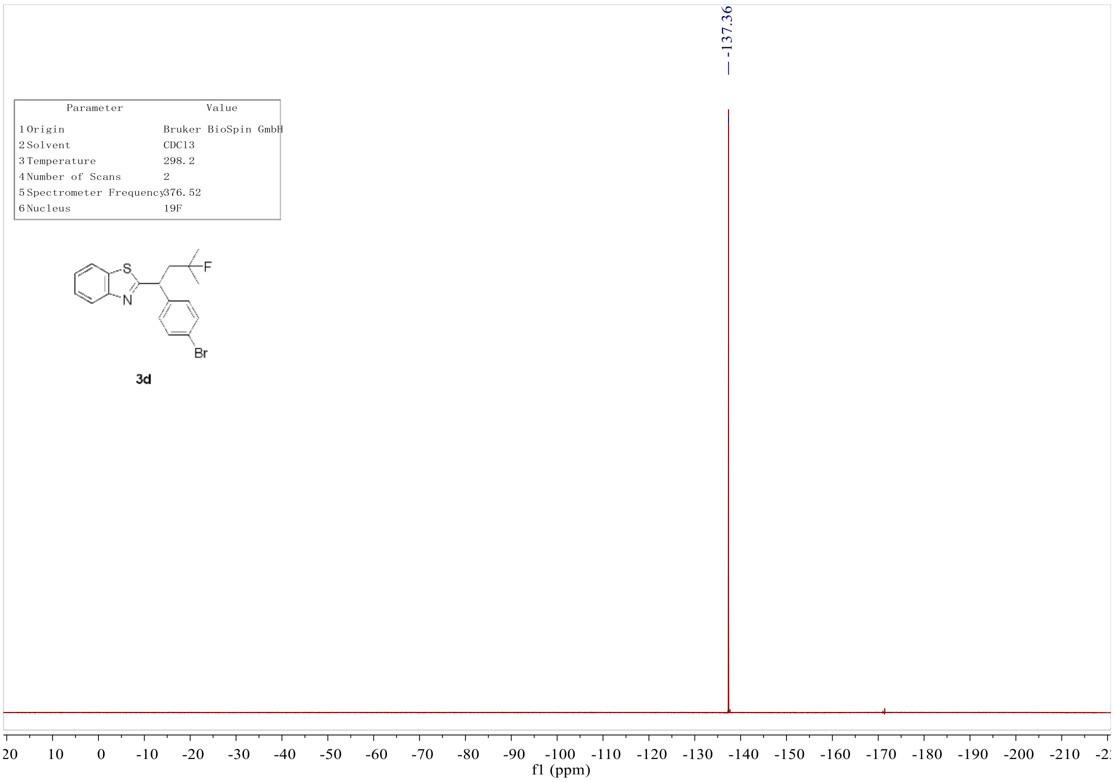

Supplementary Fig. 39 ^{19}F NMR spectra (376 MHz, $CDCl_3$, 25 °C) of **3d**

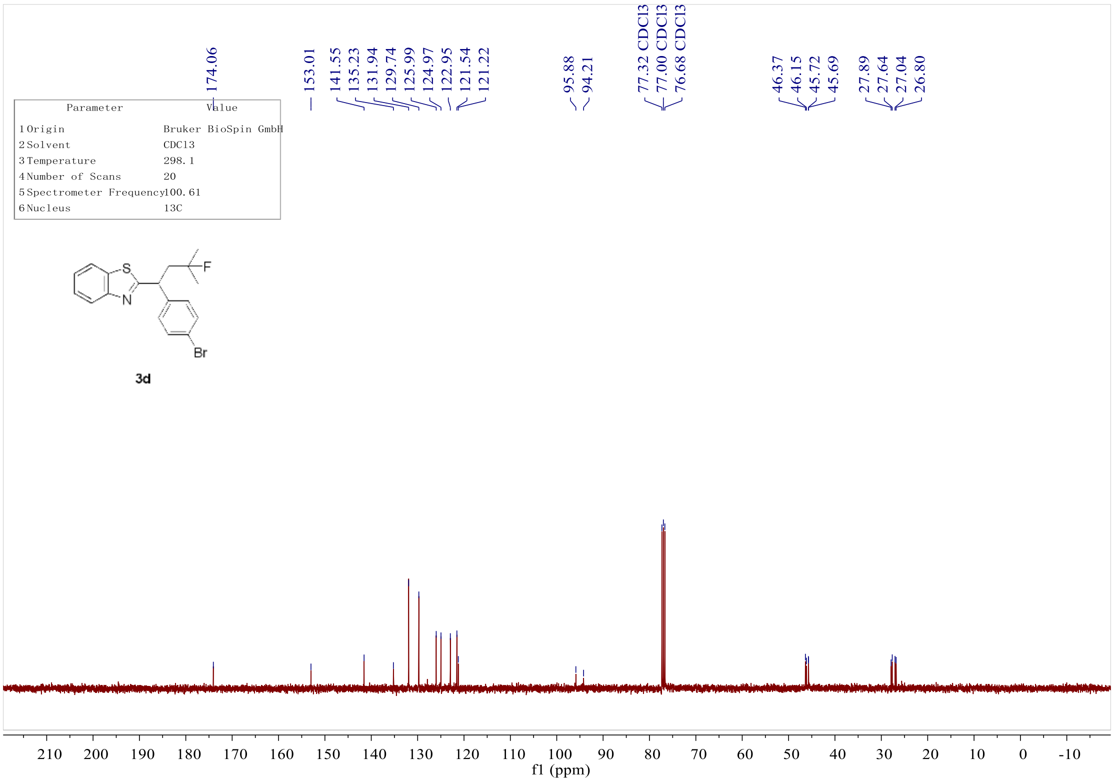

Supplementary Fig. 40 ^{13}C NMR spectra (100 MHz, $CDCl_3$, 25 °C) of **3d**

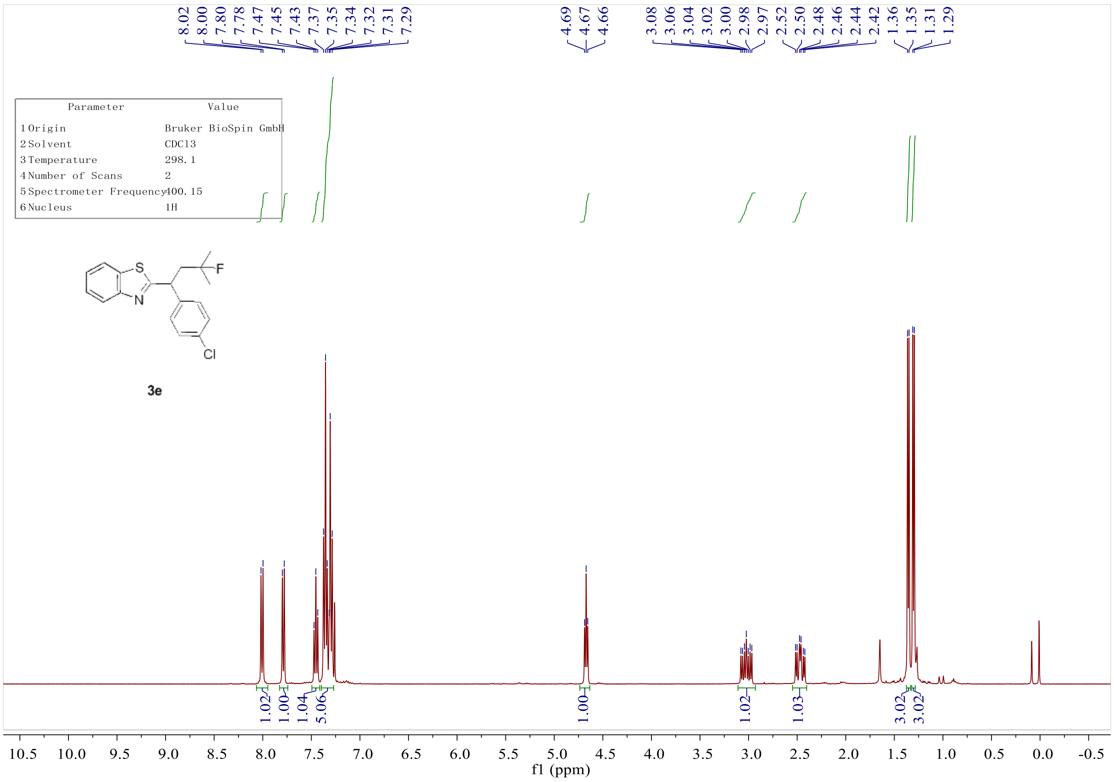

Supplementary Fig. 41 ^{1}H NMR spectra (400 MHz, $CDCl_3$, 25 °C) of **3e**

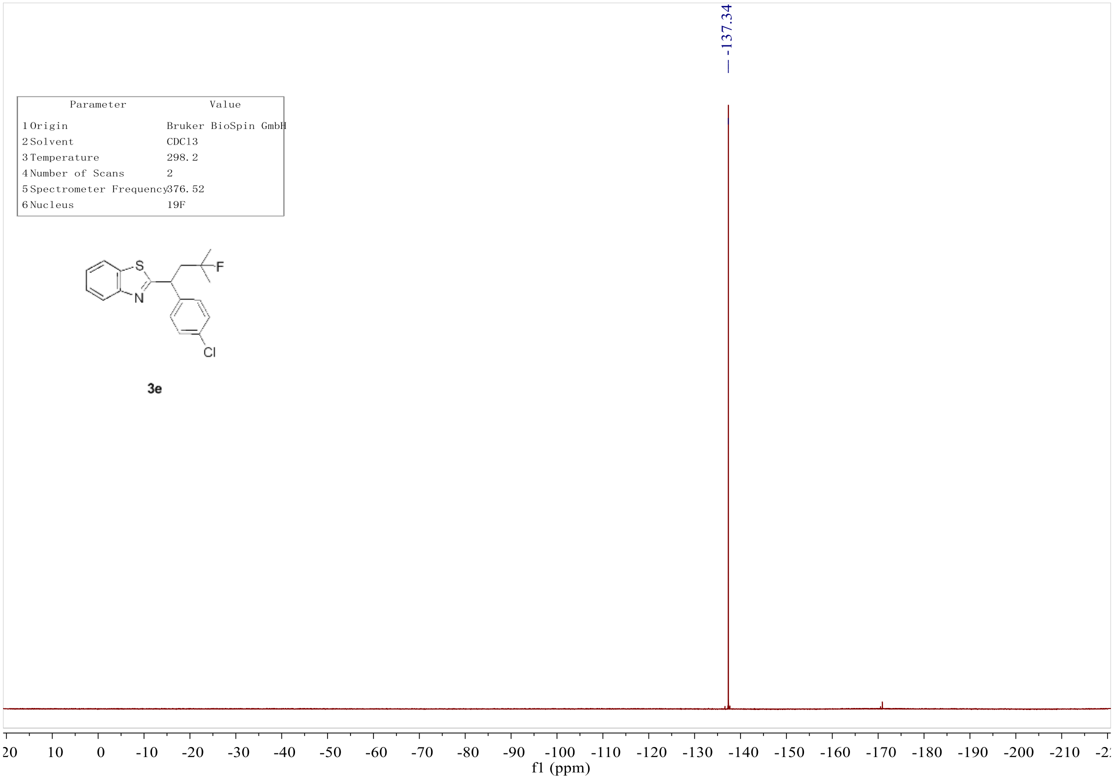

Supplementary Fig. 42 ^{19}F NMR spectra (376 MHz, $CDCl_3$, 25 °C) of **3e**

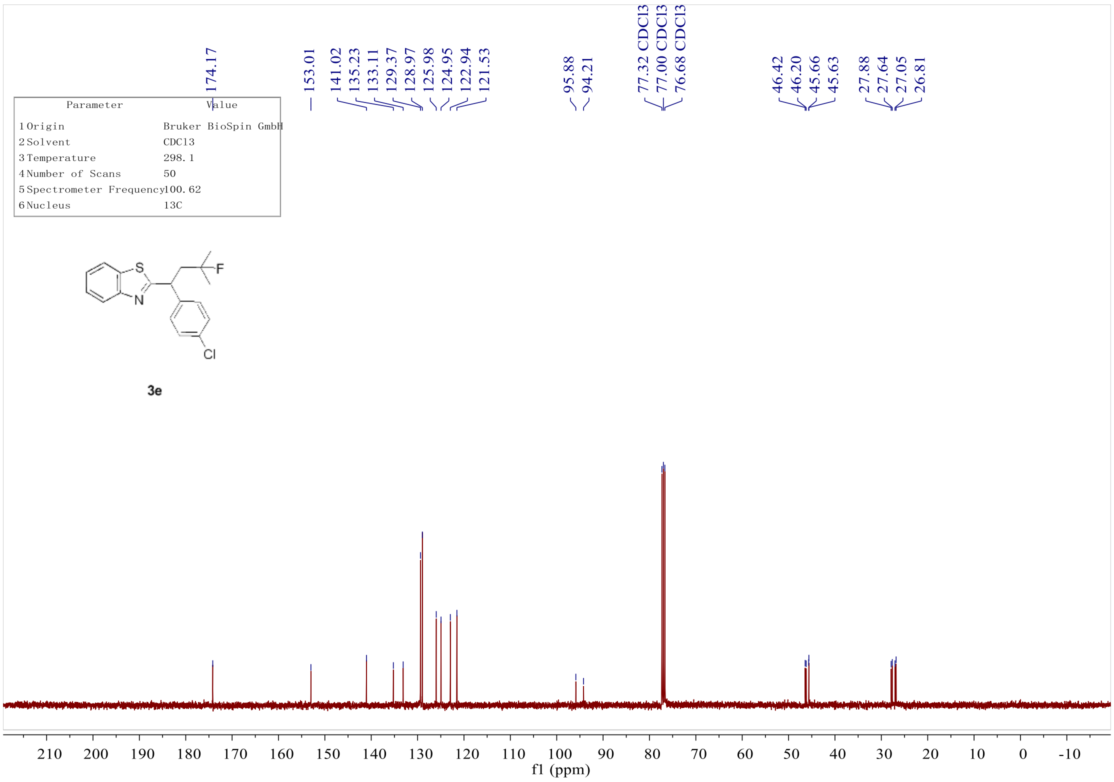

Supplementary Fig. 43 ^{13}C NMR spectra (100 MHz, $CDCl_3$, 25 °C) of **3e**

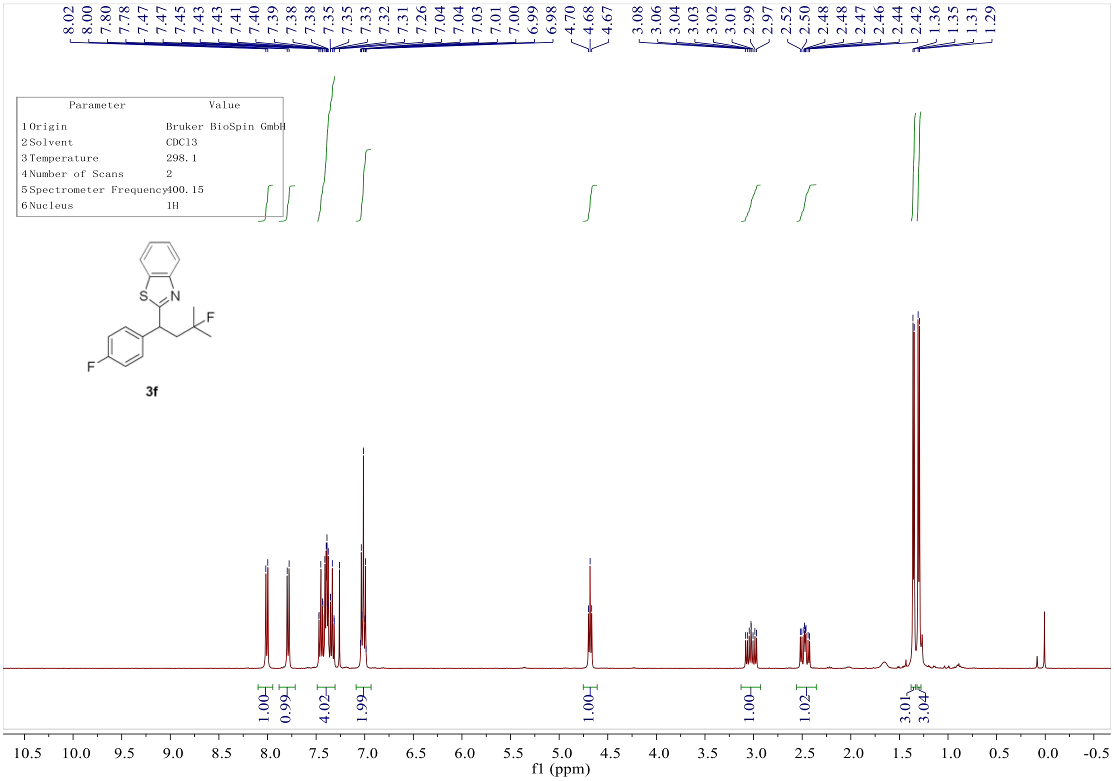

Supplementary Fig. 44 ^{1}H NMR spectra (400 MHz, $CDCl_3$, 25 °C) of **3f**

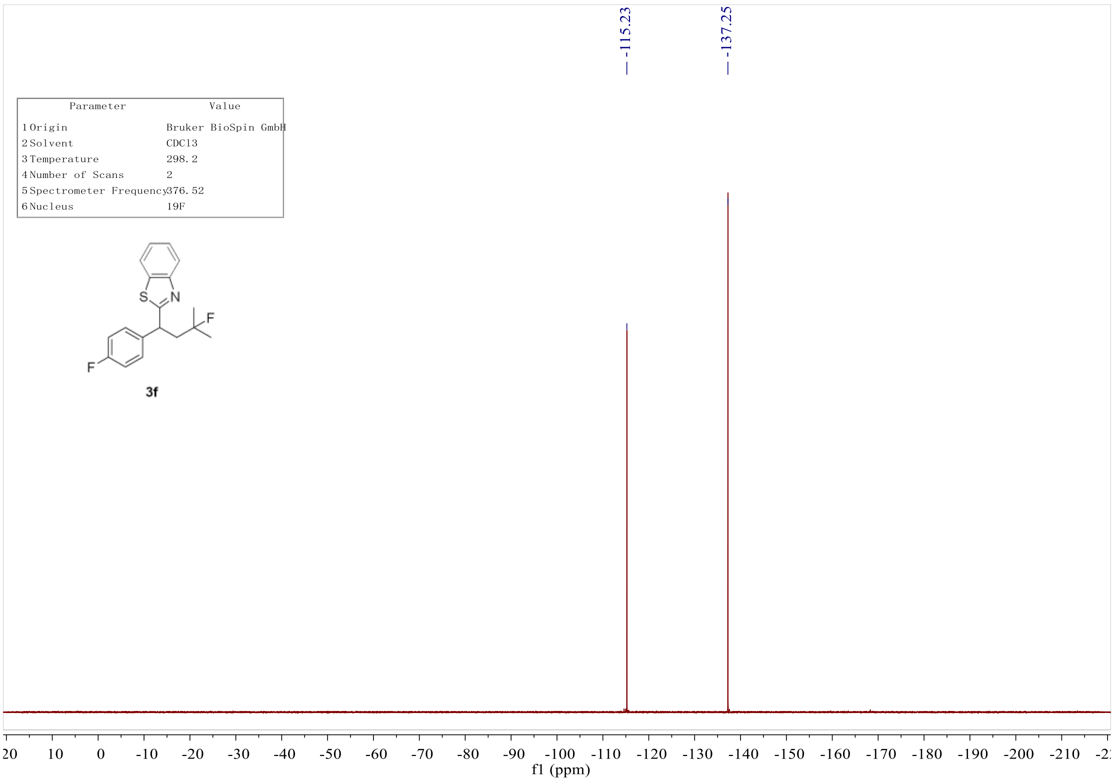

Supplementary Fig. 45 ^{19}F NMR spectra (376 MHz, $CDCl_3$, 25 °C) of **3f**

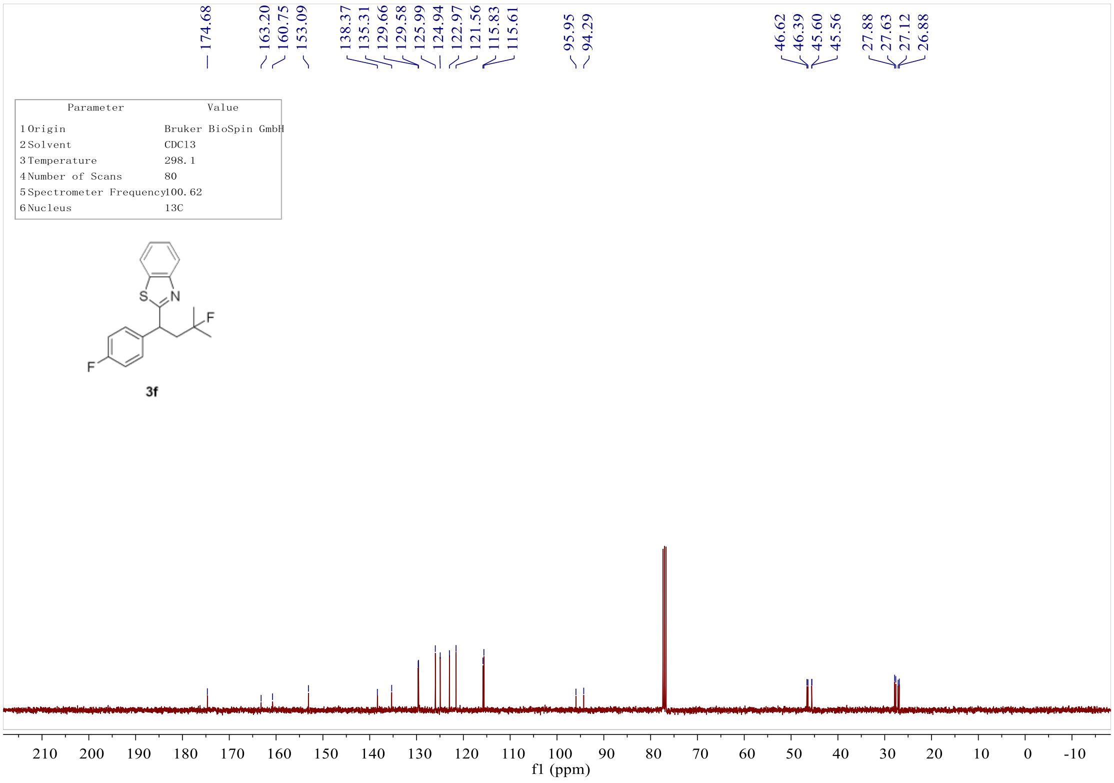

Supplementary Fig. 46 ^{13}C NMR spectra (100 MHz, $CDCl_3$, 25 °C) of **3f**

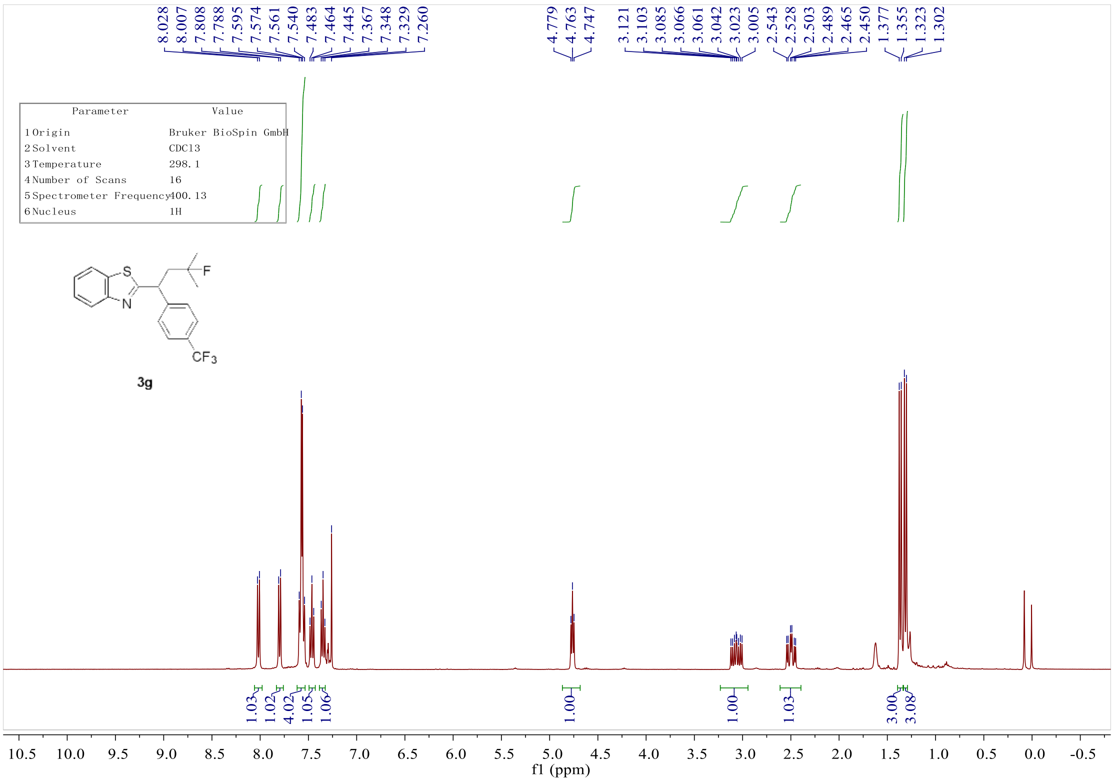

Supplementary Fig. 47 ^{1}H NMR spectra (400 MHz, $CDCl_3$, 25 °C) of **3g**

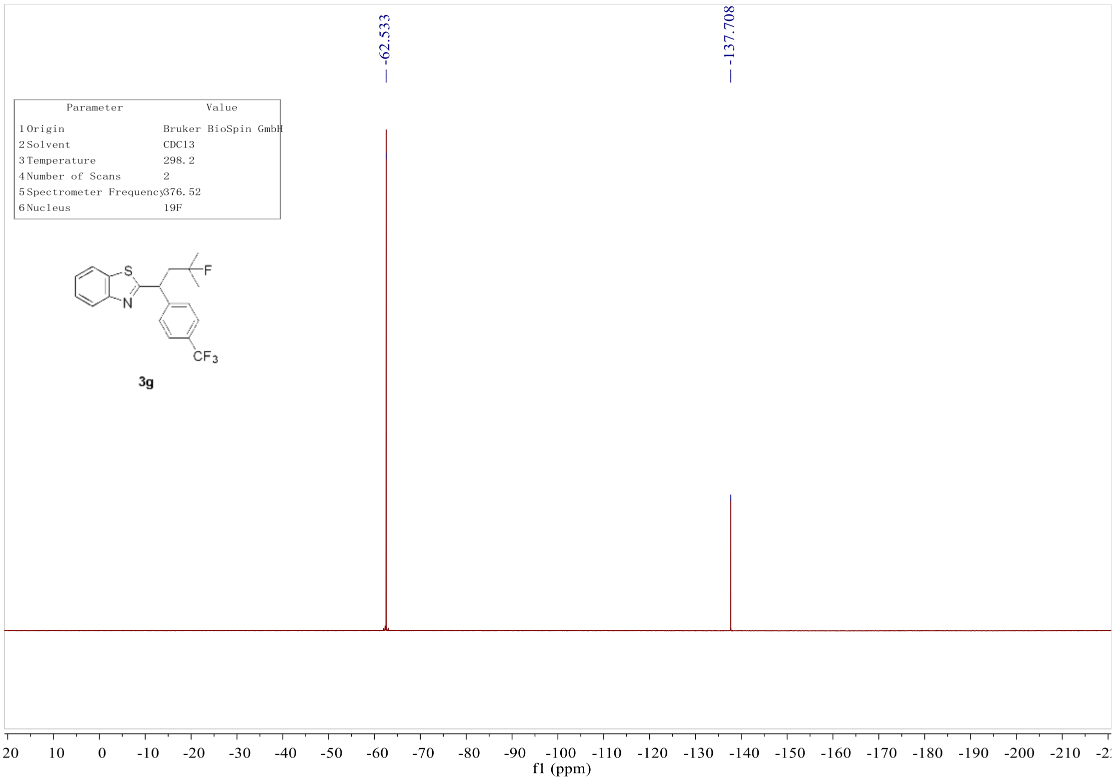

Supplementary Fig. 48 ^{19}F NMR spectra (376 MHz, $CDCl_3$, 25 °C) of **3g**

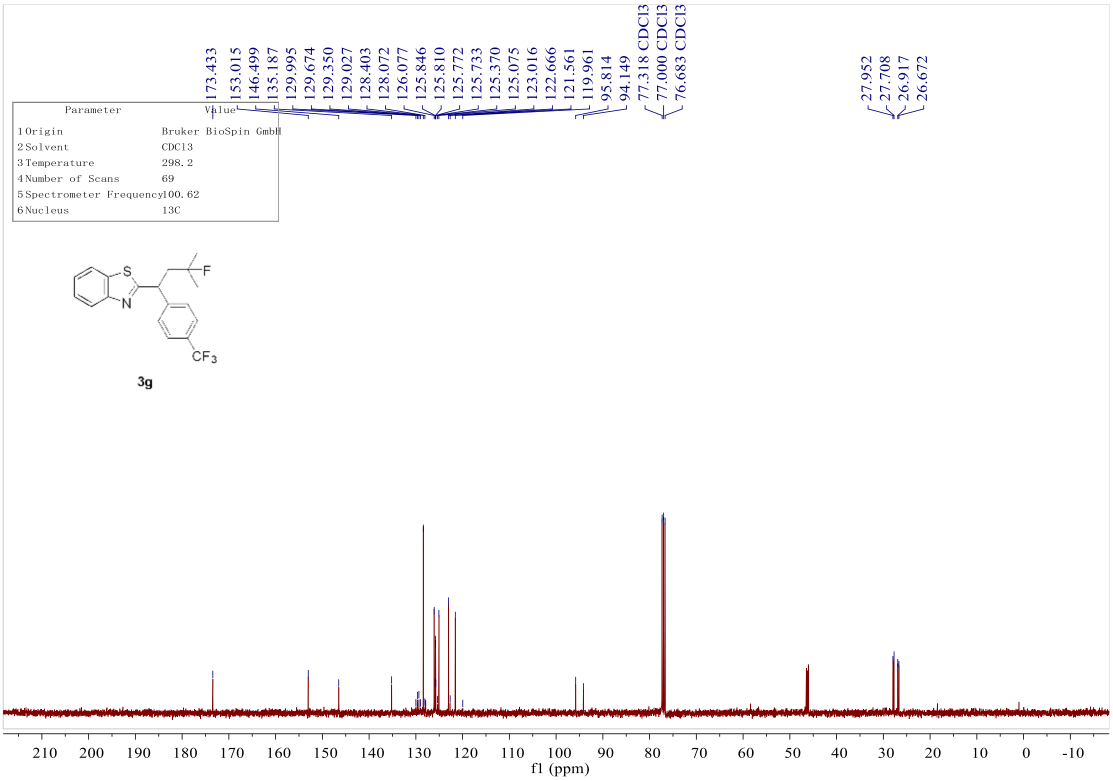

Supplementary Fig. 49 ^{13}C NMR spectra (100 MHz, $CDCl_3$, 25 °C) of **3g**

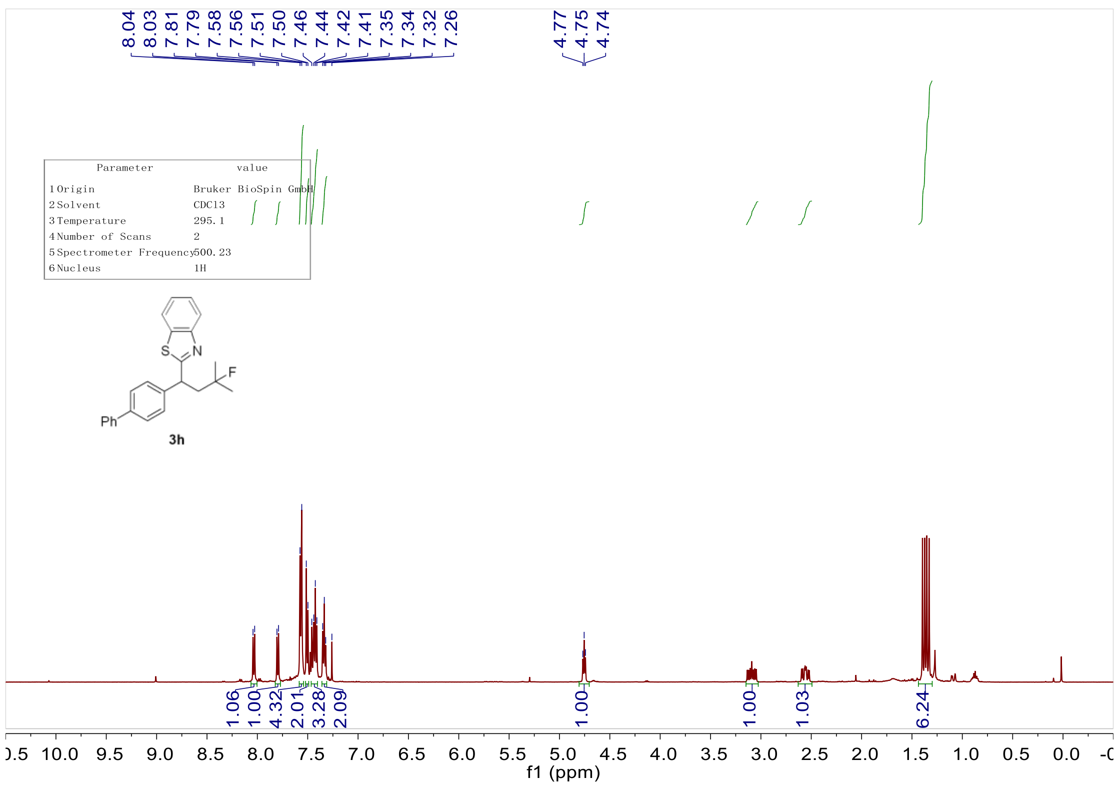

Supplementary Fig. 50 ^{1}H NMR spectra (500 MHz, $CDCl_3$, 25 °C) of **3h**

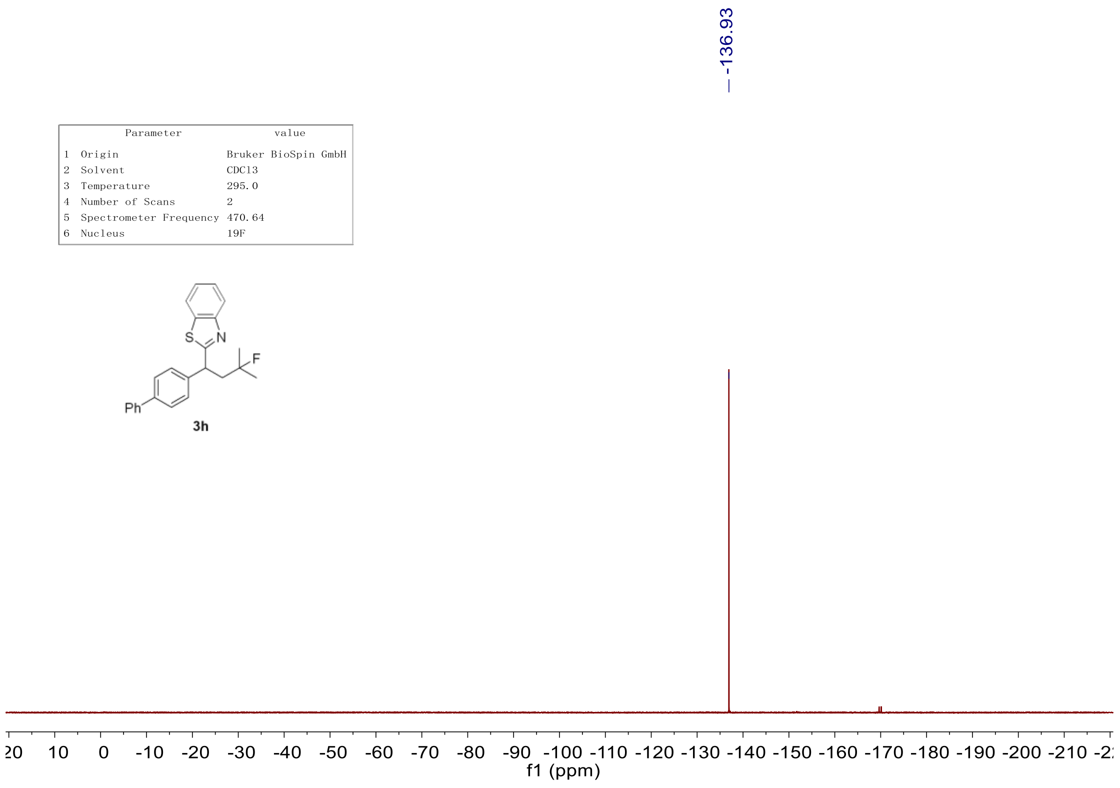

Supplementary Fig. 51 ^{19}F NMR spectra (471 MHz, $CDCl_3$, 25 °C) of **3h**

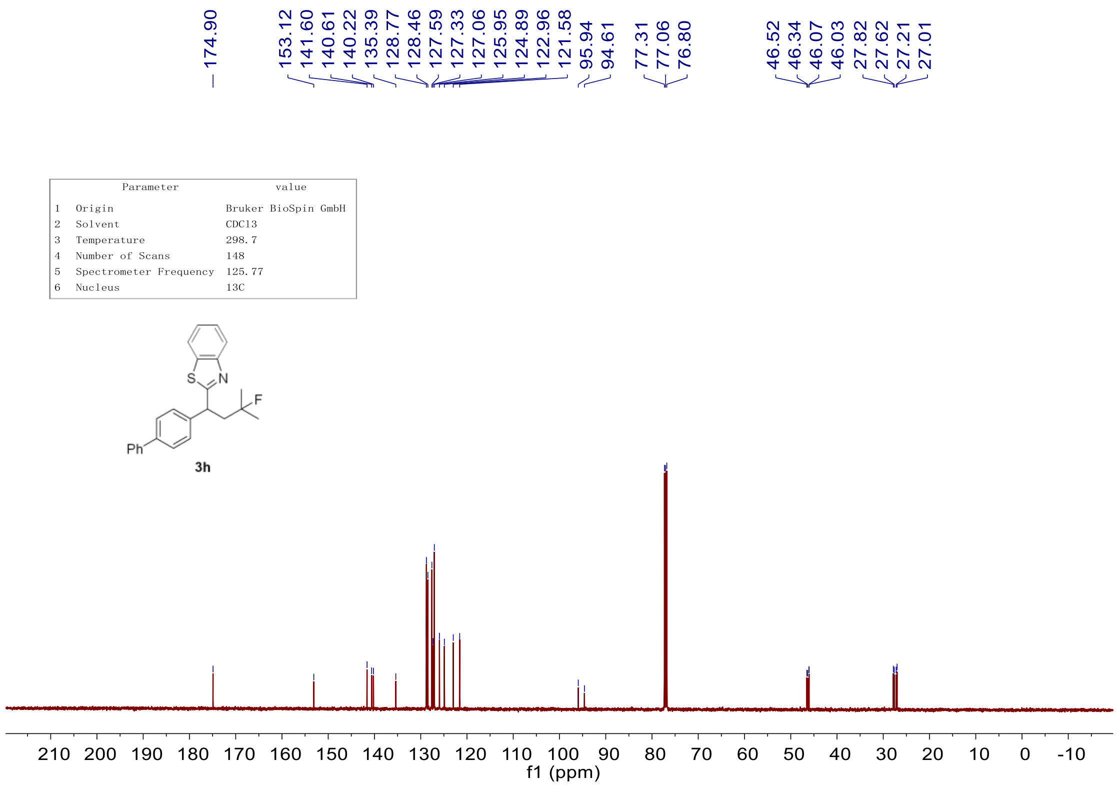

Supplementary Fig. 52 ^{13}C NMR spectra (125 MHz, $CDCl_3$, 25 °C) of **3h**

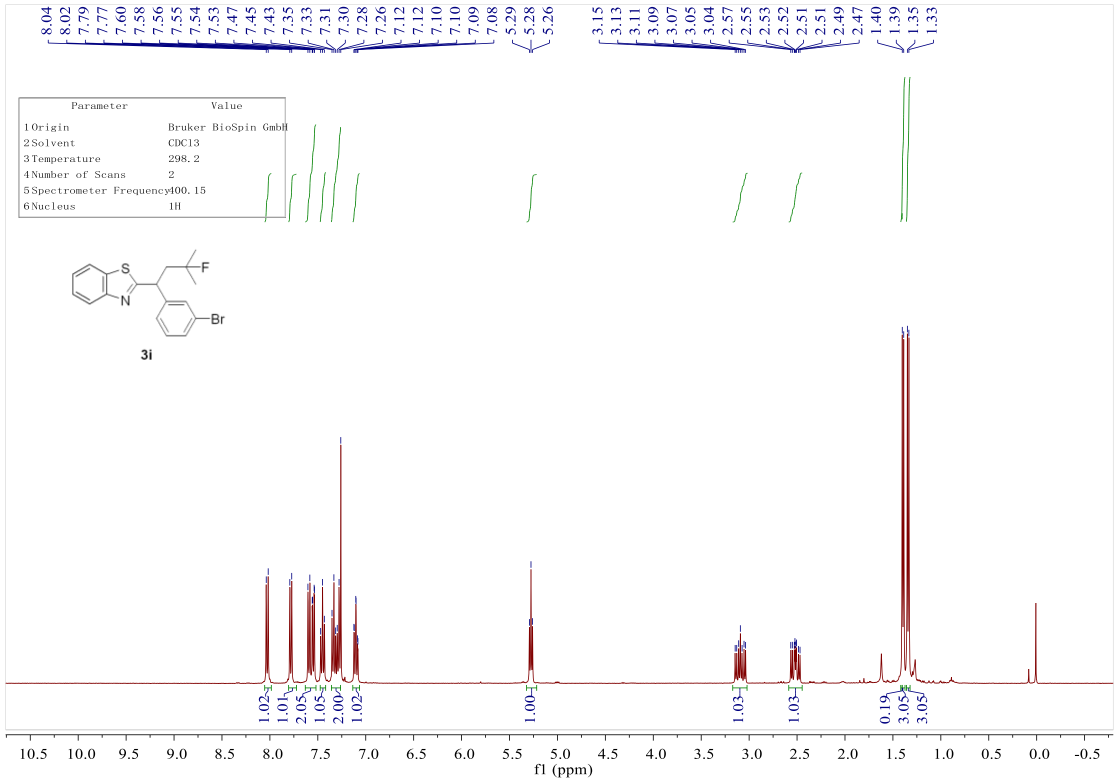

Supplementary Fig. 53 ^{1}H NMR spectra (400 MHz, $CDCl_3$, 25 °C) of **3i**

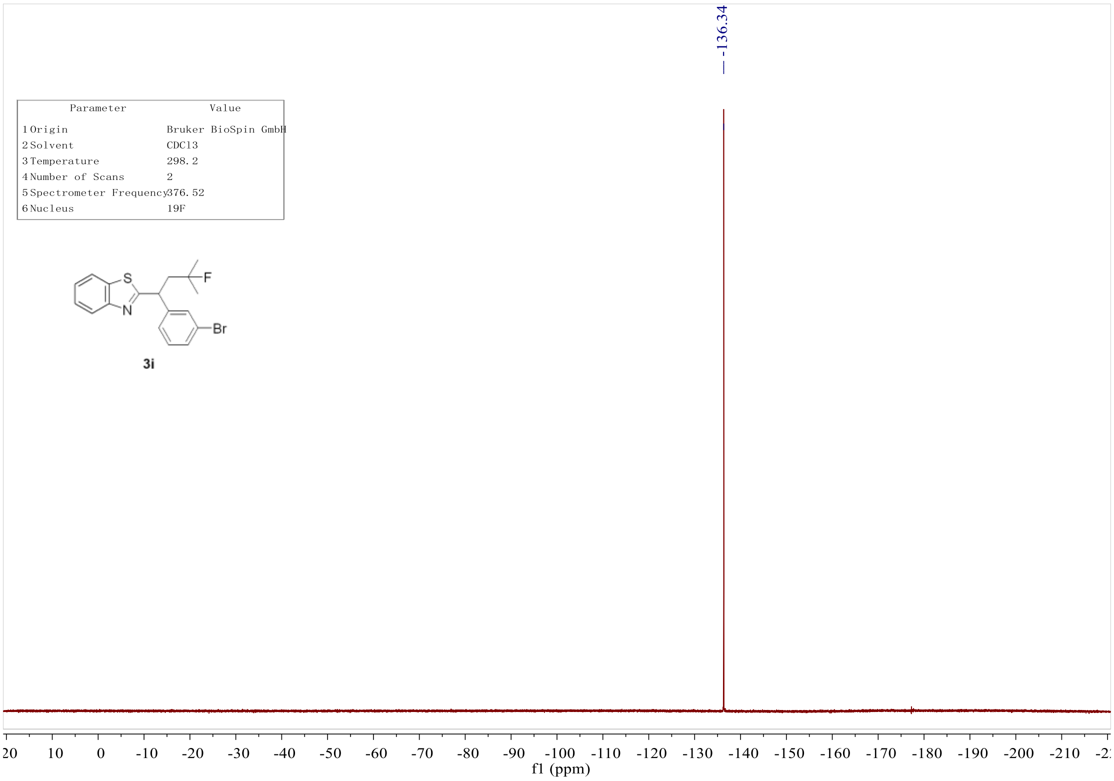

Supplementary Fig. 54 ^{19}F NMR spectra (376 MHz, $CDCl_3$, 25 ºC) of **3i**

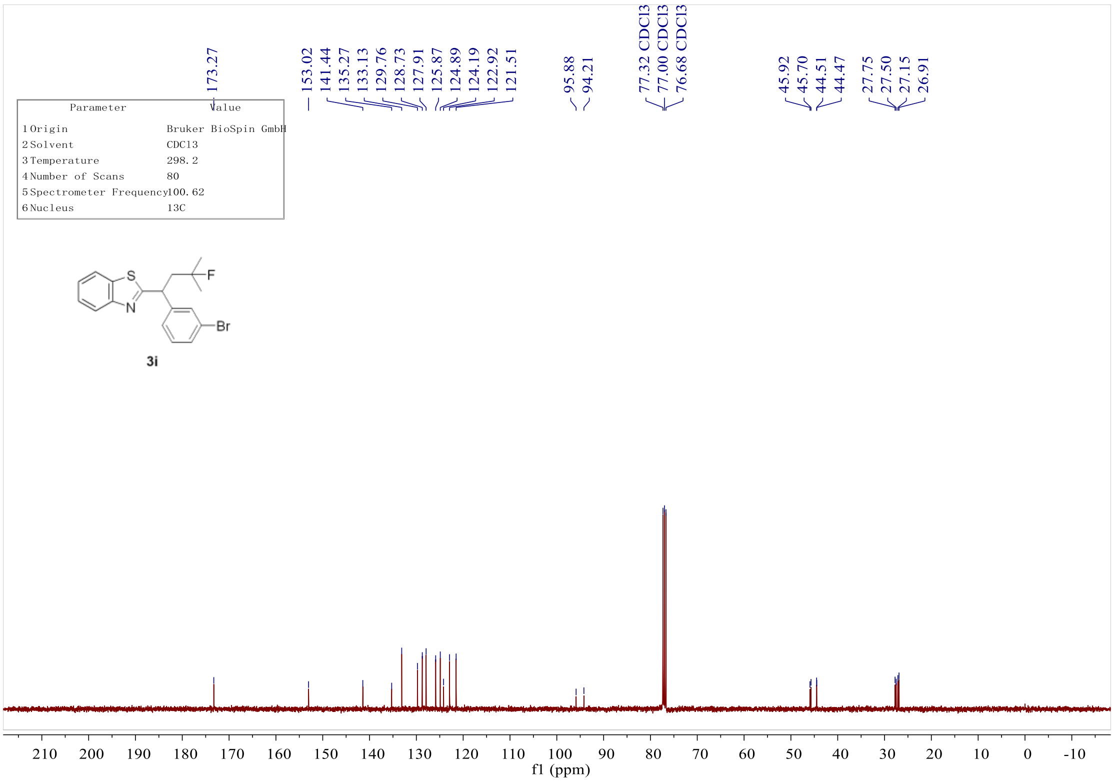

Supplementary Fig. 55 ^{13}C NMR spectra (100 MHz, $CDCl_3$, 25 ºC) of **3i**

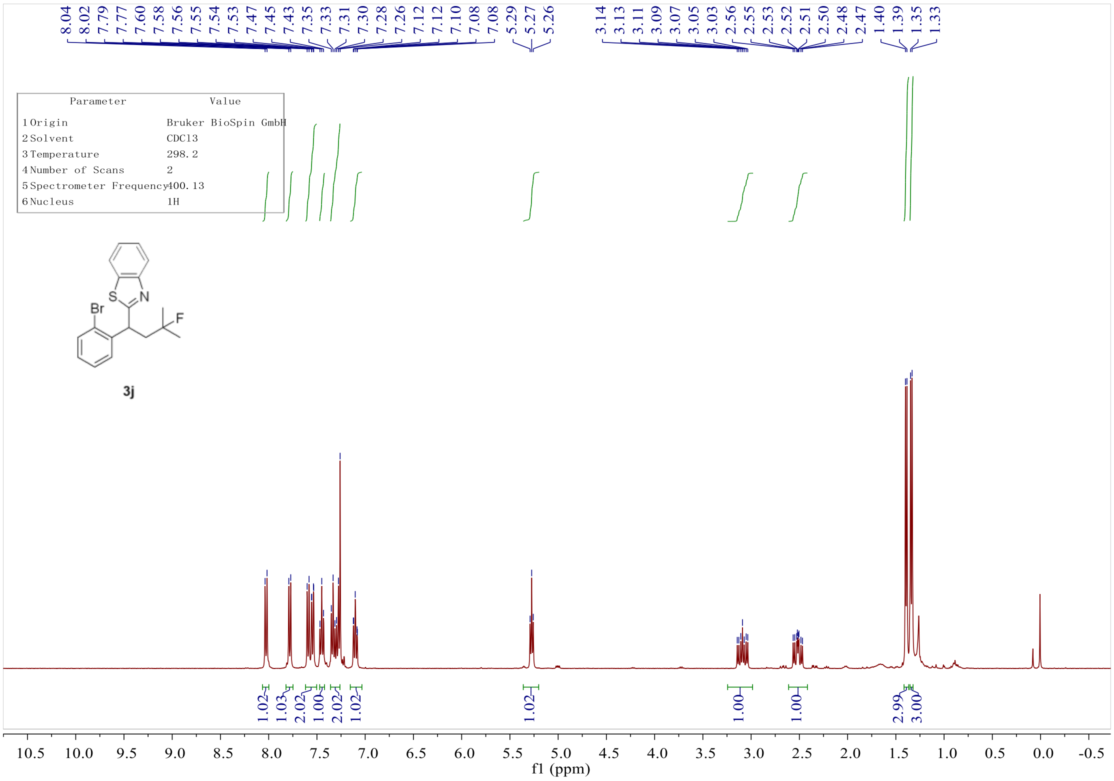

Supplementary Fig. 56 ^{1}H NMR spectra (400 MHz, $CDCl_3$, 25 °C) of **3j**

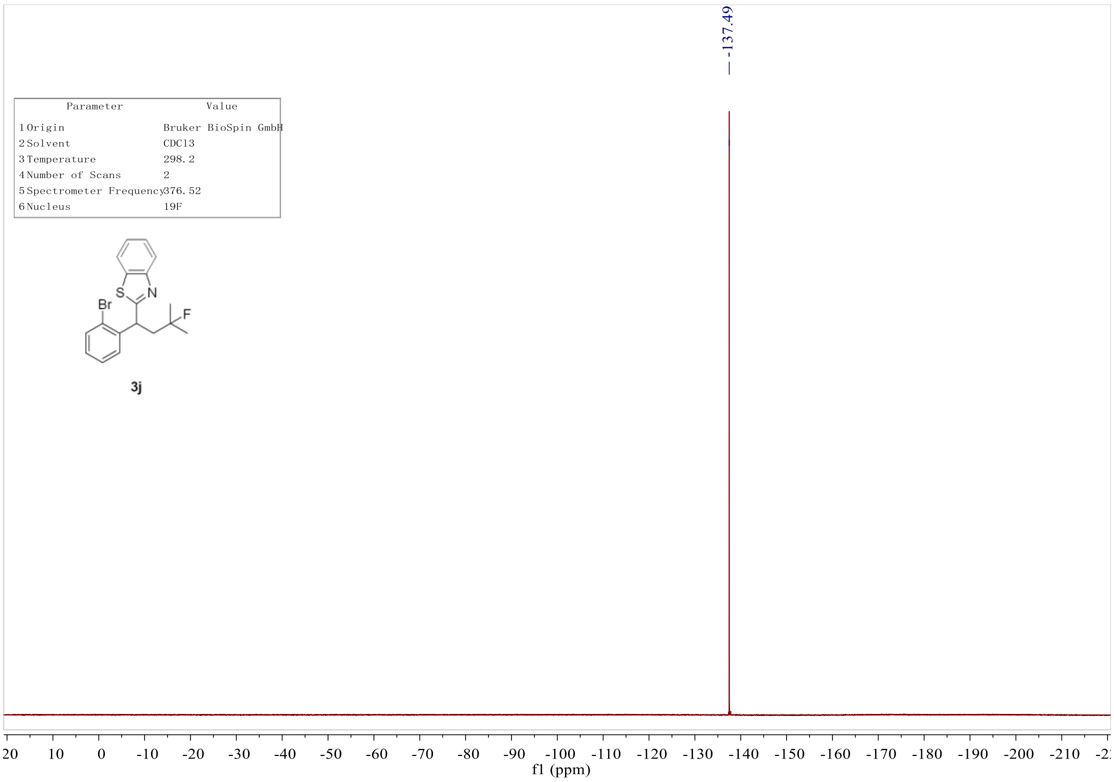

Supplementary Fig. 57 ^{19}F NMR spectra (376 MHz, $CDCl_3$, 25 °C) of **3j**

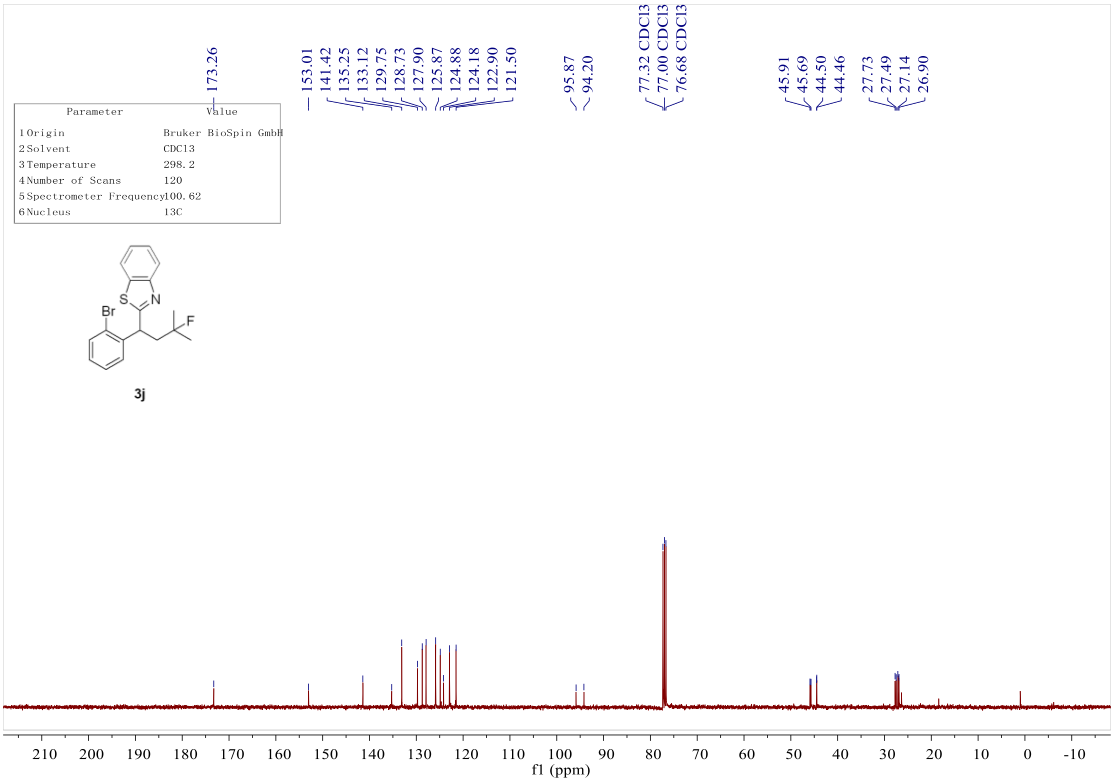

Supplementary Fig. 58 ^{13}C NMR spectra (100 MHz, $CDCl_3$, 25 °C) of **3j**

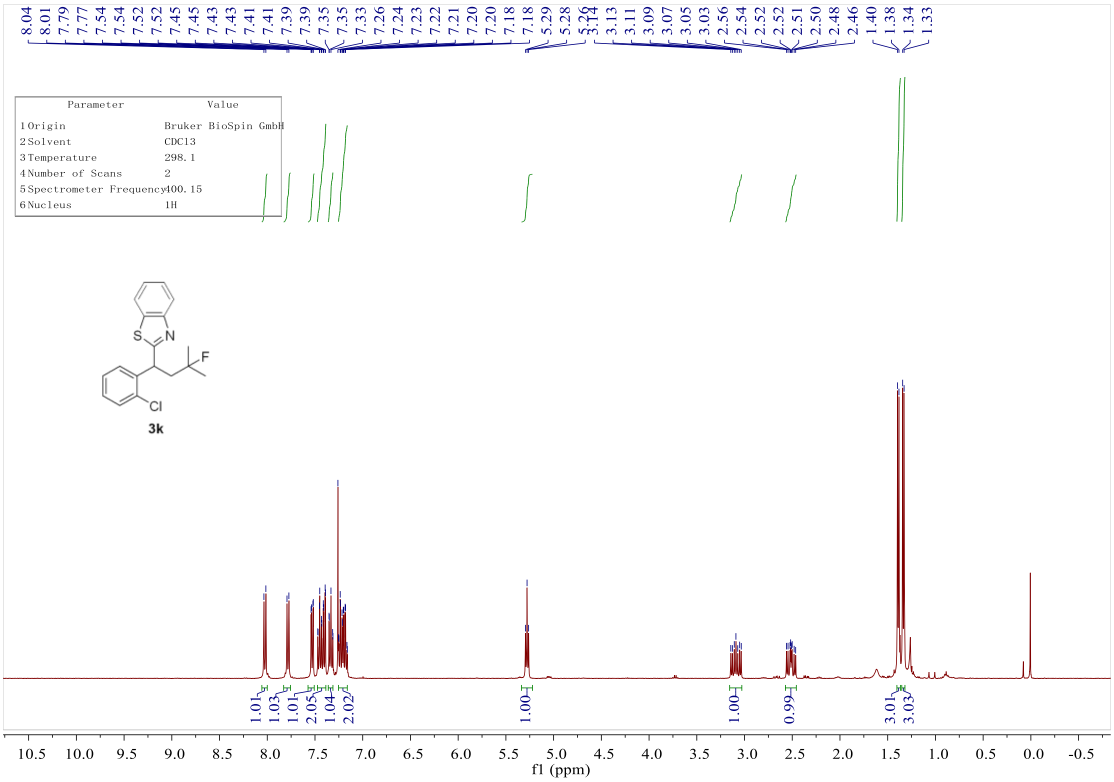

Supplementary Fig. 59 ^{1}H NMR spectra (400 MHz, $CDCl_3$, 25 °C) of **3k**

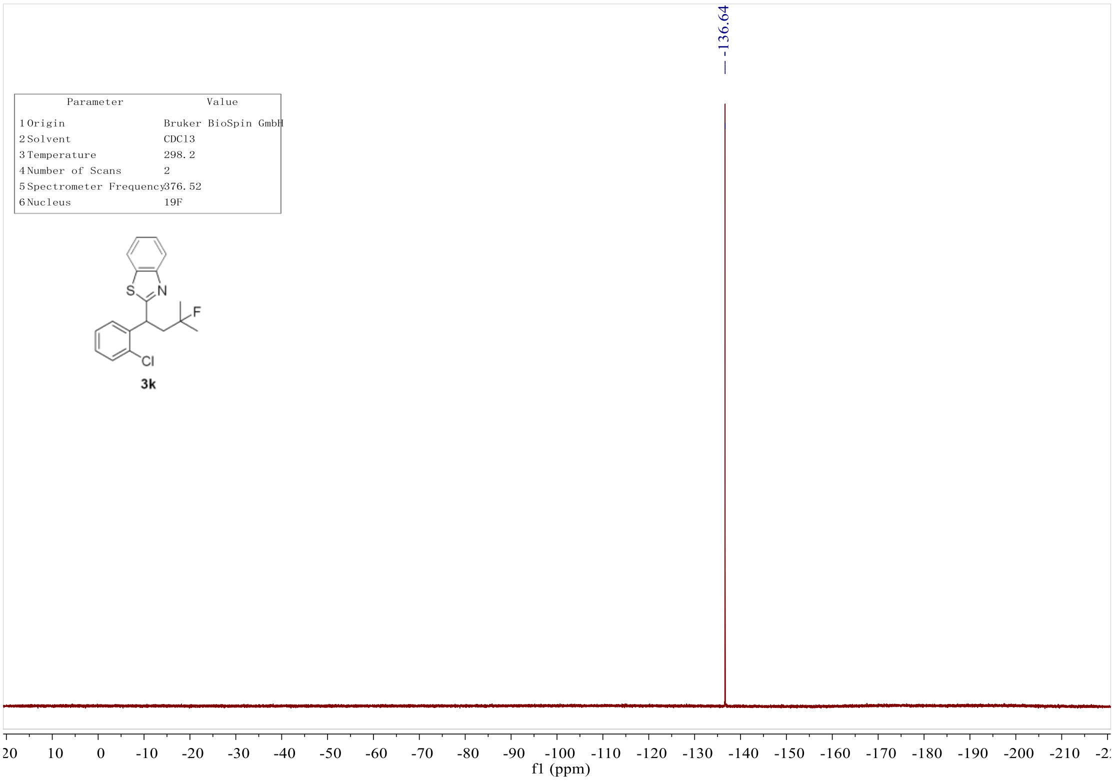

Supplementary Fig. 60 ^{19}F NMR spectra (376 MHz, $CDCl_3$, 25 °C) of **3k**

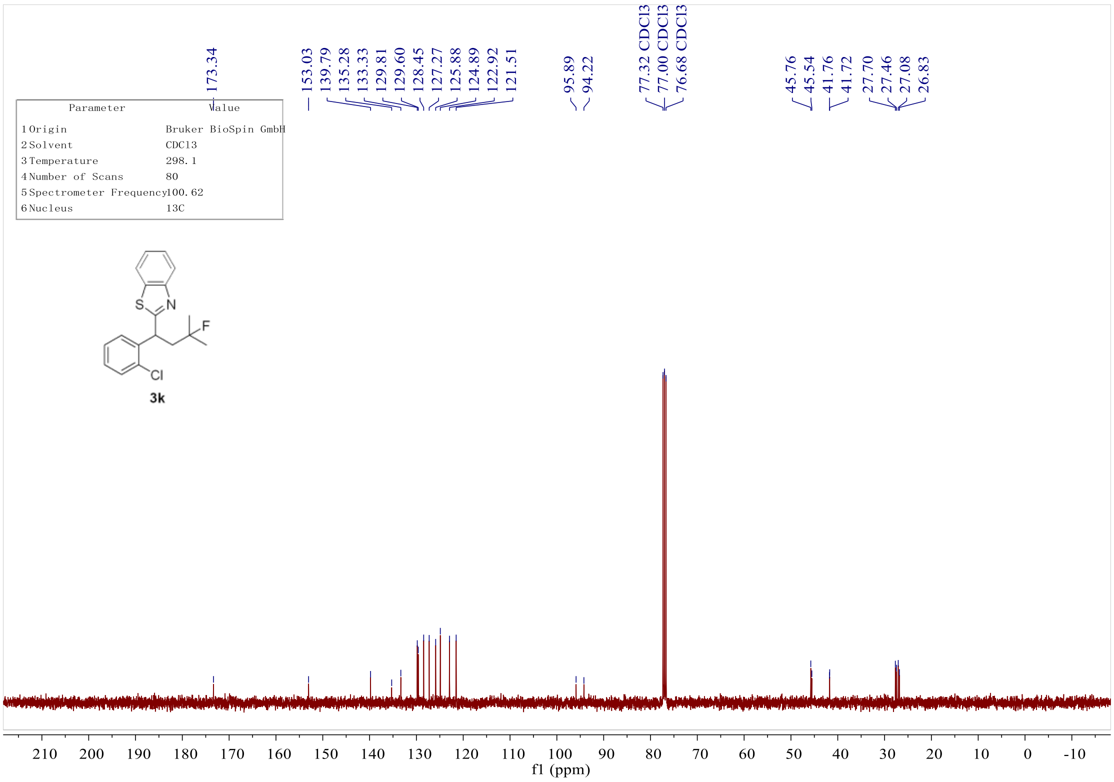

Supplementary Fig. 61 ^{13}C NMR spectra (100 MHz, $CDCl_3$, 25 °C) of **3k**

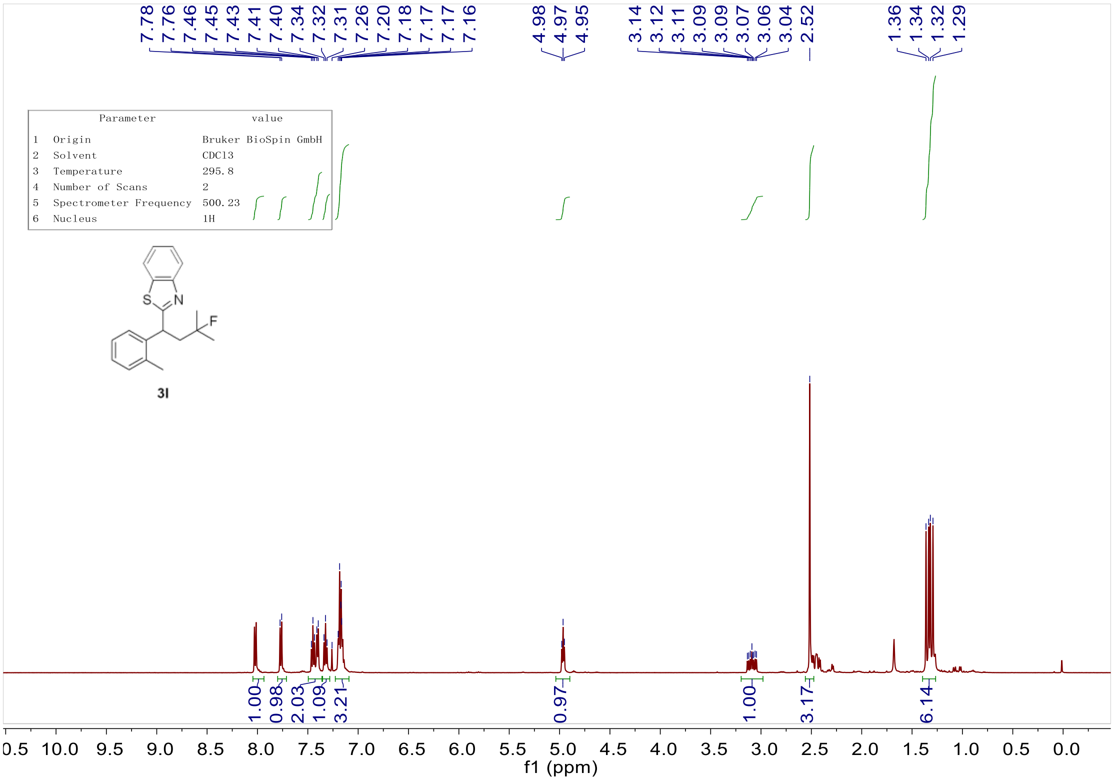

Supplementary Fig. 62 ^{1}H NMR spectra (500 MHz, $CDCl_3$, 25 °C) of **3l**

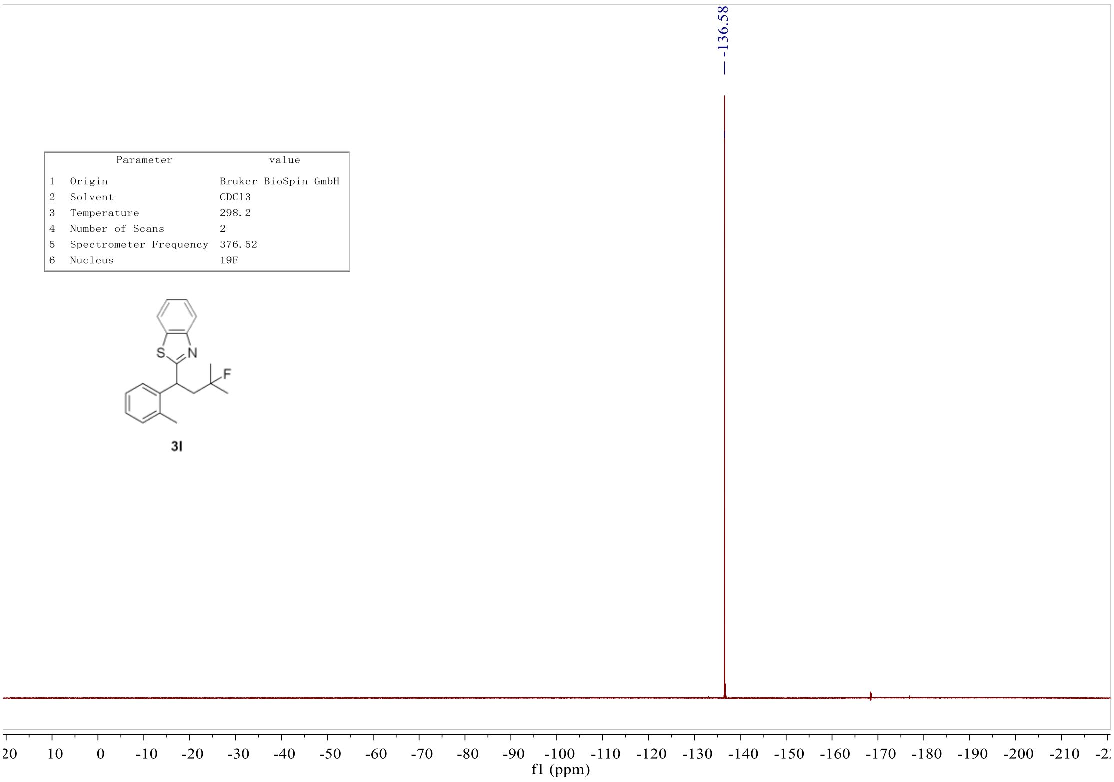

Supplementary Fig. 63 ^{19}F NMR spectra (376 MHz, $CDCl_3$, 25 °C) of **3l**

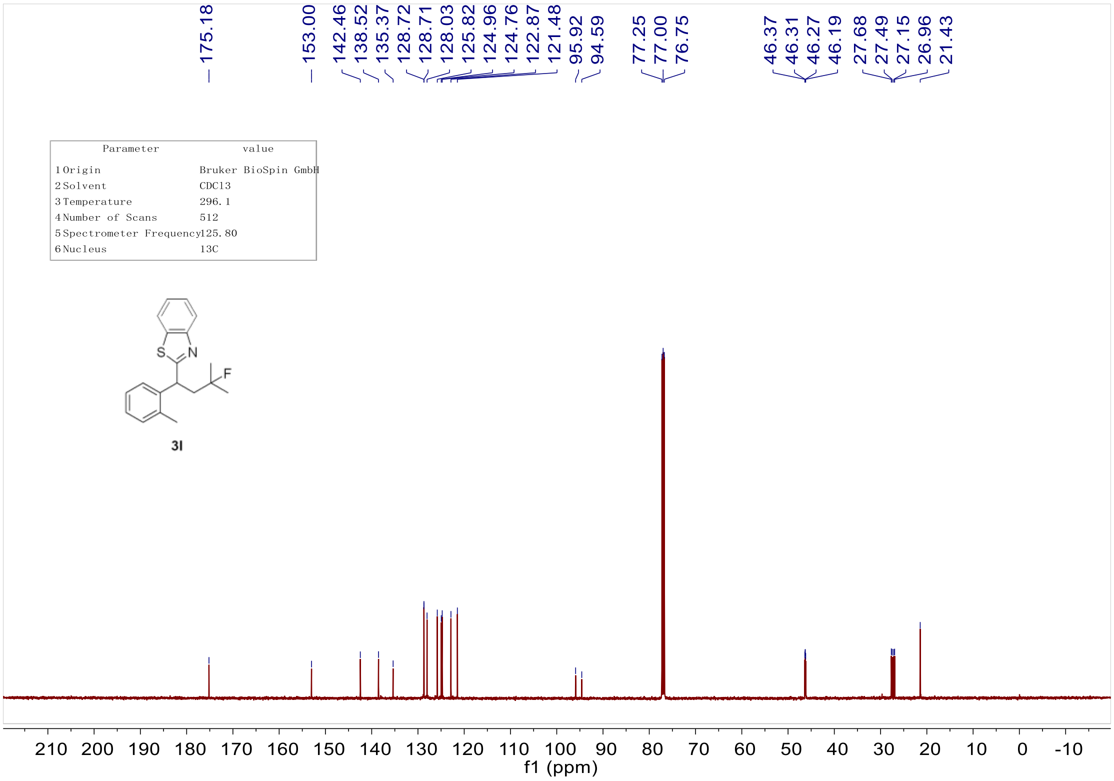

Supplementary Fig. 64 ^{13}C NMR spectra (125 MHz, $CDCl_3$, 25 ºC) of **3l**

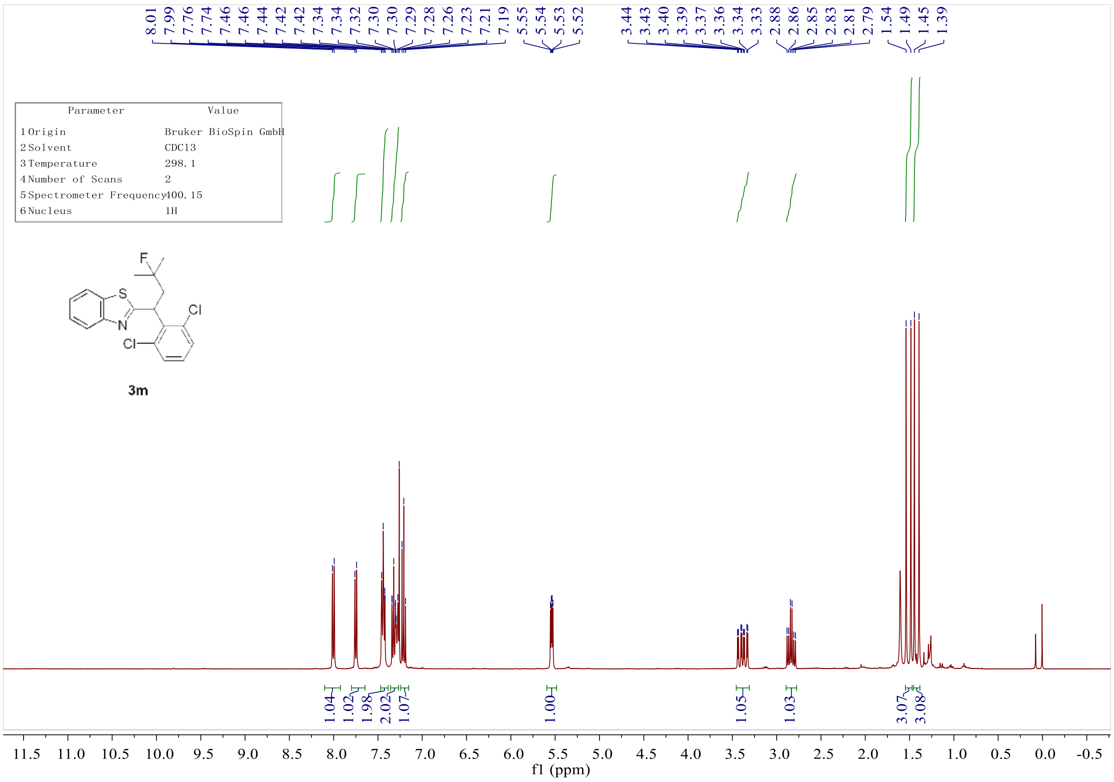

Supplementary Fig. 65 ^{1}H NMR spectra (400 MHz, $CDCl_3$, 25 ºC) of **3m**

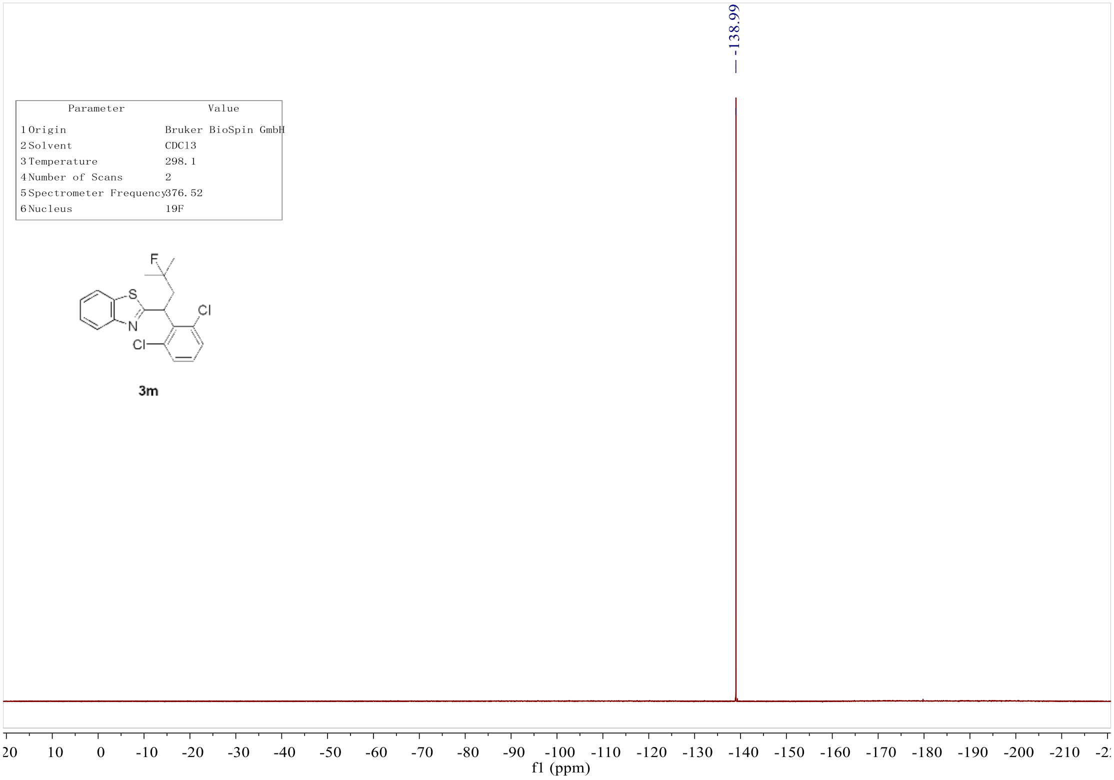

Supplementary Fig. 66 ^{19}F NMR spectra (376 MHz, $CDCl_3$, 25 °C) of **3m**

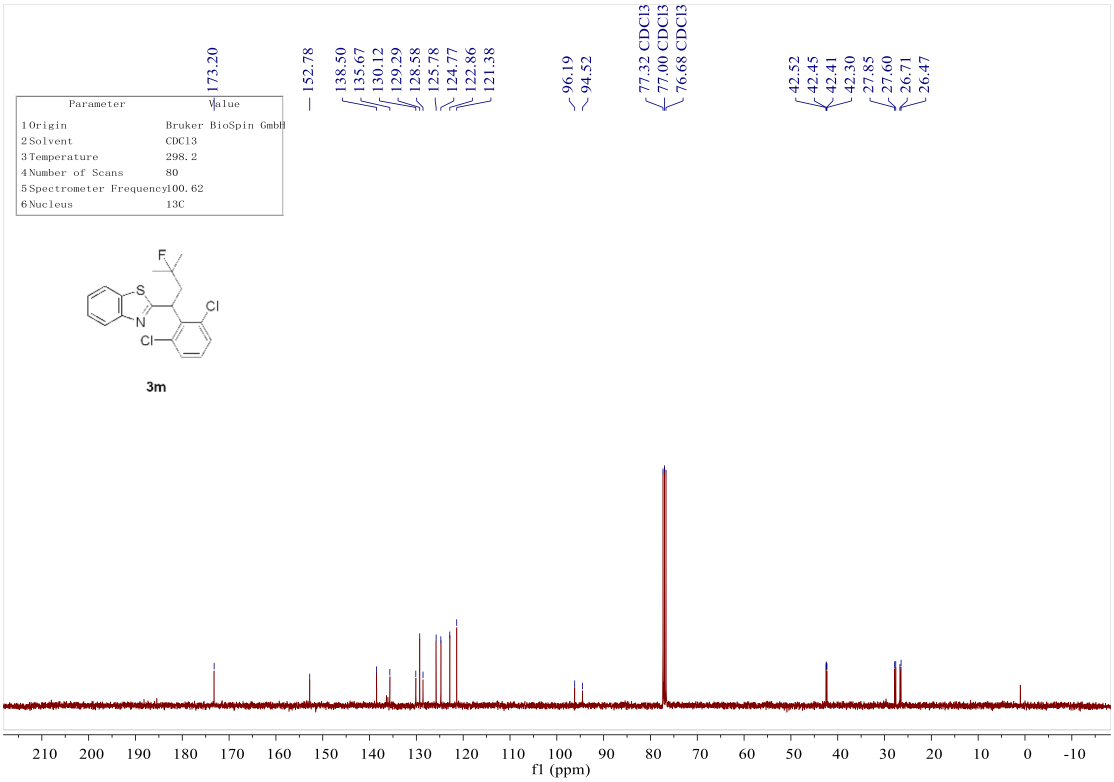

Supplementary Fig. 67 ^{13}C NMR spectra (100 MHz, $CDCl_3$, 25 °C) of **3m**

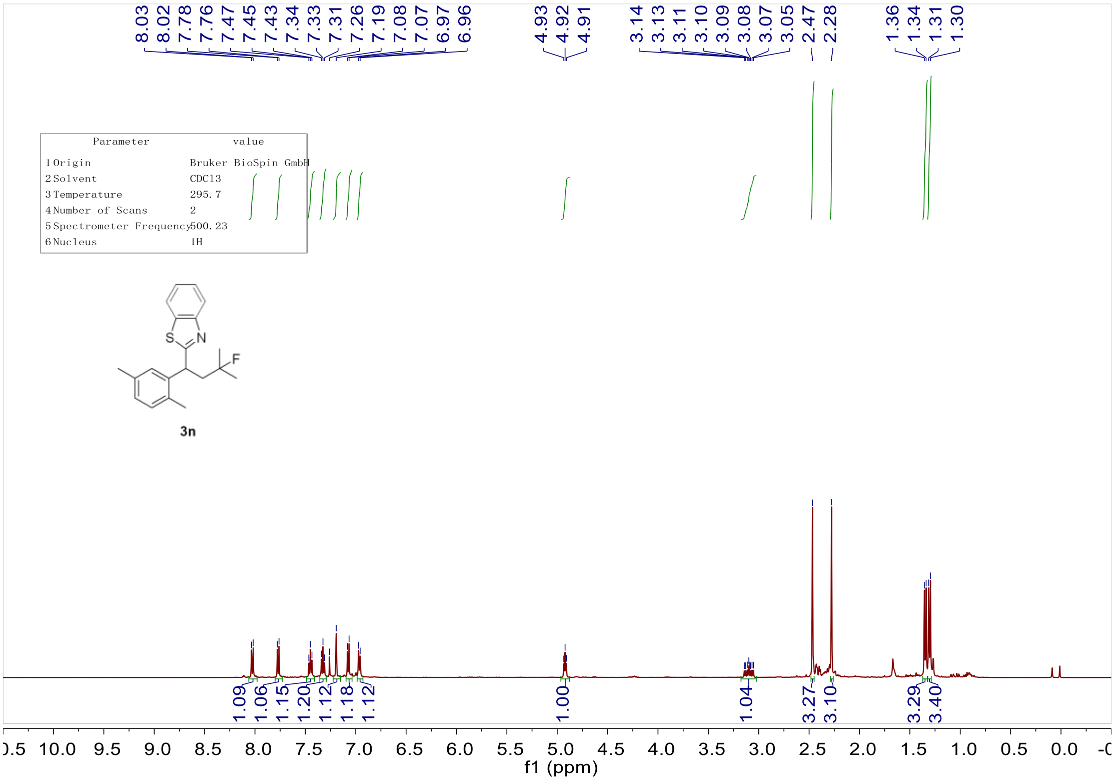

Supplementary Fig. 68 ^{1}H NMR spectra (500 MHz, $CDCl_3$, 25 °C) of **3n**

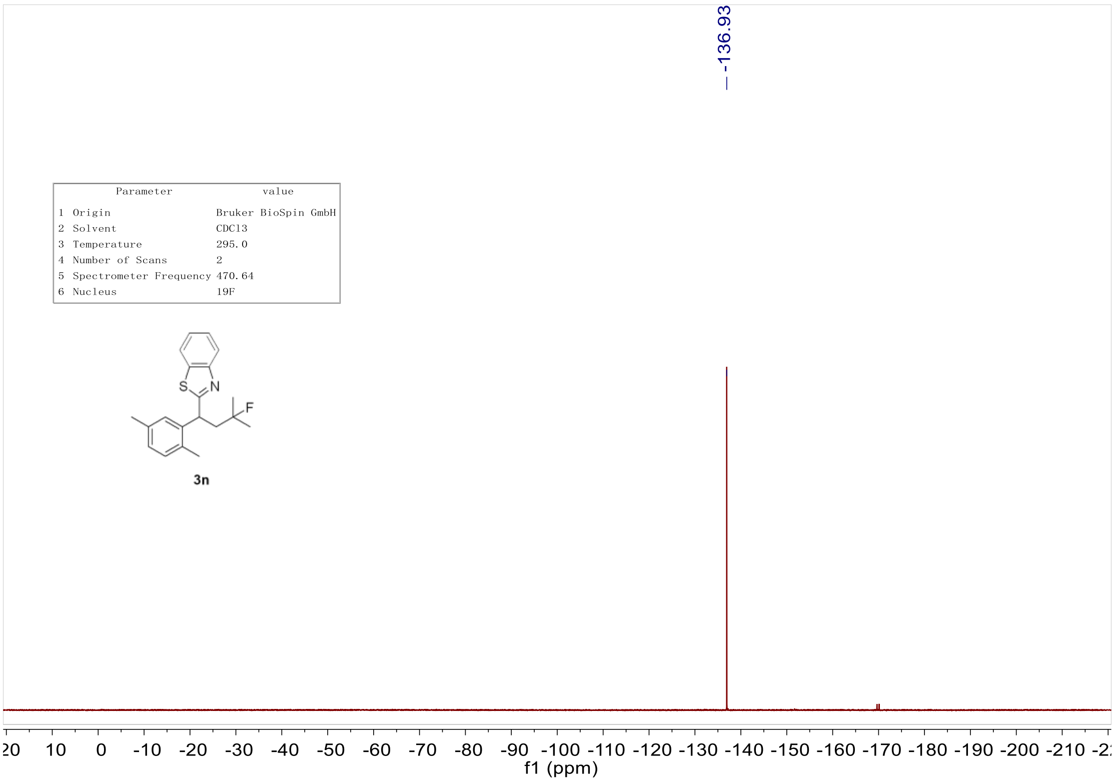

Supplementary Fig. 69 ^{19}F NMR spectra (471 MHz, $CDCl_3$, 25 °C) of **3n**

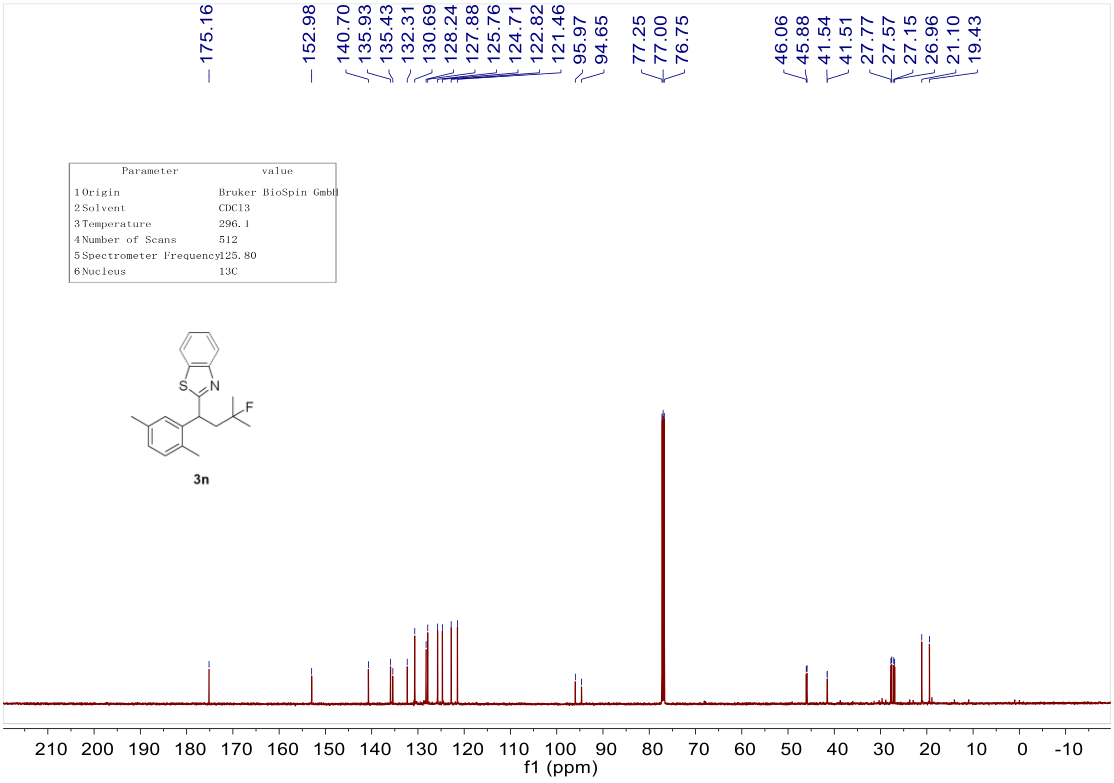

Supplementary Fig. 70 ^{13}C NMR spectra (125 MHz, $CDCl_3$, 25 °C) of **3n**

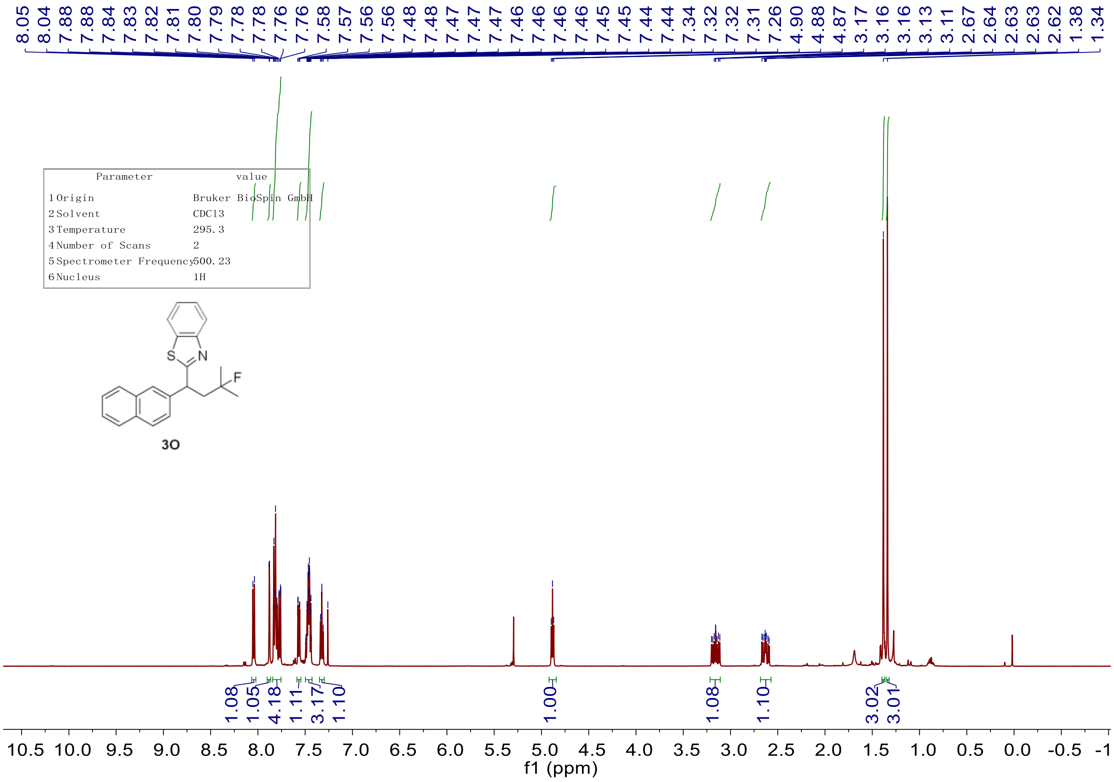

Supplementary Fig. 71 ^{1}H NMR spectra (500 MHz, $CDCl_3$, 25 °C) of **3o**

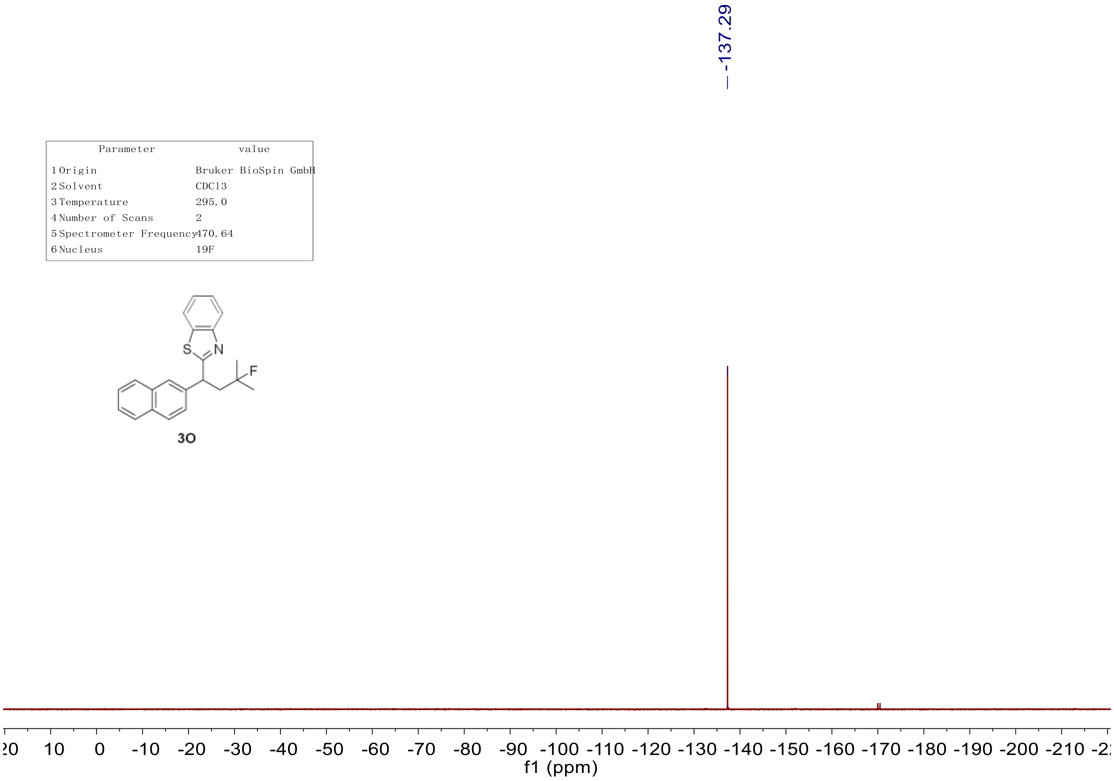

Supplementary Fig. 72 ^{19}F NMR spectra (471 MHz, $CDCl_3$, 25 °C) of **3o**

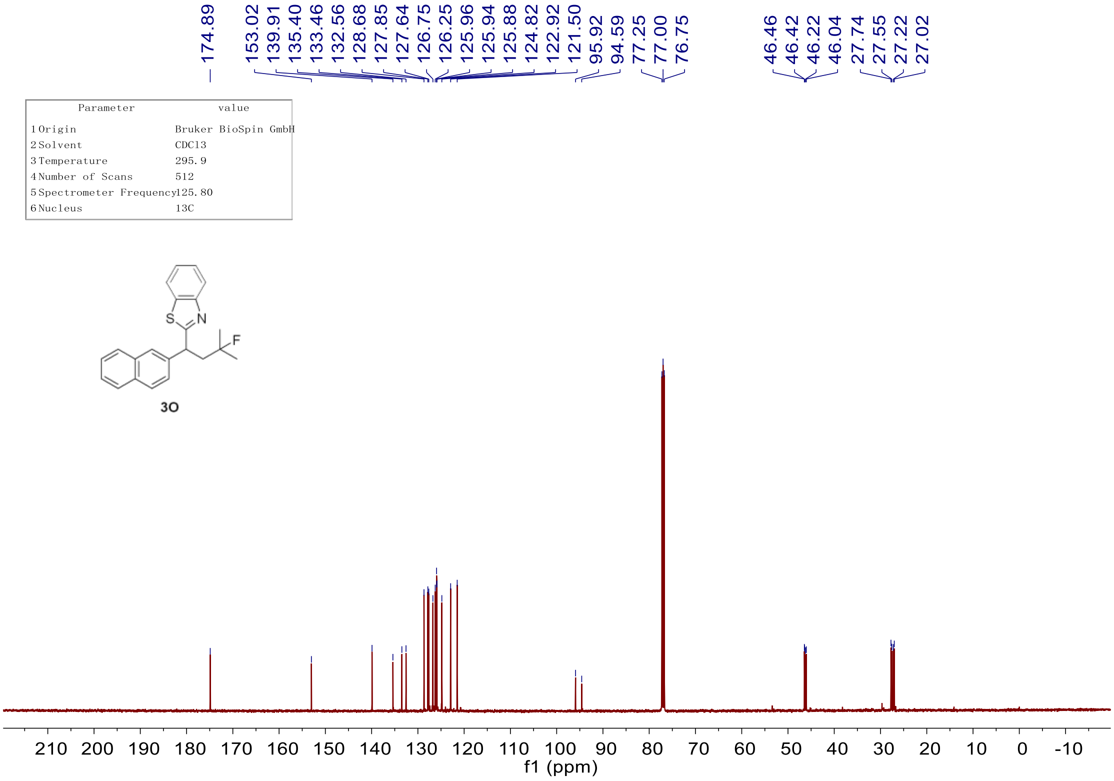

Supplementary Fig. 73 ^{13}C NMR spectra (125 MHz, $CDCl_3$, 25 °C) of **3o**

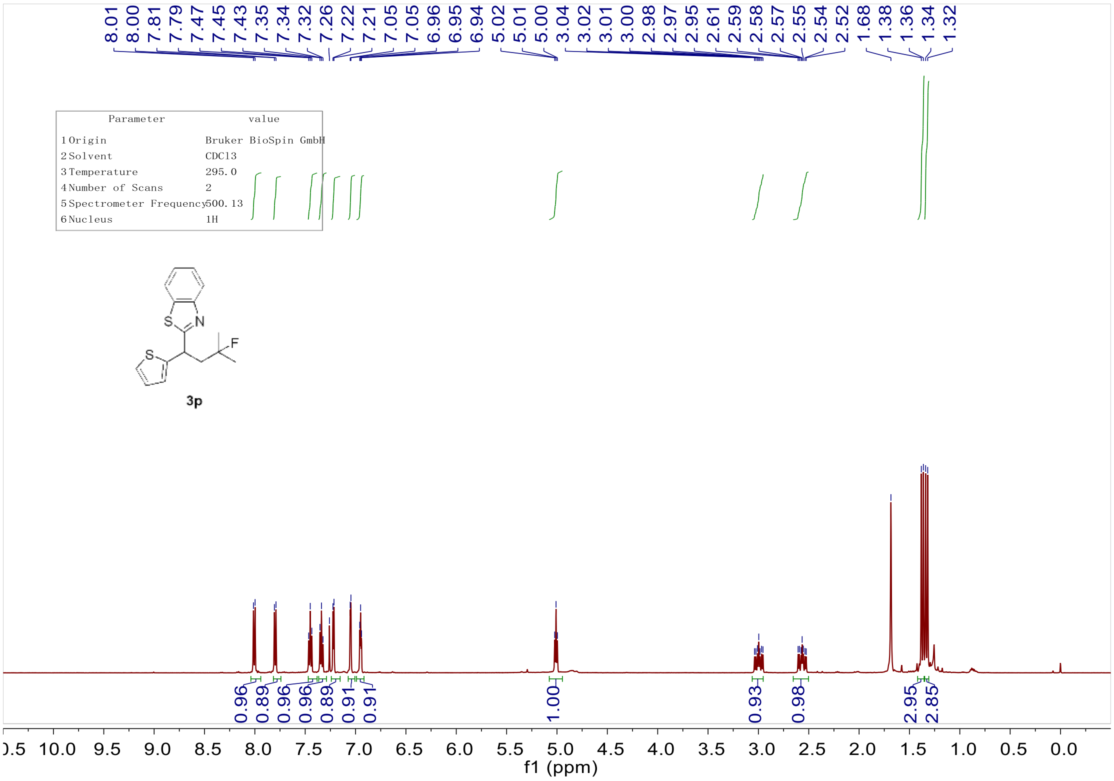

Supplementary Fig. 74 ^{1}H NMR spectra (500 MHz, $CDCl_3$, 25 °C) of **3p**

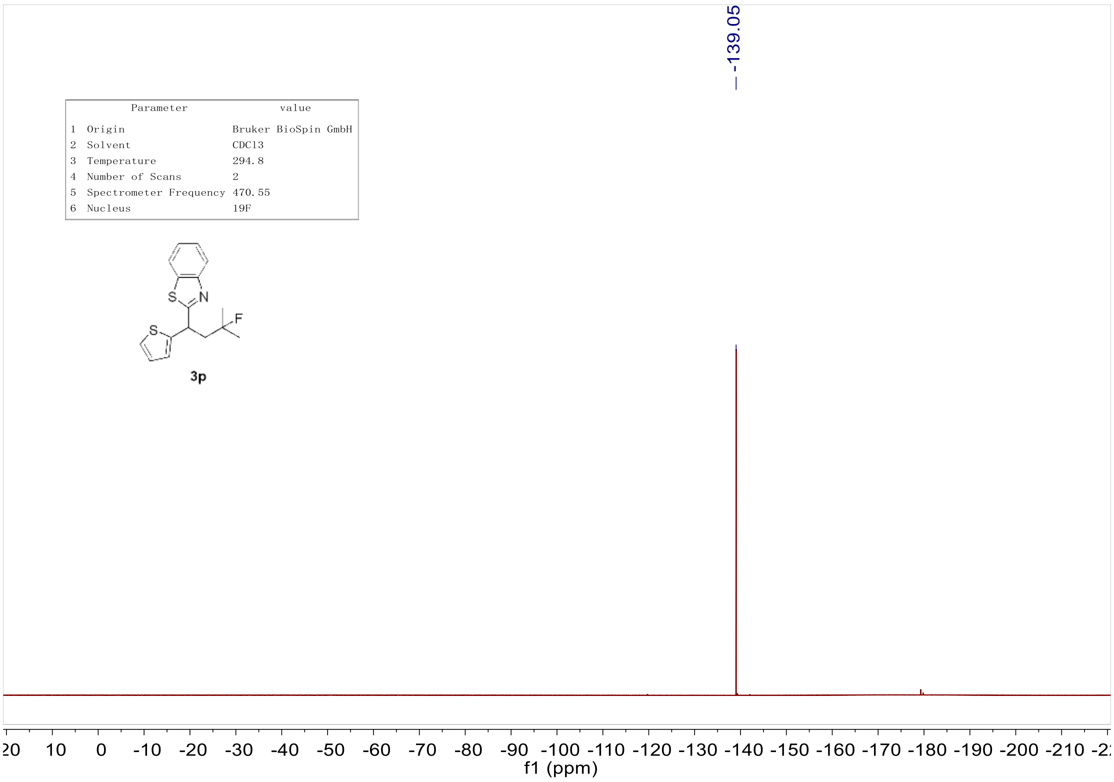

Supplementary Fig. 75 ^{19}F NMR spectra (471 MHz, $CDCl_3$, 25 °C) of **3p**

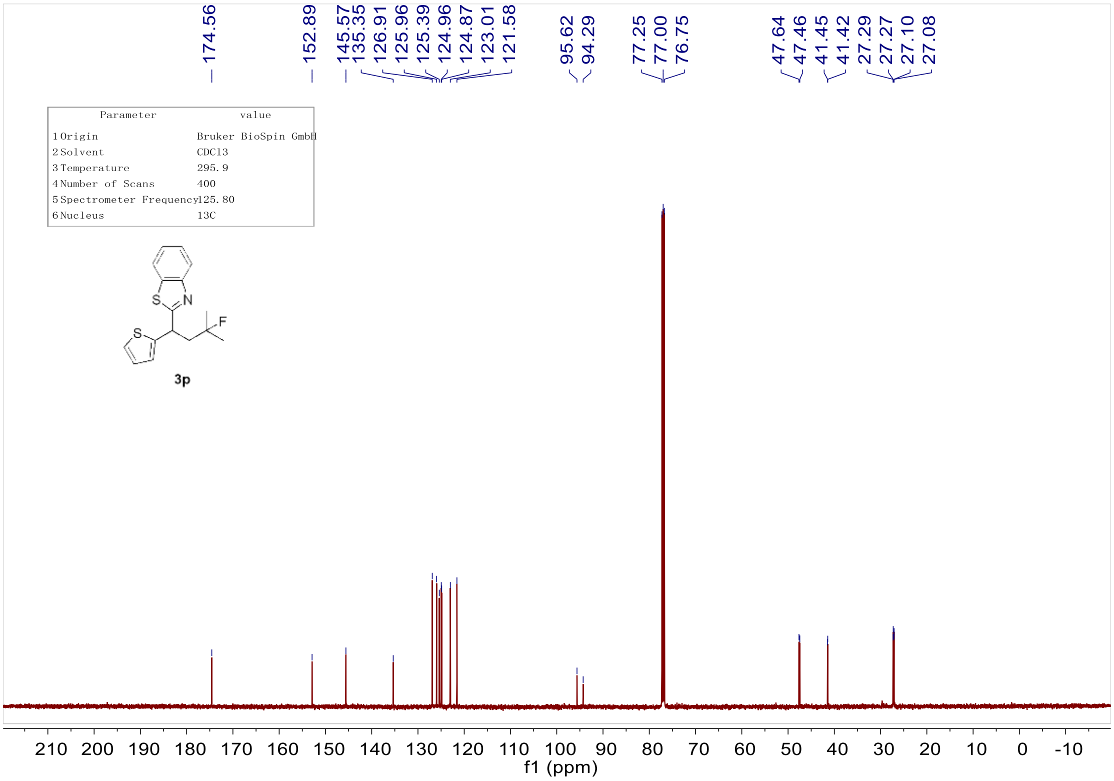

Supplementary Fig. 76 ^{13}C NMR spectra (125 MHz, $CDCl_3$, 25 ºC) of **3p**

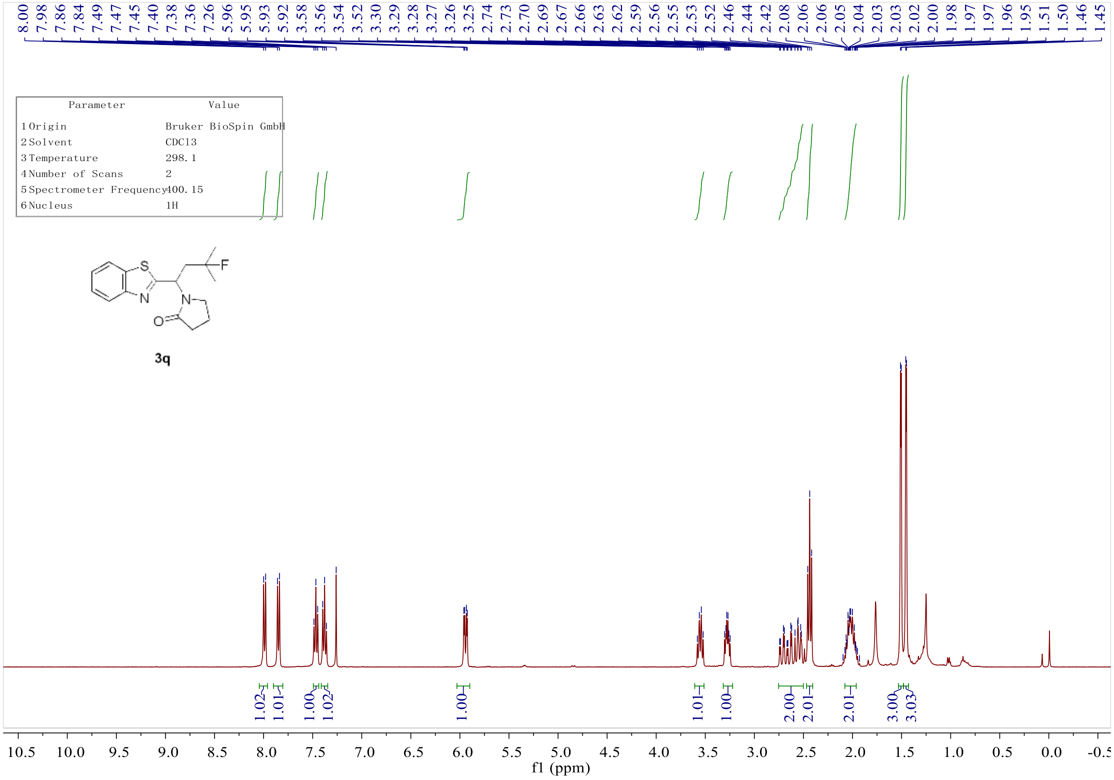

Supplementary Fig. 77 ^{1}H NMR spectra (400 MHz, $CDCl_3$, 25 ºC) of **3q**

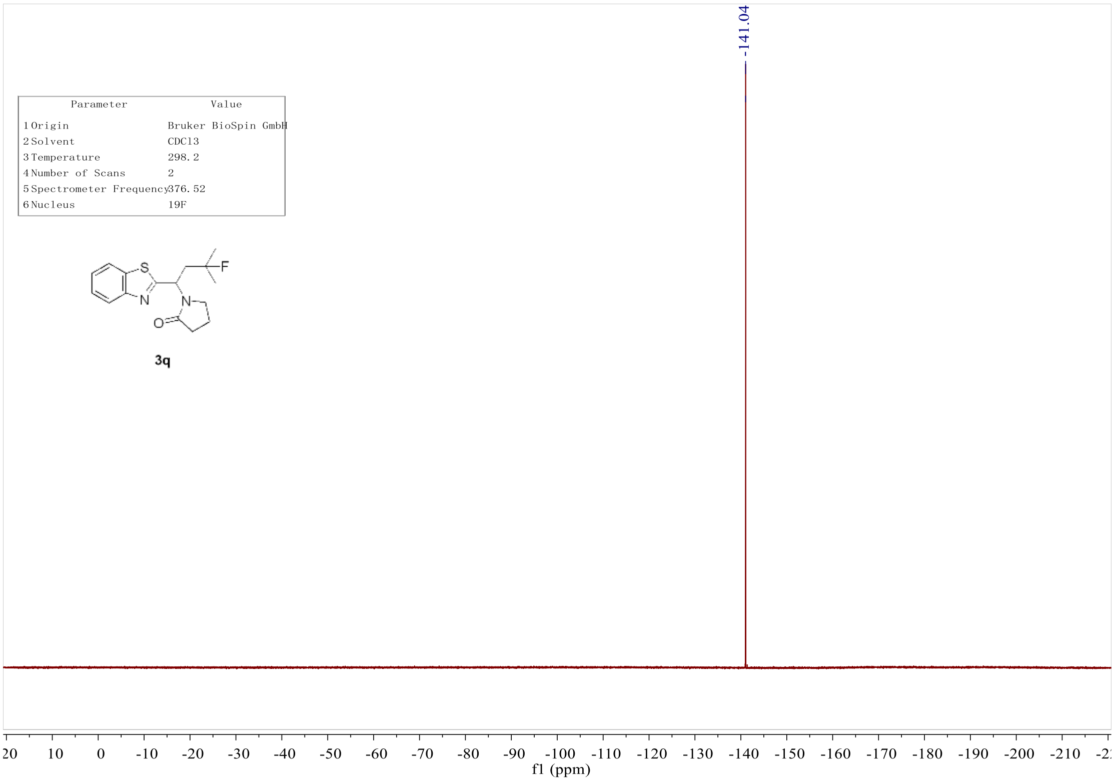

Supplementary Fig. 78 ^{19}F NMR spectra (376 MHz, $CDCl_3$, 25 °C) of **3q**

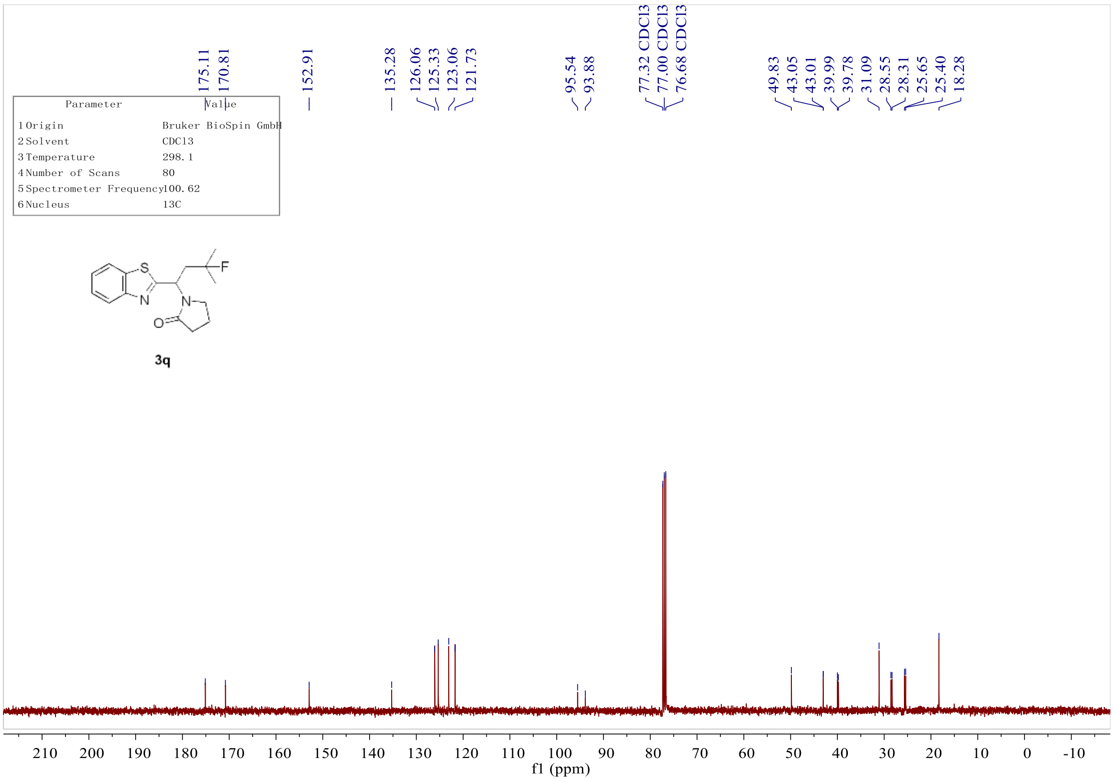

Supplementary Fig. 79 ^{13}C NMR spectra (100 MHz, $CDCl_3$, 25 °C) of **3q**

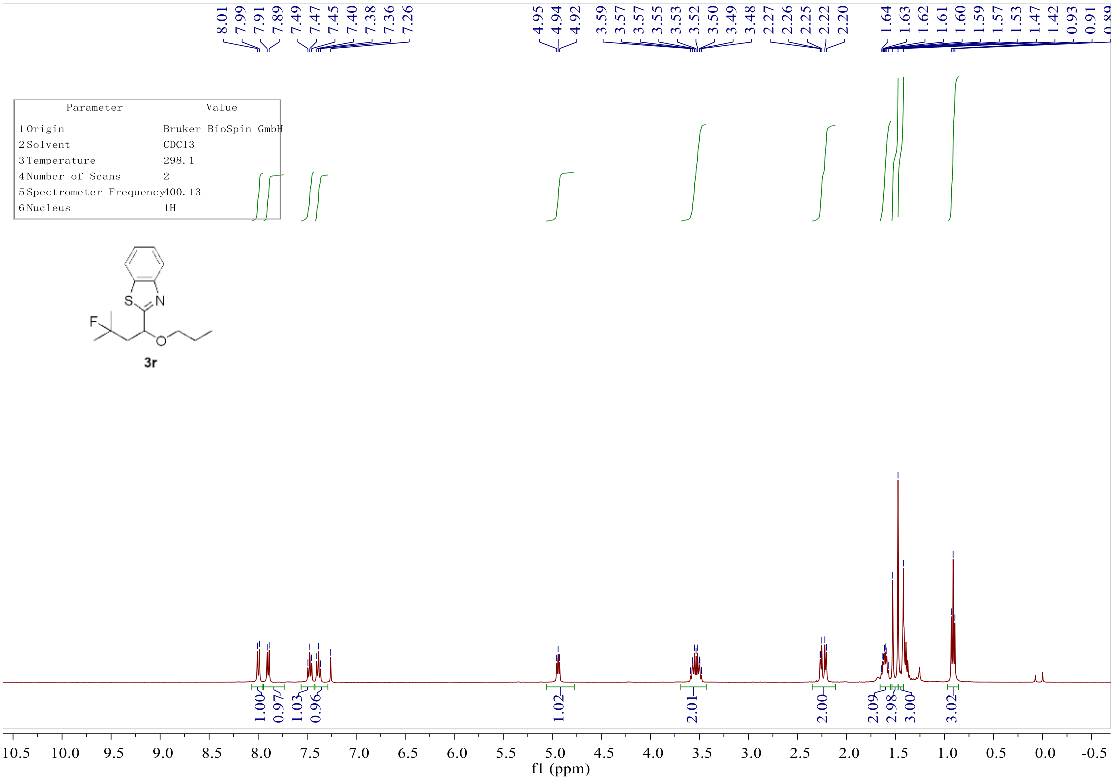

Supplementary Fig. 80 ^{1}H NMR spectra (400 MHz, $CDCl_3$, 25 °C) of **3r**

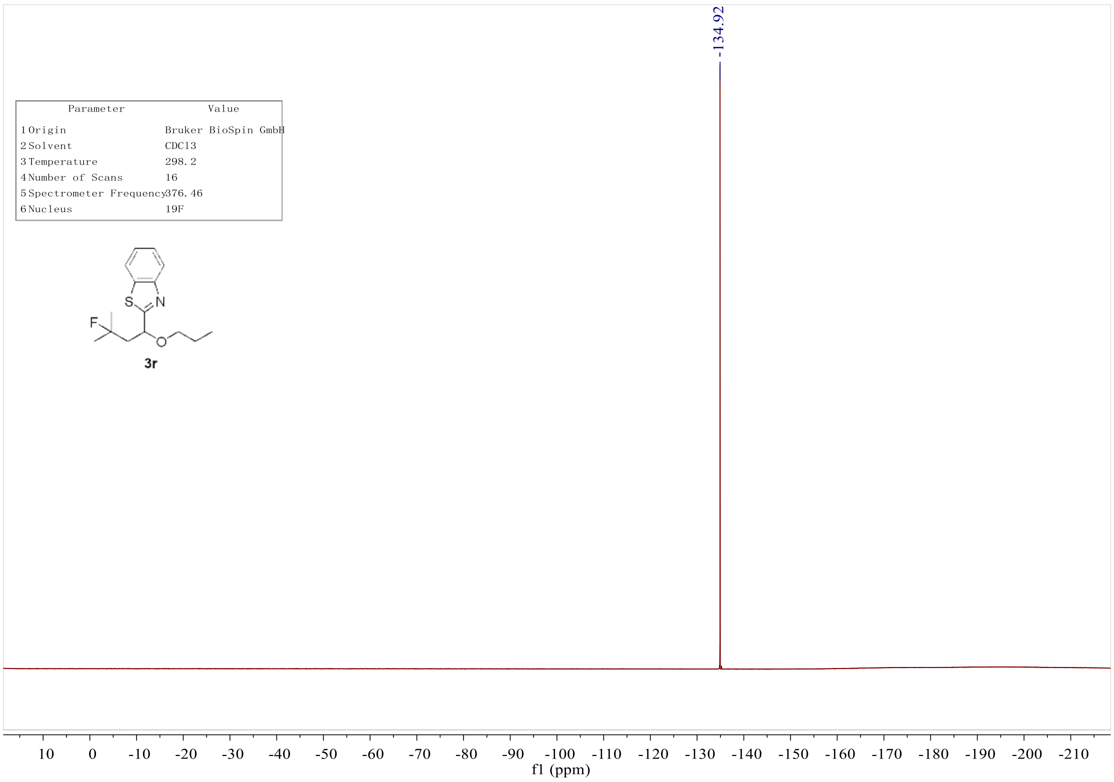

Supplementary Fig. 81 ^{19}F NMR spectra (376 MHz, $CDCl_3$, 25 °C) of **3r**

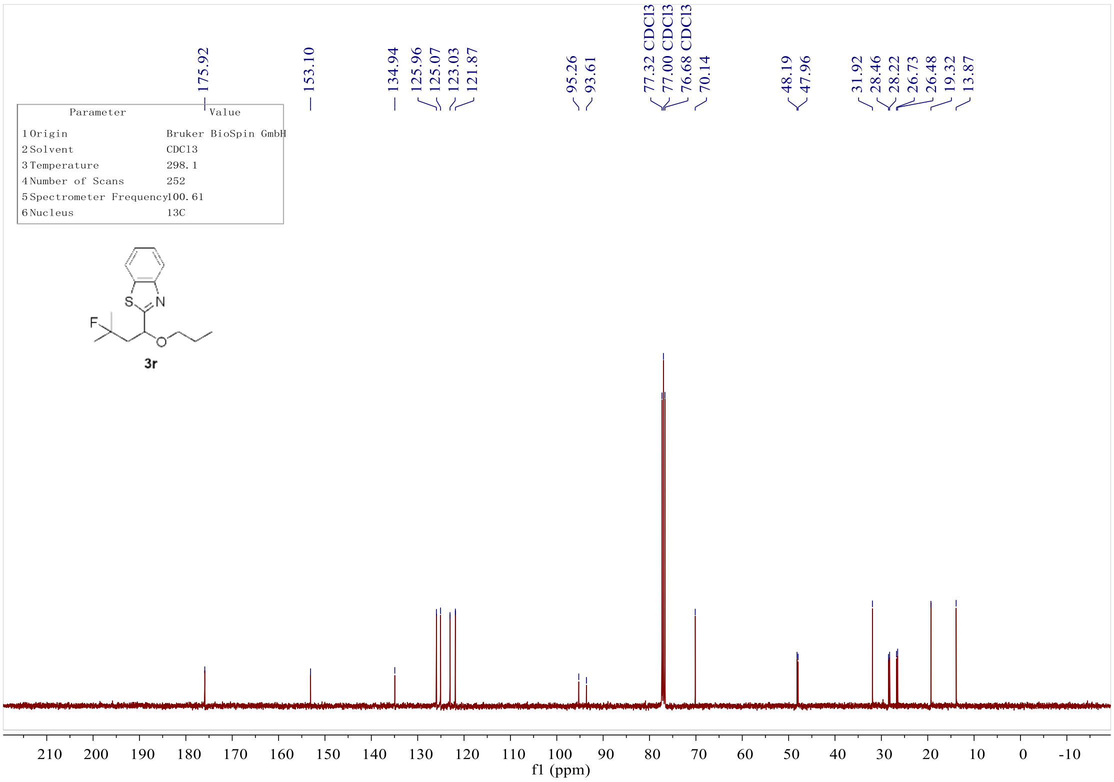

Supplementary Fig. 82 ^{13}C NMR spectra (100 MHz, CDCl$_3$, 25 °C) of **3r**

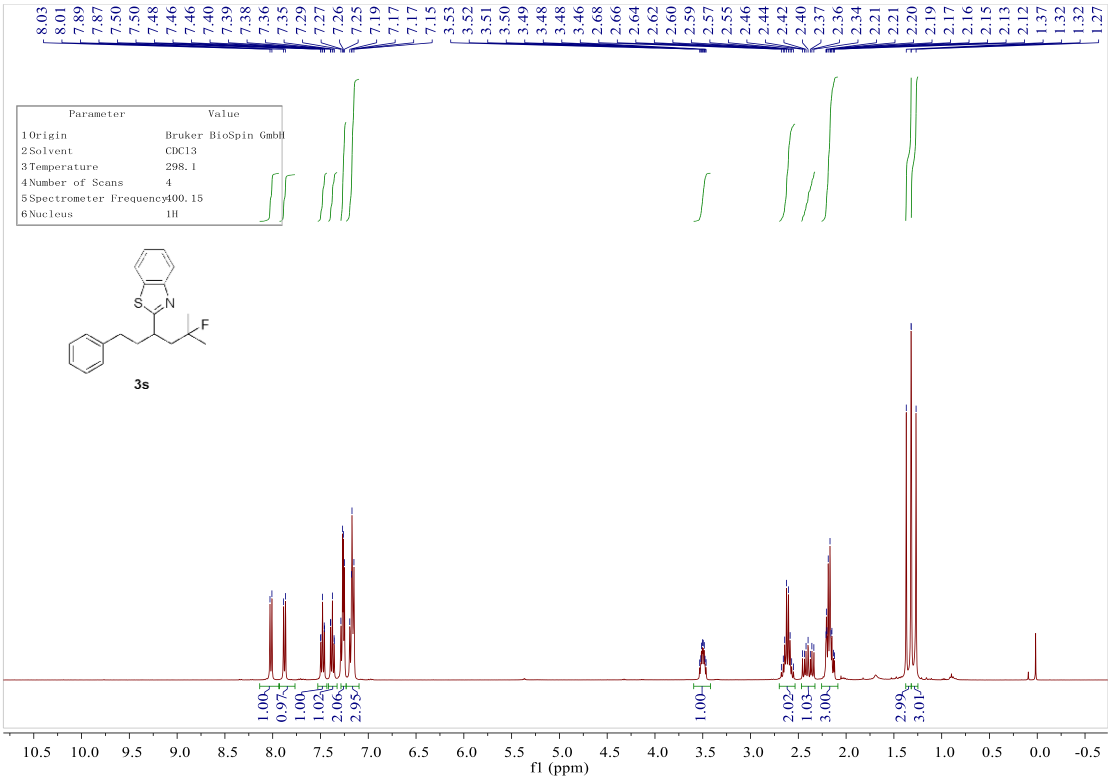

Supplementary Fig. 83 ^{1}H NMR spectra (400 MHz, CDCl$_3$, 25 °C) of **3s**

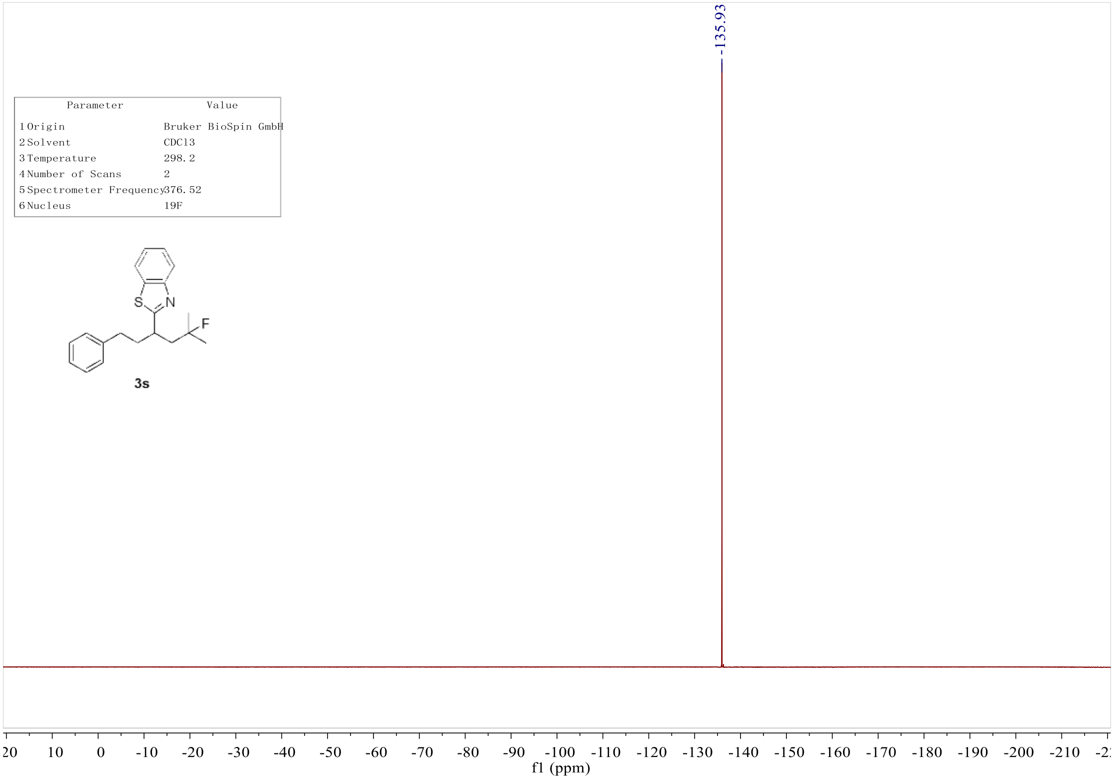

Supplementary Fig. 84 ^{19}F NMR spectra (376 MHz, $CDCl_3$, 25 °C) of **3s**

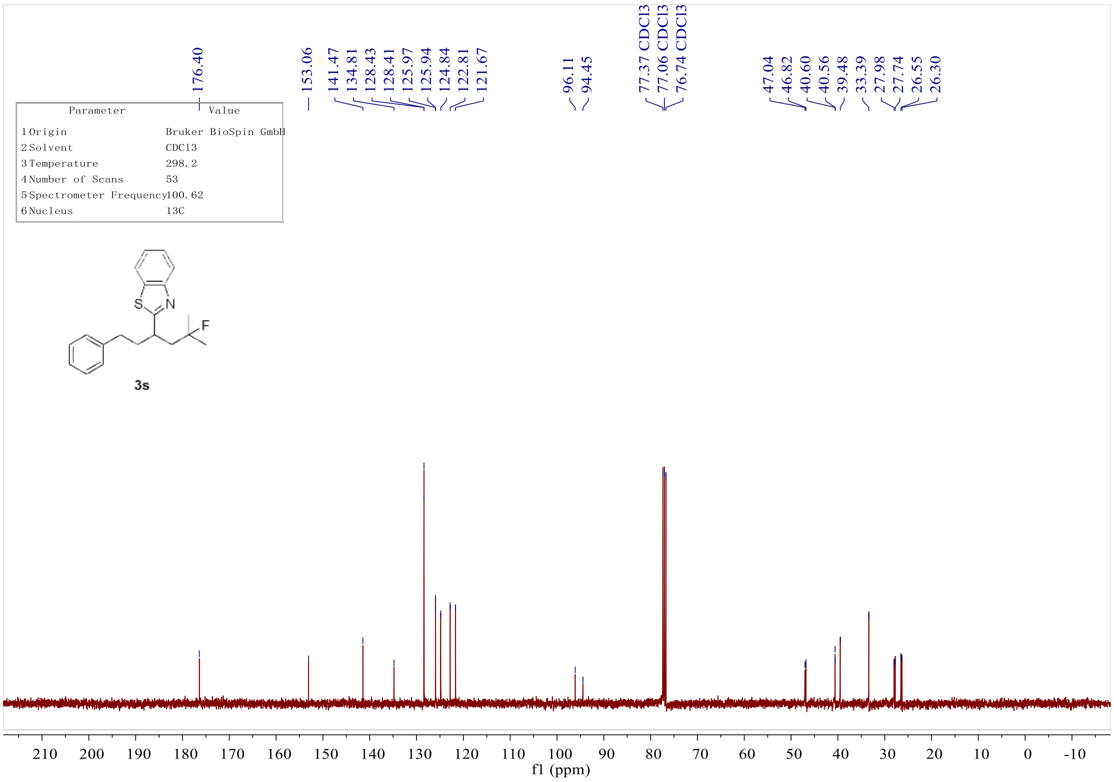

Supplementary Fig. 85 ^{13}C NMR spectra (100 MHz, $CDCl_3$, 25 °C) of **3s**

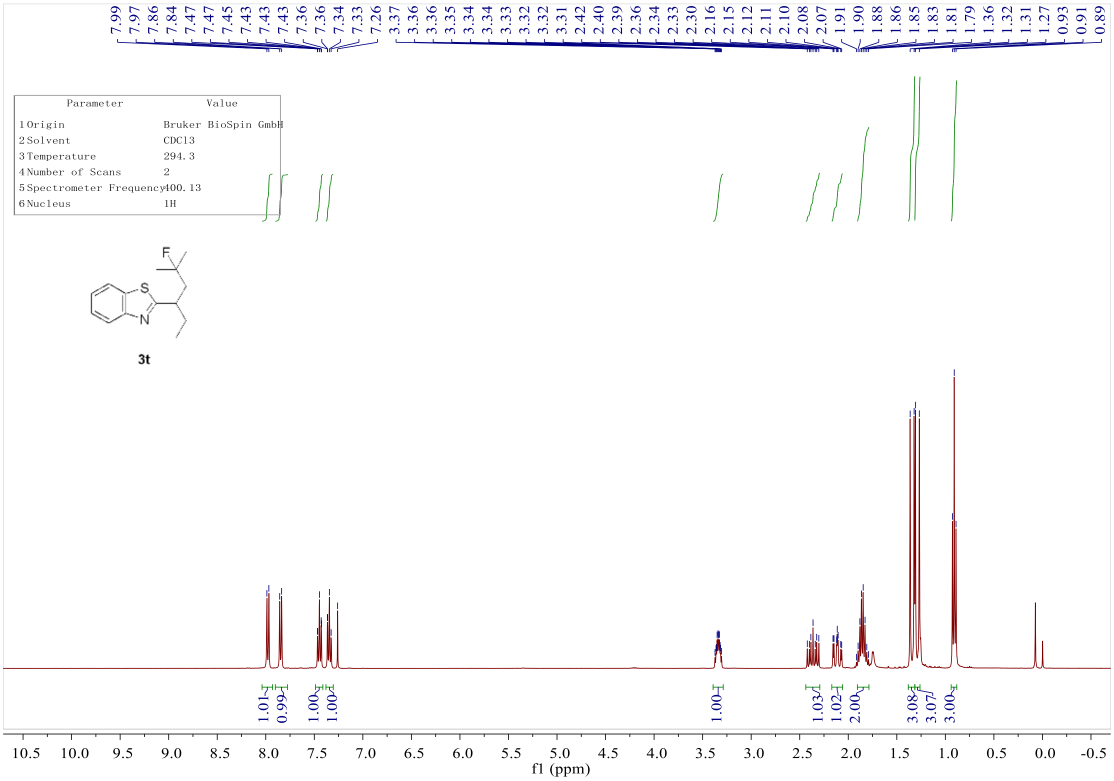

Supplementary Fig. 86 ^{1}H NMR spectra (400 MHz, $CDCl_3$, 25 °C) of **3t**

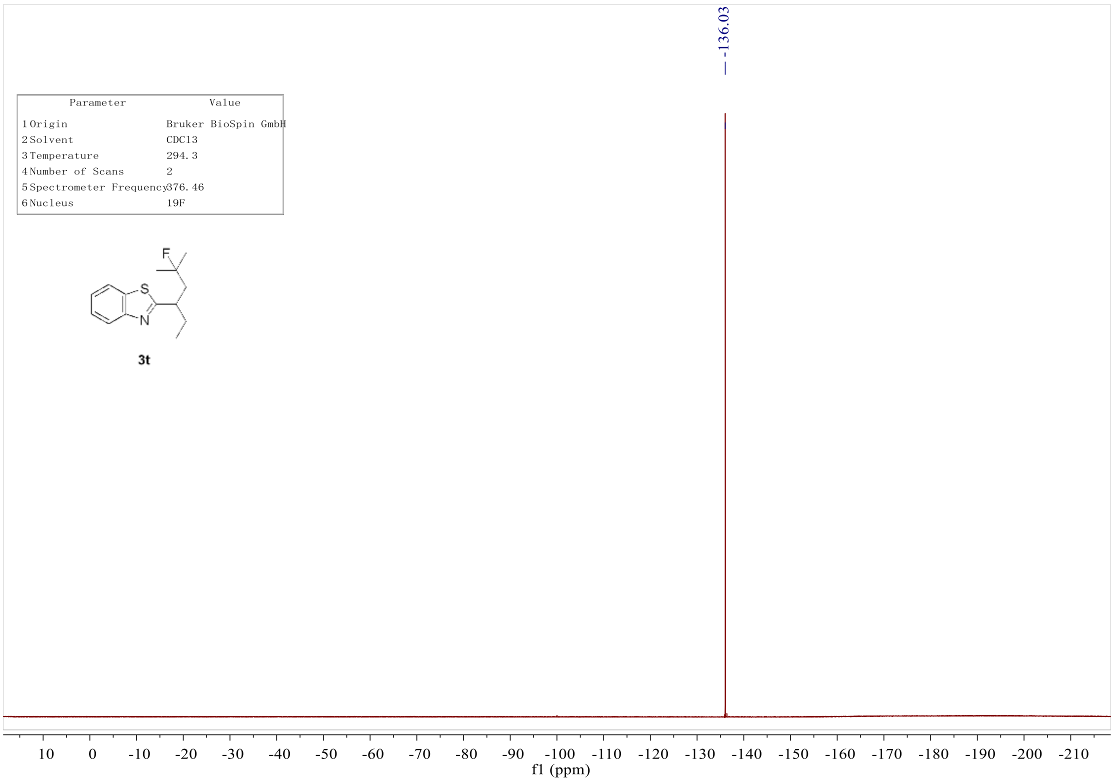

Supplementary Fig. 87 ^{19}F NMR spectra (376 MHz, $CDCl_3$, 25 °C) of **3t**

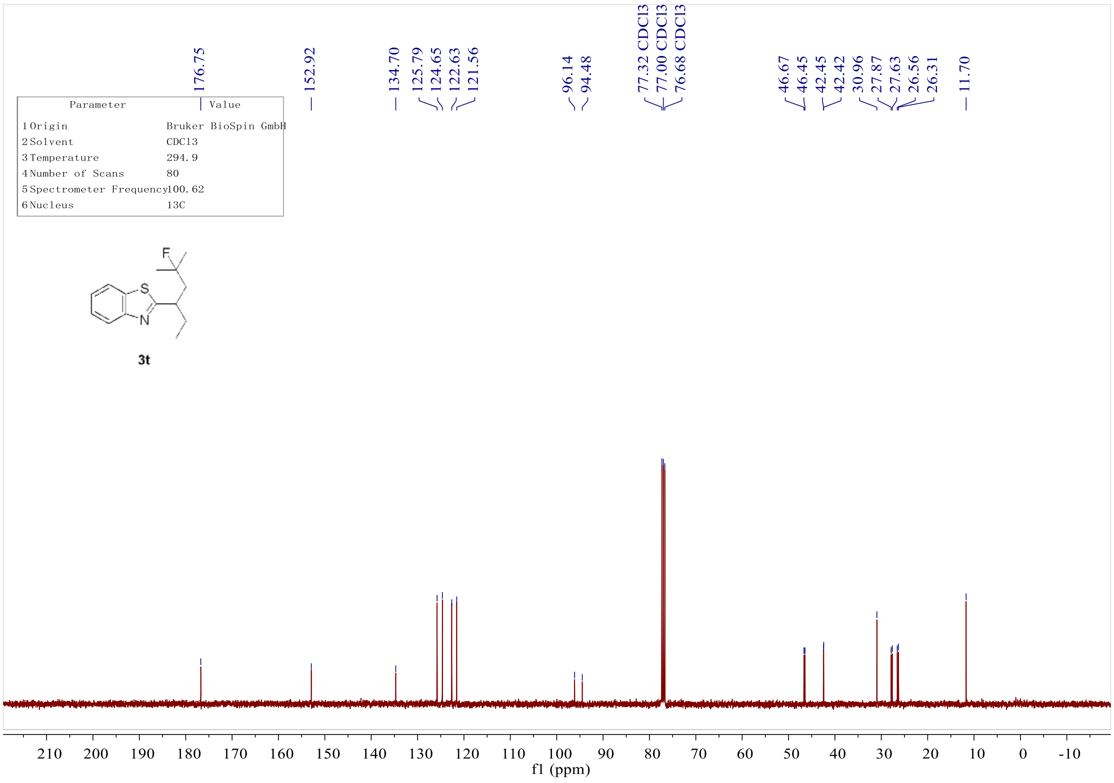

Supplementary Fig. 88 ^{13}C NMR spectra (100 MHz, $CDCl_3$, 25 ºC) of **3t**

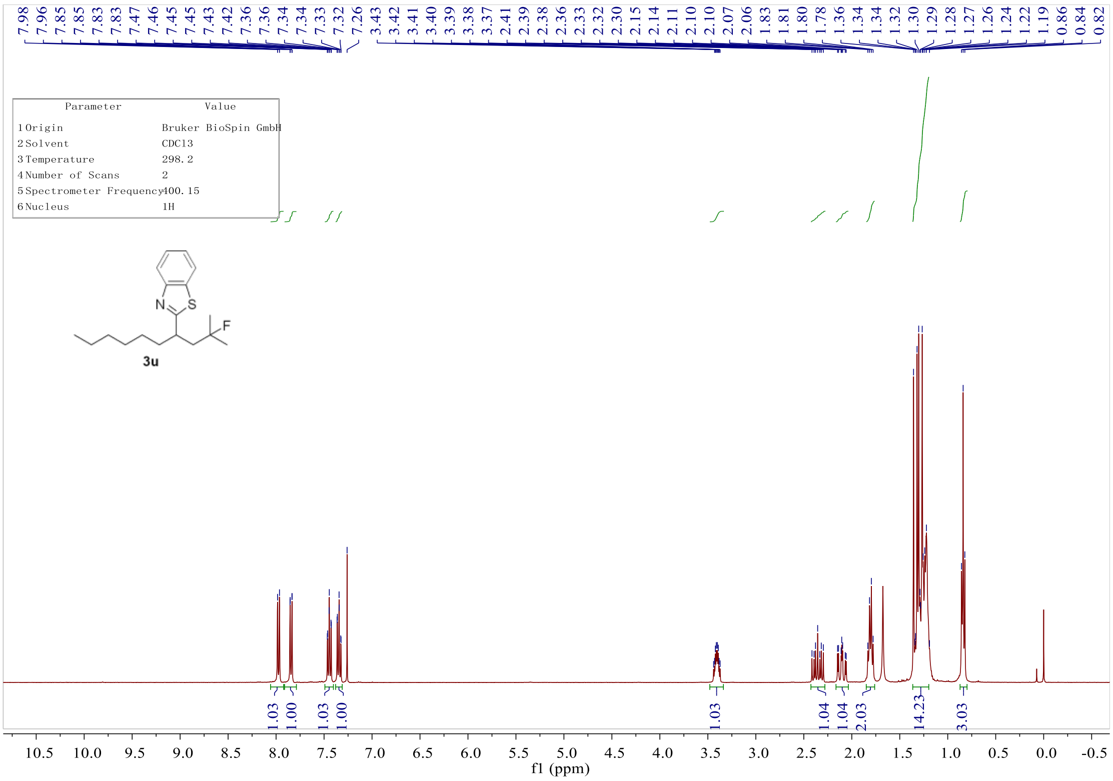

Supplementary Fig. 89 ^{1}H NMR spectra (400 MHz, $CDCl_3$, 25 ºC) of **3u**

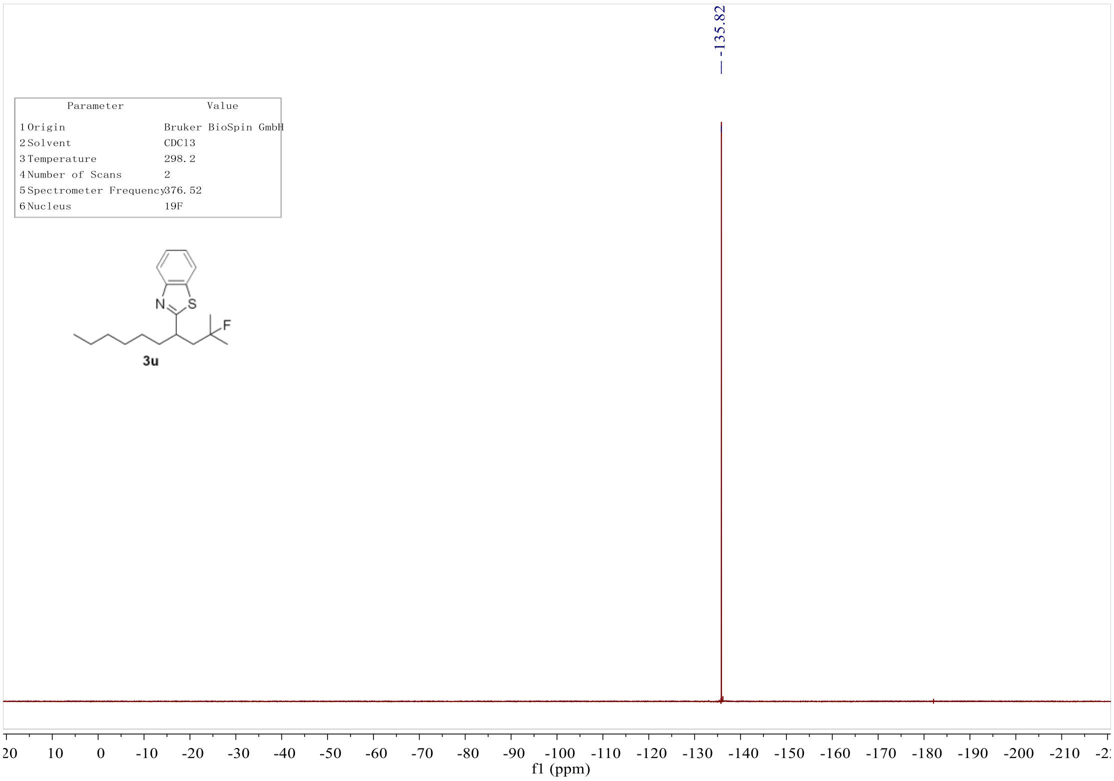

Supplementary Fig. 90 ^{19}F NMR spectra (376 MHz, $CDCl_3$, 25 °C) of **3u**

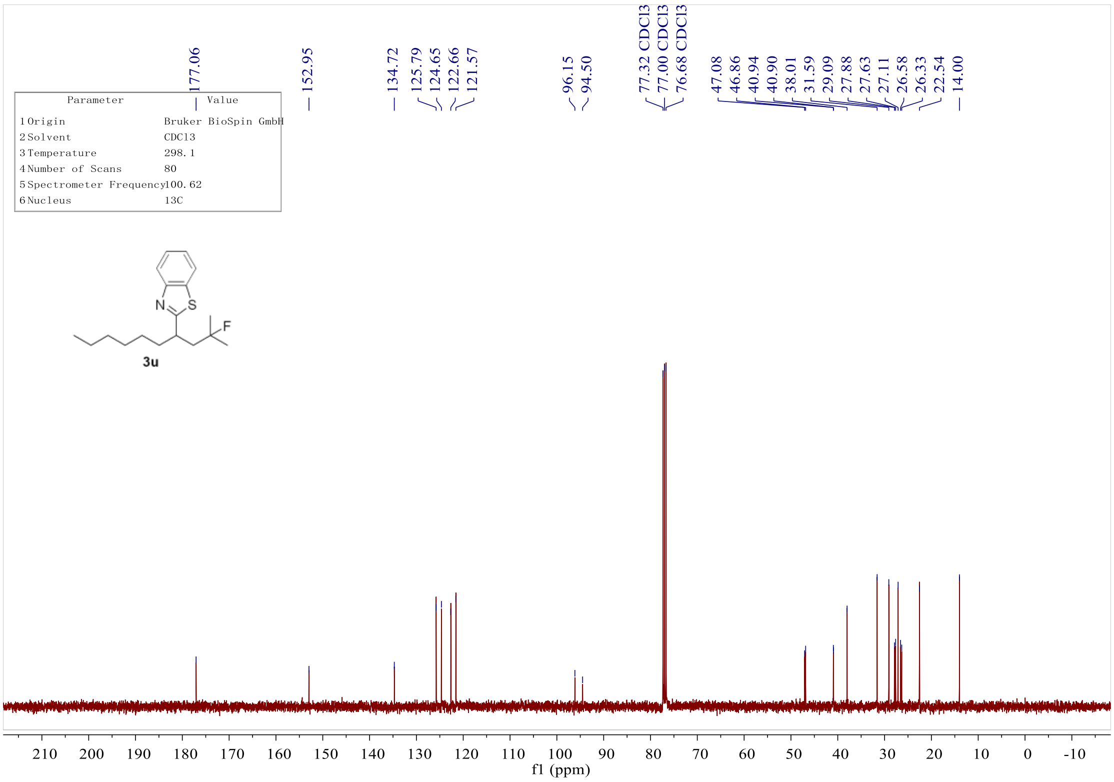

Supplementary Fig. 91 ^{13}C NMR spectra (100 MHz, $CDCl_3$, 25 °C) of **3u**

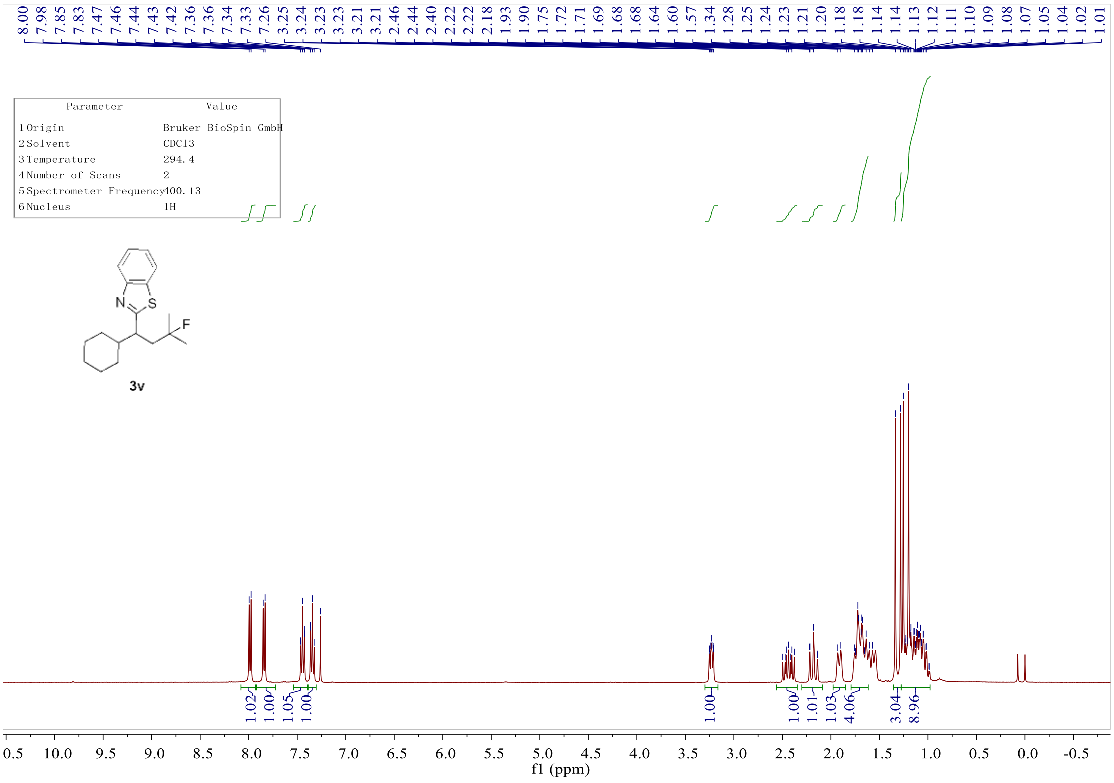

Supplementary Fig. 92 ^{1}H NMR spectra (400 MHz, $CDCl_3$, 25 °C) of **3v**

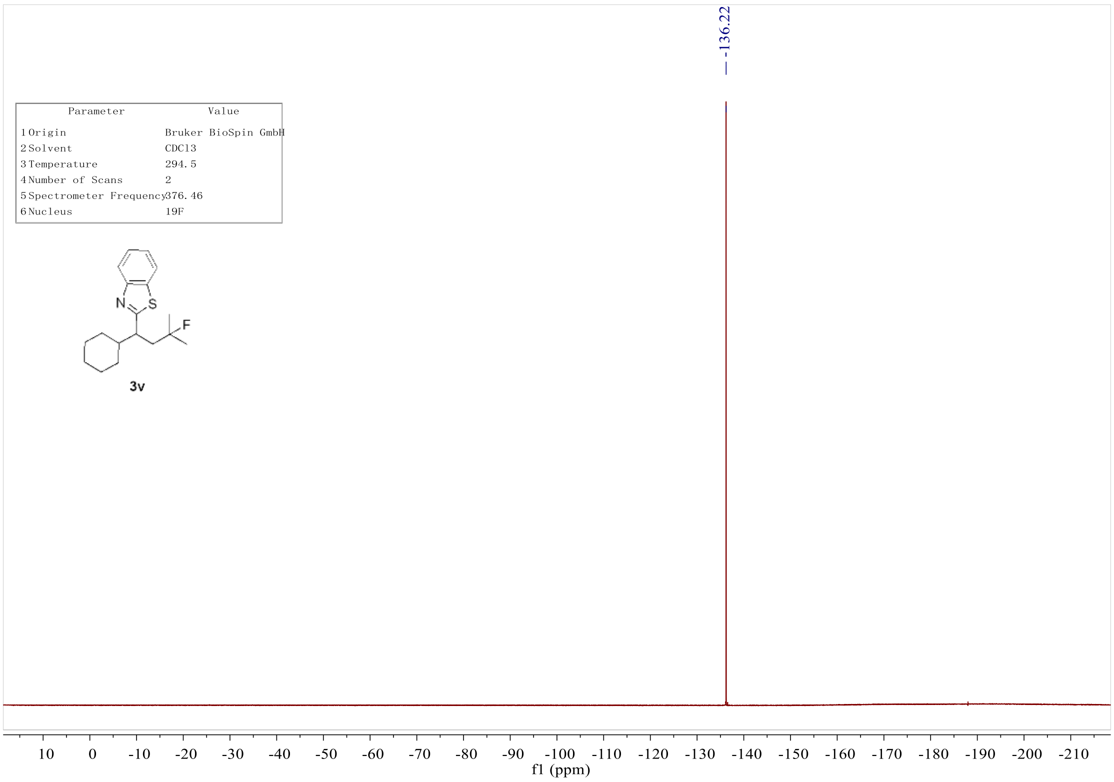

Supplementary Fig. 93 ^{19}F NMR spectra (376 MHz, $CDCl_3$, 25 °C) of **3v**

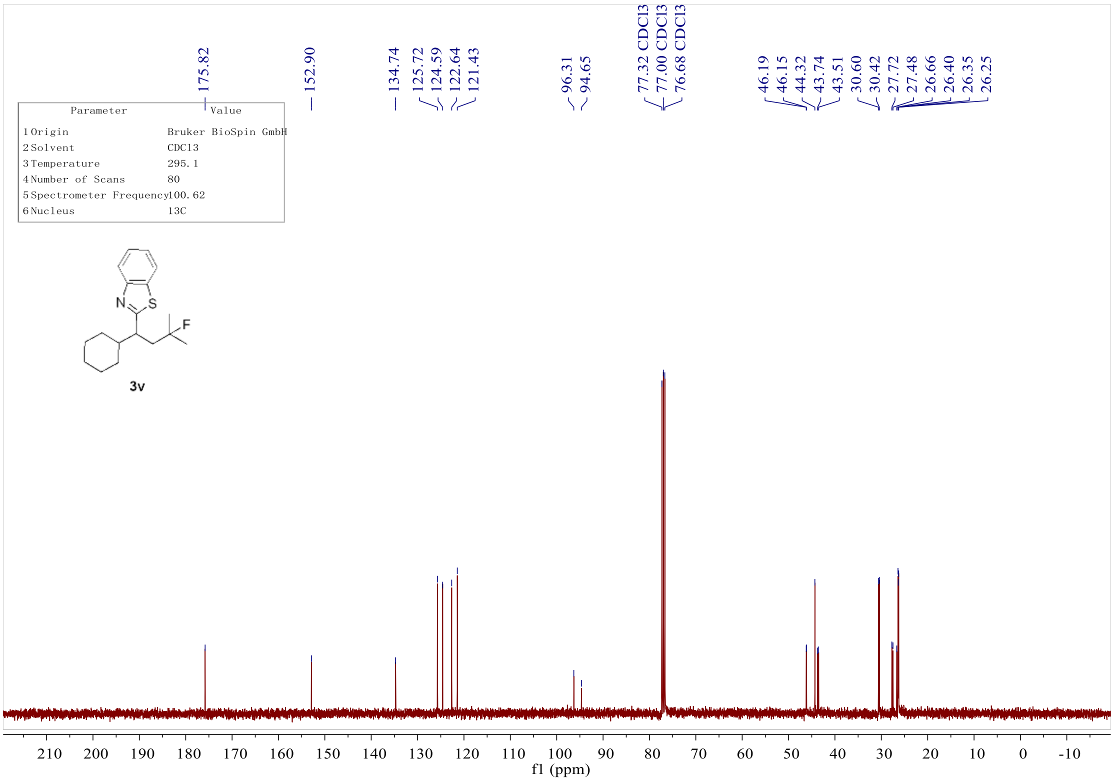

Supplementary Fig. 94 ^{13}C NMR spectra (100 MHz, $CDCl_3$, 25 ºC) of **3v**

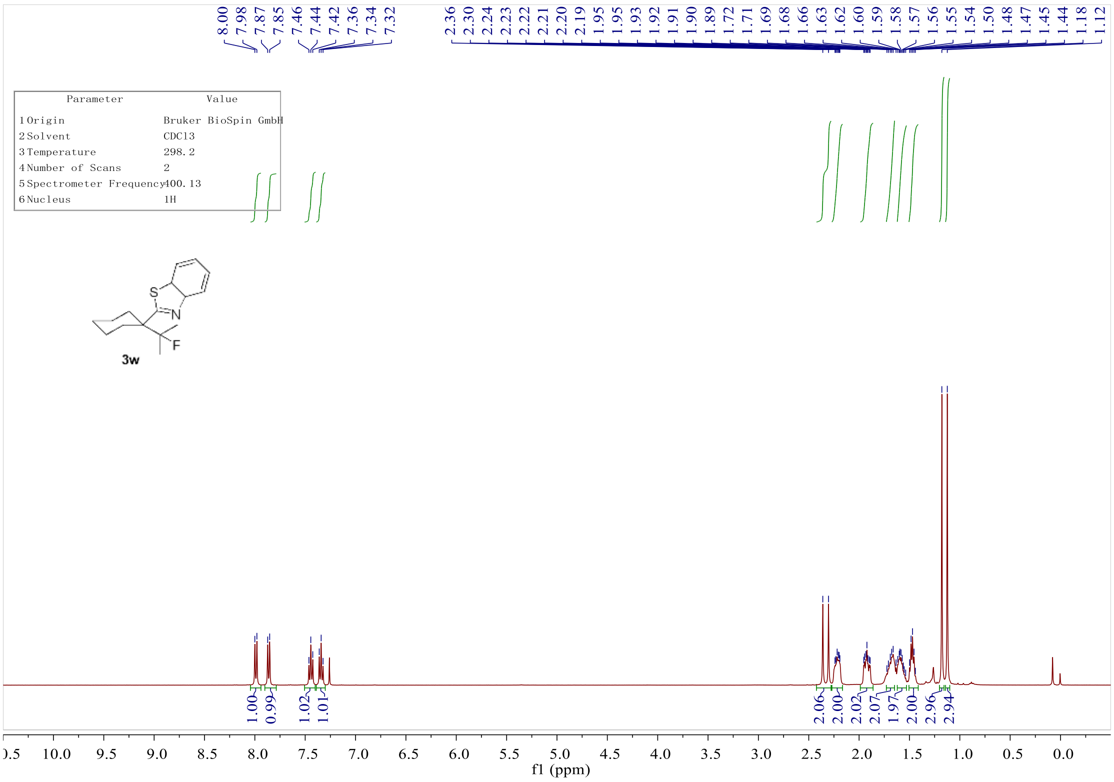

Supplementary Fig. 95 ^{1}H NMR spectra (400 MHz, $CDCl_3$, 25 ºC) of **3w**

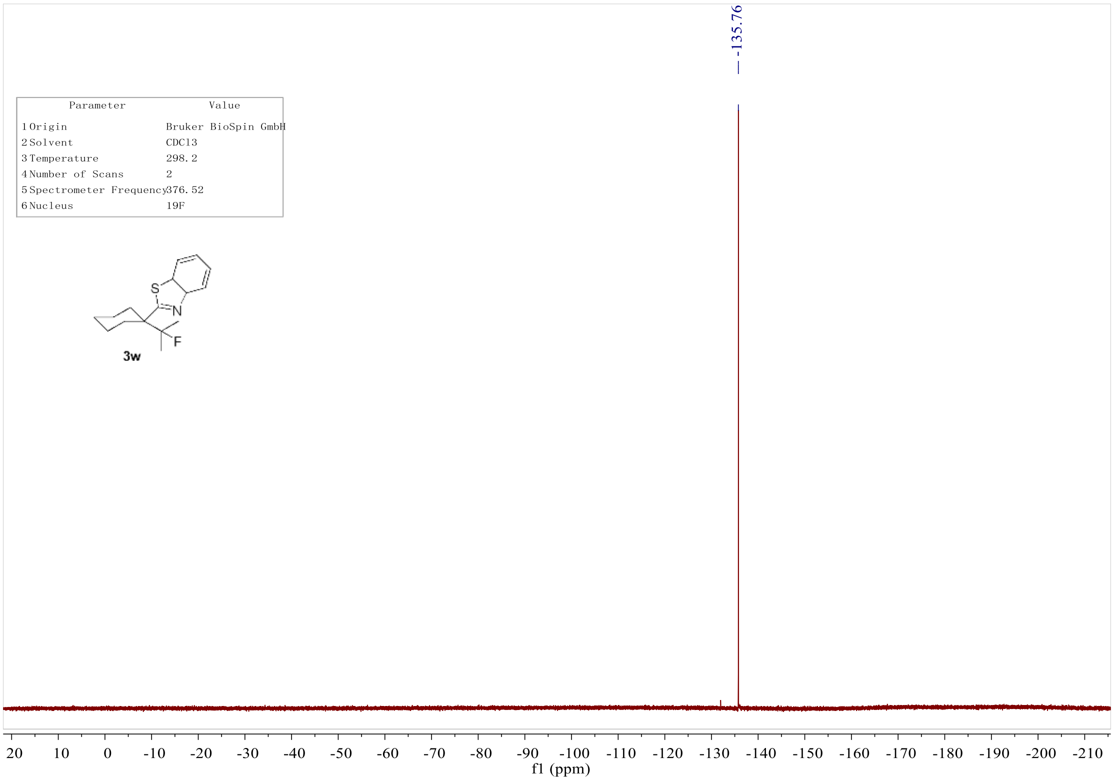

Supplementary Fig. 96 ^{19}F NMR spectra (376 MHz, $CDCl_3$, 25 ºC) of **3w**

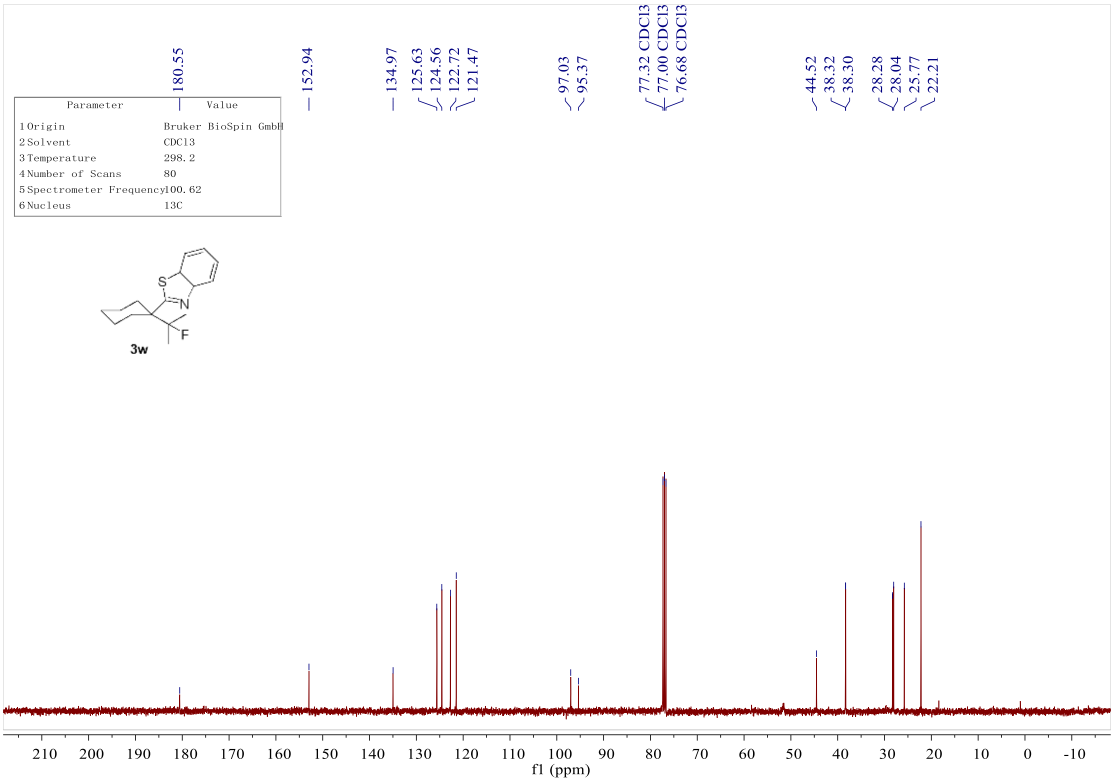

Supplementary Fig. 97 ^{13}C NMR spectra (100 MHz, $CDCl_3$, 25 ºC) of **3w**

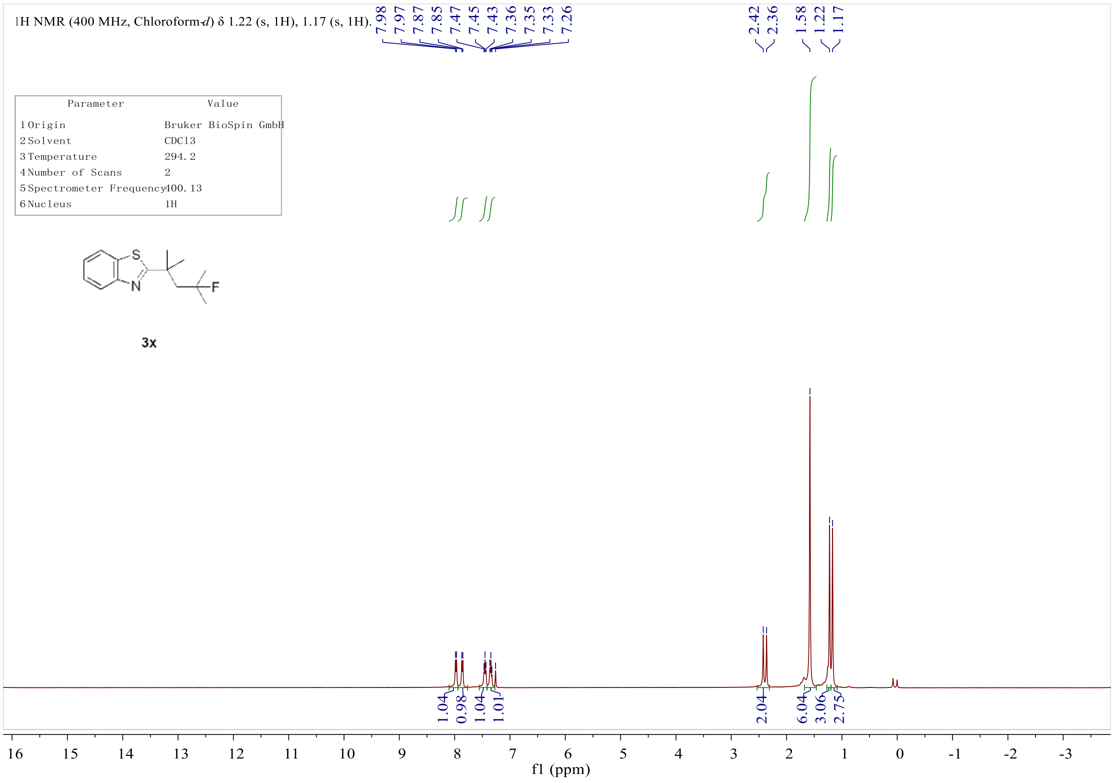

Supplementary Fig. 98 ^{1}H NMR spectra (400 MHz, $CDCl_3$, 25 °C) of **3x**

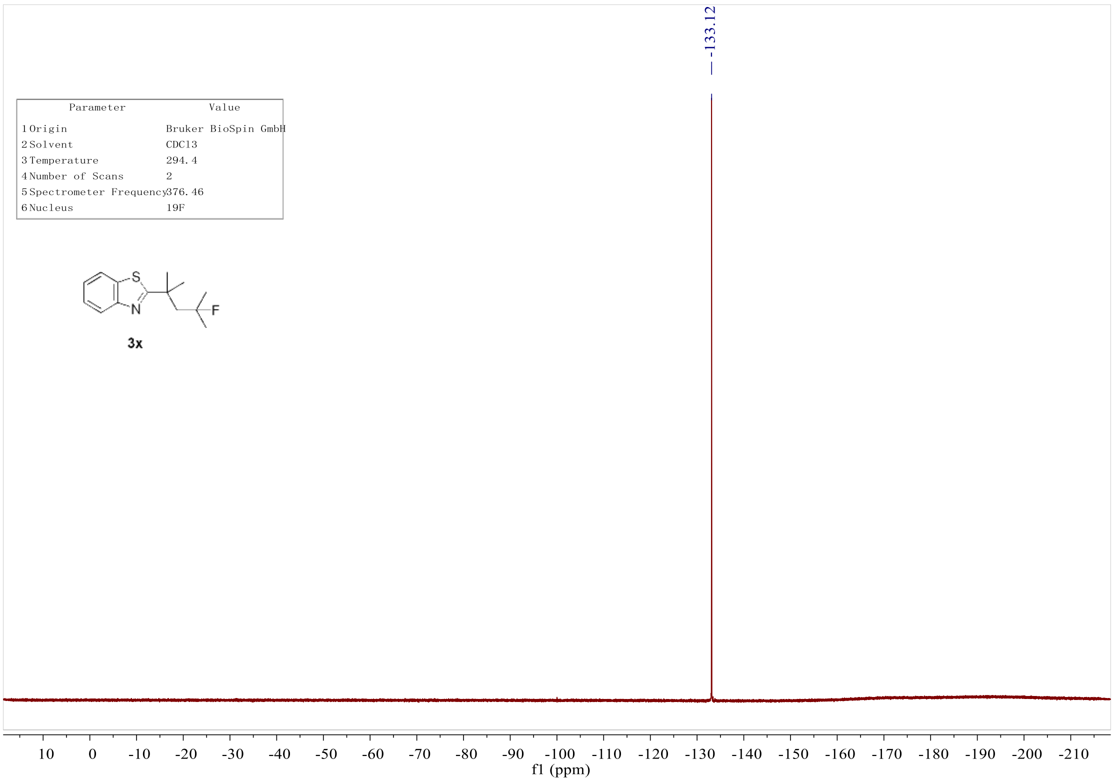

Supplementary Fig. 99 ^{19}F NMR spectra (376 MHz, $CDCl_3$, 25 °C) of **3x**

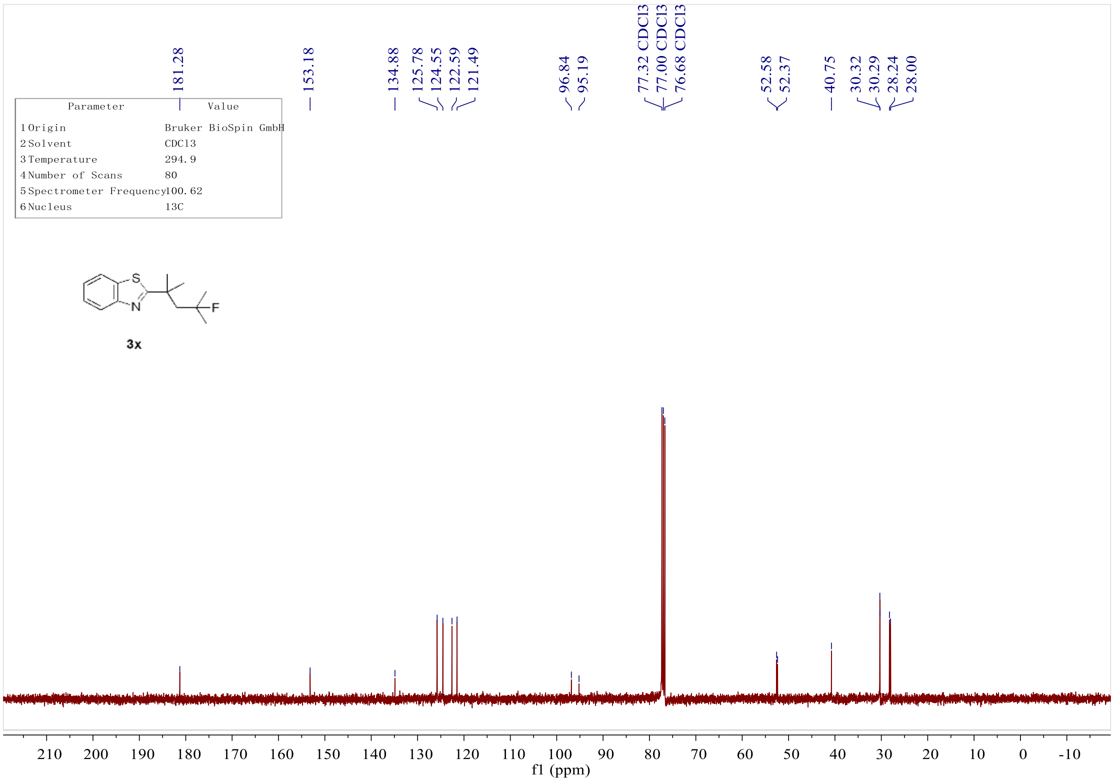

Supplementary Fig. 100 ^{13}C NMR spectra (100 MHz, $CDCl_3$, 25 ºC) of **3x**

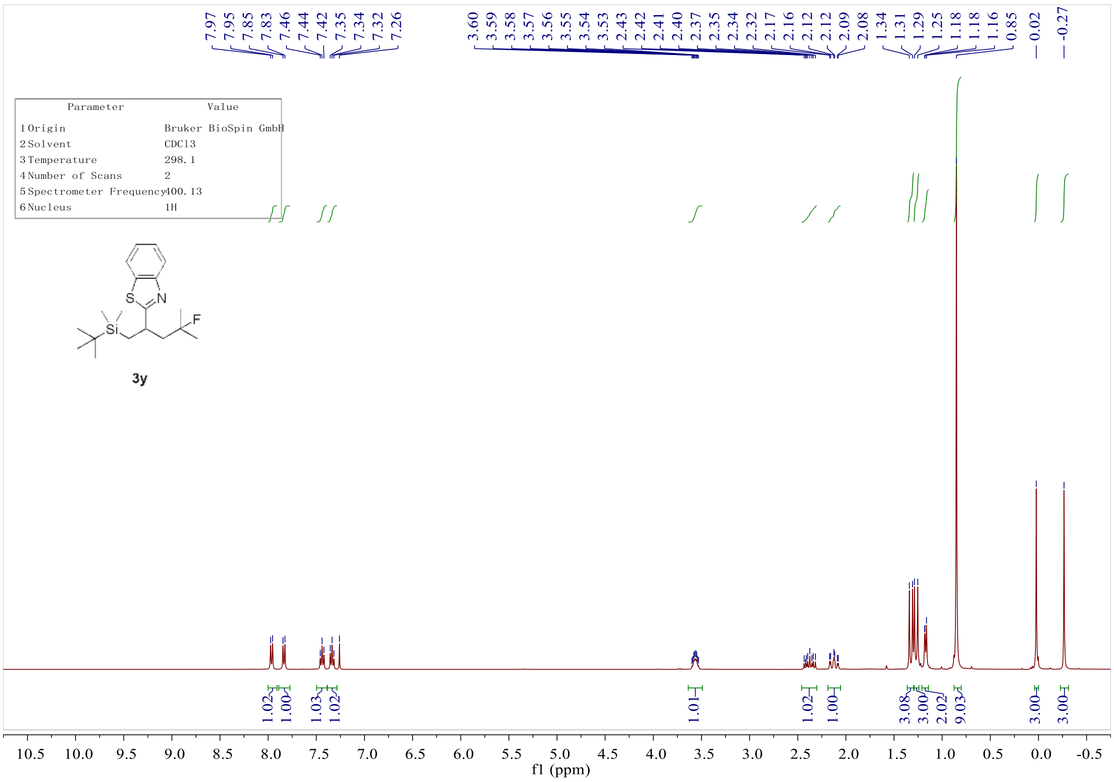

Supplementary Fig. 101 ^{1}H NMR spectra (400 MHz, $CDCl_3$, 25 ºC) of **3y**

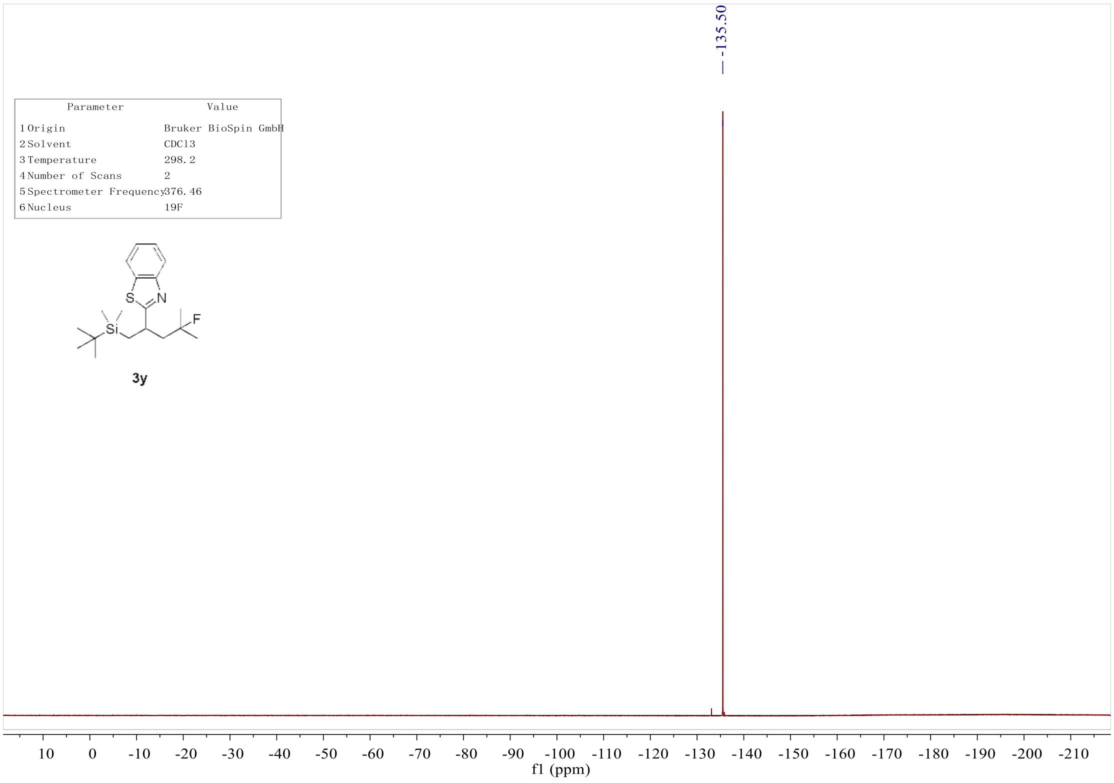

Supplementary Fig. 102 ^{19}F NMR spectra (376 MHz, $CDCl_3$, 25 °C) of **3y**

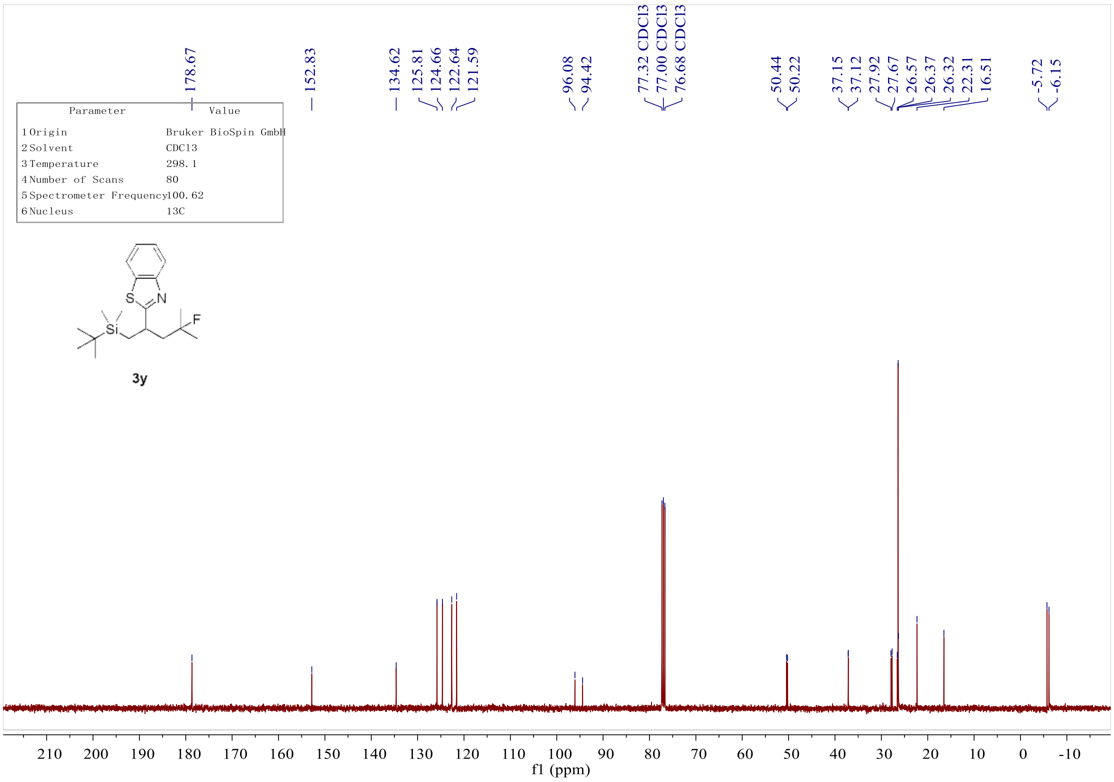

Supplementary Fig. 103 ^{13}C NMR spectra (100 MHz, $CDCl_3$, 25 °C) of **3y**

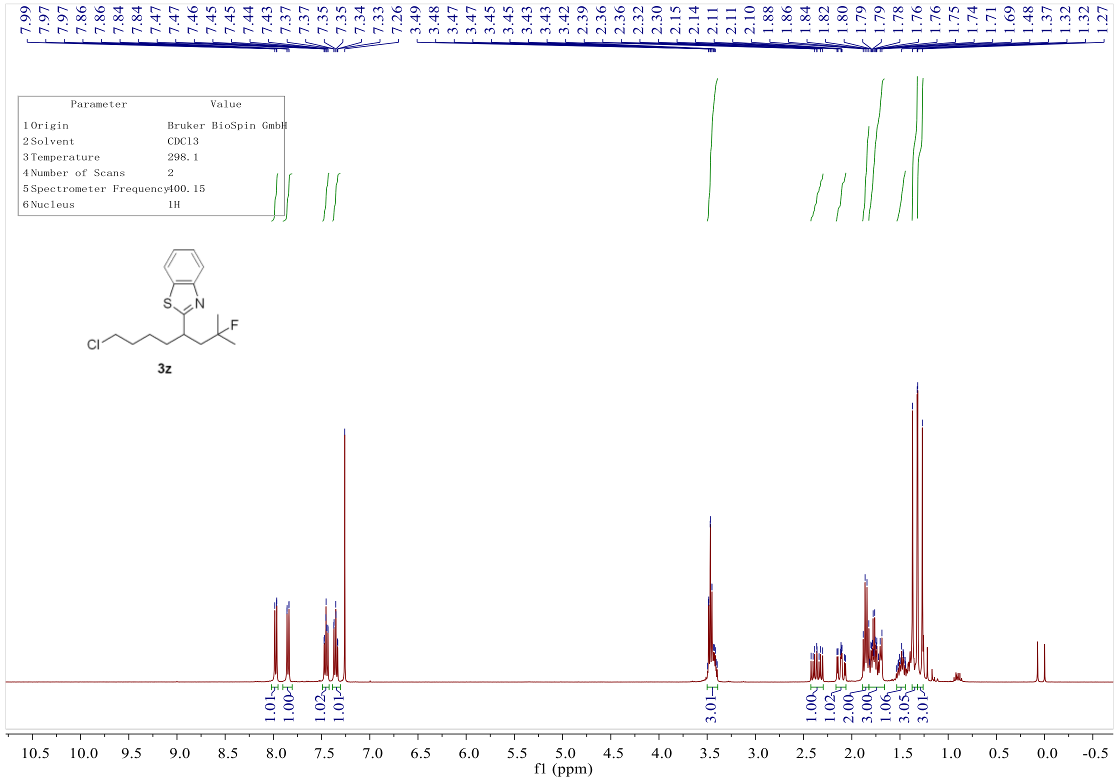

Supplementary Fig. 104 ^{1}H NMR spectra (400 MHz, $CDCl_3$, 25 ºC) of **3z**

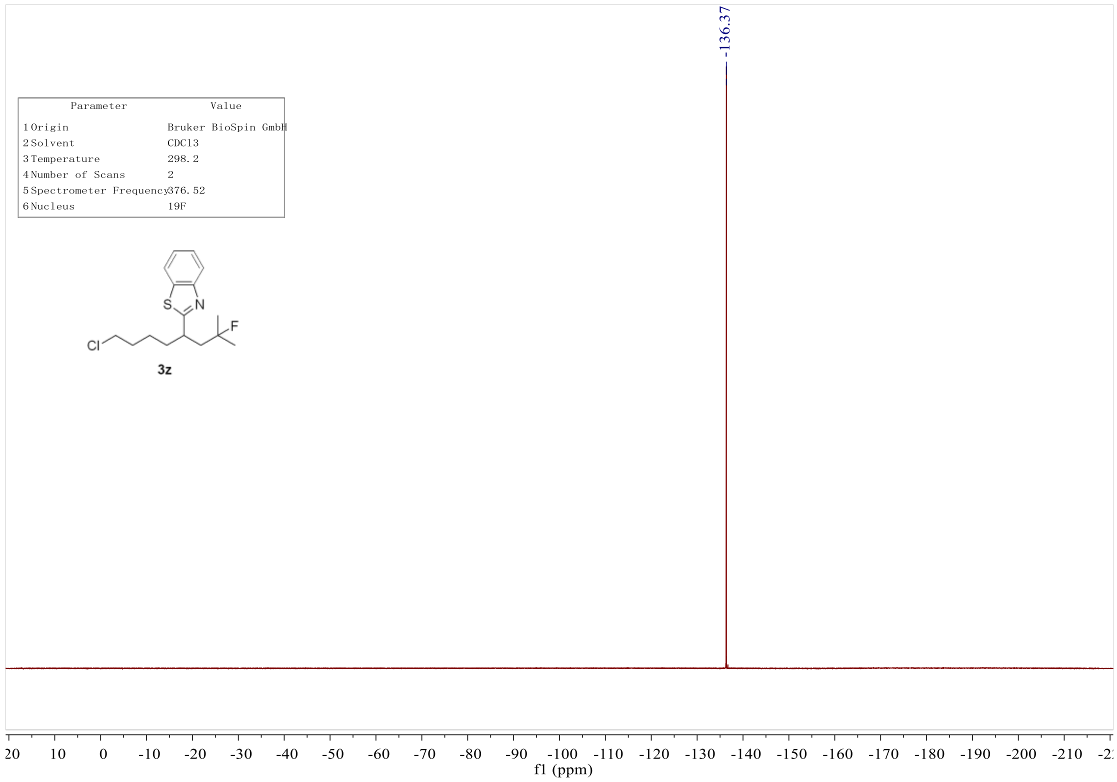

Supplementary Fig. 105 ^{19}F NMR spectra (376 MHz, $CDCl_3$, 25 ºC) of **3z**

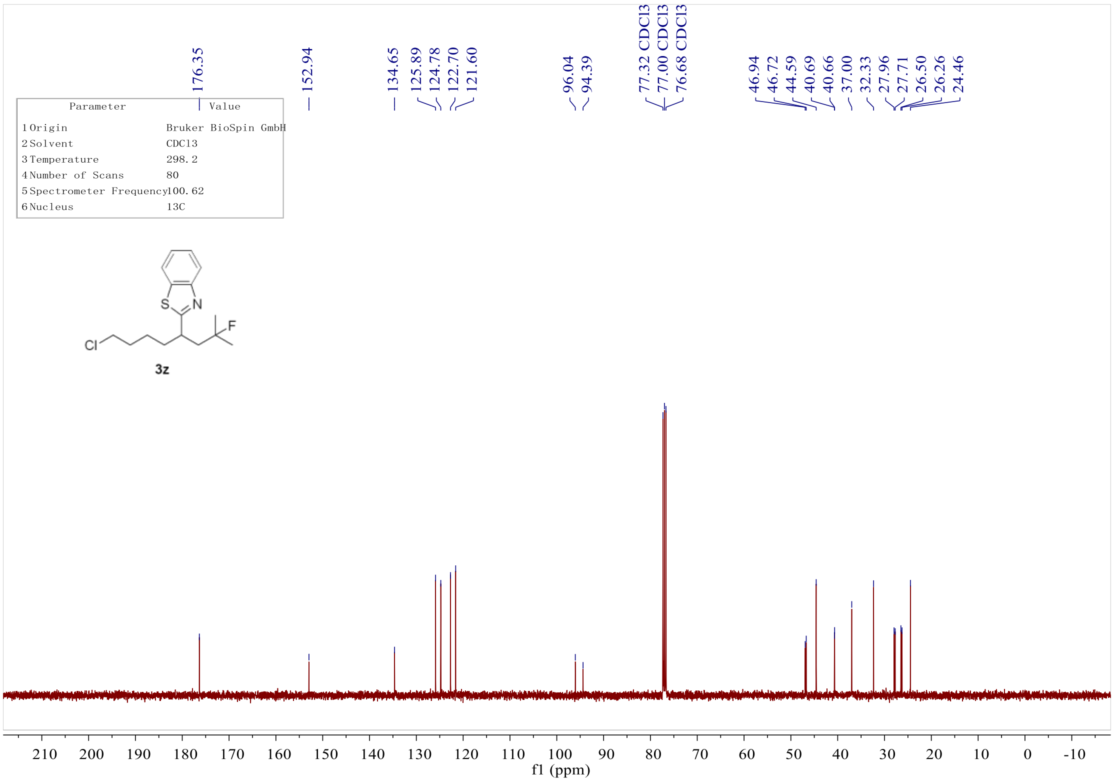

Supplementary Fig. 106 ^{13}C NMR spectra (100 MHz, $CDCl_3$, 25 ºC) of **3z**

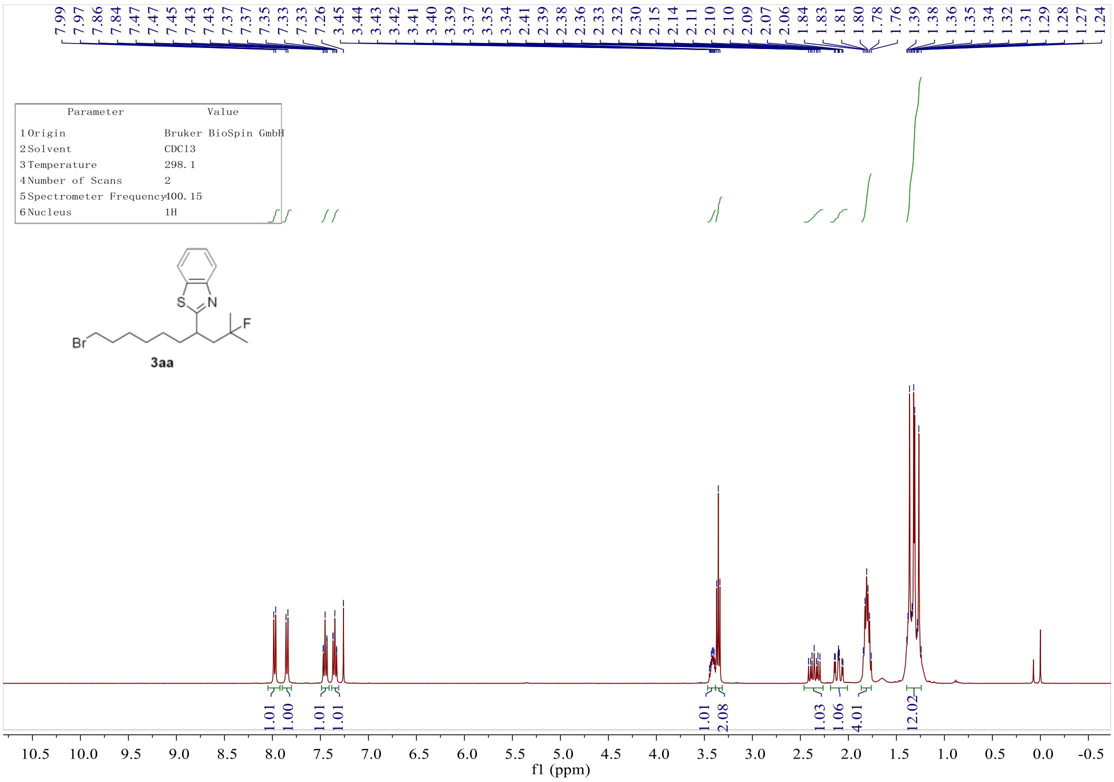

Supplementary Fig. 107 ^{1}H NMR spectra (400 MHz, $CDCl_3$, 25 ºC) of **3aa**

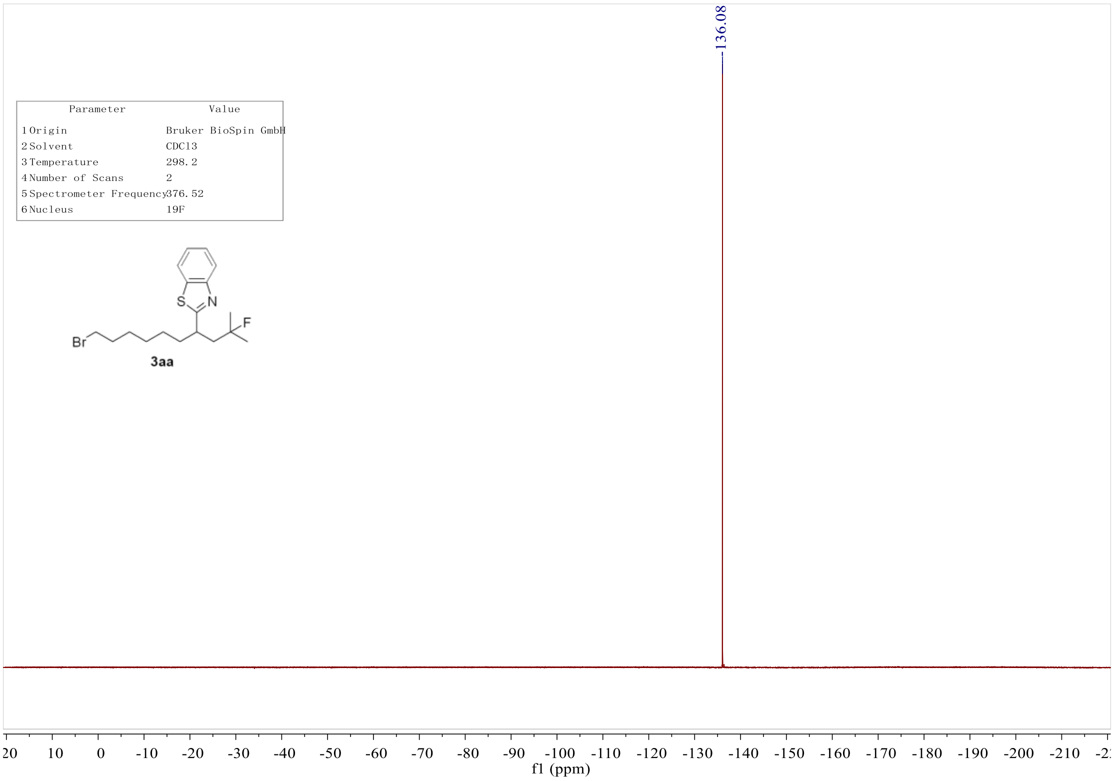

Supplementary Fig. 108 ^{19}F NMR spectra (376 MHz, $CDCl_3$, 25 °C) of **3aa**

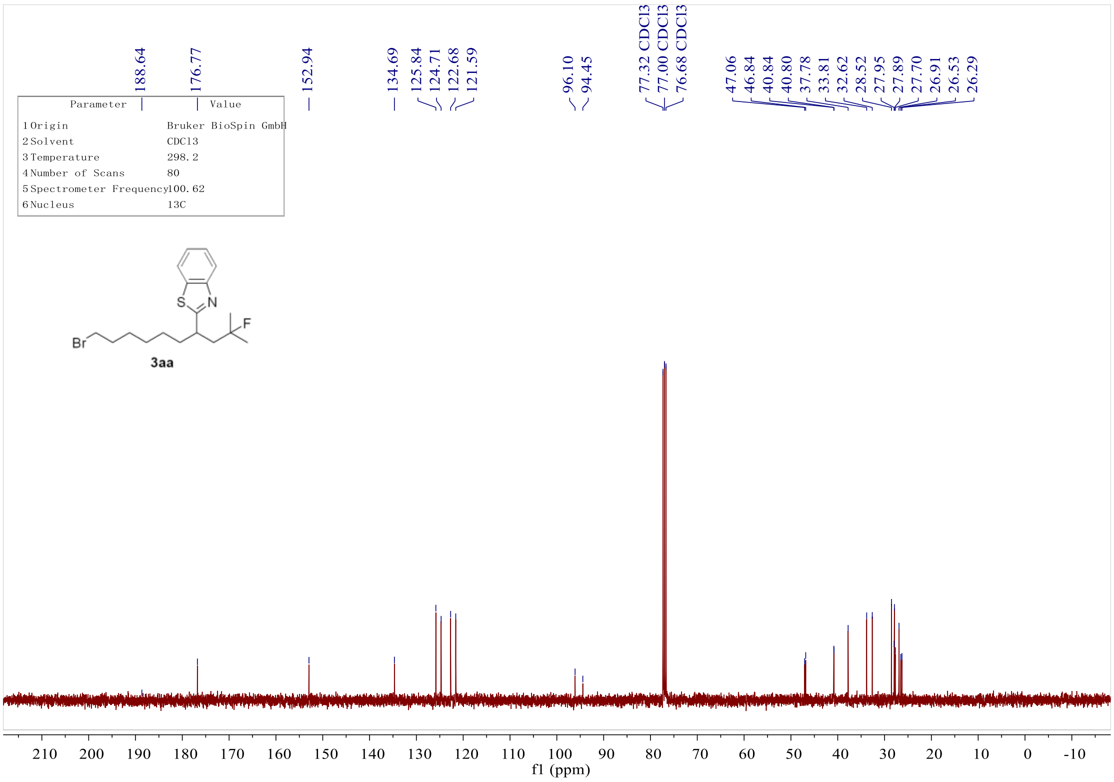

Supplementary Fig. 109 ^{13}C NMR spectra (100 MHz, $CDCl_3$, 25 °C) of **3aa**

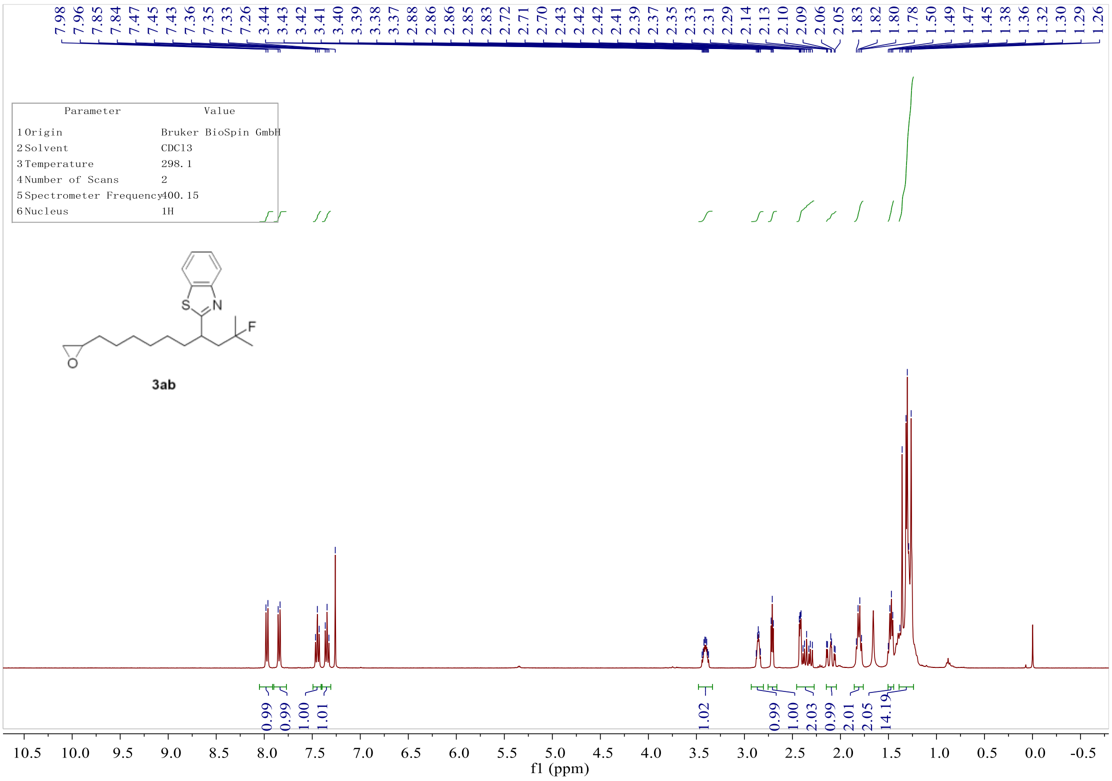

Supplementary Fig. 110 ^{1}H NMR spectra (400 MHz, $CDCl_3$, 25 °C) of **3ab**

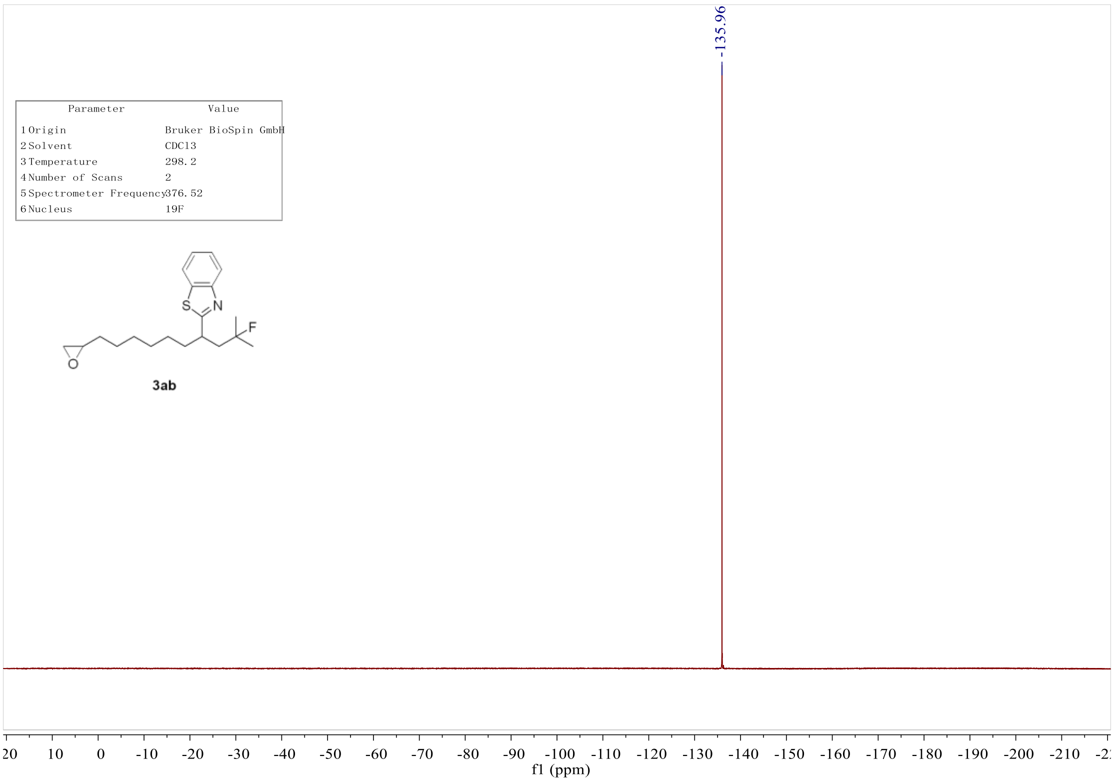

Supplementary Fig. 111 ^{19}F NMR spectra (376 MHz, $CDCl_3$, 25 °C) of **3ab**

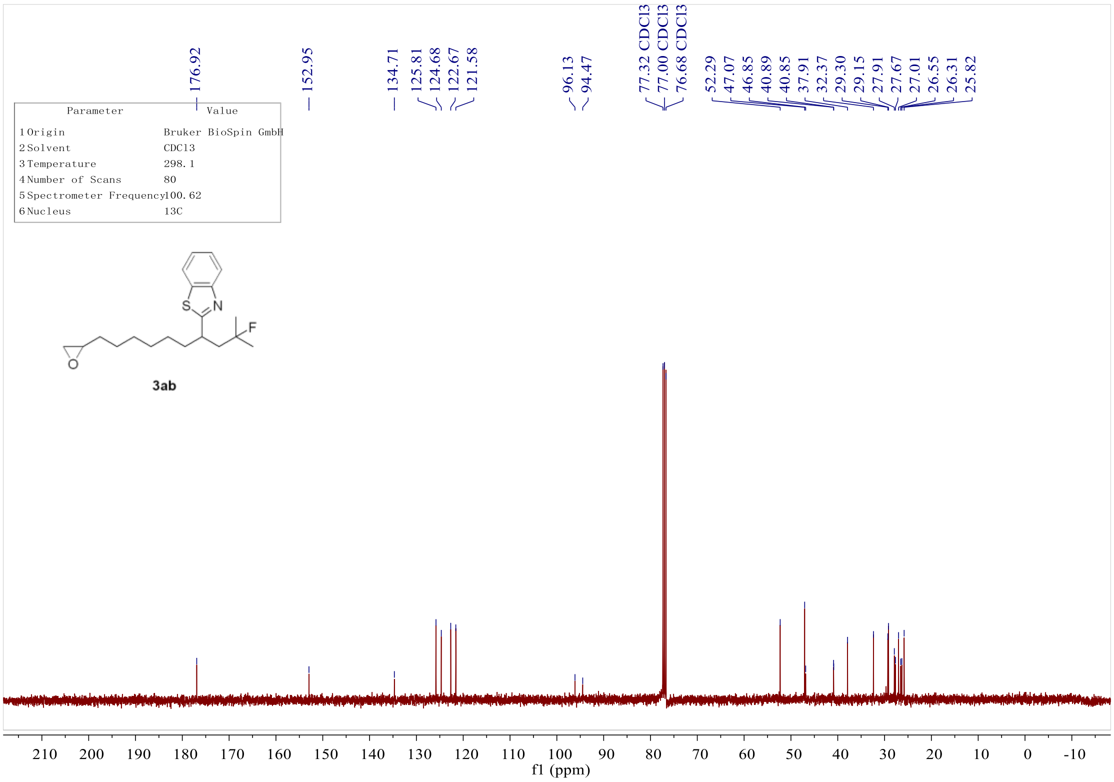

Supplementary Fig. 112 ^{13}C NMR spectra (100 MHz, $CDCl_3$, 25 °C) of **3ab**

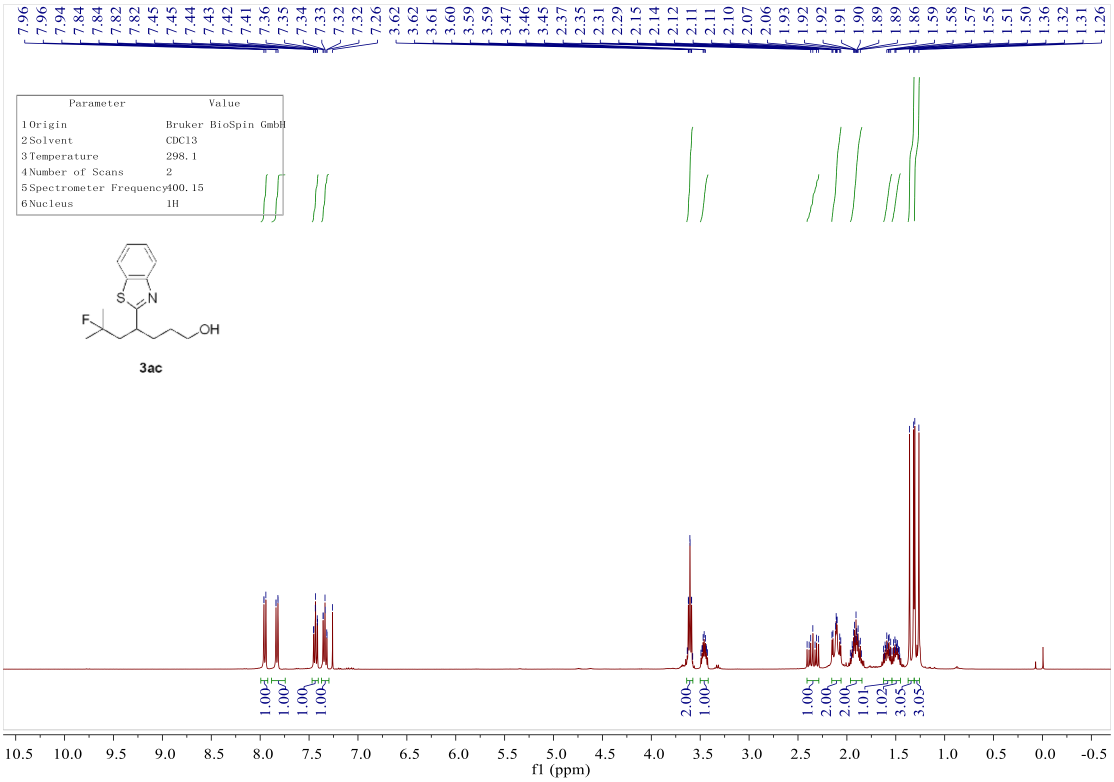

Supplementary Fig. 113 ^{1}H NMR spectra (400 MHz, $CDCl_3$, 25 °C) of **3ac**

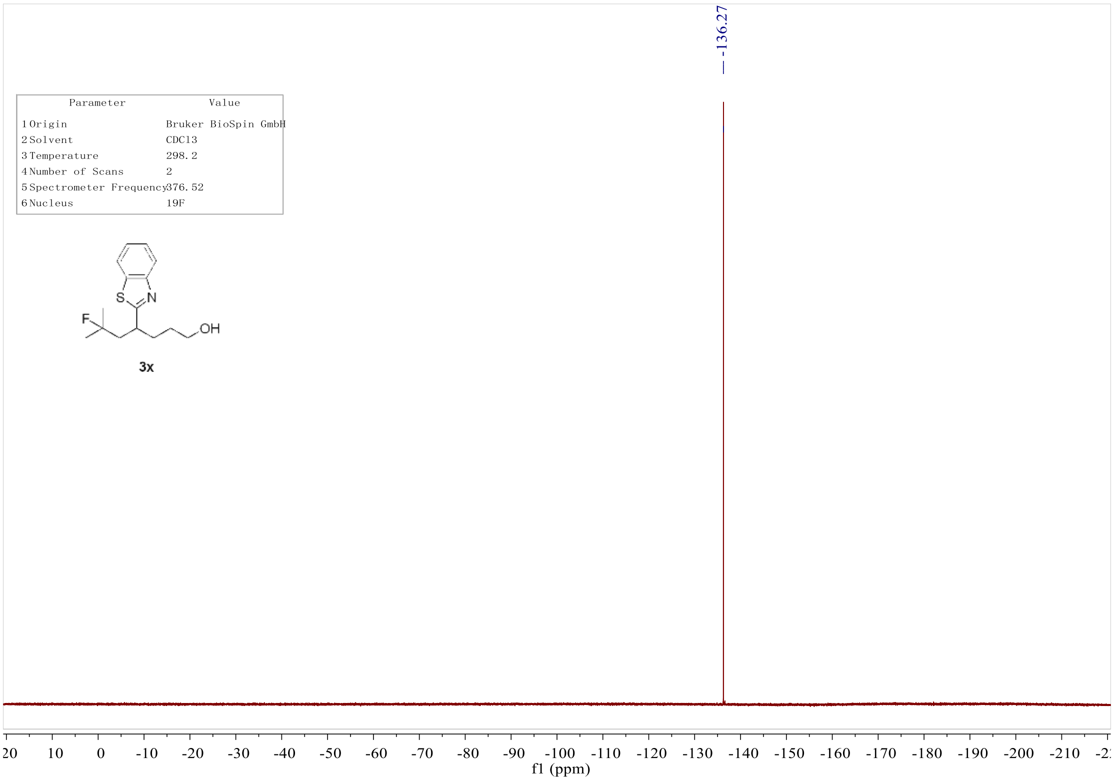

Supplementary Fig. 114 ^{19}F NMR spectra (376 MHz, $CDCl_3$, 25 °C) of **3ac**

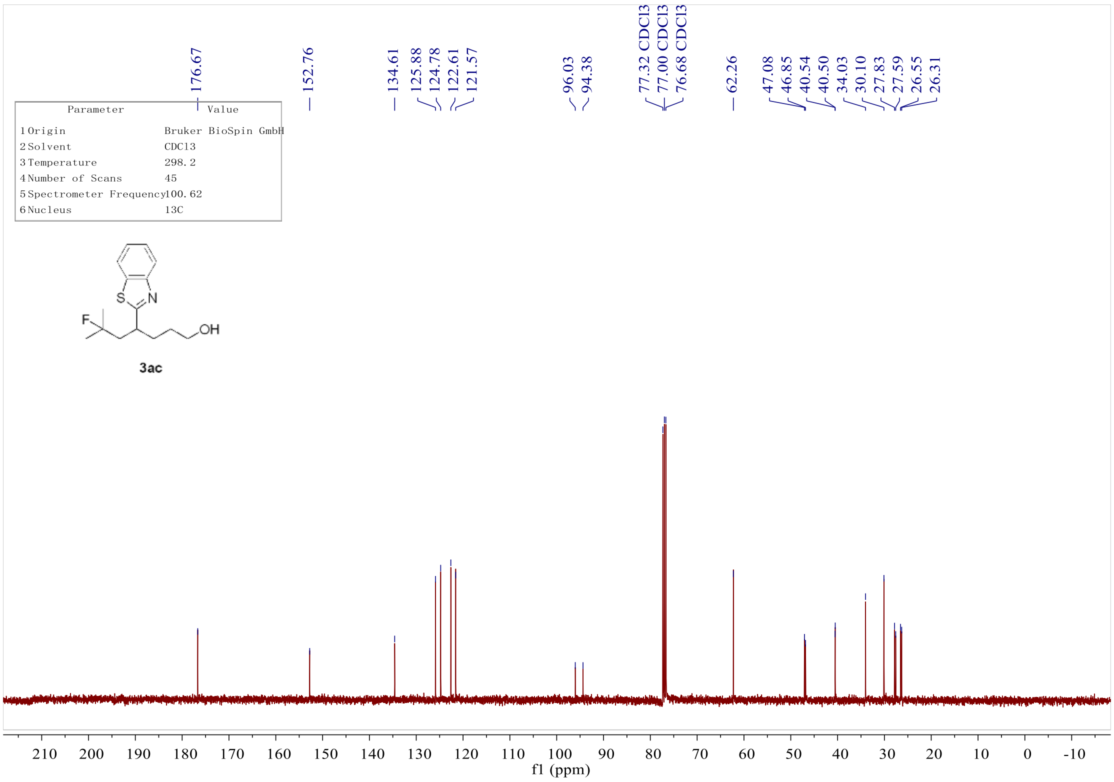

Supplementary Fig. 115 ^{13}C NMR spectra (100 MHz, $CDCl_3$, 25 °C) of **3ac**

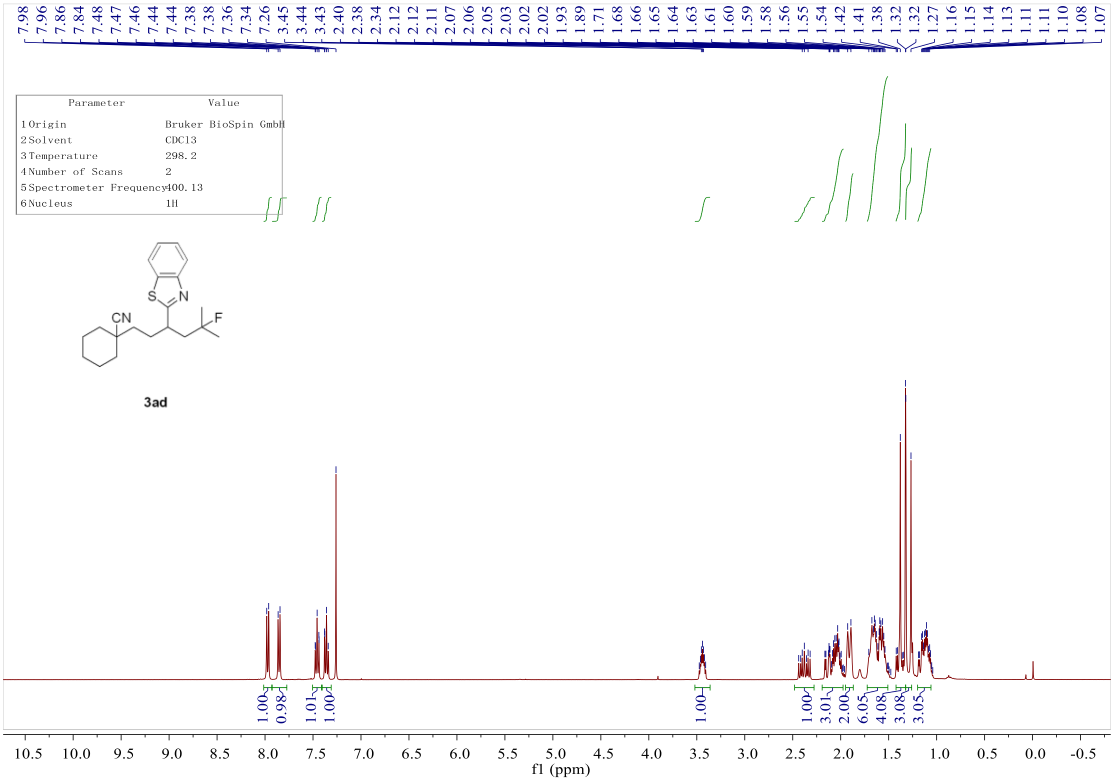

Supplementary Fig. 116 ^{1}H NMR spectra (400 MHz, $CDCl_3$, 25 °C) of **3ad**

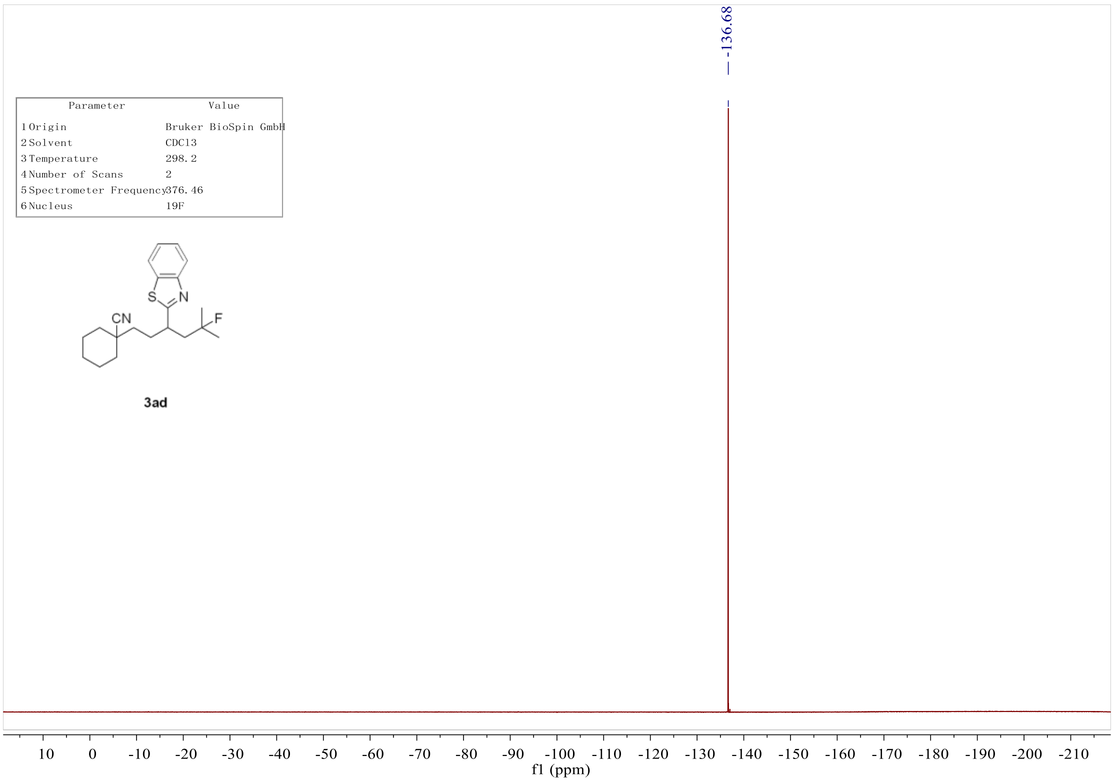

Supplementary Fig. 117 ^{19}F NMR spectra (376 MHz, $CDCl_3$, 25 °C) of **3ad**

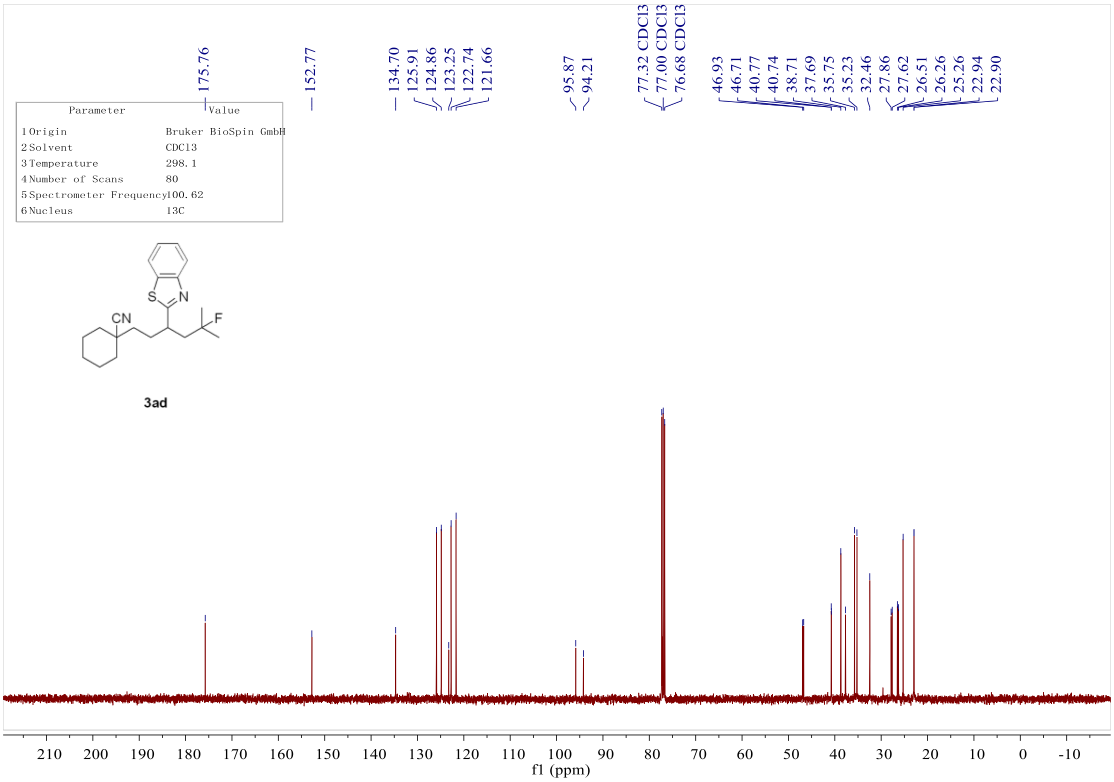

Supplementary Fig. 118 ^{13}C NMR spectra (100 MHz, $CDCl_3$, 25 °C) of **3ad**

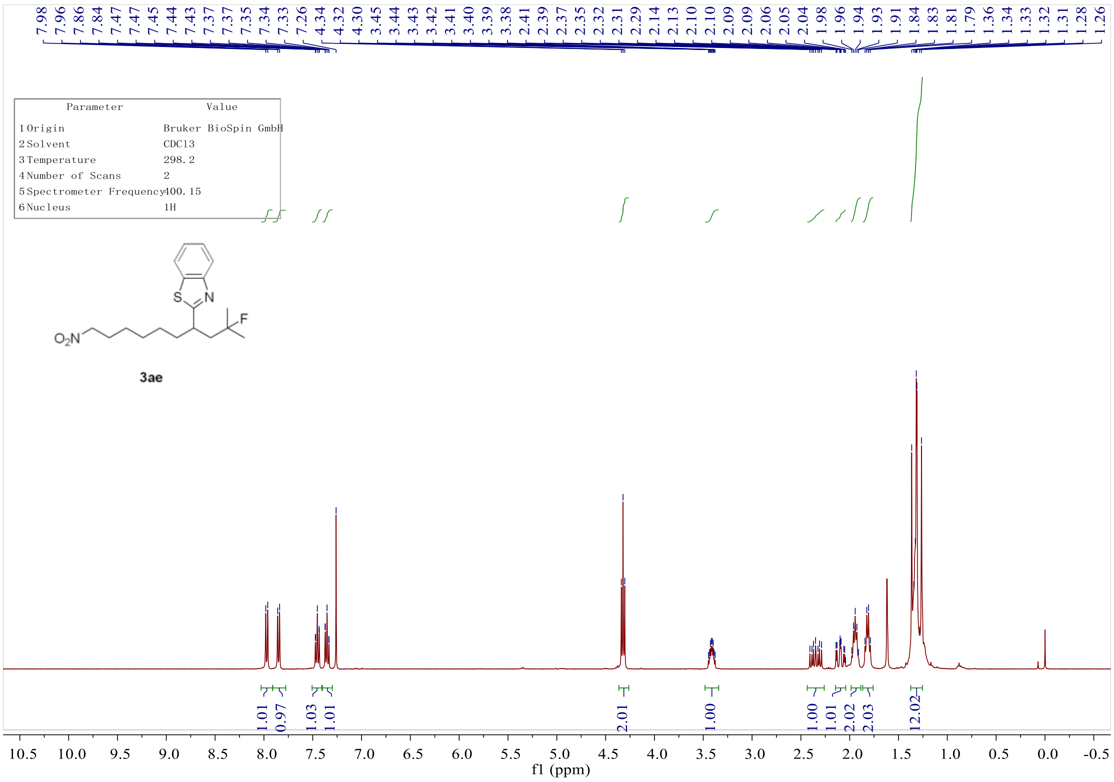

Supplementary Fig. 119 ^{1}H NMR spectra (400 MHz, $CDCl_3$, 25 °C) of **3ae**

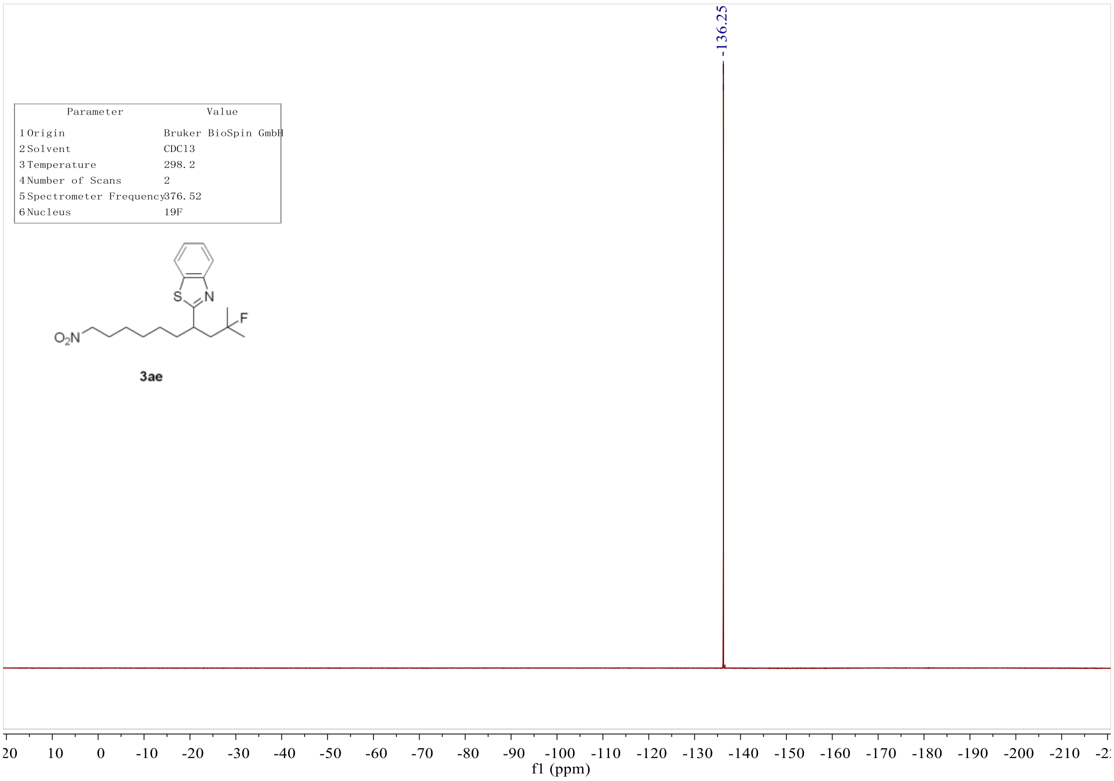

Supplementary Fig. 120 ^{19}F NMR spectra (376 MHz, $CDCl_3$, 25 ºC) of **3ae**

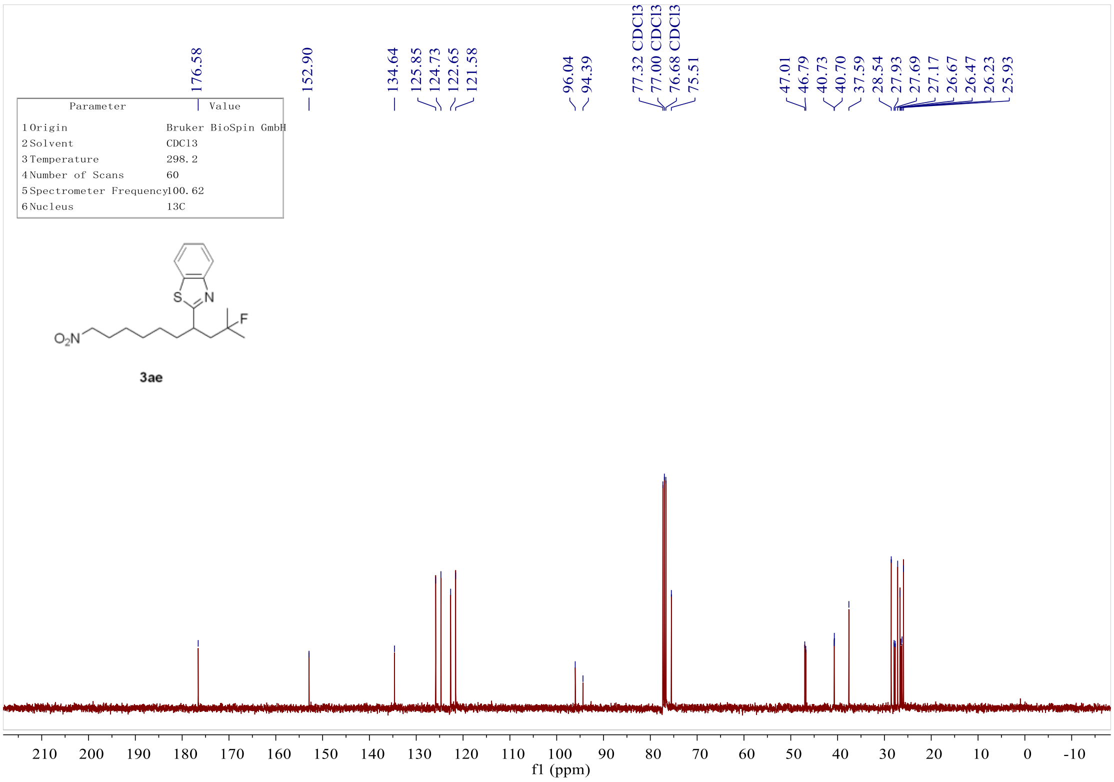

Supplementary Fig. 121 ^{13}C NMR spectra (100 MHz, $CDCl_3$, 25 ºC) of **3ae**

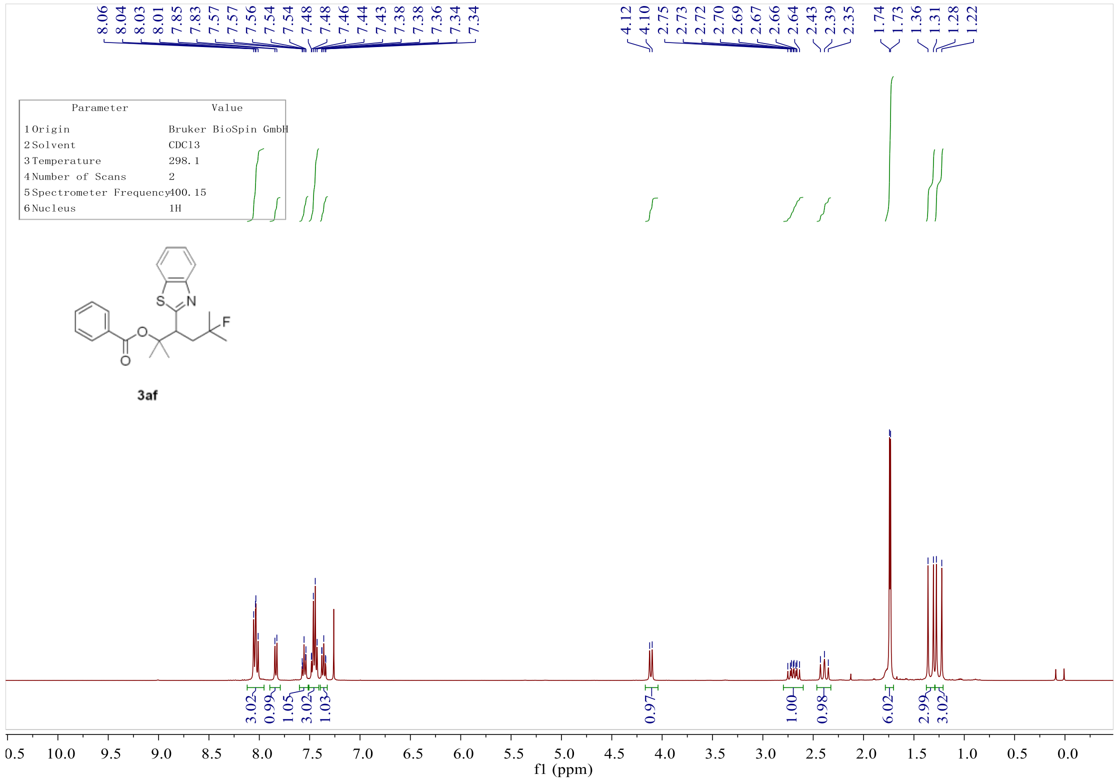

Supplementary Fig. 122 ^{1}H NMR spectra (400 MHz, $CDCl_3$, 25 °C) of **3af**

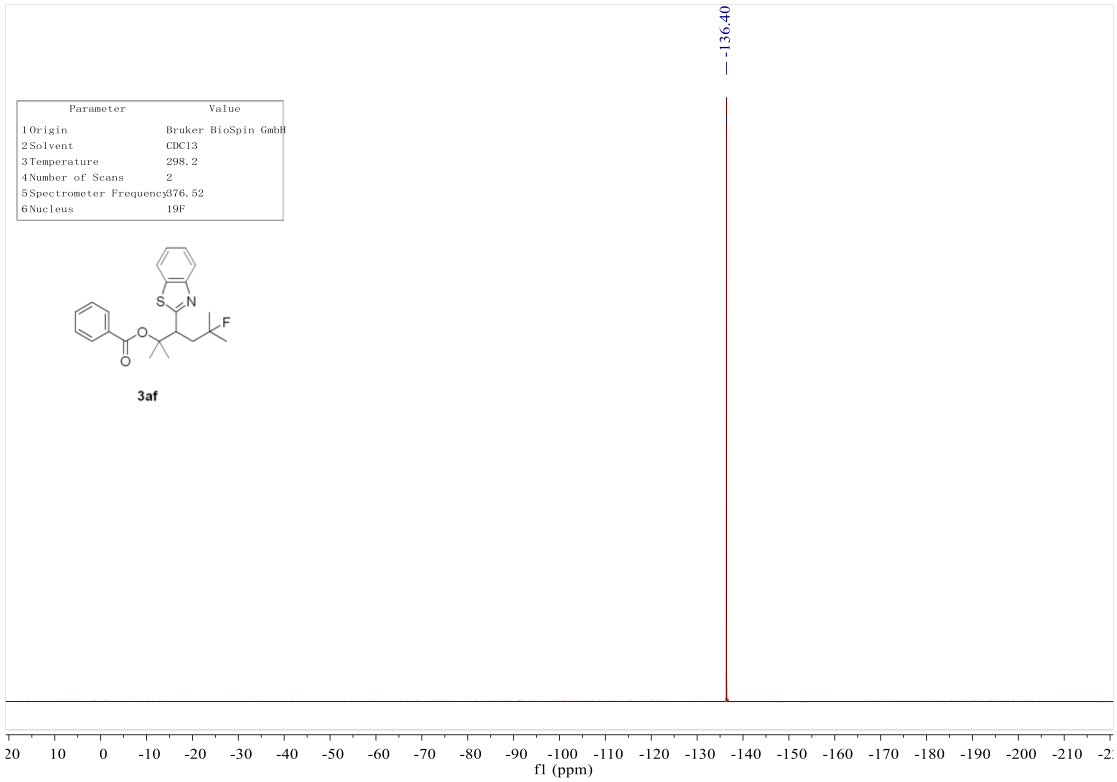

Supplementary Fig. 123 ^{19}F NMR spectra (376 MHz, $CDCl_3$, 25 °C) of **3af**

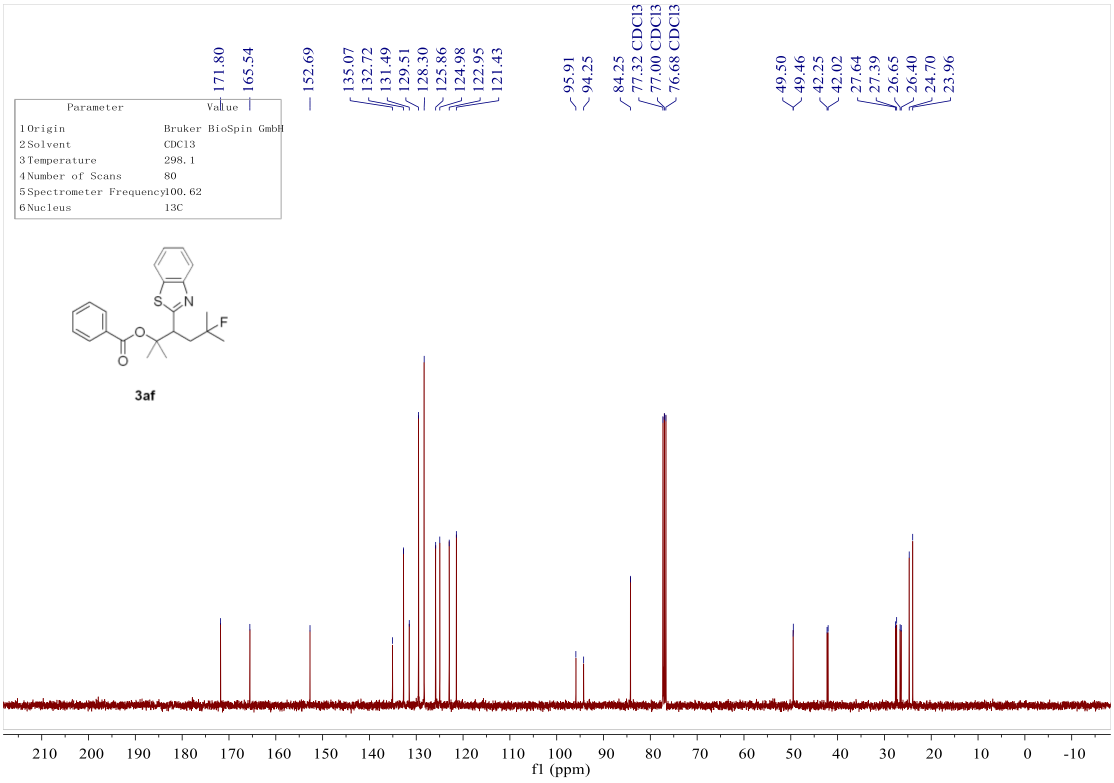

Supplementary Fig. 124 ^{13}C NMR spectra (100 MHz, $CDCl_3$, 25 °C) of **3af**

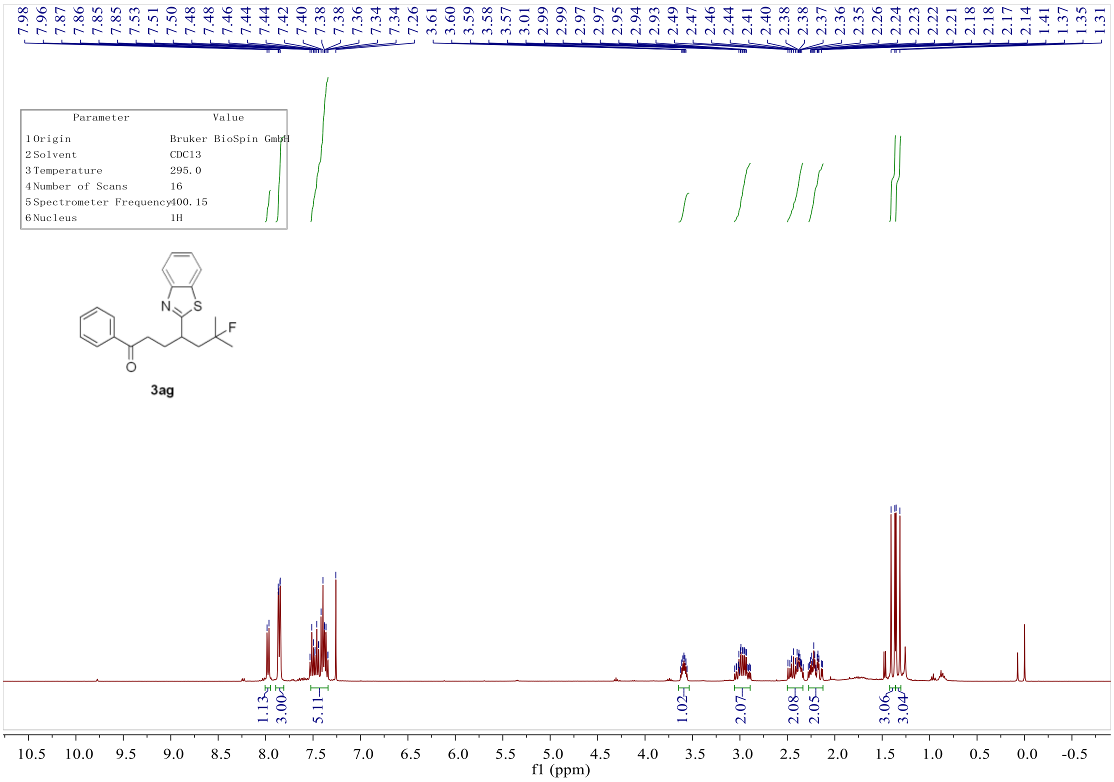

Supplementary Fig. 125 ^{1}H NMR spectra (400 MHz, $CDCl_3$, 25 °C) of **3ag**

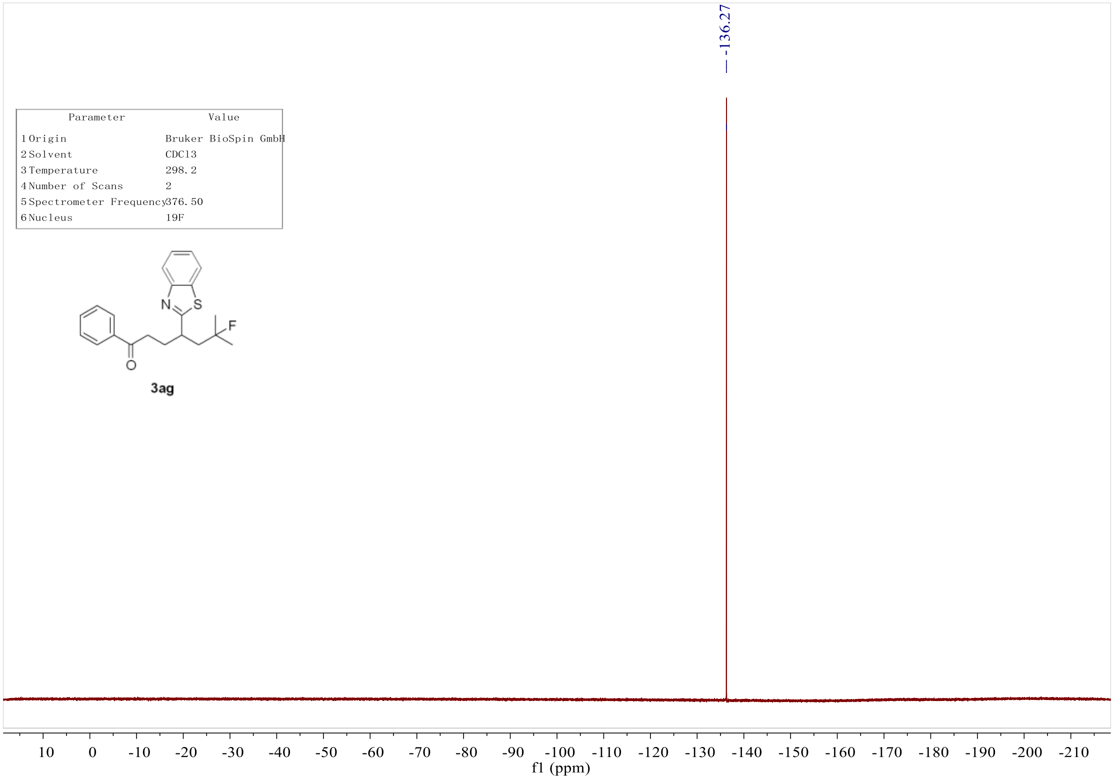

Supplementary Fig. 126 ^{19}F NMR spectra (376 MHz, $CDCl_3$, 25 ºC) of **3ag**

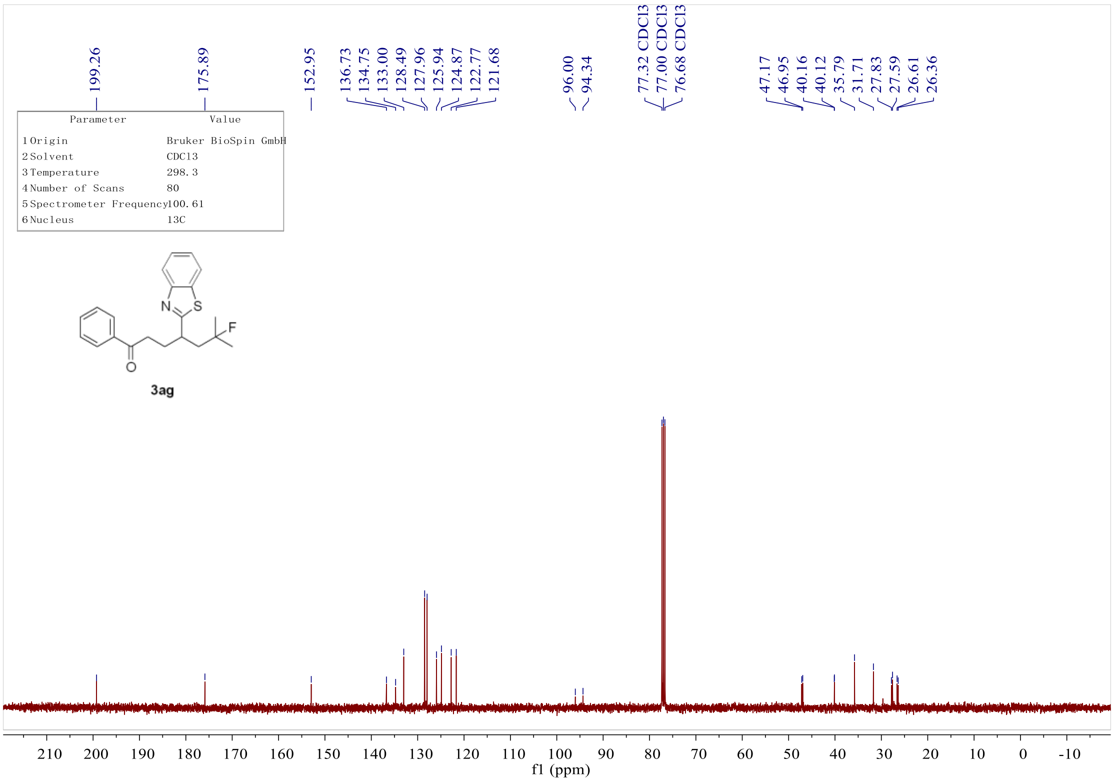

Supplementary Fig. 127 ^{13}C NMR spectra (100 MHz, $CDCl_3$, 25 ºC) of **3ag**

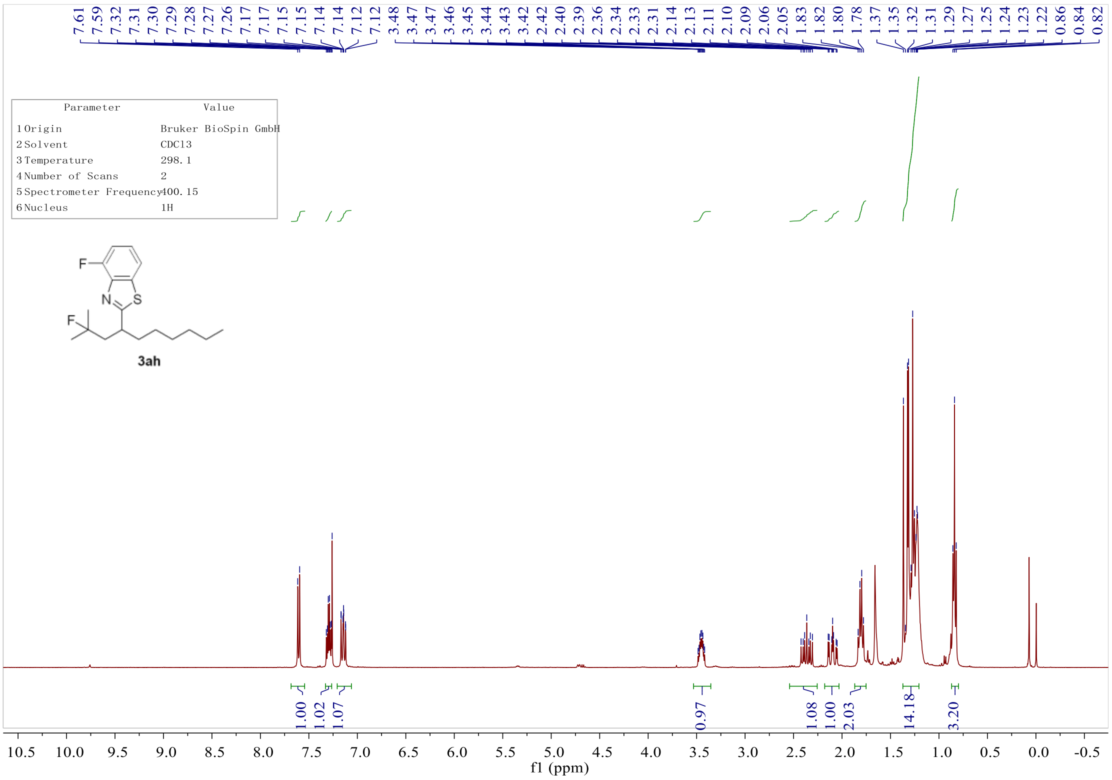

Supplementary Fig. 128 ^{1}H NMR spectra (400 MHz, $CDCl_3$, 25 °C) of **3ah**

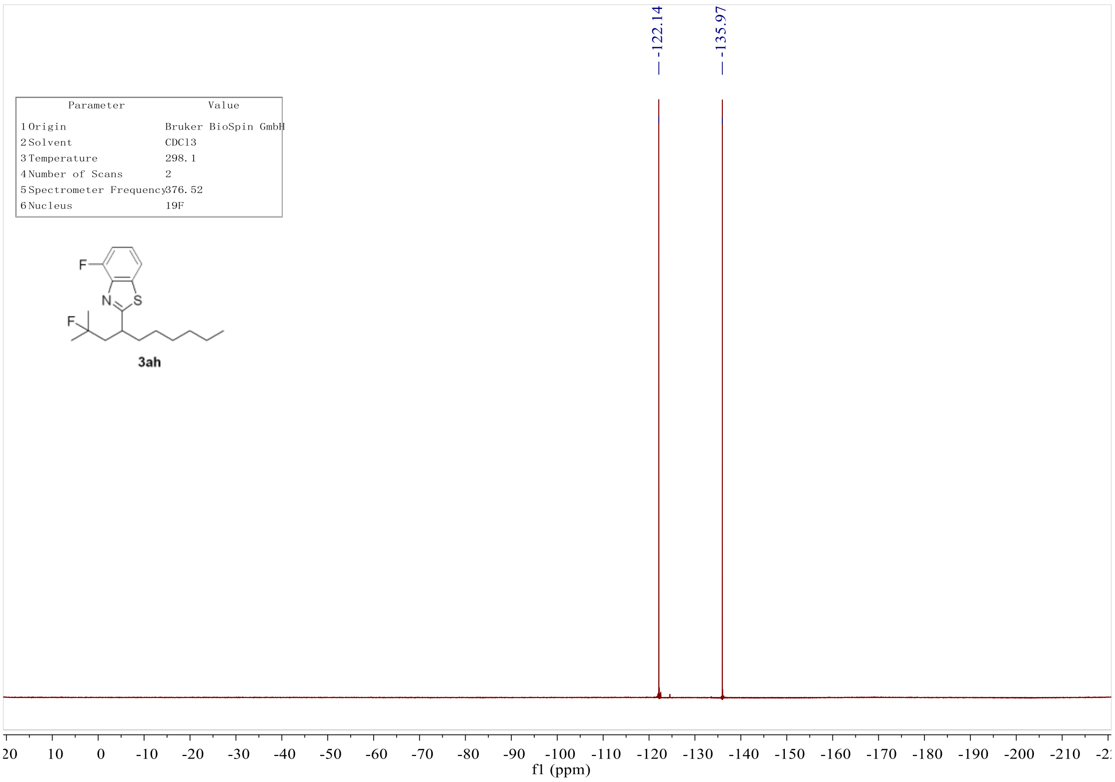

Supplementary Fig. 129 ^{19}F NMR spectra (376 MHz, $CDCl_3$, 25 °C) of **3ah**

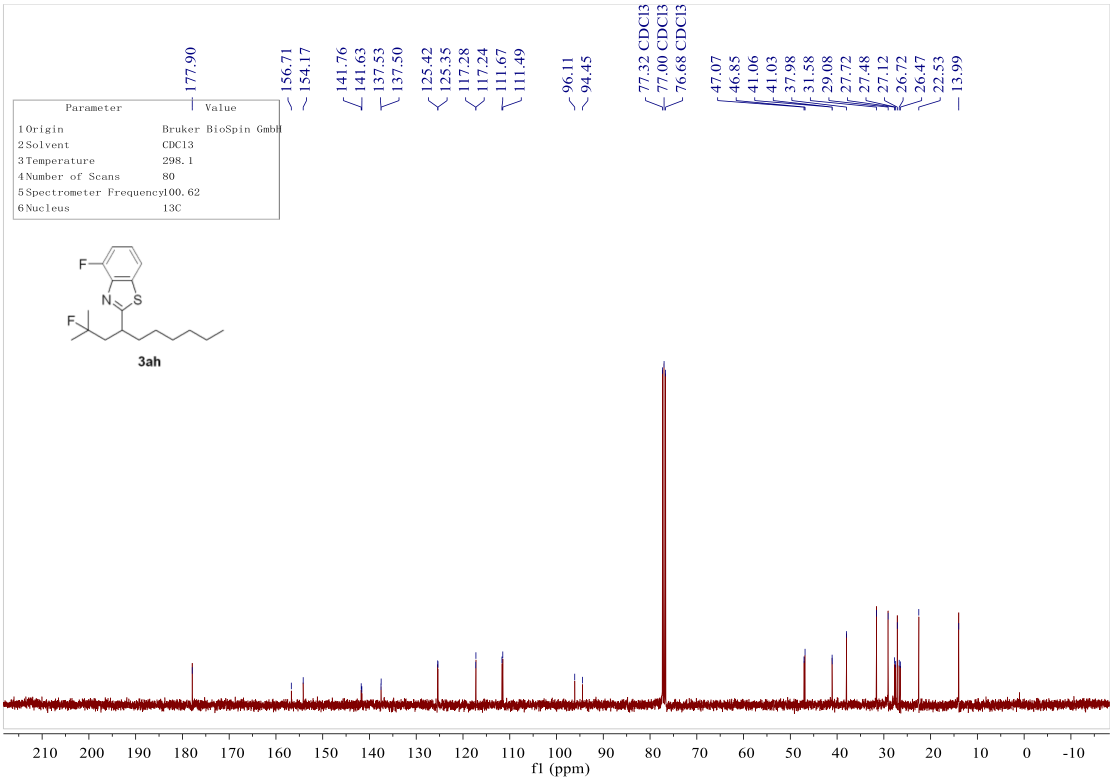

Supplementary Fig. 130 ^{13}C NMR spectra (100 MHz, $CDCl_3$, 25 ºC) of **3ah**

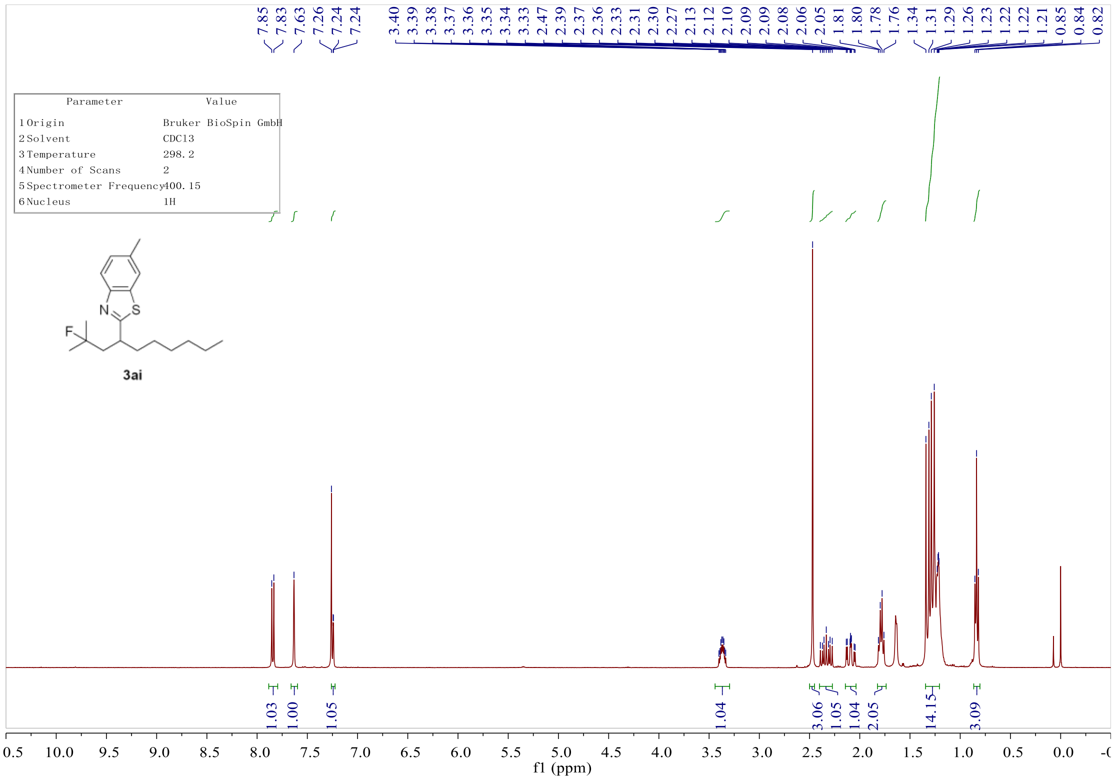

Supplementary Fig. 131 ^{1}H NMR spectra (400 MHz, $CDCl_3$, 25 ºC) of **3ai**

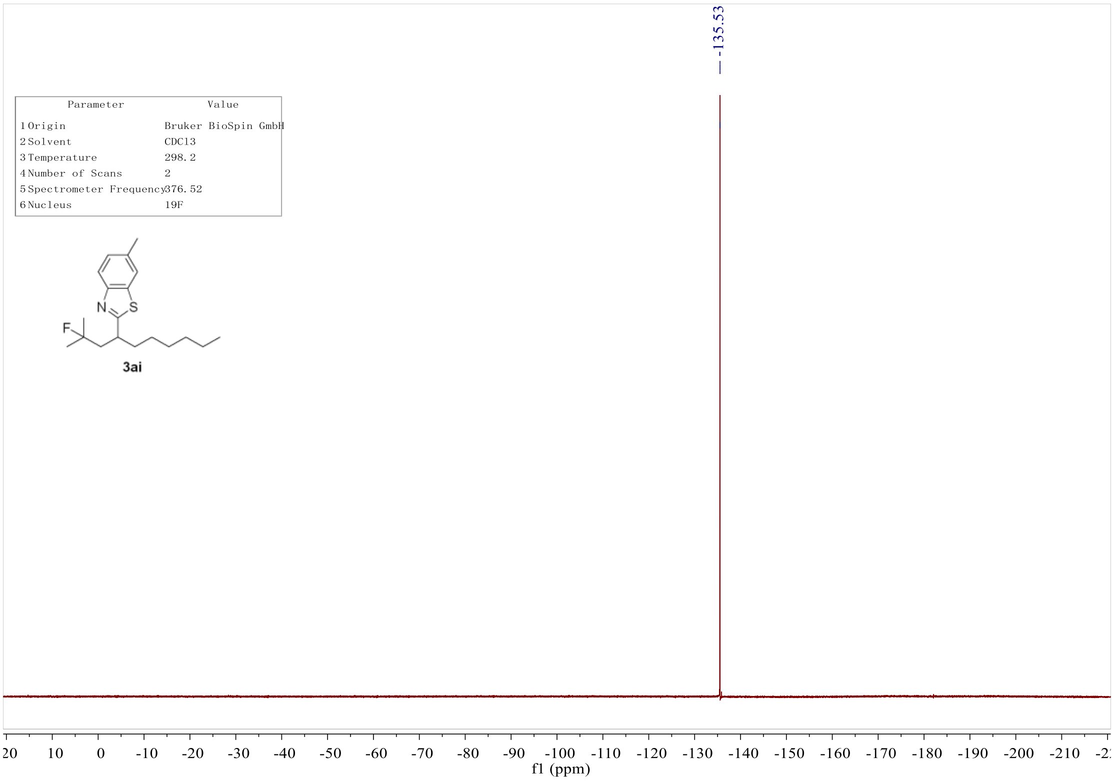

Supplementary Fig. 132 ^{19}F NMR spectra (376 MHz, $CDCl_3$, 25 ºC) of **3ai**

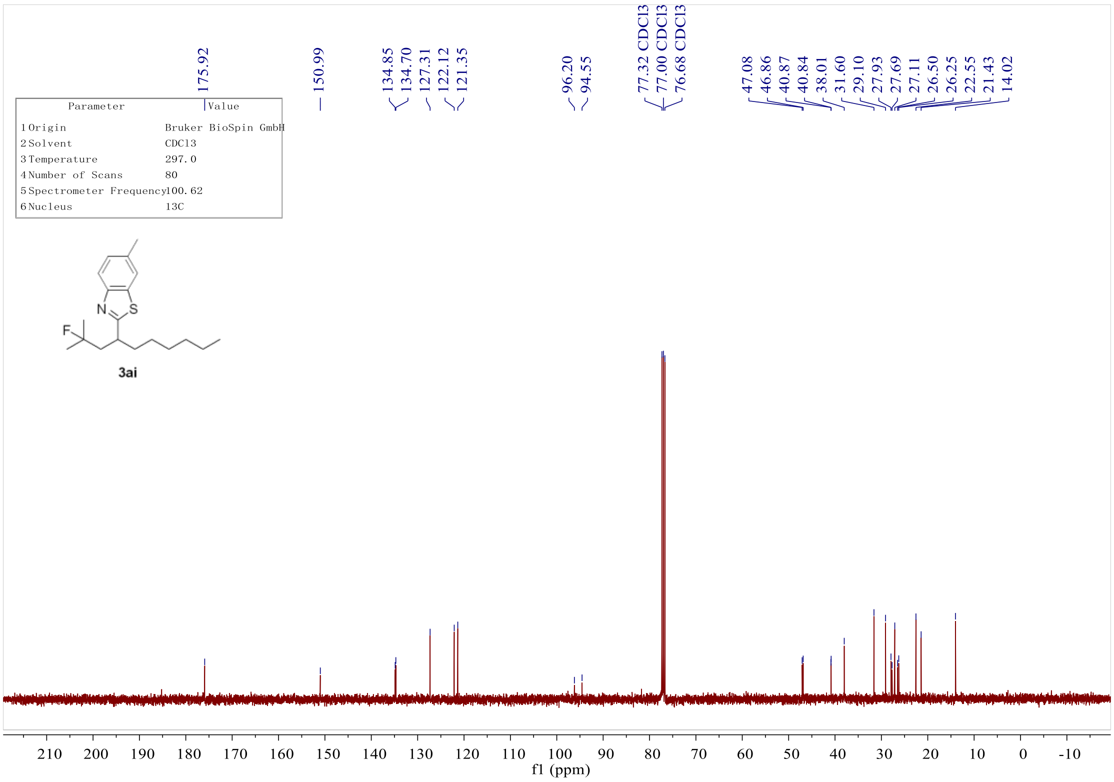

Supplementary Fig. 133 ^{13}C NMR spectra (100 MHz, $CDCl_3$, 25 ºC) of **3ai**

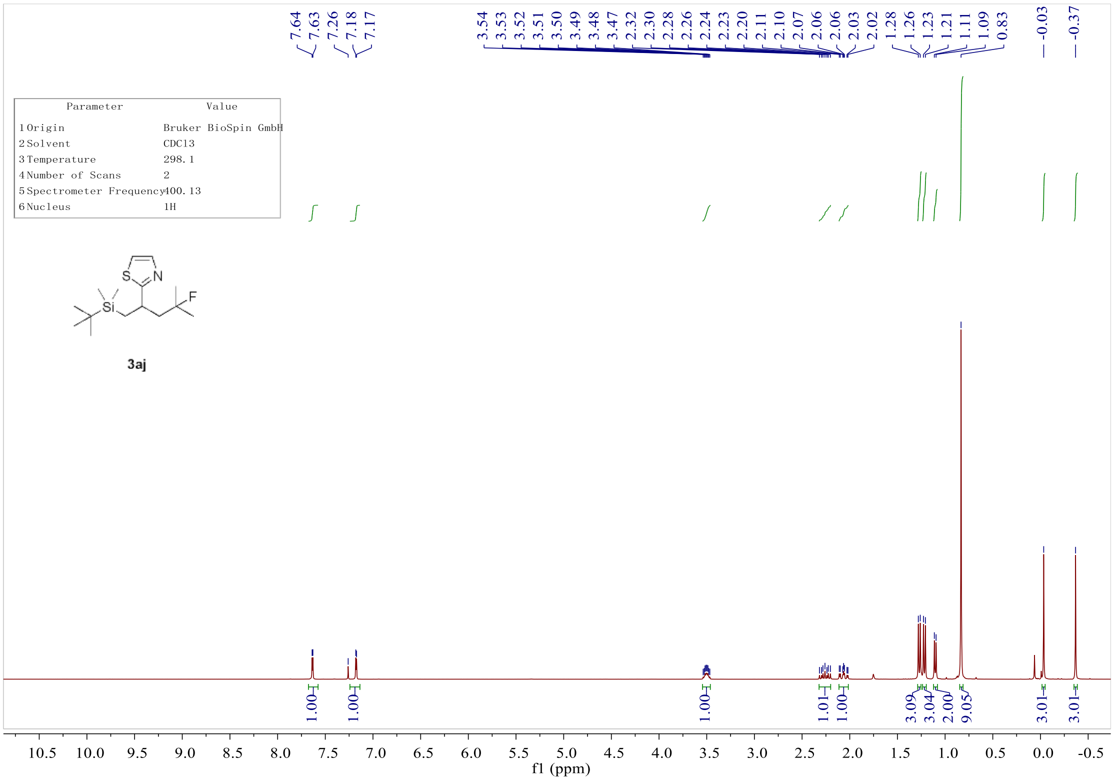

Supplementary Fig. 134 ^{1}H NMR spectra (400 MHz, $CDCl_3$, 25 ºC) of **3aj**

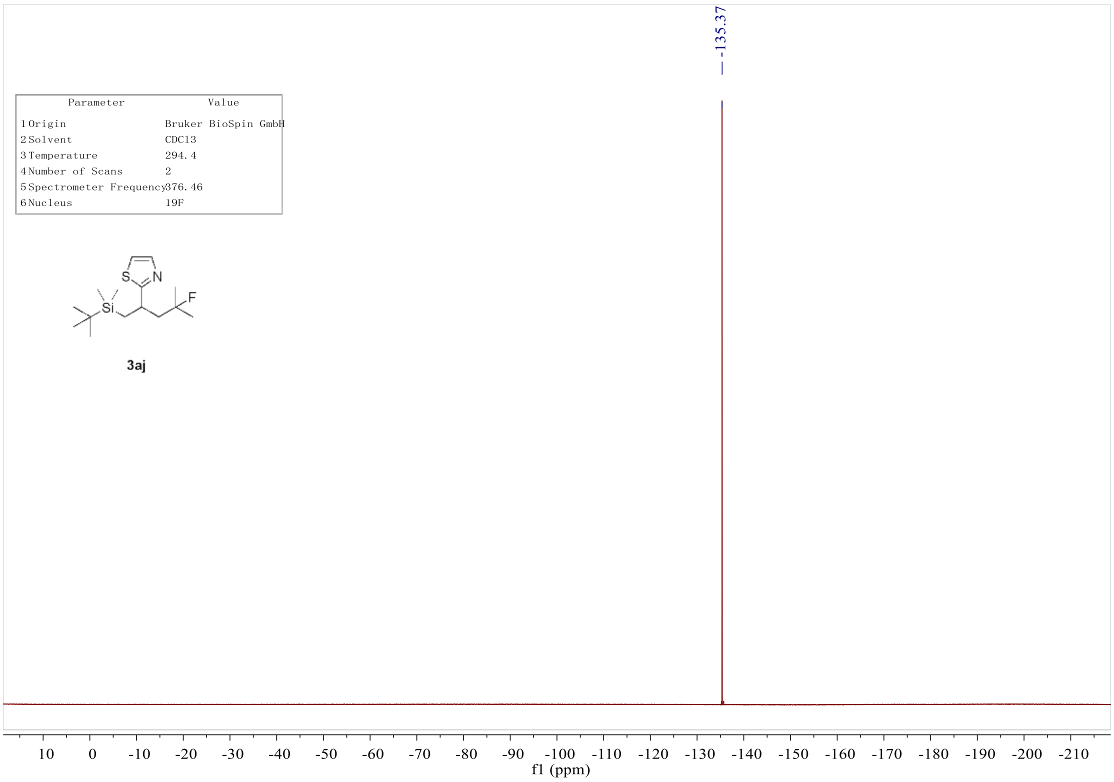

Supplementary Fig. 135 ^{19}F NMR spectra (376 MHz, $CDCl_3$, 25 ºC) of **3aj**

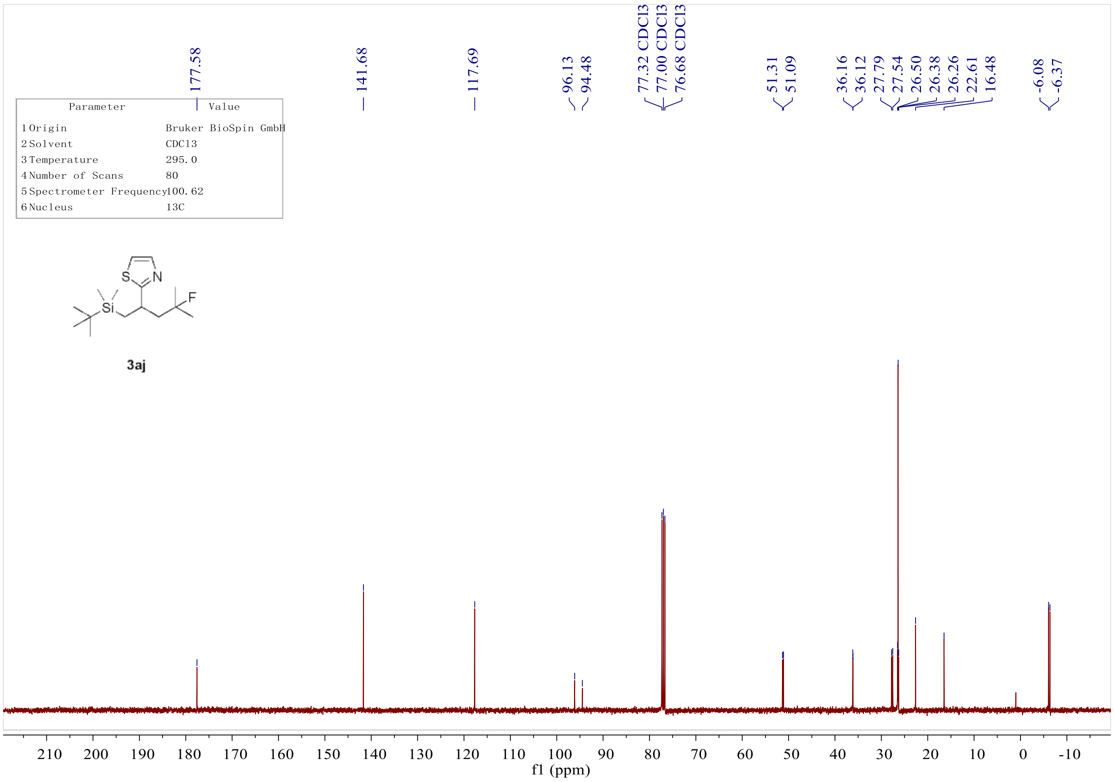

Supplementary Fig. 136 ^{13}C NMR spectra (100 MHz, $CDCl_3$, 25 ºC) of **3aj**

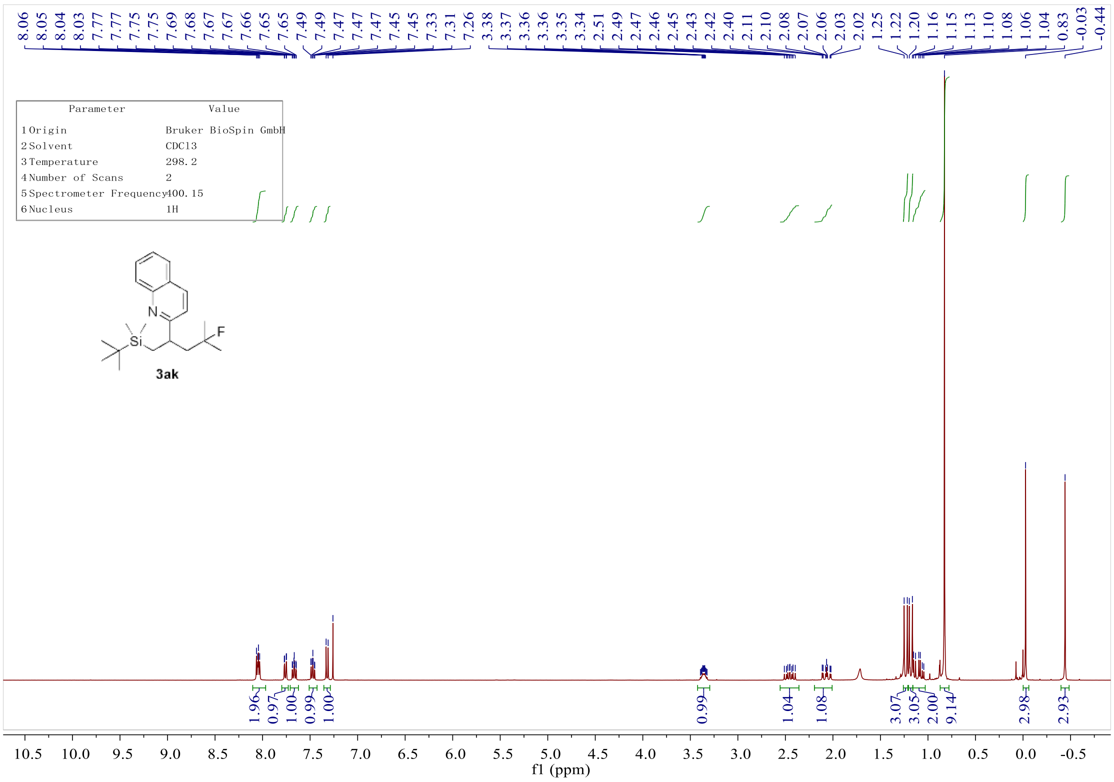

Supplementary Fig. 137 ^{1}H NMR spectra (400 MHz, $CDCl_3$, 25 ºC) of **3ak**

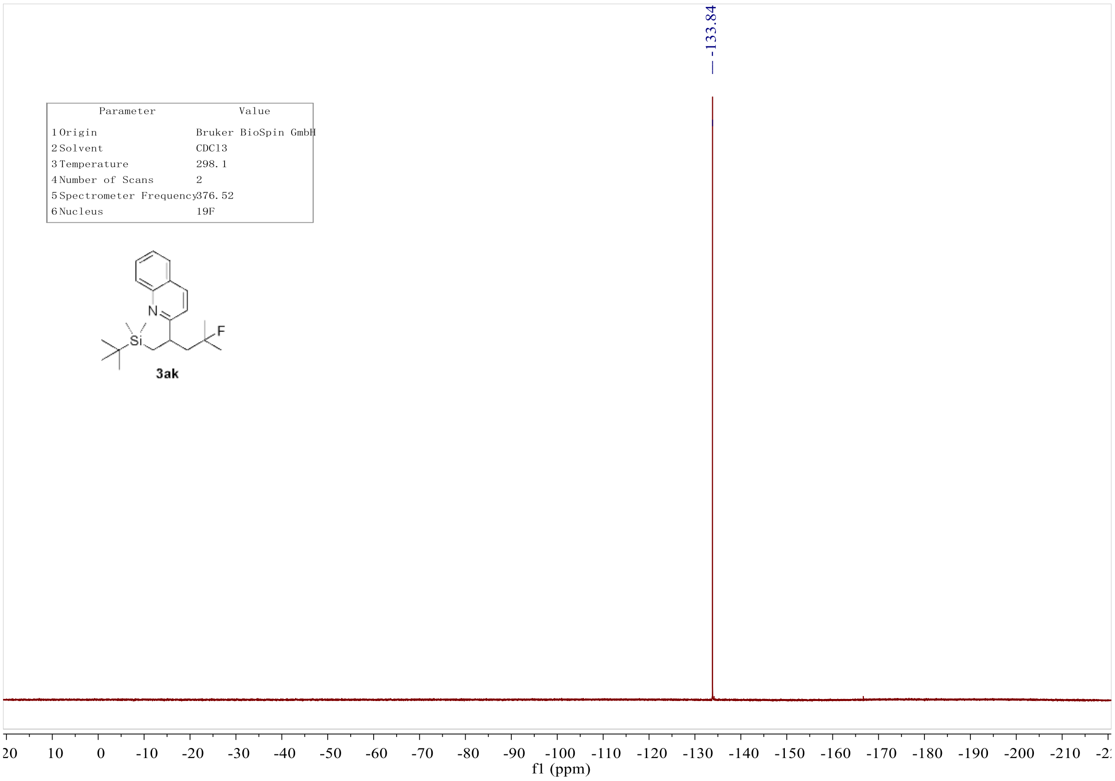

Supplementary Fig. 138 ^{19}F NMR spectra (376 MHz, $CDCl_3$, 25 °C) of **3ak**

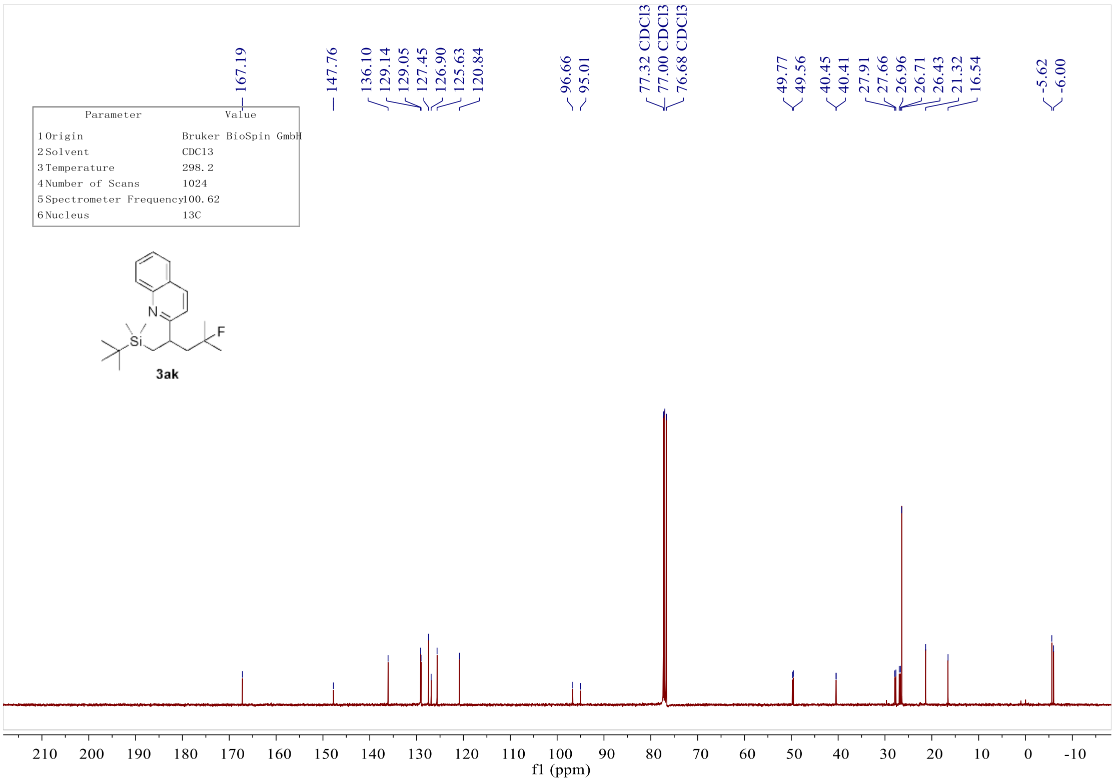

Supplementary Fig. 139 ^{13}C NMR spectra (100 MHz, $CDCl_3$, 25 °C) of **3ak**

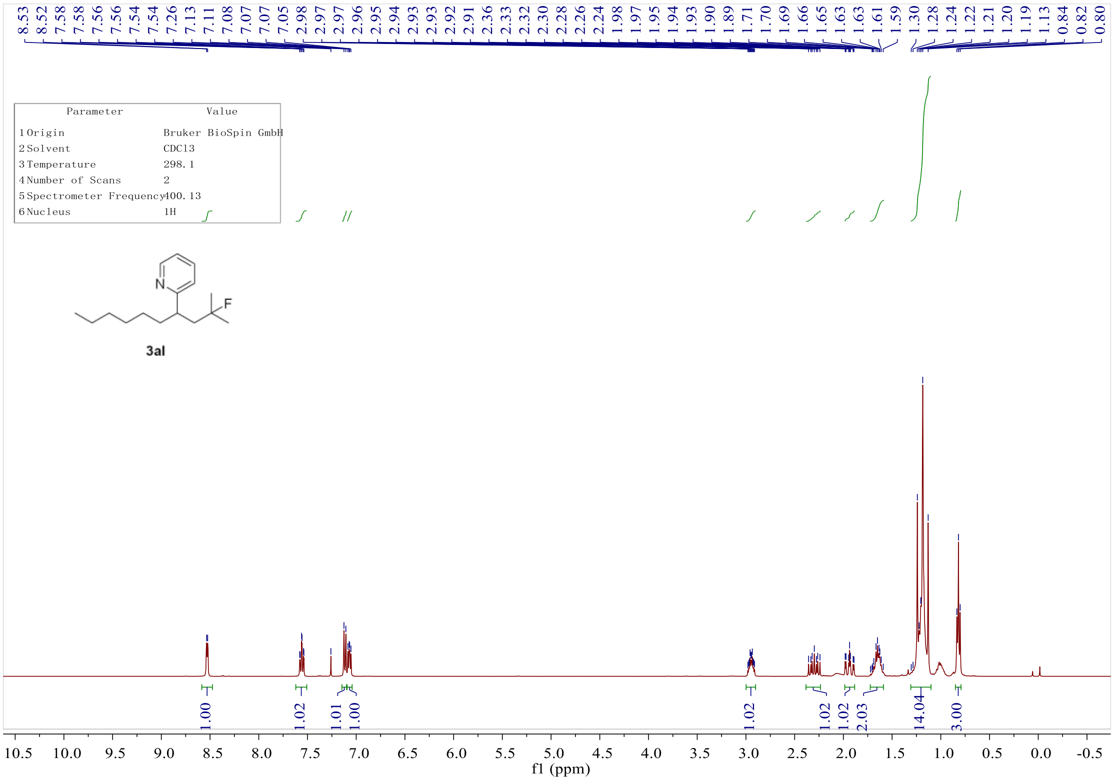

Supplementary Fig. 140 ^{1}H NMR spectra (400 MHz, $CDCl_3$, 25 ºC) of **3al**

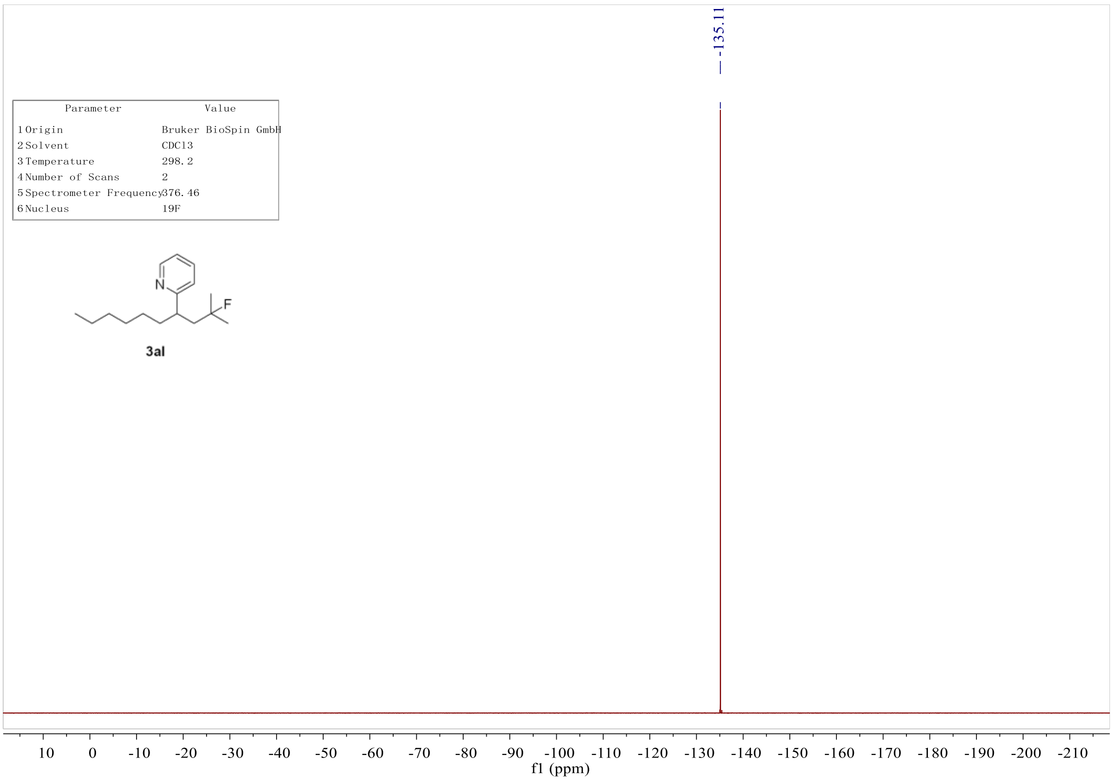

Supplementary Fig. 141 ^{19}F NMR spectra (376 MHz, $CDCl_3$, 25 ºC) of **3al**

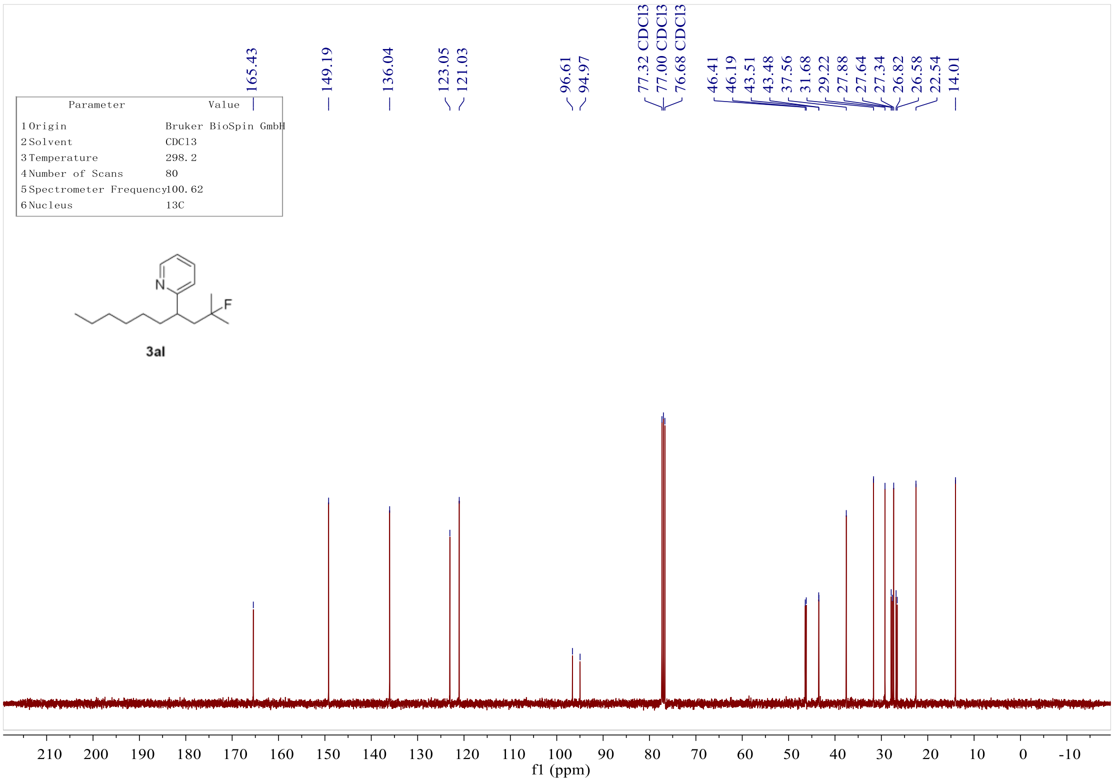

Supplementary Fig. 142 ^{13}C NMR spectra (100 MHz, $CDCl_3$, 25 ºC) of **3al**

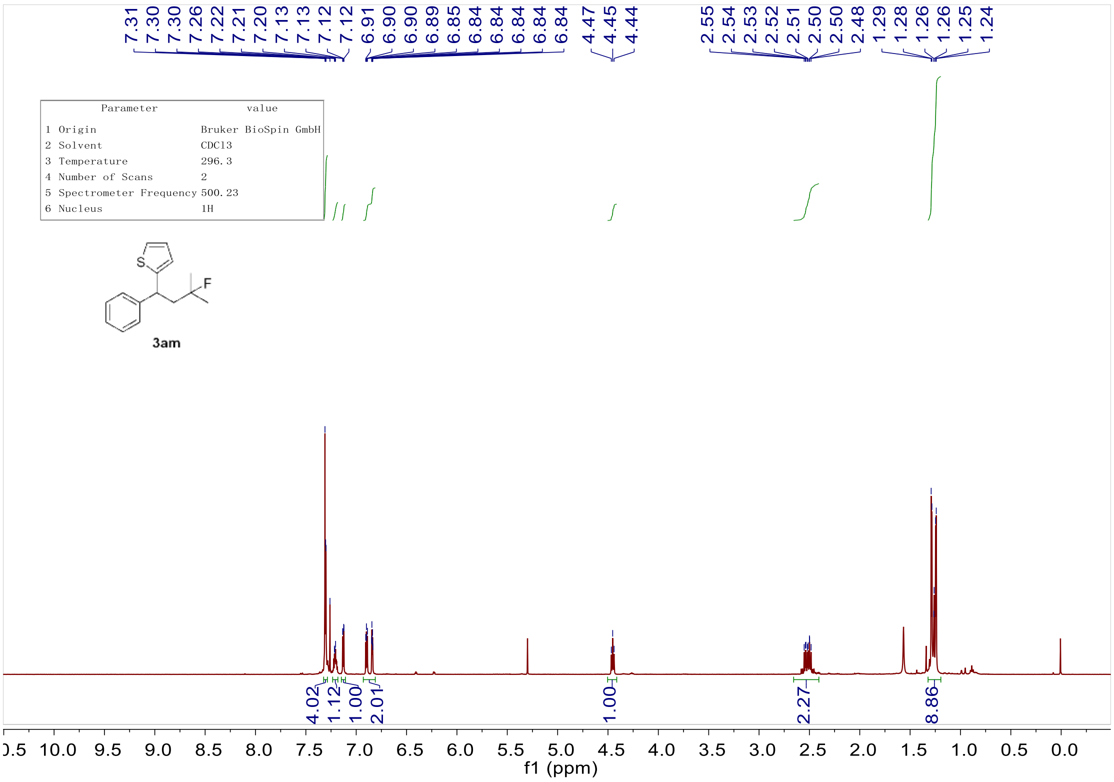

Supplementary Fig. 143 ^{1}H NMR spectra (500 MHz, $CDCl_3$, 25 ºC) of **3am**

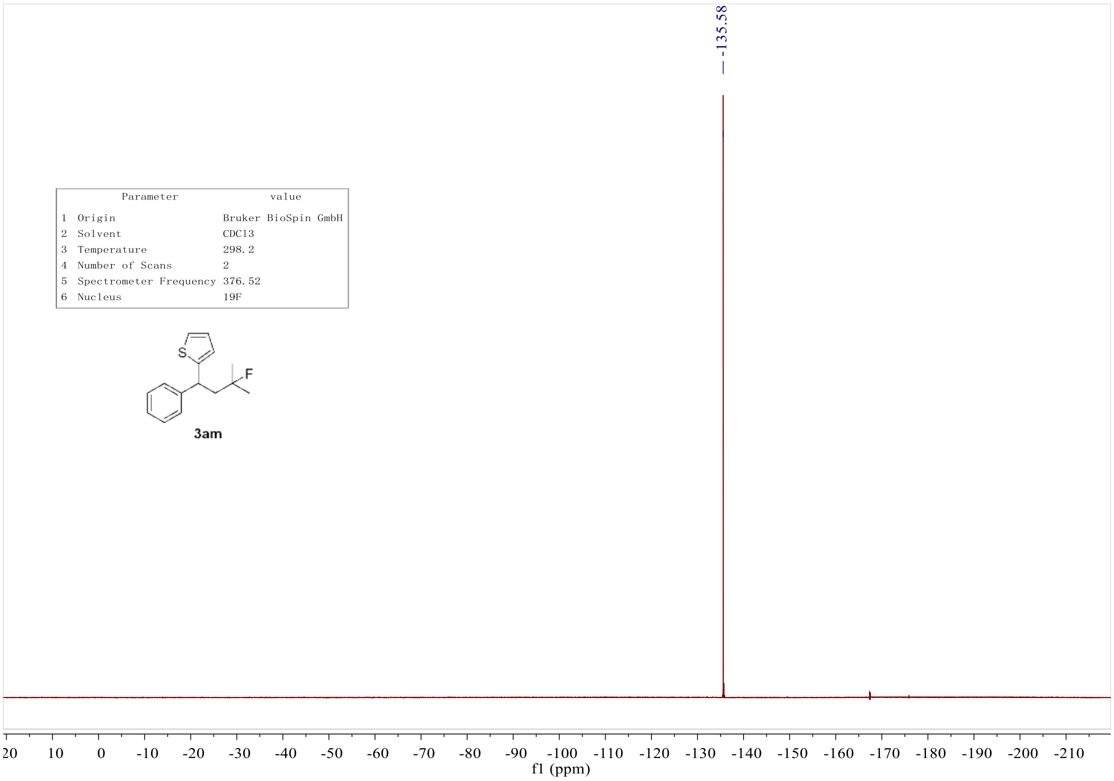

Supplementary Fig. 144 ^{19}F NMR spectra (376 MHz, $CDCl_3$, 25 ºC) of **3am**

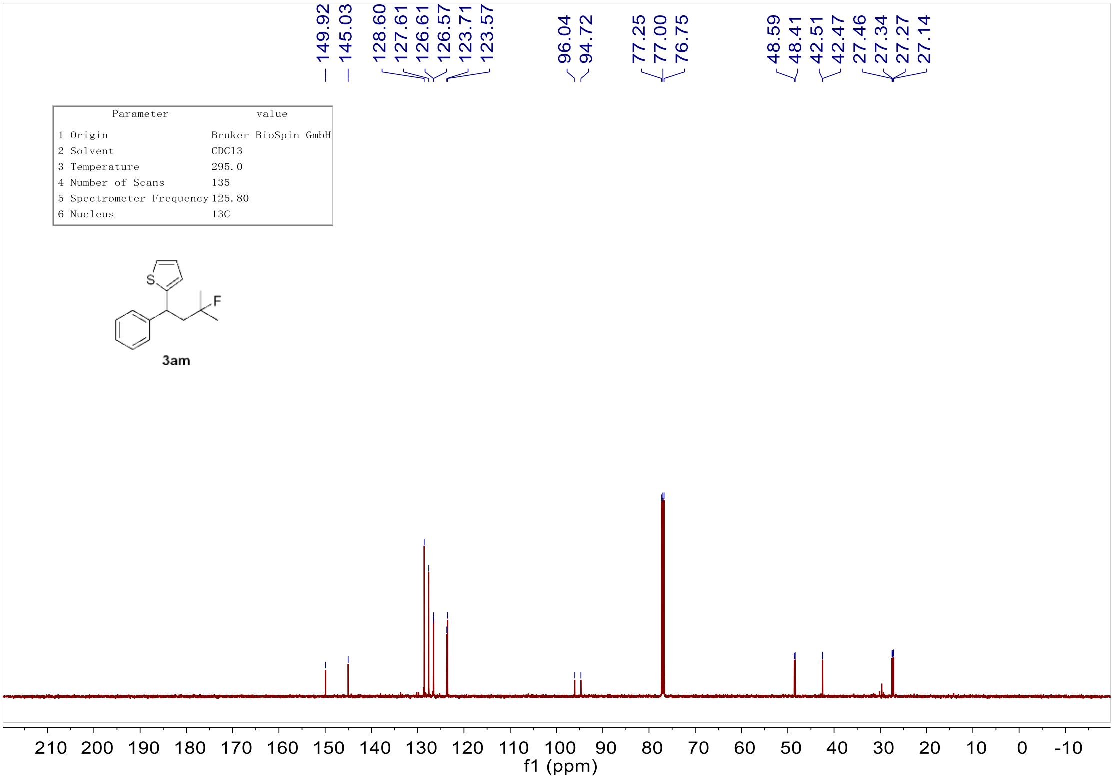

Supplementary Fig. 145 ^{13}C NMR spectra (125 MHz, $CDCl_3$, 25 ºC) of **3am**

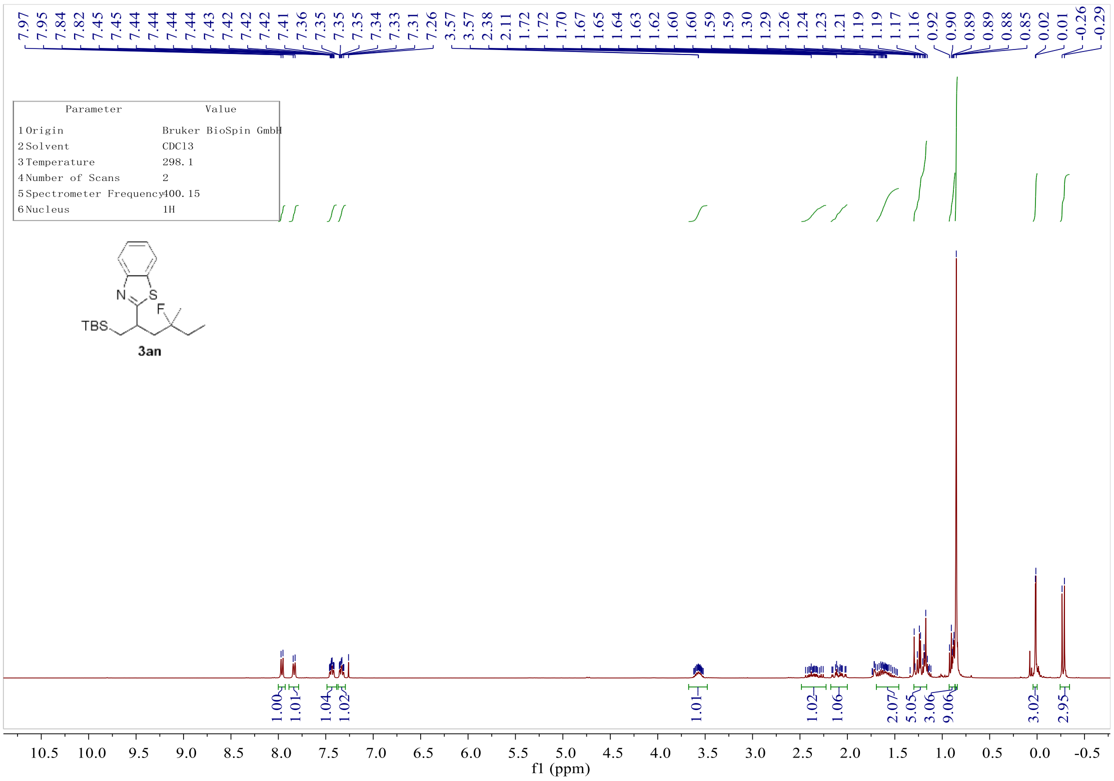

Supplementary Fig. 146 ^{1}H NMR spectra (400 MHz, $CDCl_3$, 25 °C) of **3an**

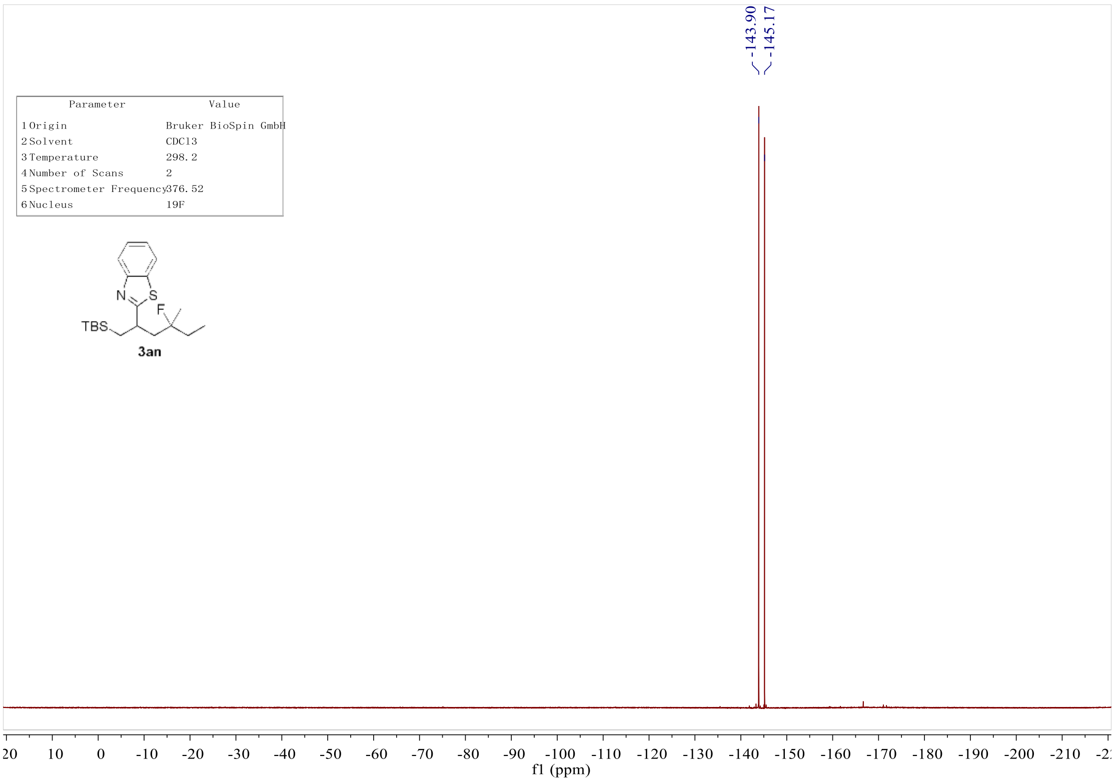

Supplementary Fig. 147 ^{19}F NMR spectra (376 MHz, $CDCl_3$, 25 °C) of **3an**

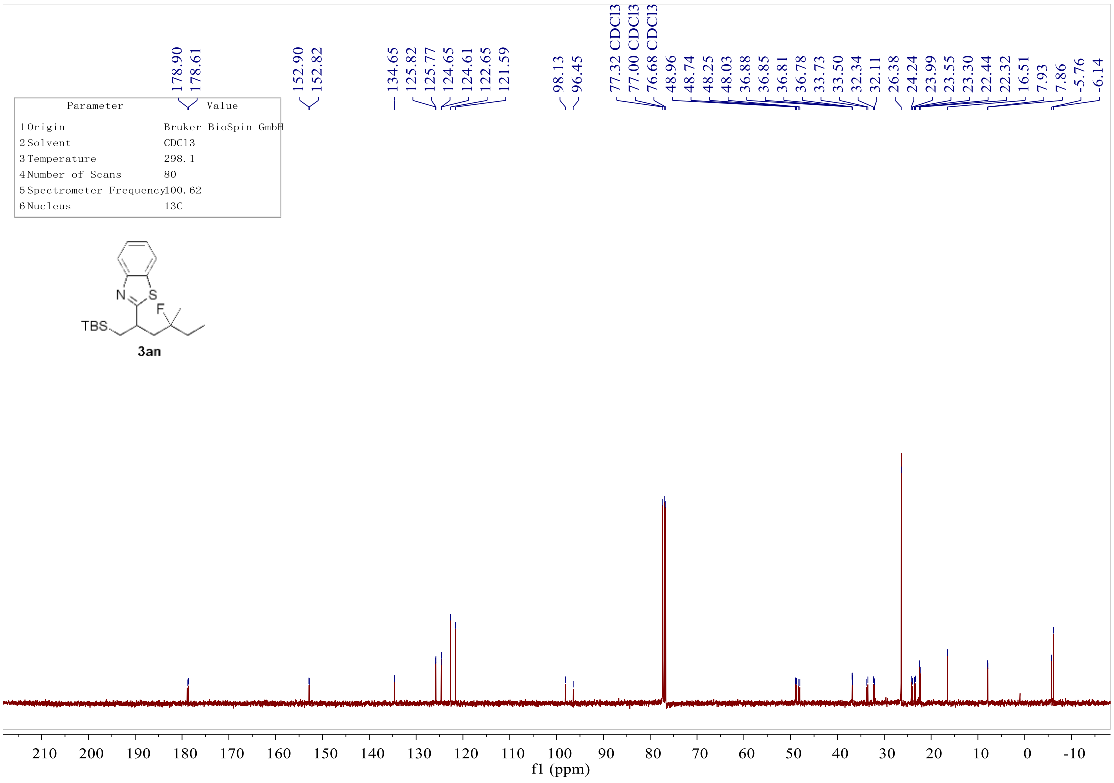

Supplementary Fig. 148 ^{13}C NMR spectra (100 MHz, $CDCl_3$, 25 °C) of **3an**

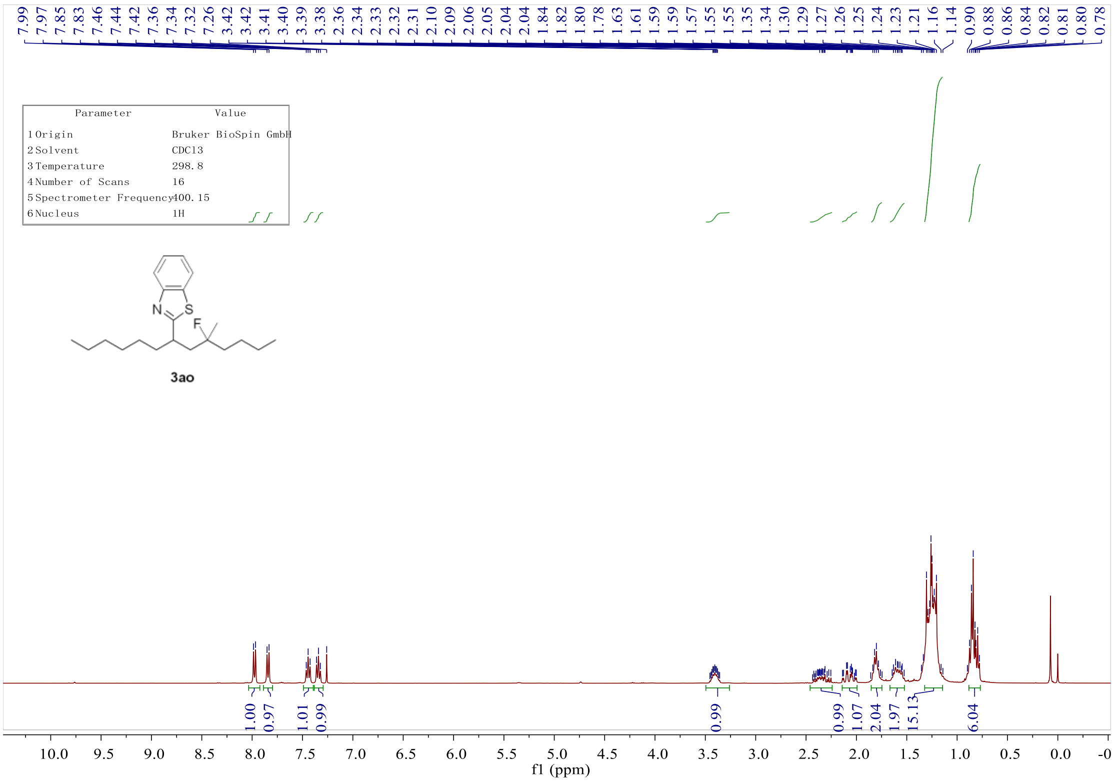

Supplementary Fig. 149 ^{1}H NMR spectra (400 MHz, $CDCl_3$, 25 °C) of **3ao**

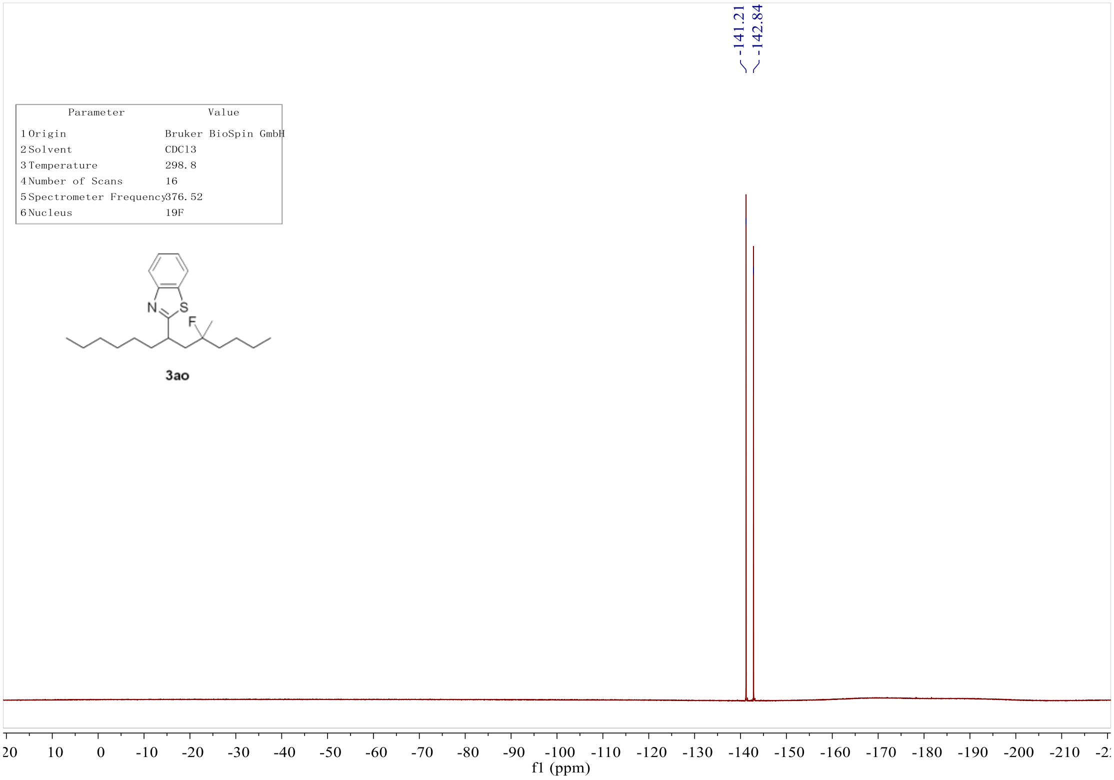

Supplementary Fig. 150 ^{19}F NMR spectra (376 MHz, $CDCl_3$, 25 °C) of **3ao**

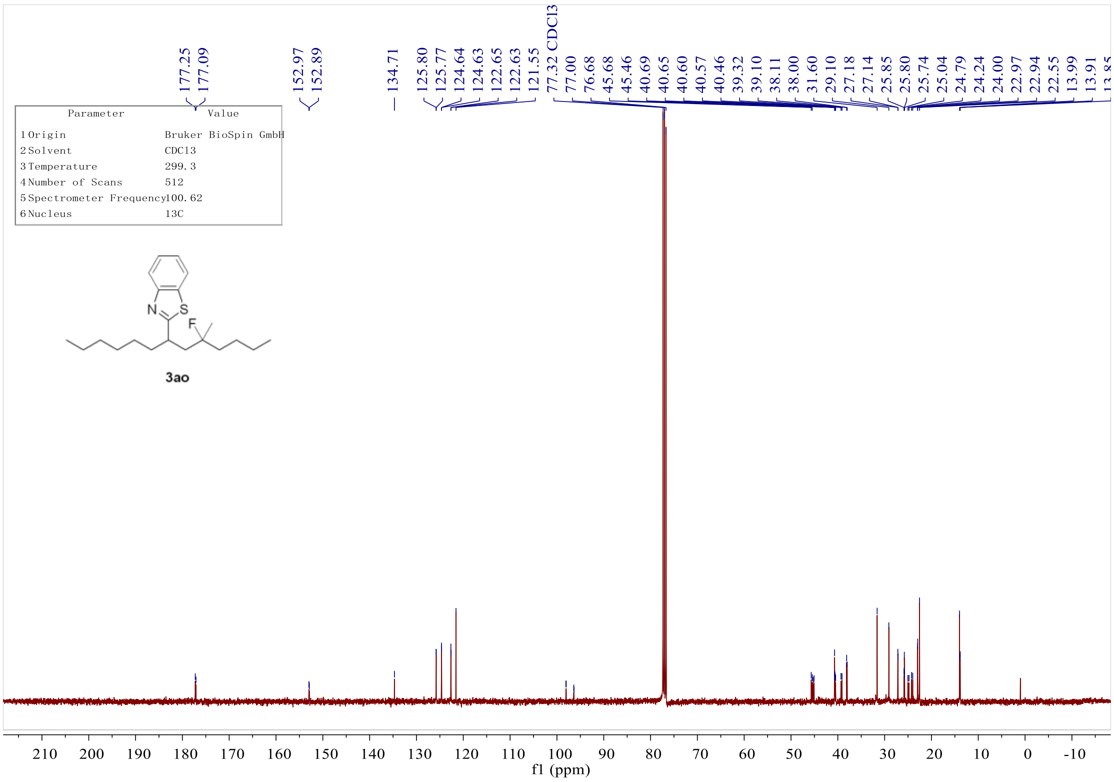

Supplementary Fig. 151 ^{13}C NMR spectra (100 MHz, $CDCl_3$, 25 °C) of **3ao**

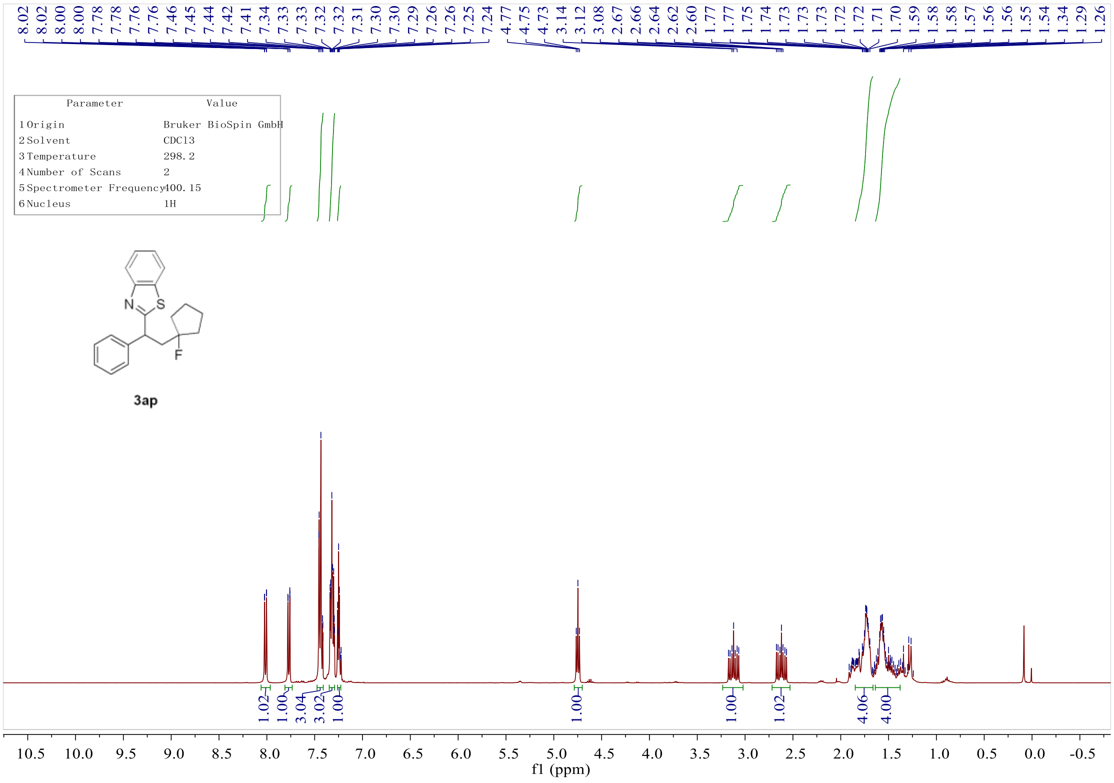

Supplementary Fig. 152 ^{1}H NMR spectra (400 MHz, $CDCl_3$, 25 °C) of **3ap**

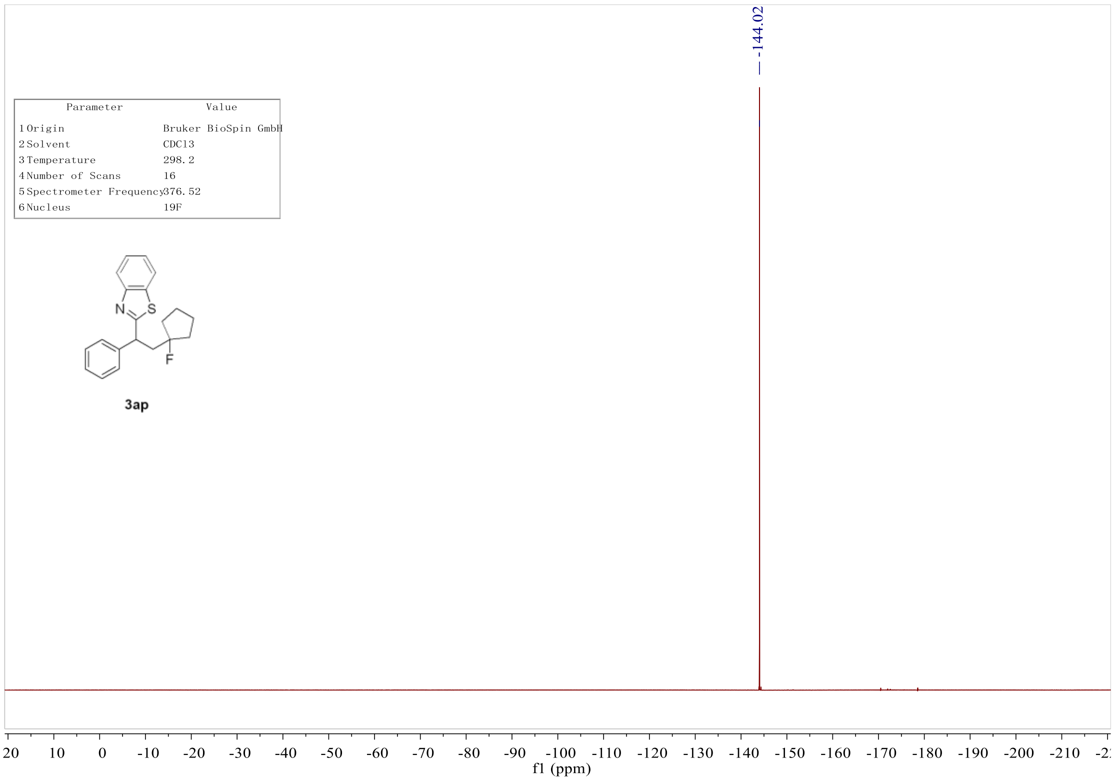

Supplementary Fig. 153 ^{19}F NMR spectra (376 MHz, $CDCl_3$, 25 °C) of **3ap**

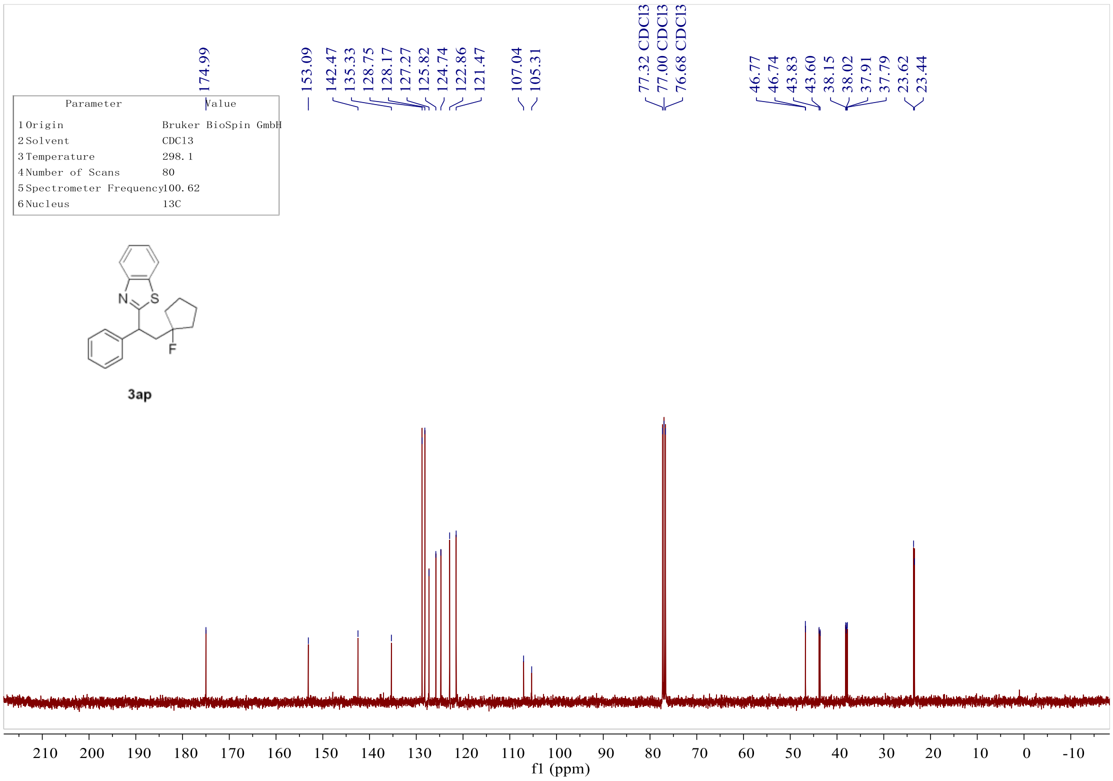

Supplementary Fig. 154 ^{13}C NMR spectra (100 MHz, $CDCl_3$, 25 °C) of **3ap**

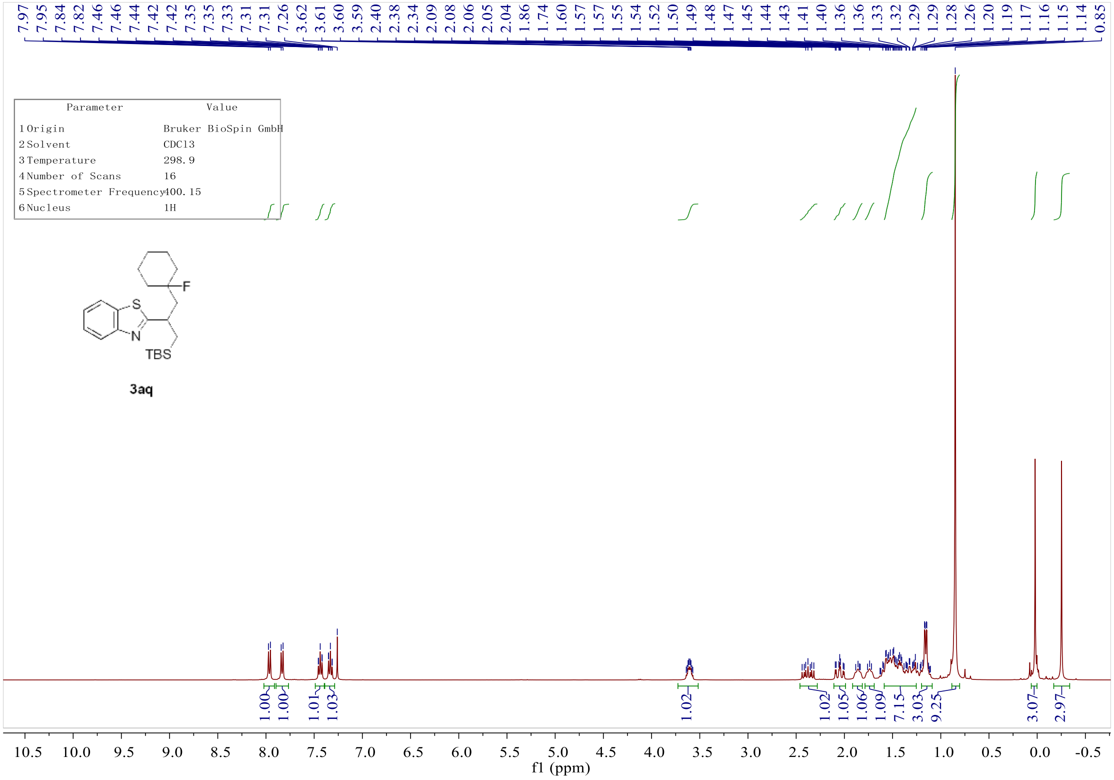

Supplementary Fig. 155 ^{1}H NMR spectra (400 MHz, $CDCl_3$, 25 °C) of **3aq**

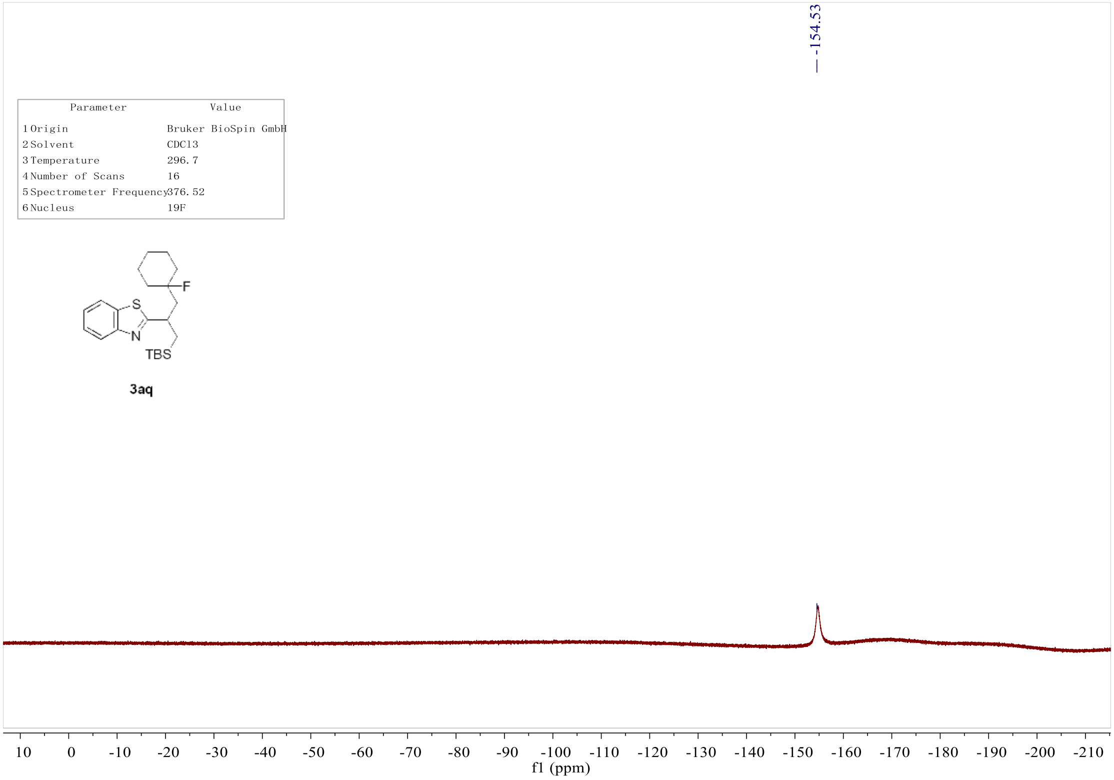

Supplementary Fig. 156 ^{19}F NMR spectra (376 MHz, $CDCl_3$, 25 °C) of **3aq**

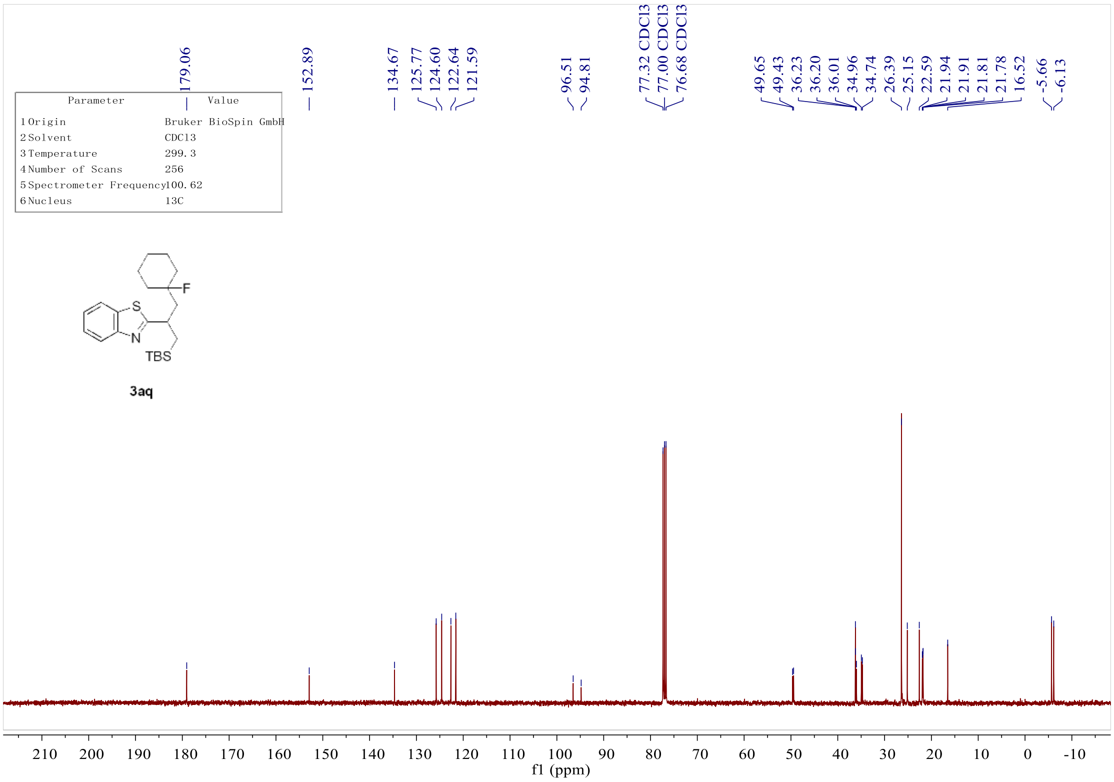

Supplementary Fig. 157 ^{13}C NMR spectra (100 MHz, $CDCl_3$, 25 °C) of **3aq**

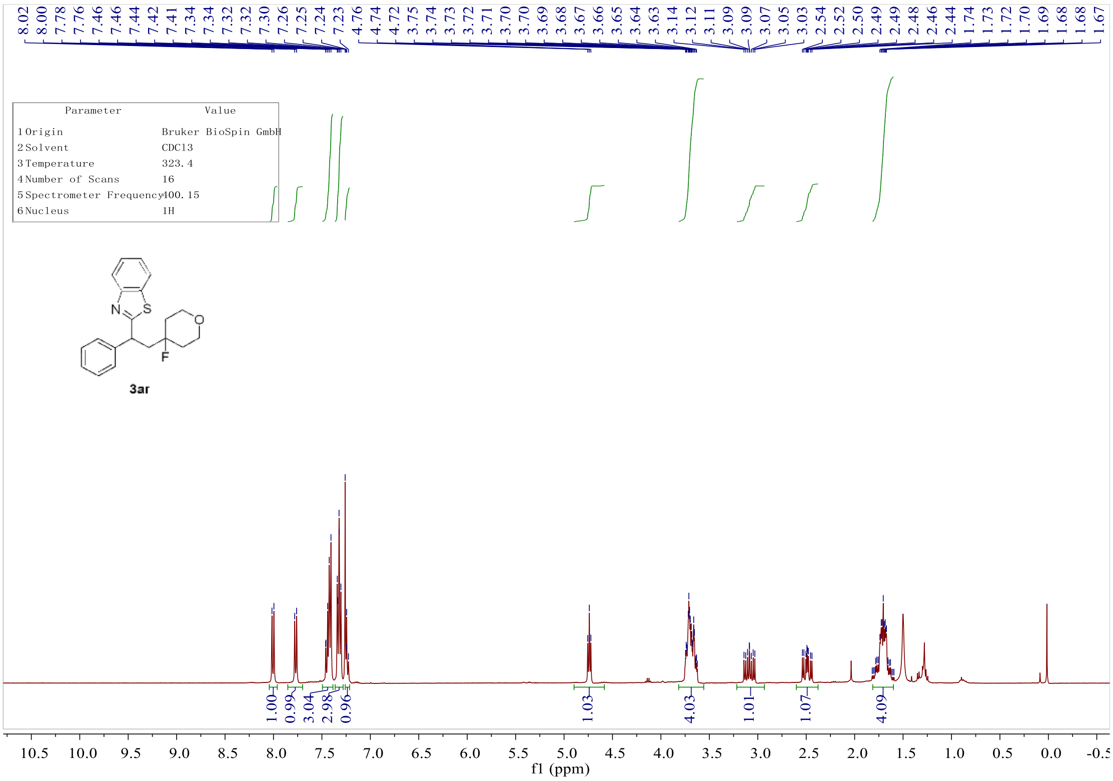

Supplementary Fig. 158 ^{1}H NMR spectra (400 MHz, $CDCl_3$, 25 °C) of **3ar**

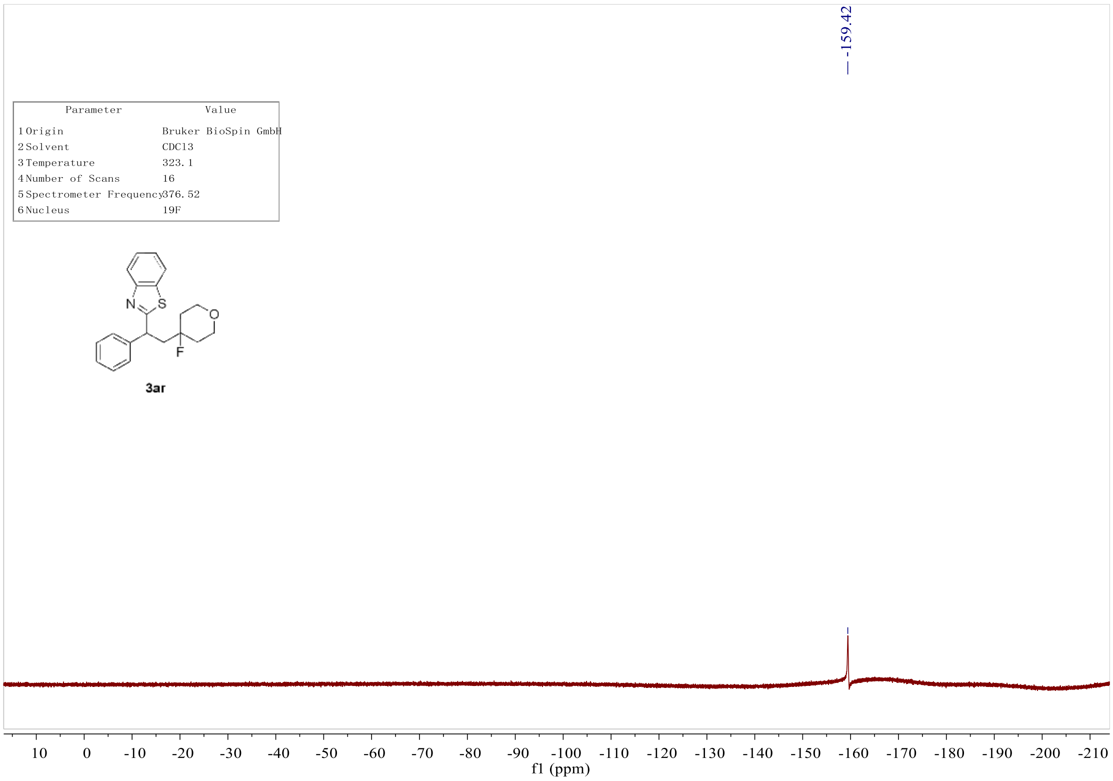

Supplementary Fig. 159 ^{19}F NMR spectra (376 MHz, $CDCl_3$, 25 °C) of **3ar**

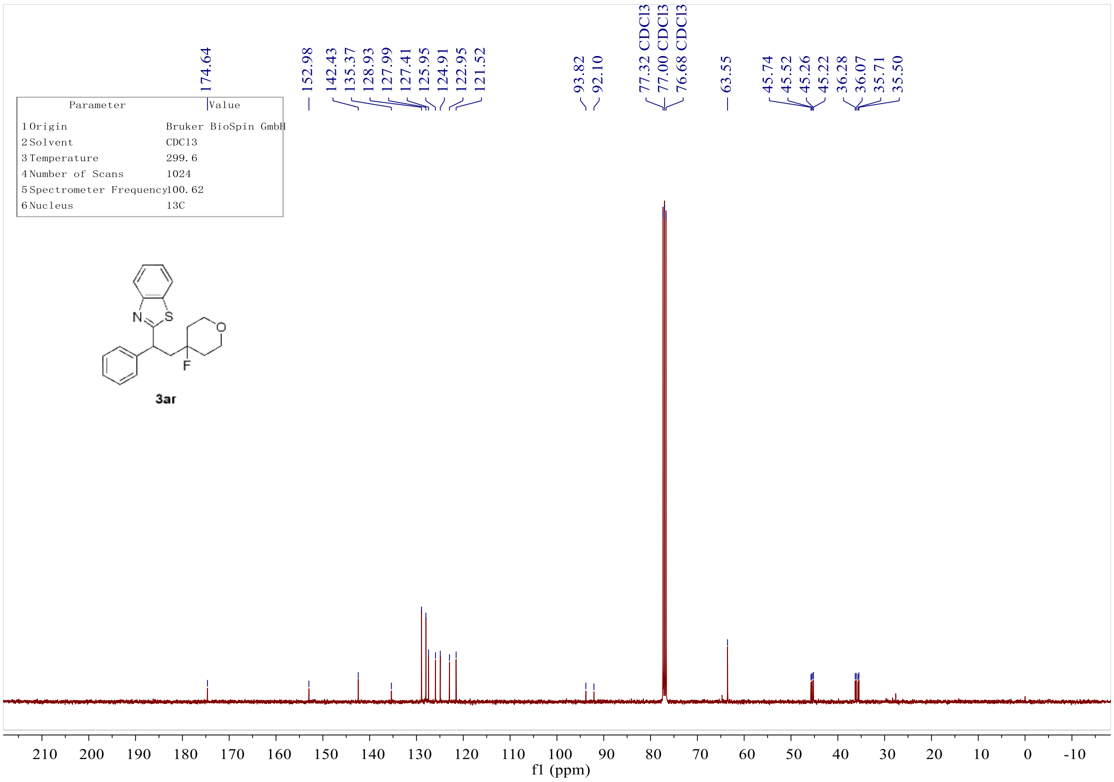

Supplementary Fig. 160 ^{13}C NMR spectra (100 MHz, $CDCl_3$, 25 °C) of **3ar**

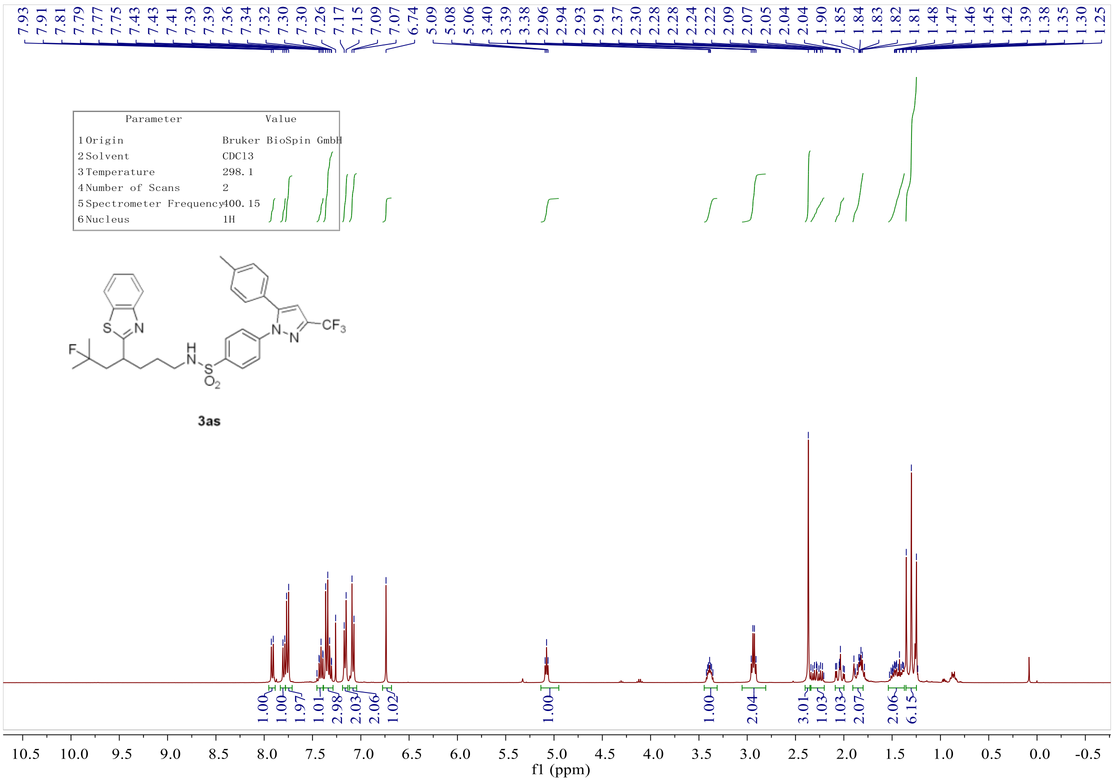

Supplementary Fig. 161 ^{1}H NMR spectra (400 MHz, $CDCl_3$, 25 °C) of **3as**

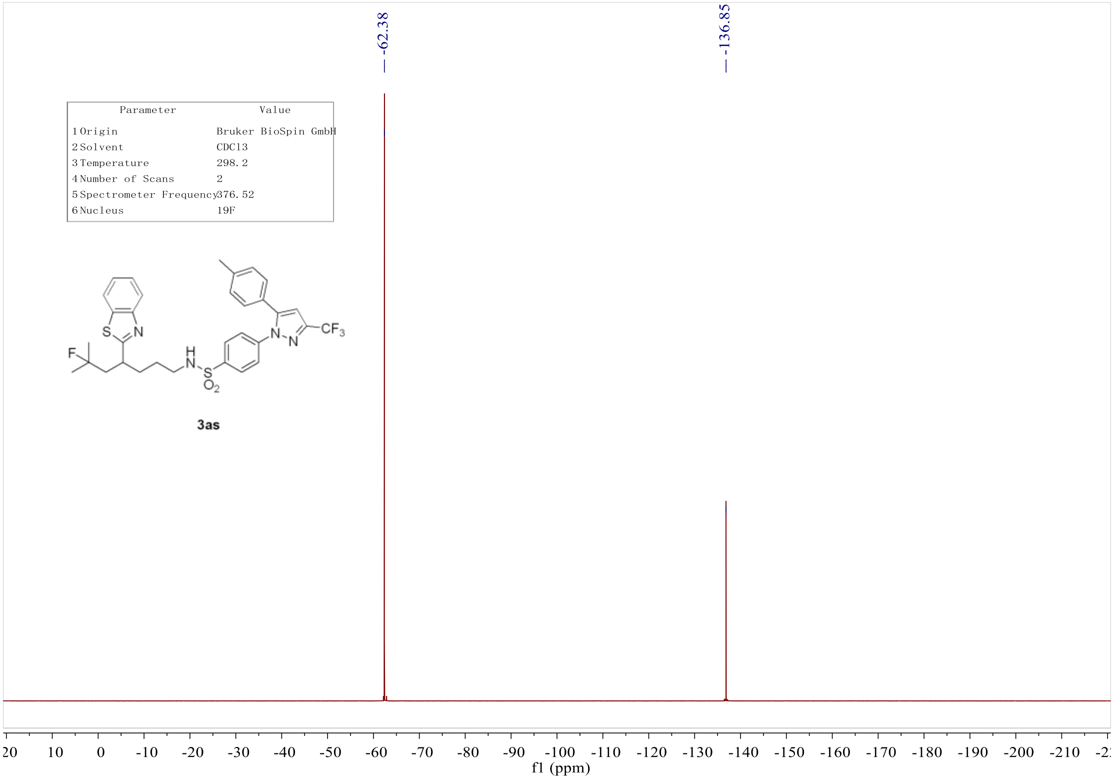

Supplementary Fig. 162 ^{19}F NMR spectra (376 MHz, $CDCl_3$, 25 °C) of **3as**

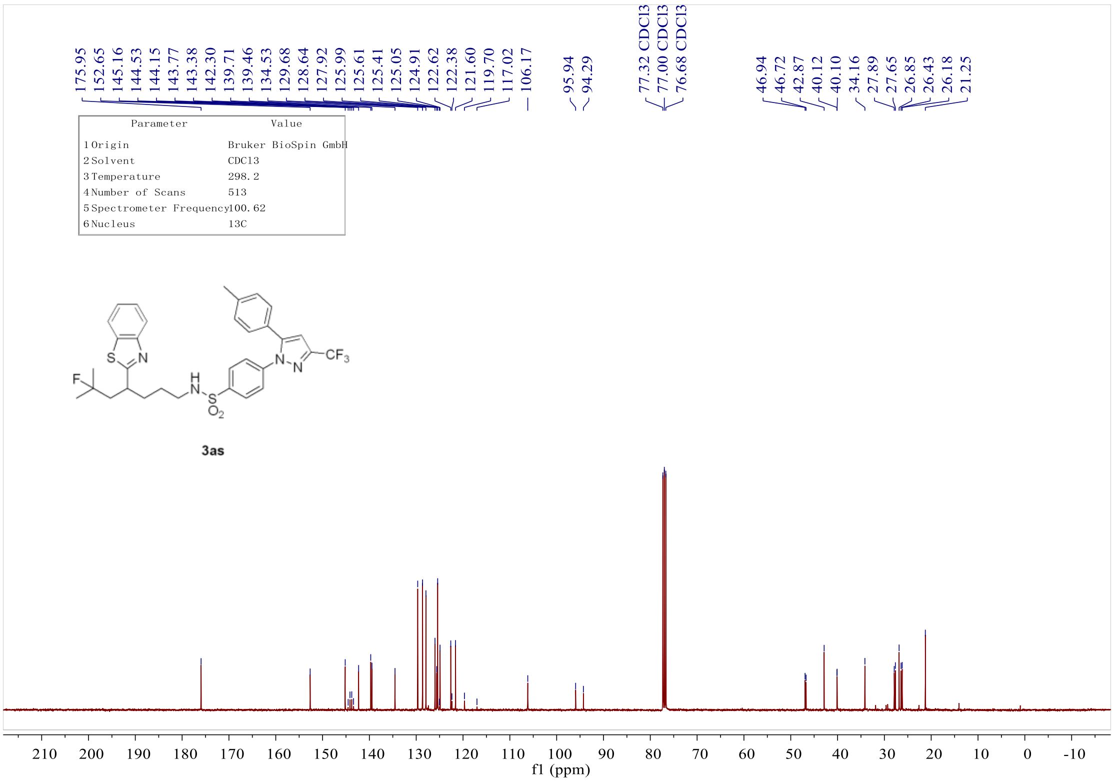

Supplementary Fig. 163 ^{13}C NMR spectra (100 MHz, $CDCl_3$, 25 °C) of **3as**

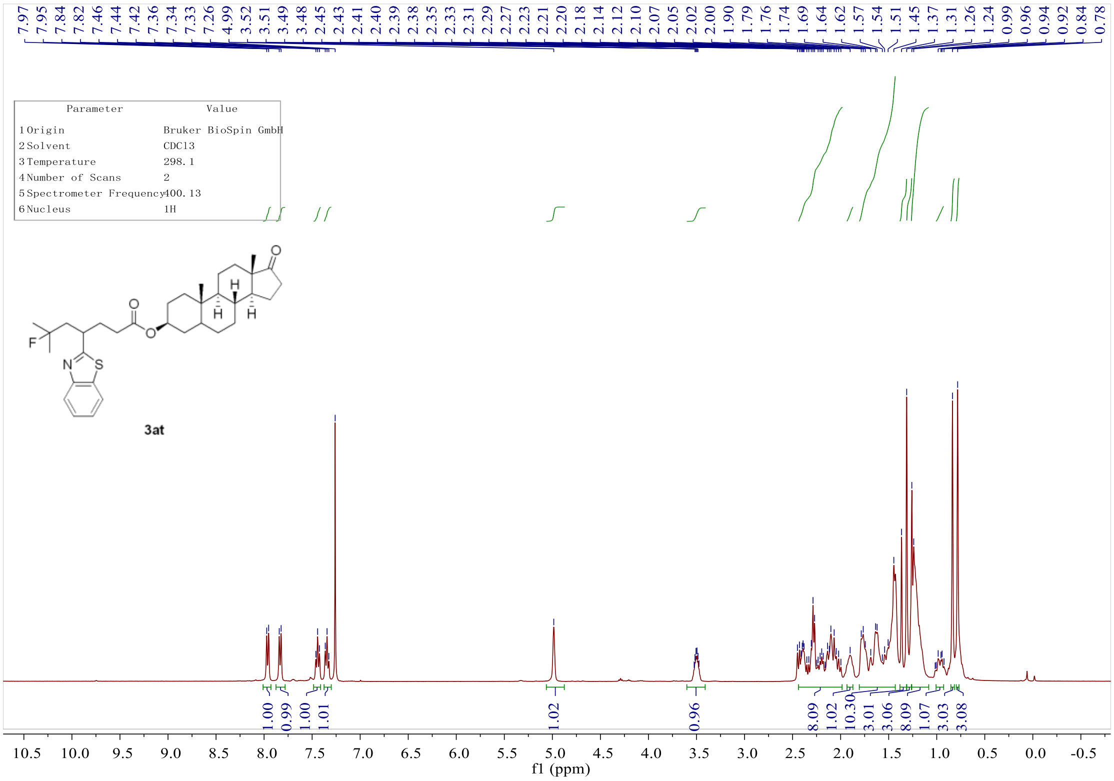

Supplementary Fig. 164 ^{1}H NMR spectra (400 MHz, $CDCl_3$, 25 ºC) of **3at**

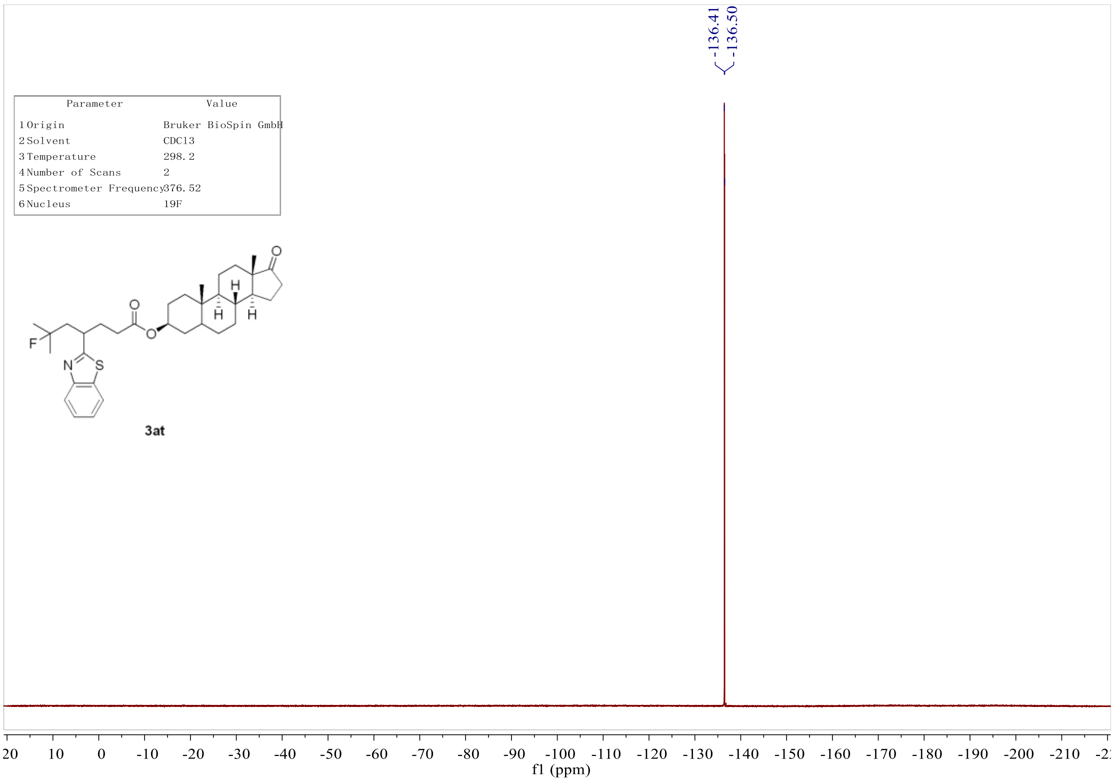

Supplementary Fig. 165 ^{19}F NMR spectra (376 MHz, $CDCl_3$, 25 ºC) of **3at**

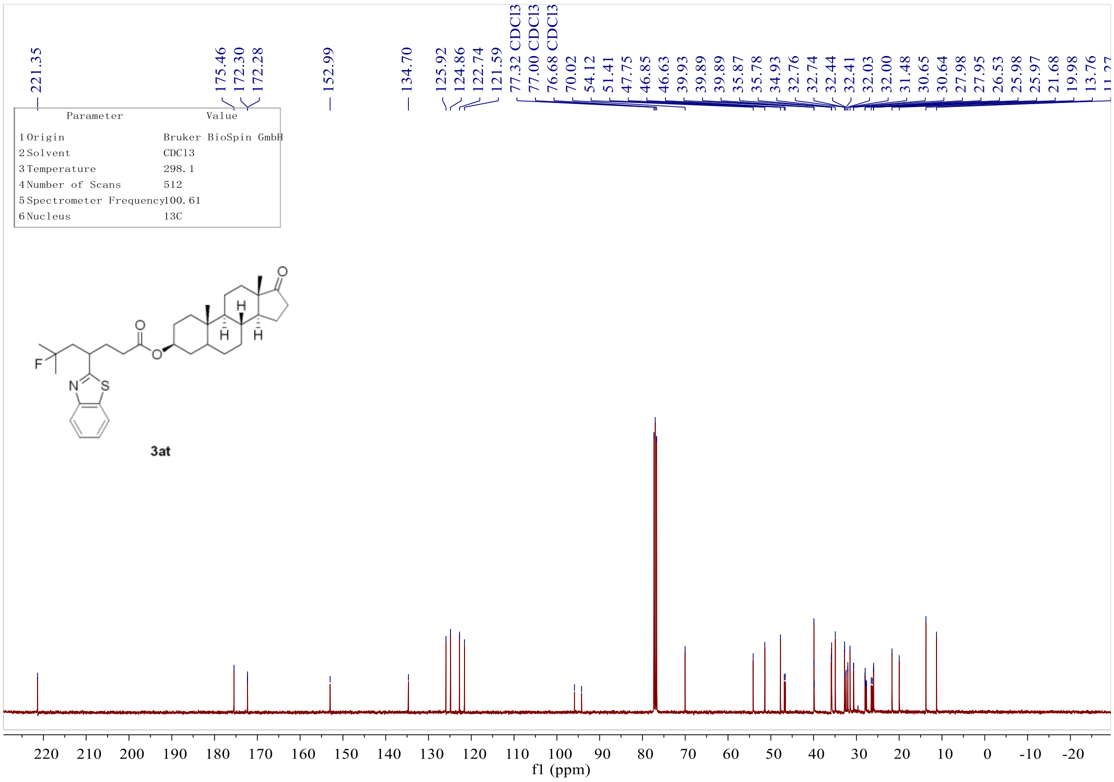

Supplementary Fig. 166 ^{13}C NMR spectra (100 MHz, $CDCl_3$, 25 °C) of **3at**

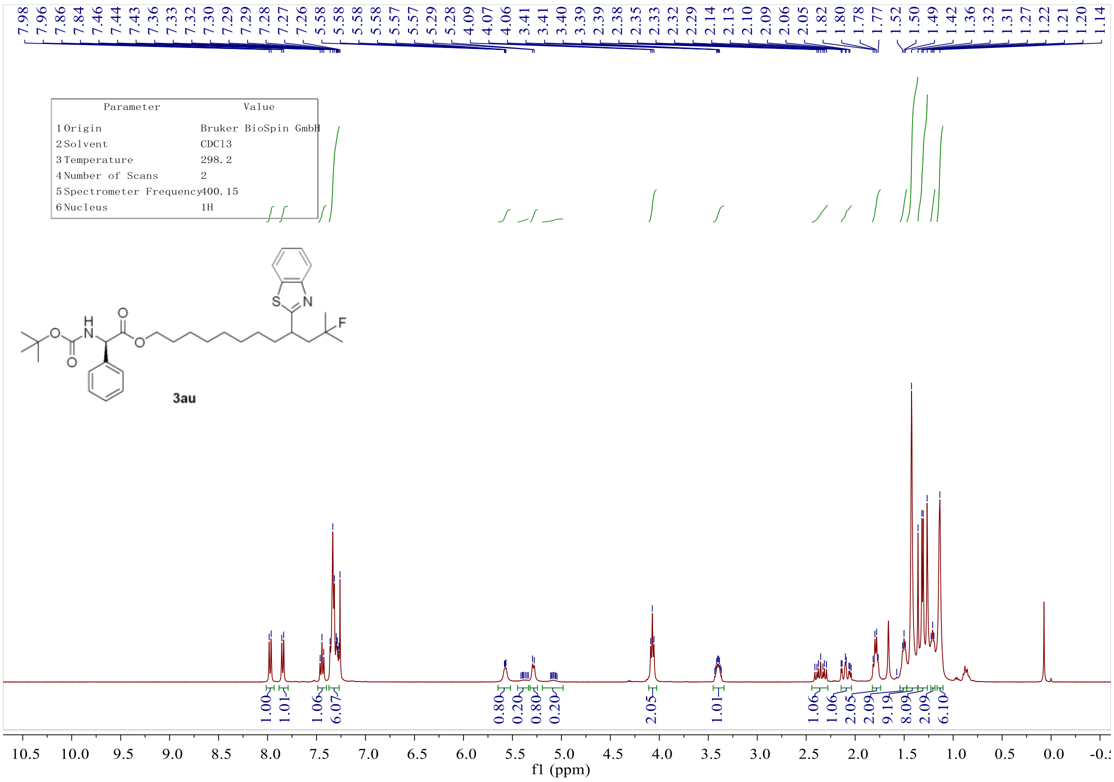

Supplementary Fig. 167 ^{1}H NMR spectra (400 MHz, $CDCl_3$, 25 °C) of **3au**

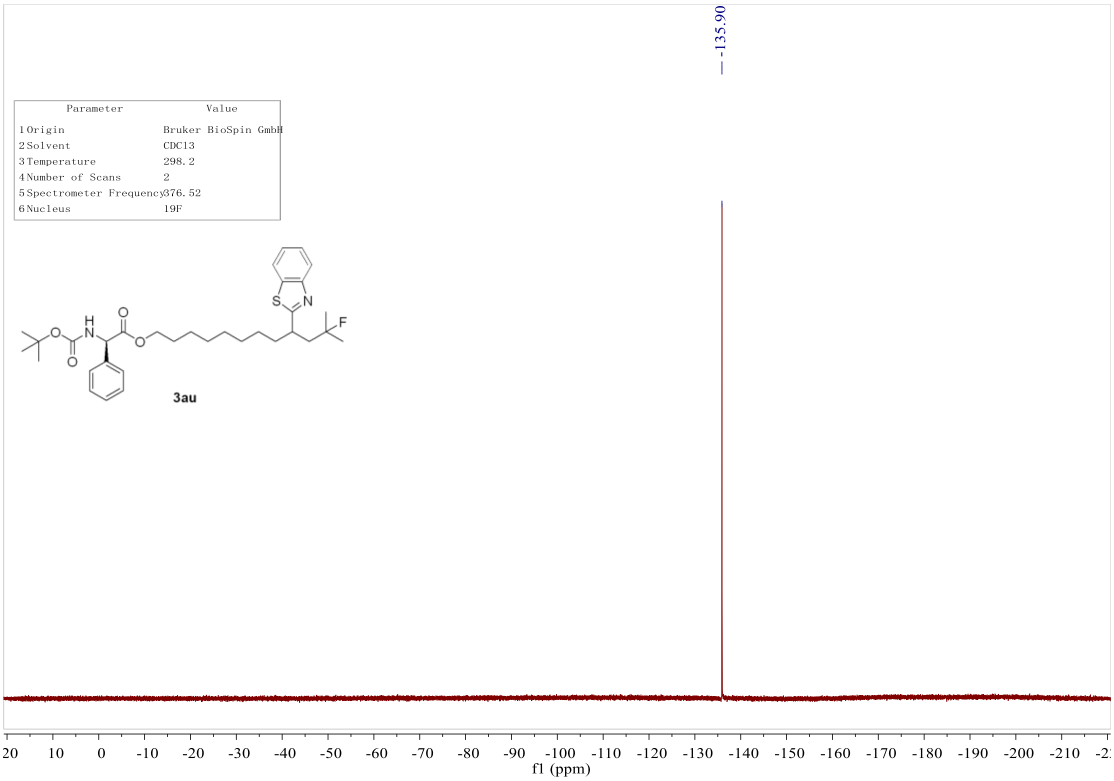

Supplementary Fig. 168 ^{19}F NMR spectra (376 MHz, $CDCl_3$, 25 °C) of **3au**

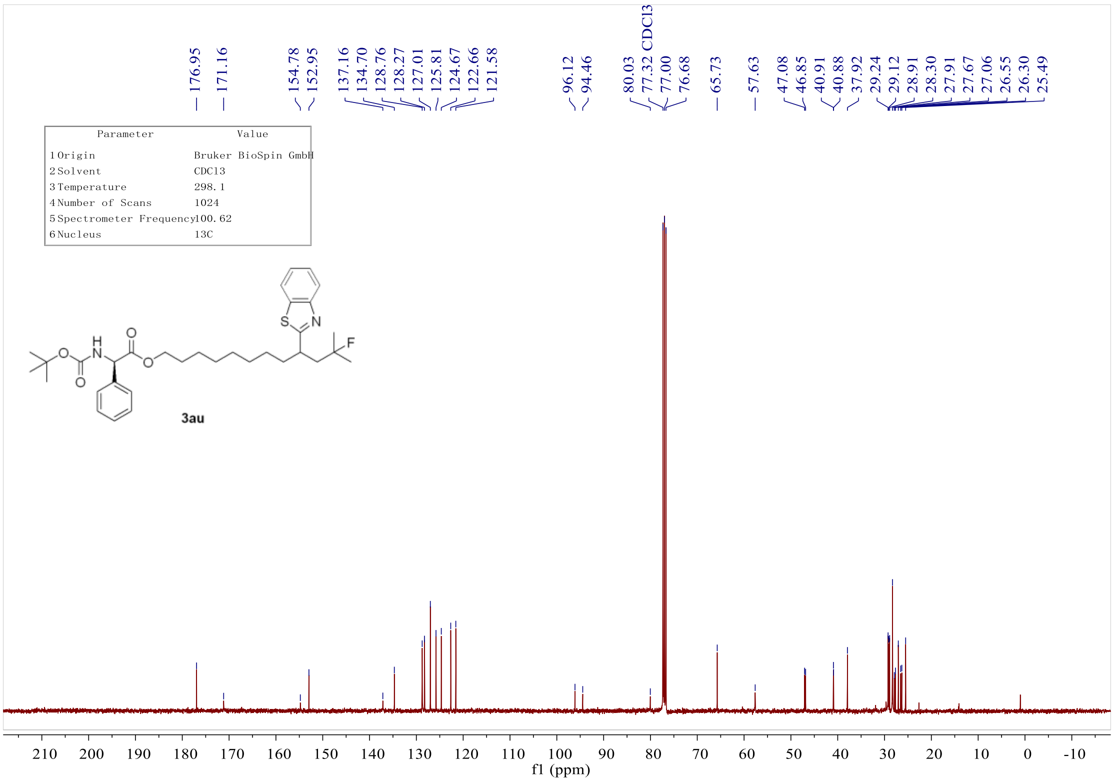

Supplementary Fig. 169 ^{13}C NMR spectra (100 MHz, $CDCl_3$, 25 °C) of **3au**

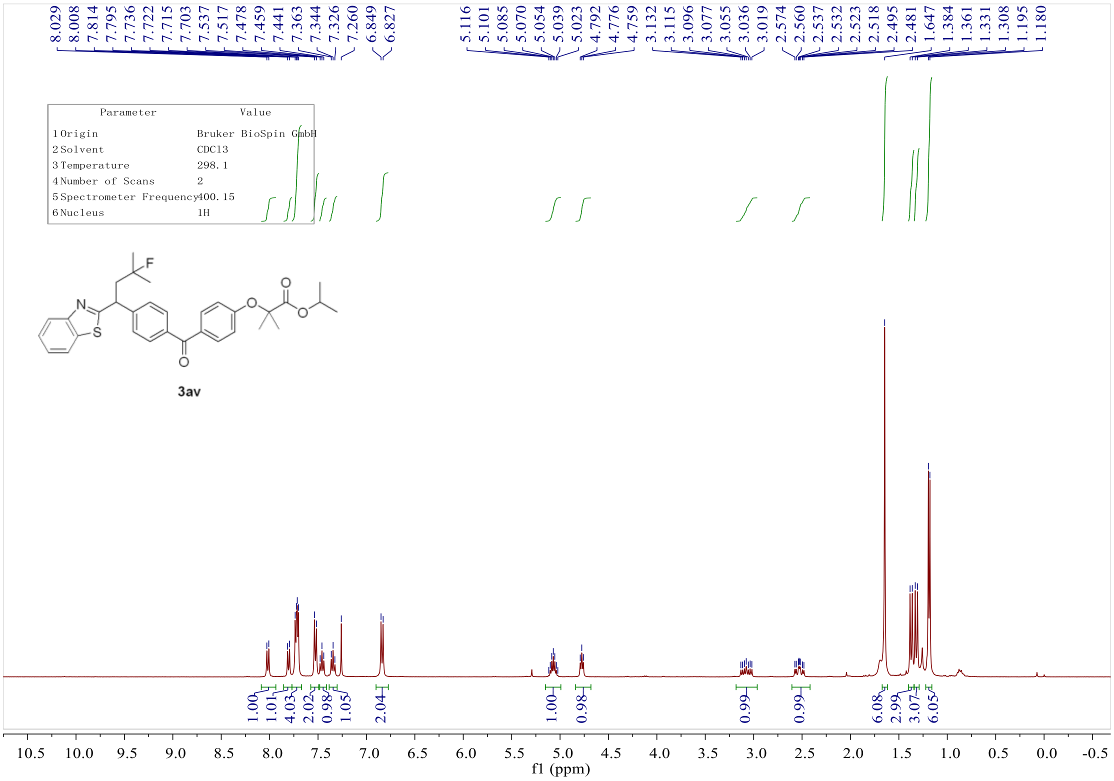

Supplementary Fig. 170 ^{1}H NMR spectra (400 MHz, $CDCl_3$, 25 °C) of **3av**

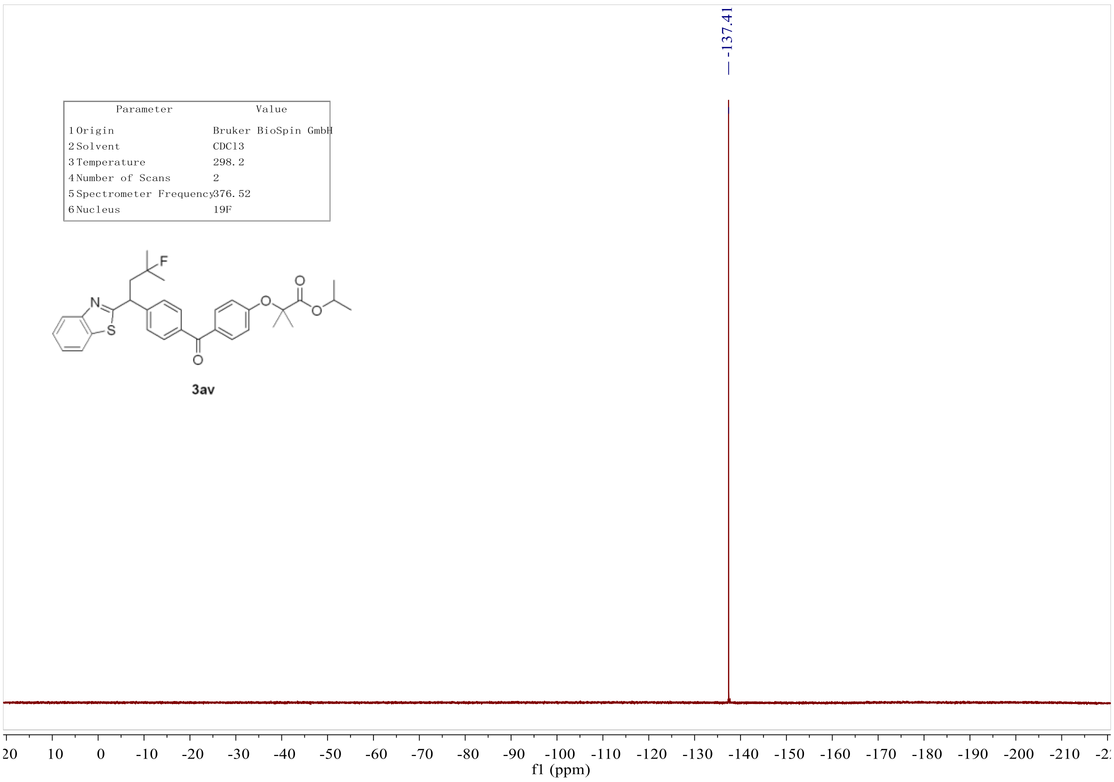

Supplementary Fig. 171 ^{19}F NMR spectra (376 MHz, $CDCl_3$, 25 °C) of **3av**

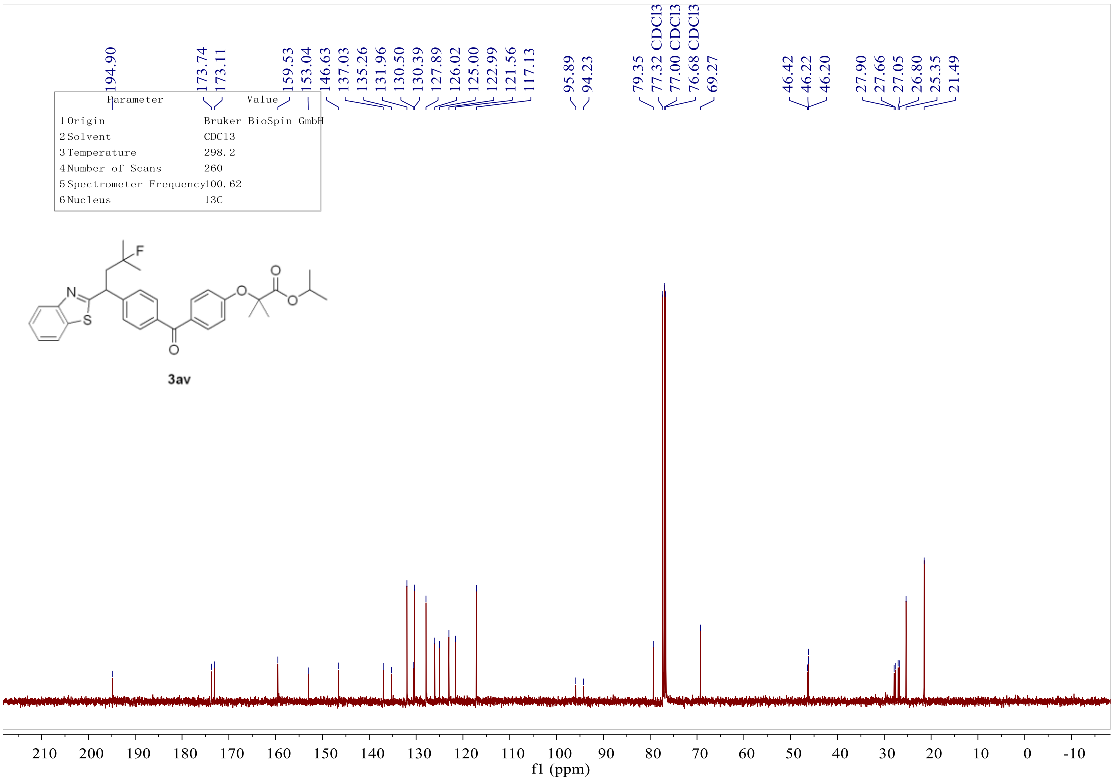

Supplementary Fig. 172 ^{13}C NMR spectra (100 MHz, $CDCl_3$, 25 ºC) of **3av**

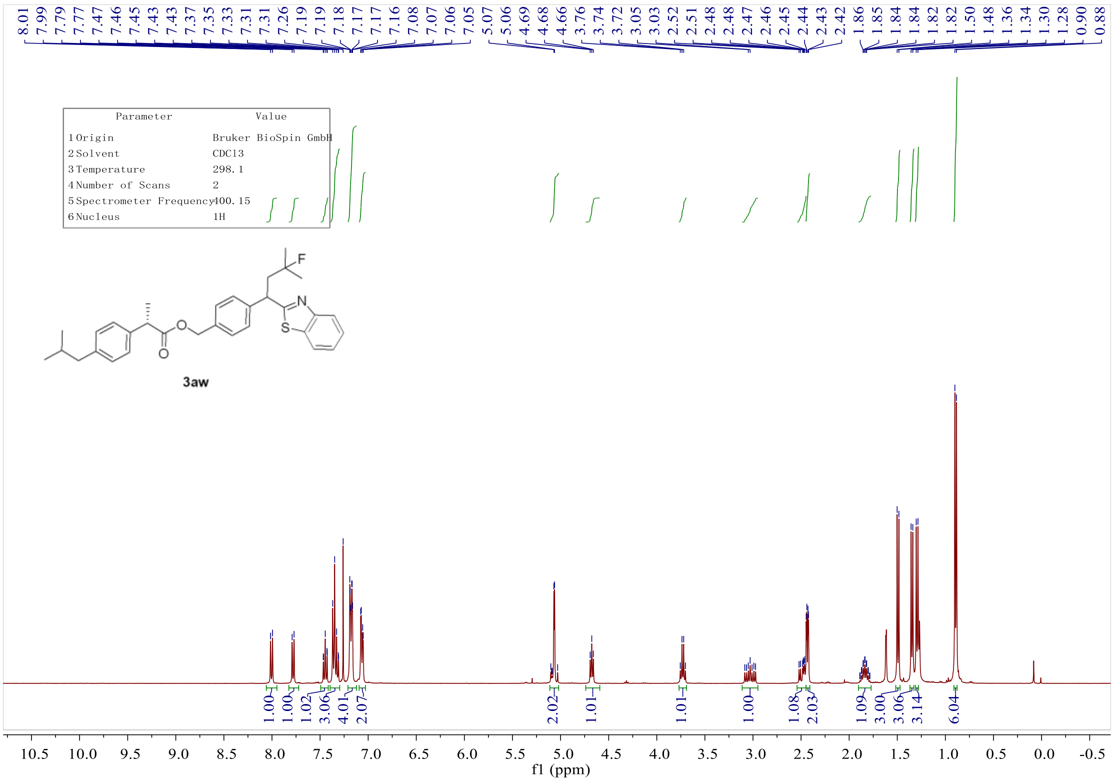

Supplementary Fig. 173 ^{1}H NMR spectra (400 MHz, $CDCl_3$, 25 ºC) of **3aw**

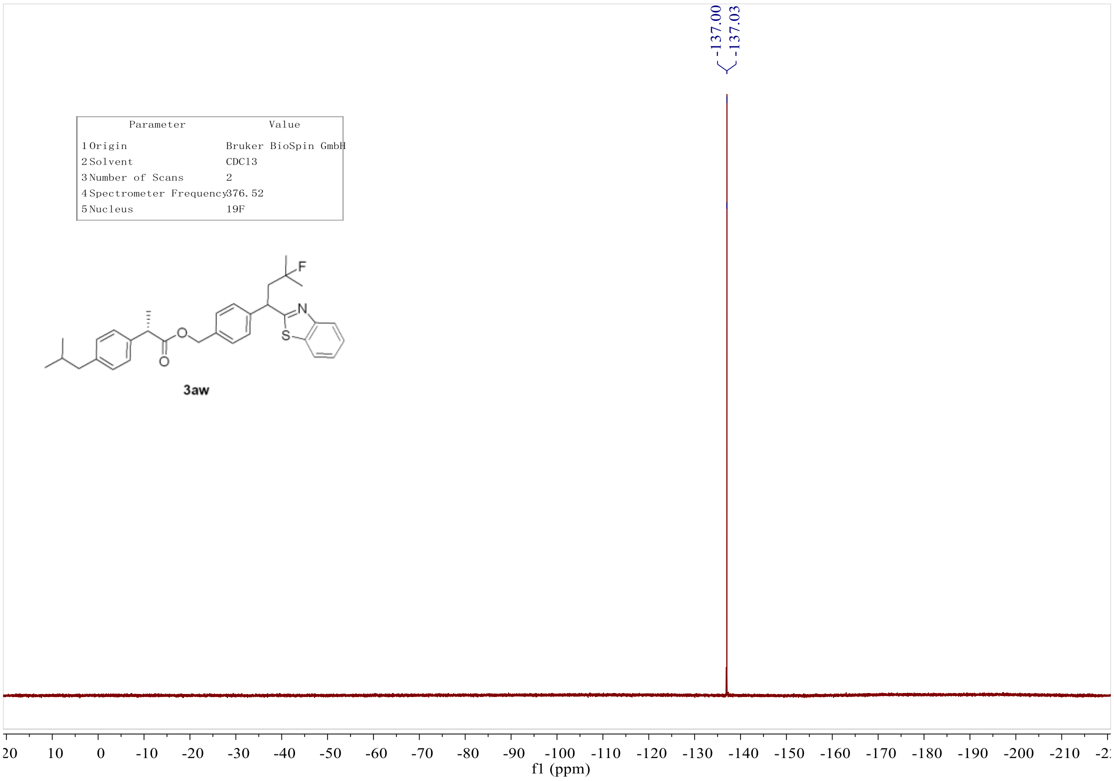

Supplementary Fig. 174 ^{19}F NMR spectra (376 MHz, $CDCl_3$, 25 ºC) of **3aw**

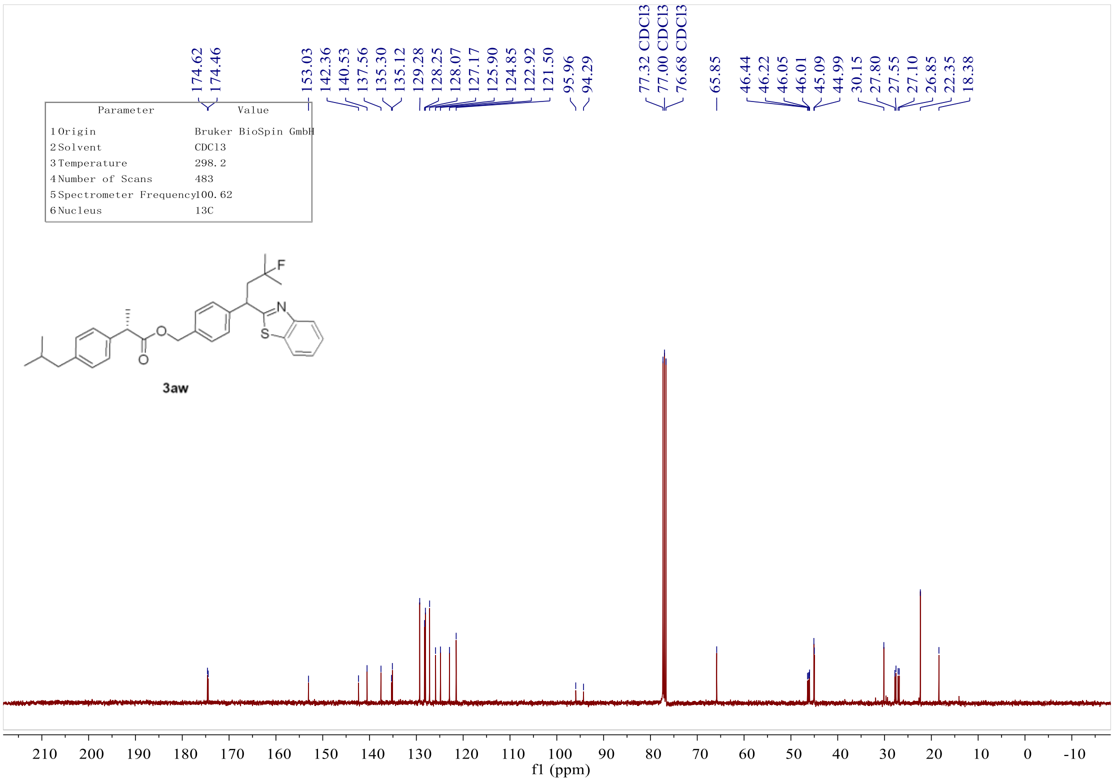

Supplementary Fig. 175 ^{13}C NMR spectra (100 MHz, $CDCl_3$, 25 ºC) of **3aw**

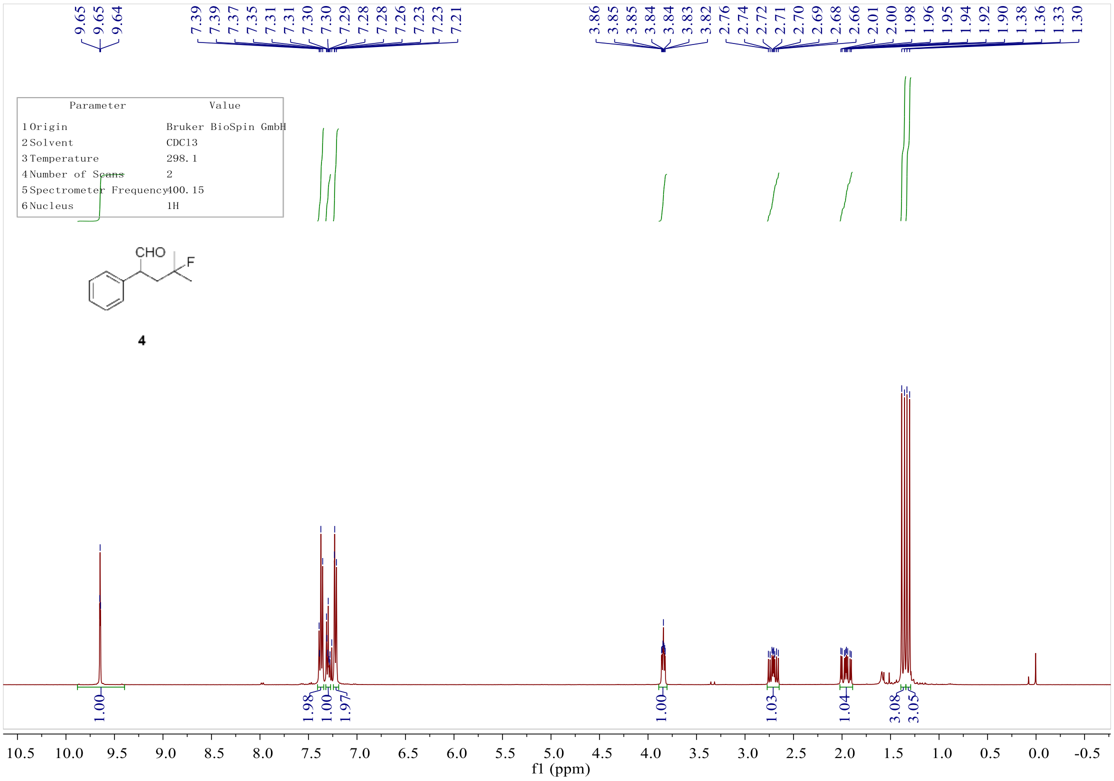

Supplementary Fig. 176 ^{1}H NMR spectra (400 MHz, $CDCl_3$, 25 ºC) of **4**

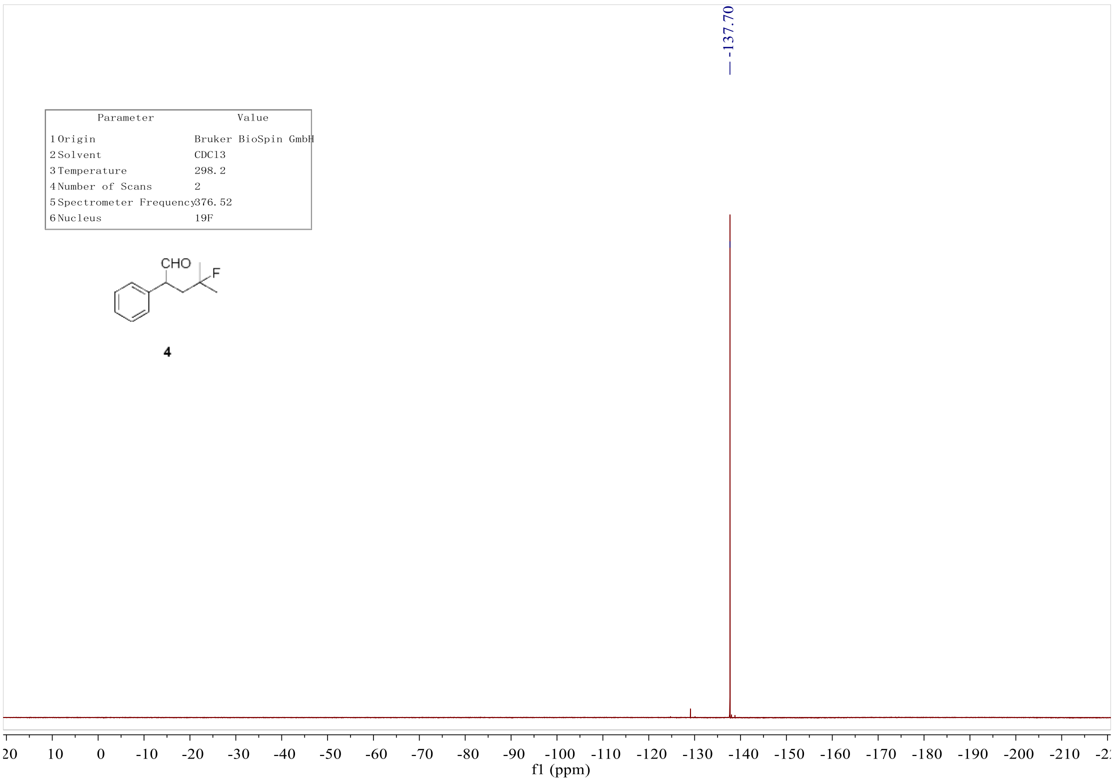

Supplementary Fig. 177 ^{19}F NMR spectra (376 MHz, $CDCl_3$, 25 ºC) of **4**

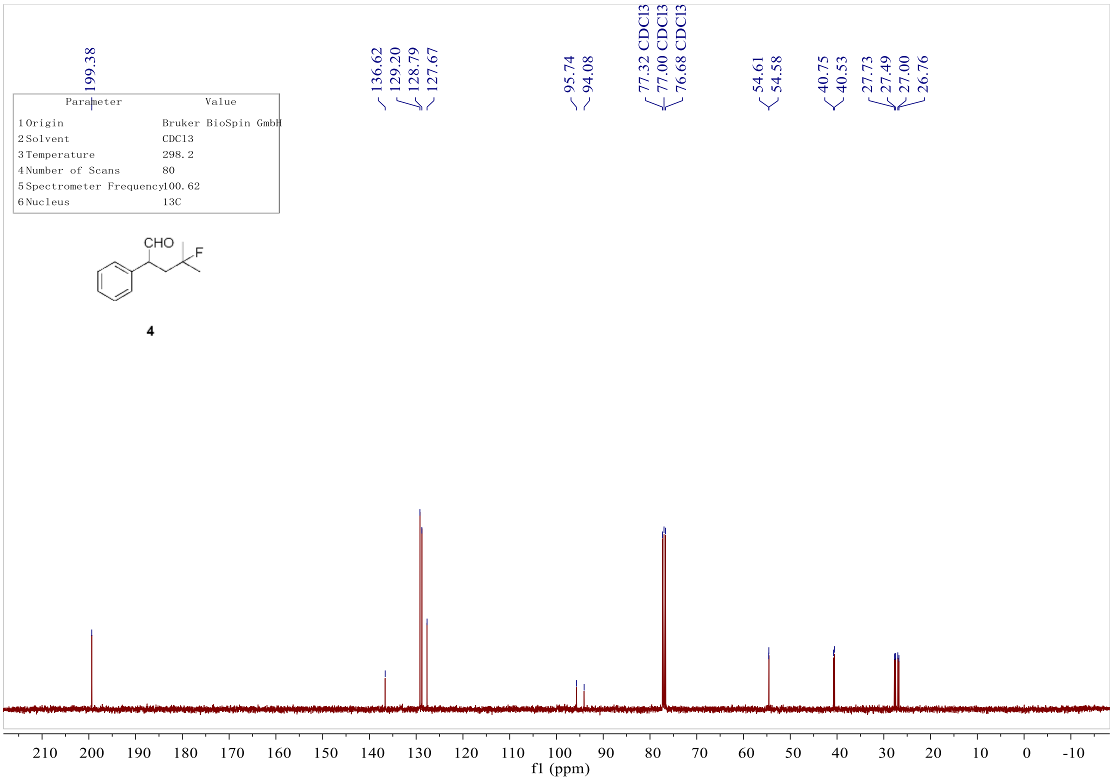

Supplementary Fig. 178 ^{13}C NMR spectra (100 MHz, $CDCl_3$, 25 °C) of **4**

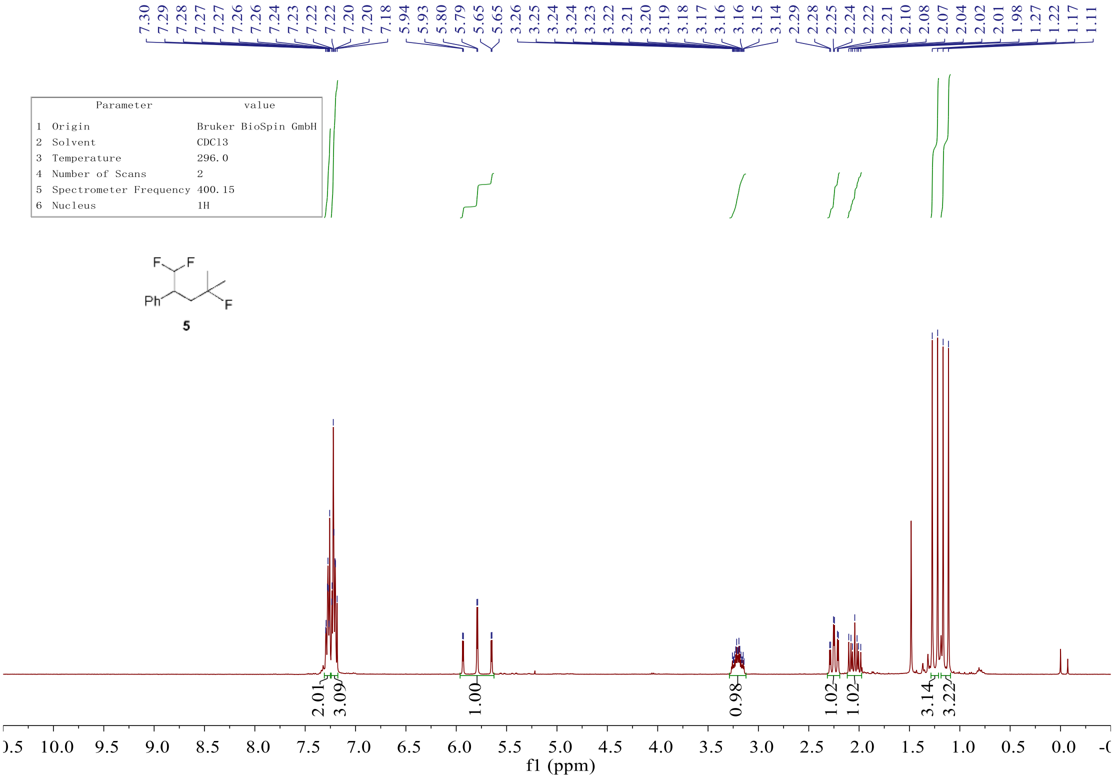

Supplementary Fig. 179 ^{1}H NMR spectra (400 MHz, $CDCl_3$, 25 °C) of **5**

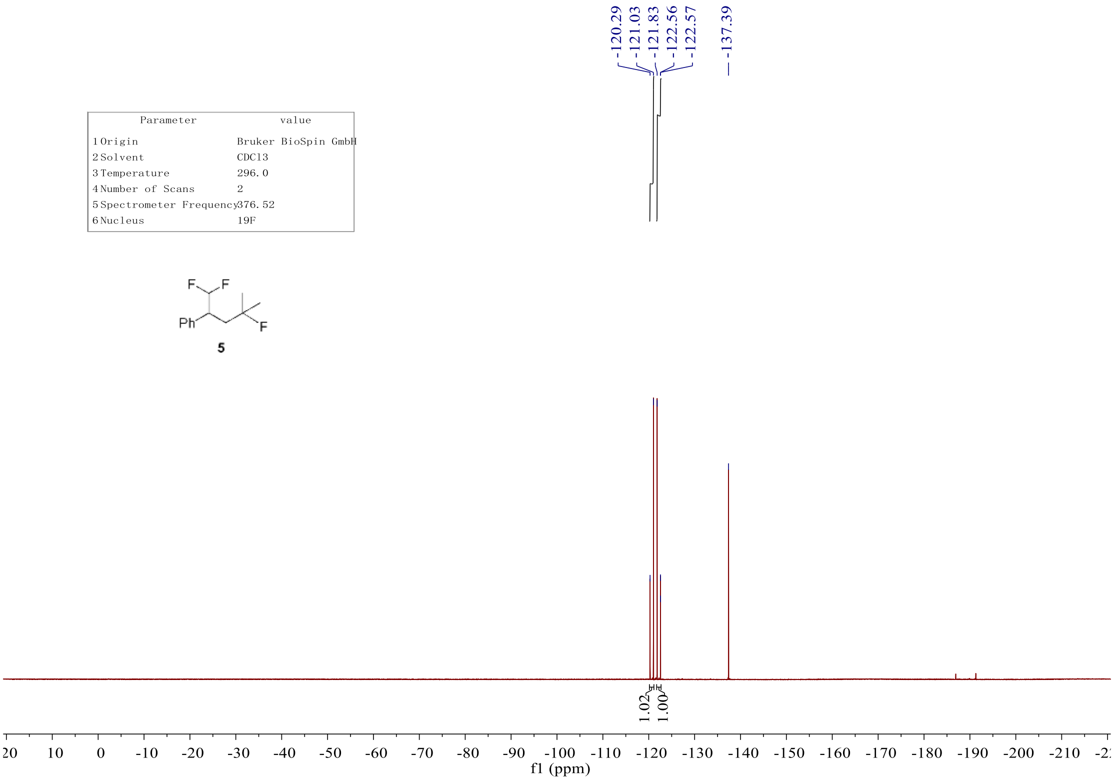

Supplementary Fig. 180 ^{19}F NMR spectra (376 MHz, $CDCl_3$, 25 °C) of **5**

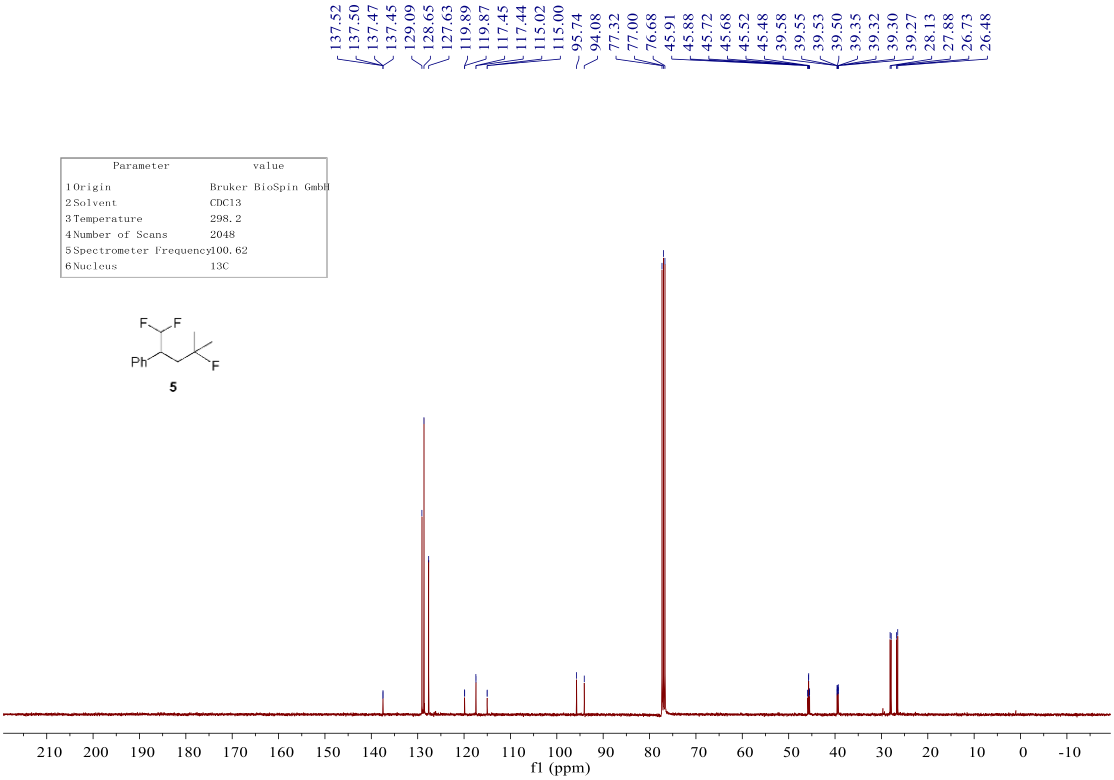

Supplementary Fig. 181 ^{13}C NMR spectra (100 MHz, $CDCl_3$, 25 °C) of **5**

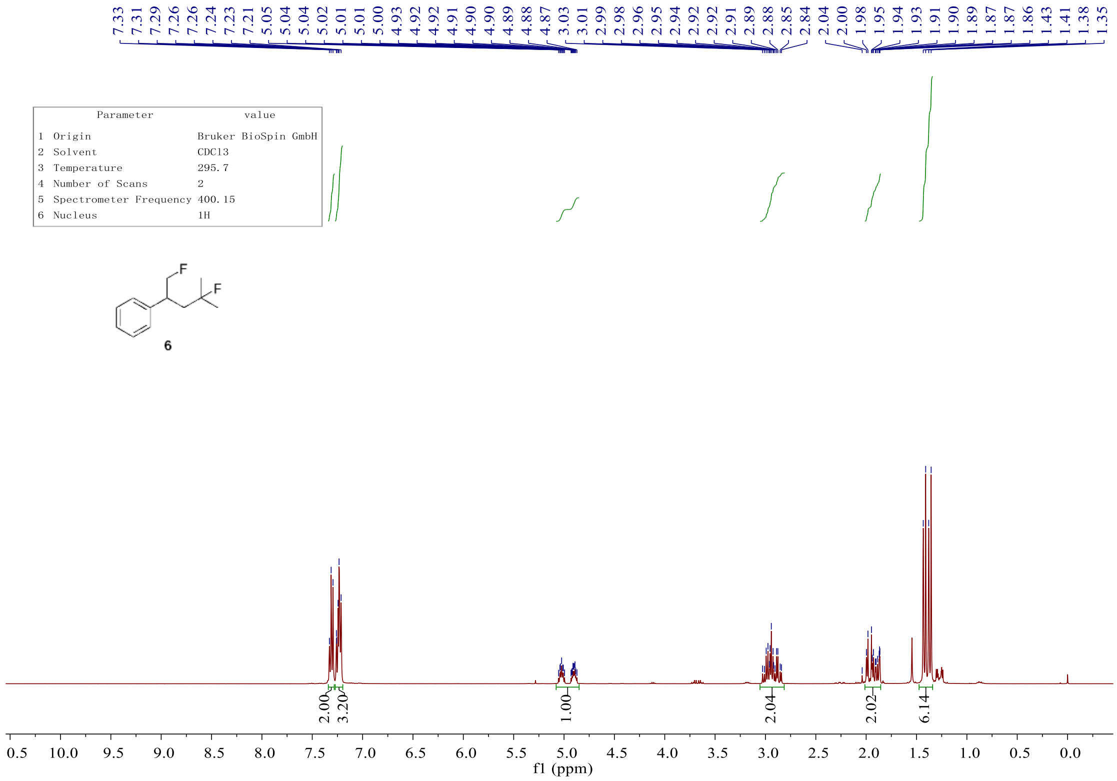

Supplementary Fig. 182 ^{1}H NMR spectra (400 MHz, $CDCl_3$, 25 ºC) of **6**

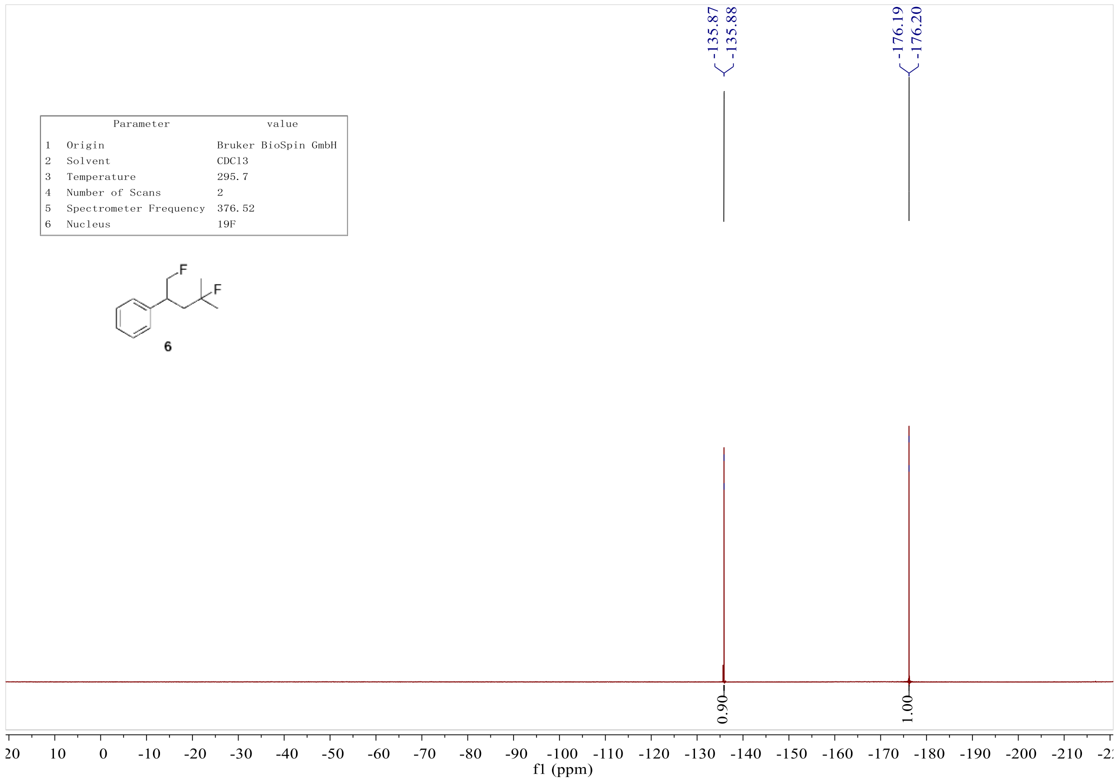

Supplementary Fig. 183 ^{19}F NMR spectra (376 MHz, $CDCl_3$, 25 ºC) of **6**

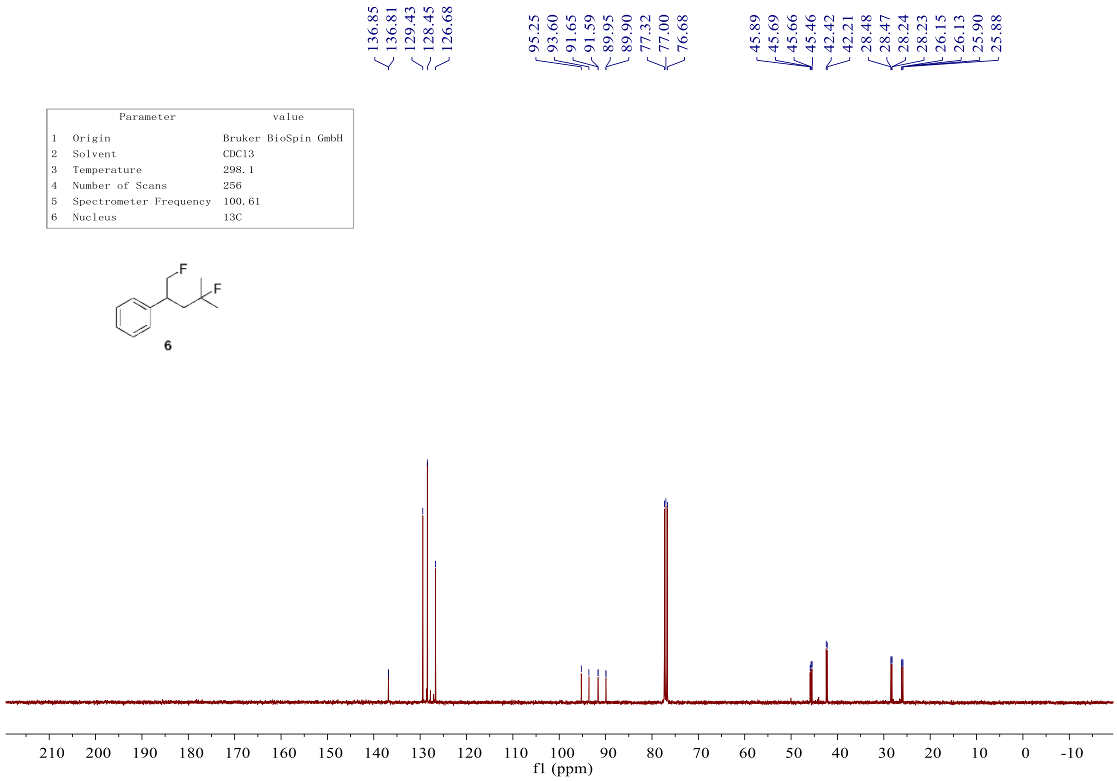

Supplementary Fig. 184 ^{13}C NMR spectra (100 MHz, $CDCl_3$, 25 °C) of **6**

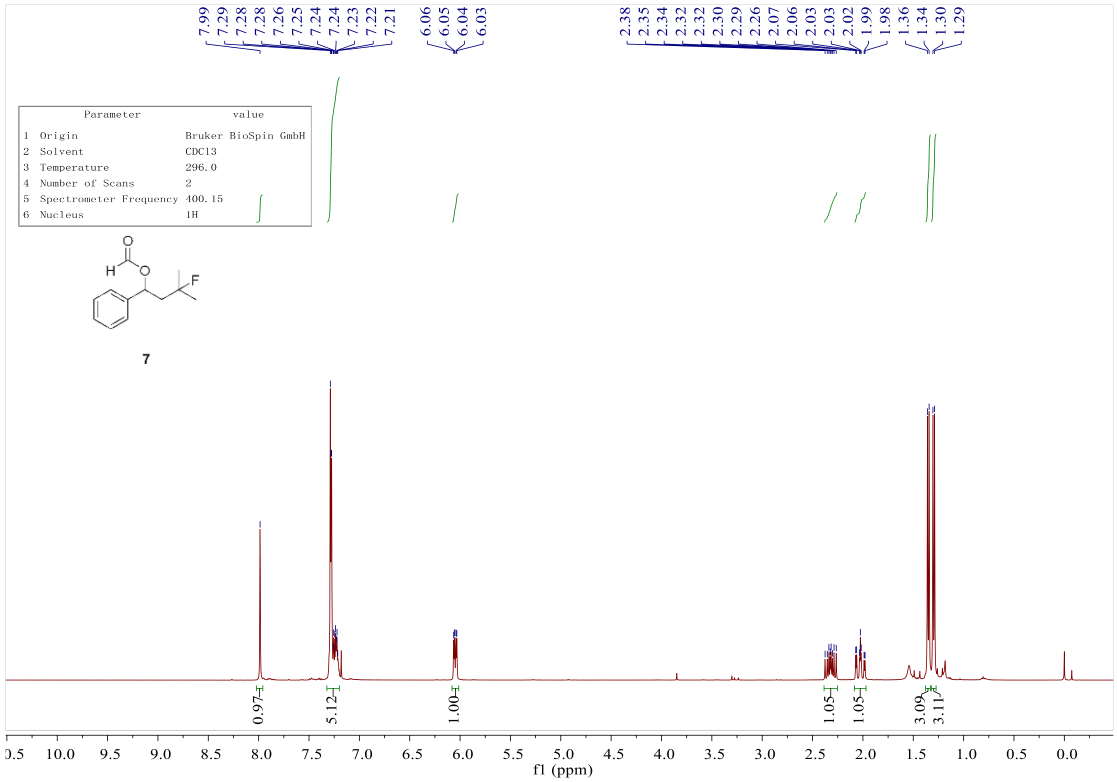

Supplementary Fig. 185 ^{1}H NMR spectra (400 MHz, $CDCl_3$, 25 °C) of **7**

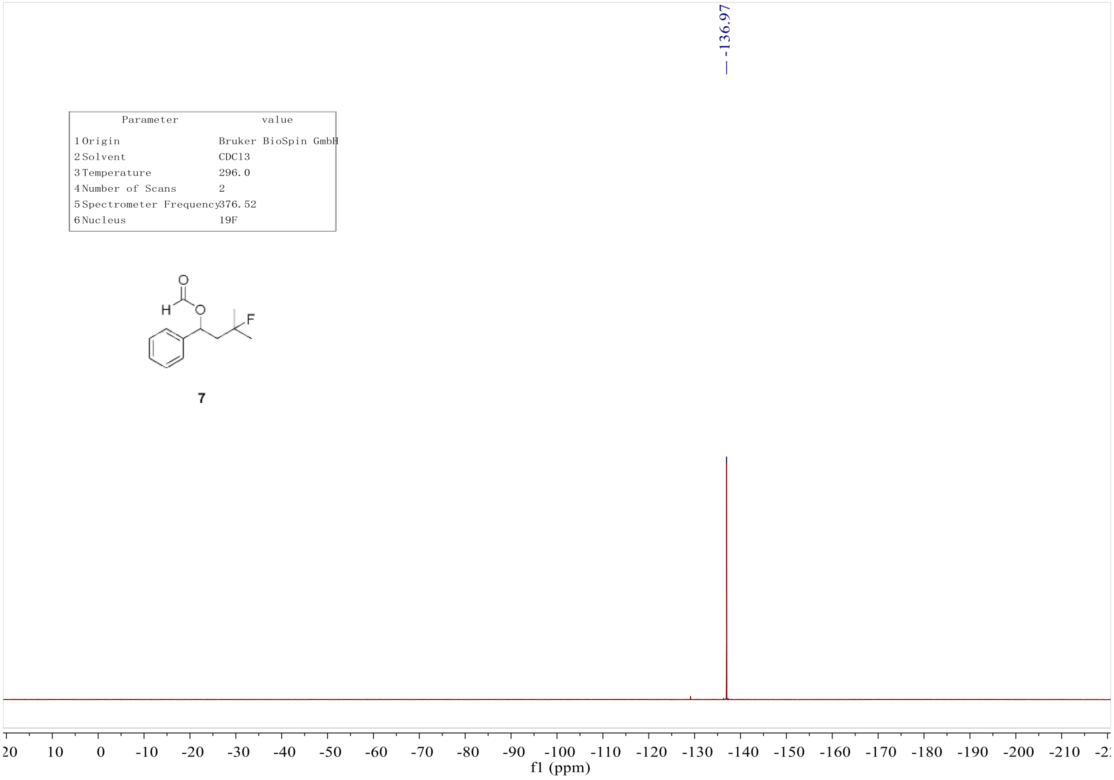

Supplementary Fig. 186 ^{19}F NMR spectra (376 MHz, $CDCl_3$, 25 ºC) of **7**

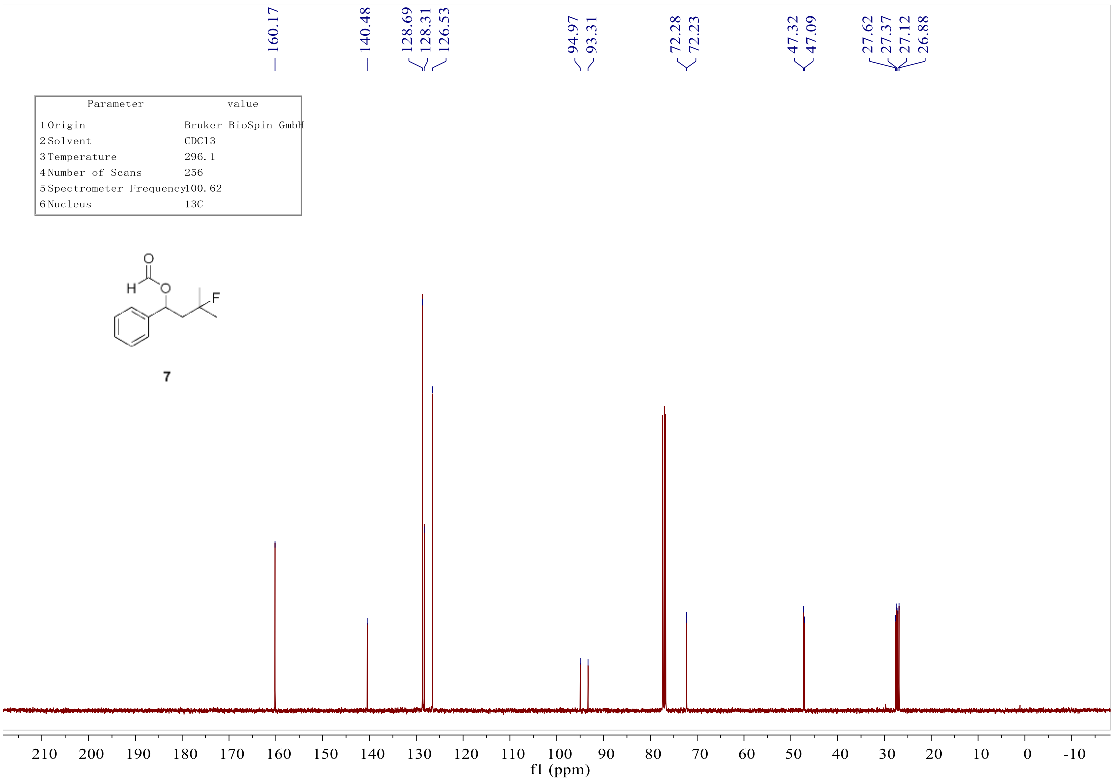

Supplementary Fig. 187 ^{13}C NMR spectra (100 MHz, $CDCl_3$, 25 ºC) of **7**

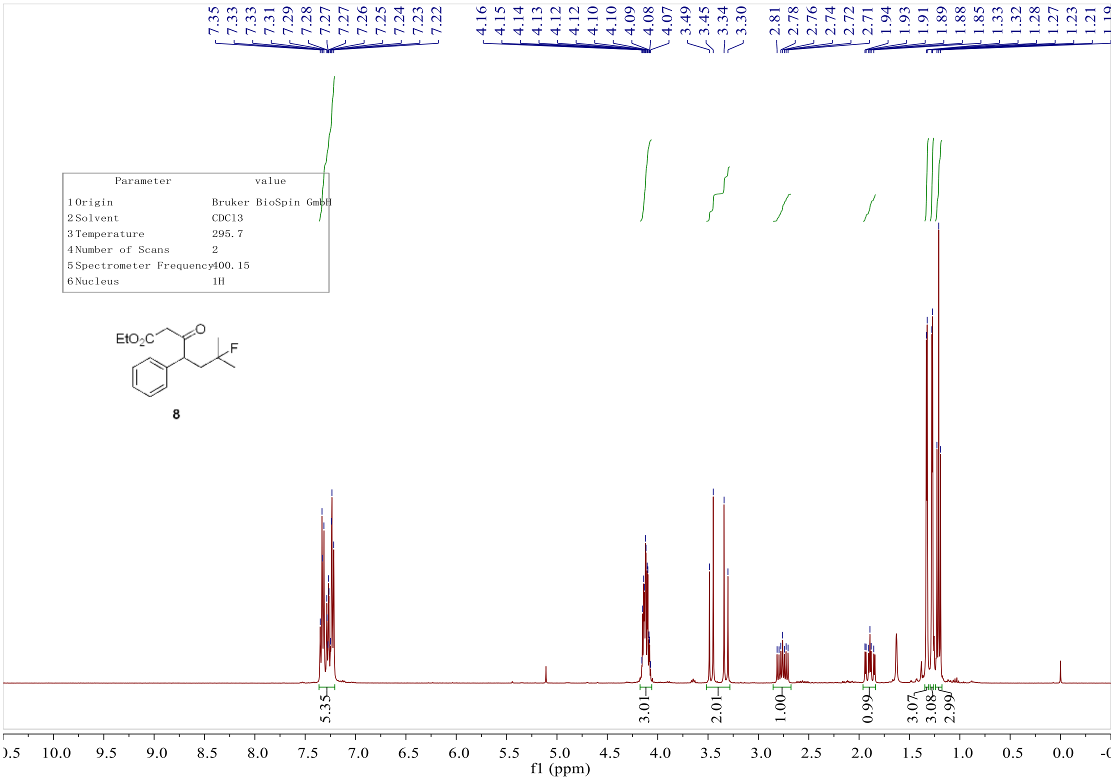

Supplementary Fig. 188 ^{1}H NMR spectra (400 MHz, $CDCl_3$, 25 °C) of **8**

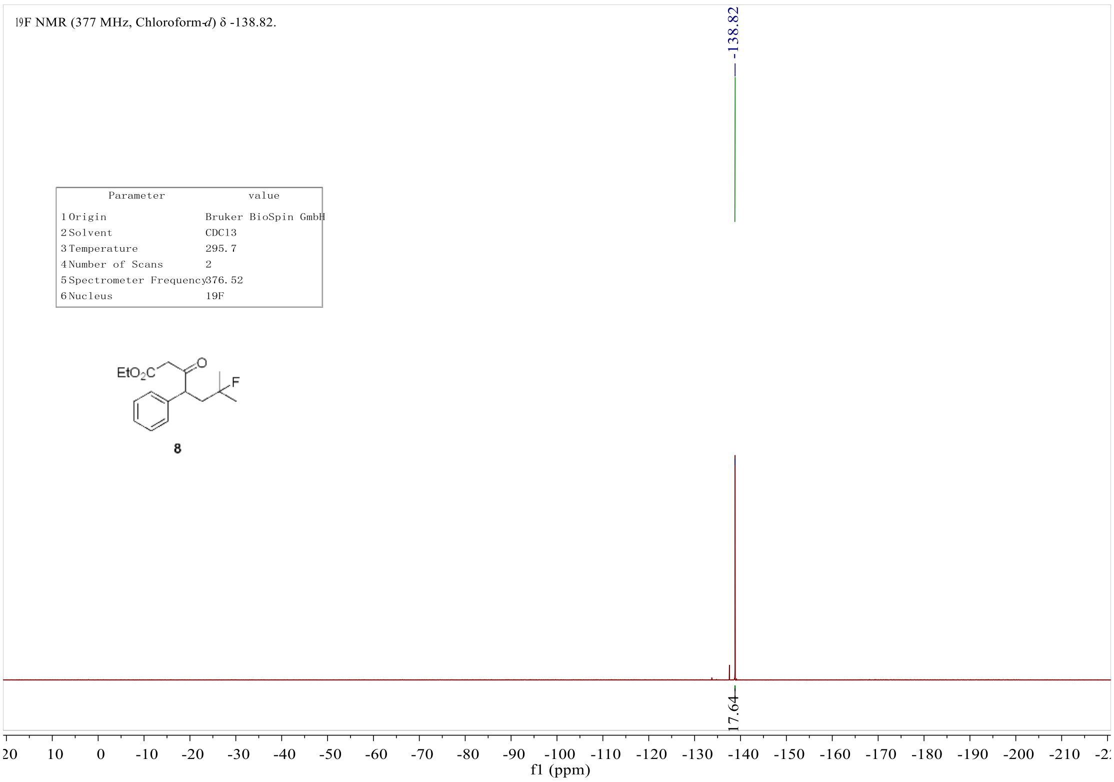

Supplementary Fig. 189 ^{19}F NMR spectra (376 MHz, $CDCl_3$, 25 °C) of **8**

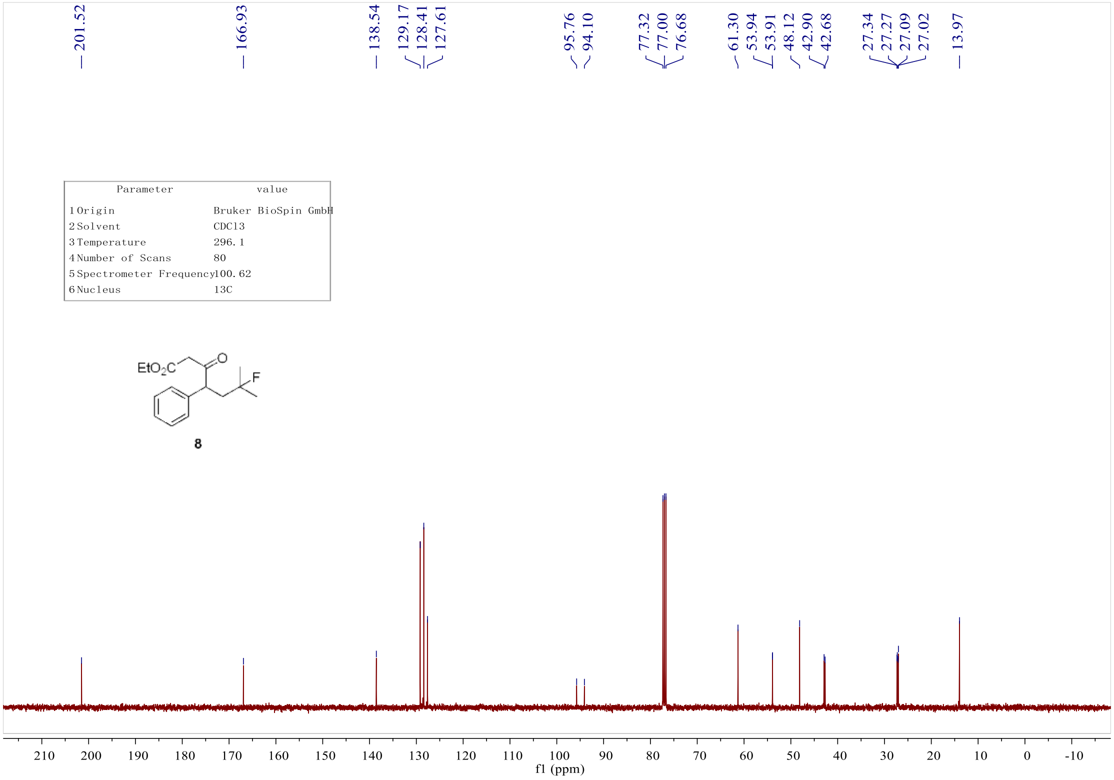

Supplementary Fig. 190 ^{13}C NMR spectra (100 MHz, $CDCl_3$, 25 °C) of **8**

10. References

1. Wu, X., Wang, M., Huan, L., Wang, D., Wang, J. & Zhu, C. Tertiary-alcohol-directed functionalization of remote C(sp^3)-H bonds by sequential hydrogen atom and heteroaryl migrations. *Angew. Chem., Int. Ed*. **57**, 1640-1644 (2018).

2. Sabol, J. S., Brake, N. W. & McDonald, I. A. Fluorinated amino acids Part 3: Synthesis of β-difluoromethyl-m-tyrosine. *Tetrahedron Lett.* **35**, 1821-1824 (1994).

3. Purrington, S. T. & Pittman, J. H. A new synthesis of alkyl fluorides. *Tetrahedron Lett.* **29**, 6851-6852 (1998).

4. Horn, A. & Kazmaier, U. Purified *m*-CPBA, a useful reagent for the oxidation of aldehydes. *Eur. J. Org. Chem.* **20**, 2531-2536 (2018).

5. Jeyakumar, K. & Chand, D. K. Molybdenum (VI) dichloride dioxide catalyzed synthesis of *β*-keto esters by CH insertion of ethyl diazoacetate into aldehydes. *Synthesis* **11**, 1685-1687 (2008).
